# Pd-catalyzed asymmetric allylic substitution cascade using α-(pyridin-1-yl)-acetamides formed *in situ* as nucleophiles†
†Electronic supplementary information (ESI) available: Experimental procedures and characterization data. CCDC 1860880. For ESI and crystallographic data in CIF or other electronic format see DOI: 10.1039/c8sc04626c


**DOI:** 10.1039/c8sc04626c

**Published:** 2018-12-04

**Authors:** Kun Yao, Qianjia Yuan, Xingxin Qu, Yangang Liu, Delong Liu, Wanbin Zhang

**Affiliations:** a Shanghai Key Laboratory for Molecular Engineering of Chiral Drugs , School of Pharmacy , Shanghai Jiao Tong University , 800 Dongchuan Road , Shanghai 200240 , P. R. China . Email: dlliu@sjtu.edu.cn ; Email: wanbin@sjtu.edu.cn; b School of Chemistry and Chemical Engineering , Shanghai Jiao Tong University , 800 Dongchuan Road , Shanghai 200240 , P. R. China

## Abstract

A Pd-catalyzed asymmetric allylic substitution cascade reaction, using α-(pyridin-1-yl)-acetamides (formed *in situ*) as nucleophiles, has been developed, generating chiral piperidine-containing amino acid derivatives *via* a one-pot procedure in high yields and with up to 96% ee. The products can be easily converted into potential bioactive compounds, unnatural chiral amino acids and dipeptides.

## Abstract 2

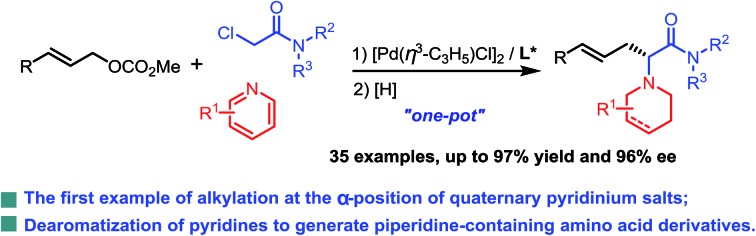
A Pd-catalyzed asymmetric allylic substitution cascade reaction, using α-(pyridin-1-yl)-acetamides (formed *in situ*) as nucleophiles, has been developed.

## 

Piperidines are among the most prevalent nitrogen ring systems found in numerous bioactive compounds and natural products.^1^ They are also prevalent in many well-selling pharmaceuticals, such as donepezil, methylphenidate, raloxifene, risperidone and paliperidone.^2^ Although several strategies have been developed to obtain piperidines,^3^ the activation of easily accessible pyridine ring systems followed by functionalization is considered to be the most efficient methodology.^4^

Quaternary pyridinium salts **A** (Fig. 1), generally used as activated pyridines, are very attractive synthons for synthetic chemists because of their ease of preparation and unique reactivity.^5^–^8^ They have received significant interest for use in many types of transformation, for example, Michael additions,5*a* 1,3-dipole additions,5*b* Kröhnke reaction,5*c**etc.* Therefore, quaternary pyridinium salts **A** are widely applied in the synthesis of heterocyclic compounds. From the viewpoint of functionalization, **A** can roughly be divided into three activated types: ketones **A1**,^6^ esters **A2**^7^ and amides **A3** (Fig. 1),^8^ which have been applied in the synthesis of anti-mycobacterial compounds,6*a* NF-κB inhibitors7*a* and 5-HT2c modulators,8*a* respectively. The main method to prepare these heterocyclic compounds is *via* functionalization of the α-positions of **A1**, **A2** and **A3**. To the best of our knowledge, however, alkylation at the α-position of the aforementioned quaternary pyridinium salts *via* a substitution reaction, has not yet been developed.

**Fig. 1:**
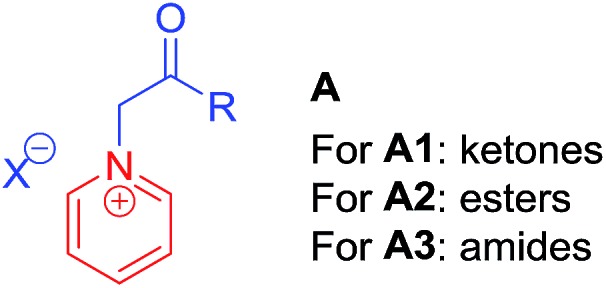
Generally used pyridine quaternary salts.

Pd-catalyzed allylic substitution is a powerful synthetic tool for the formation of C–C and C–X bonds (X = N, O, S, *etc.*).^9^ Our group has developed several novel Pd-catalyzed asymmetric alkylations for the construction of biologically-active chiral molecules with excellent catalytic behavior,^10^ particularly using several activated nucleophiles formed *in situ* by either an organic10a–d or transition-metallic catalyst.10i–m Herein, we disclose the use of pyridine quaternary salts **A**, formed *in situ* from pyridines and haloacetamides, as novel activated nucleophiles, allowing for the preparation of piperidine-containing amino acid derivatives easily *via* a cascade process (Scheme 1). Differing from previously reported methodologies in which the pyridine ring of the activated species **A** is generally removed or transferred to a fused aromatic ring moiety in the final products,^5^–^8^ in our methodology the pyridine rings were reduced to piperidine groups in the products. The obtained *N*-azacyclic (*e.g.* piperidyl) substituted α-amino acid structural motif is present in numerous bioactive compounds, natural products and medicines.3a,3c,^11^

**Scheme 1:**
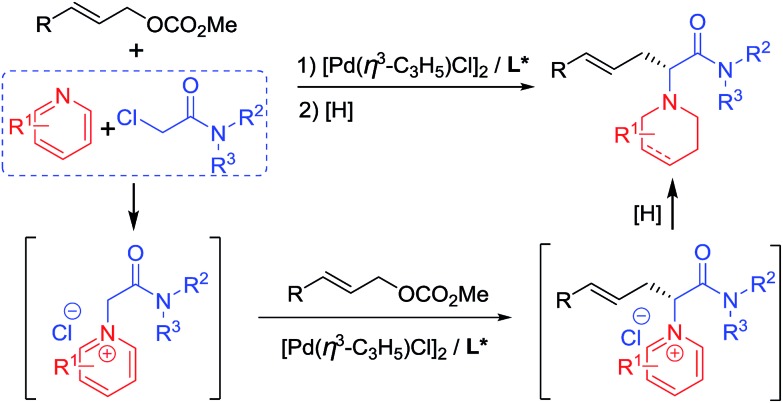
Pd-catalyzed asymmetric allylic substitution cascade.

Initially, we carried out the reaction by using both ketones **A1** and esters **A2** as nucleophiles. However, only racemic products were obtained albeit in high yields, probably due to the strong acidity of the α-hydrogen.^12^ Therefore, pyridine quaternary salt **A3**, bearing a less acidic α-hydrogen than that of **A1** and **A2**, was used, providing the desired products with excellent catalytic behaviour. After a dearomatizing reduction, a series of piperidine-containing amino acid derivatives can be obtained in high yields and with excellent enantioselectivities (Scheme 1).

Our study began with the reaction of cinnamyl acetate (**1a**), 2-chloro-*N*,*N*-dibutylacetamide (**2a**) and pyridine (**3a**) using a catalytic system consisting of [Pd(*η*^3^-C_3_H_5_)Cl]_2_ and planar chiral phosphino-oxazoline ligands **L1**^13^ under a nitrogen atmosphere at 20 °C over 12 h. Unfortunately, no reaction occurred because chloroacetamide is too inert to form quaternary pyridinium salts. Next, the more reactive 2-bromo-*N*,*N*-dibutylacetamide (**2b**) was employed. To our delight, the reaction proceeded smoothly and the desired product **4a** was formed in 92% yield but with low enantioselectivity (Table 1, entry 1).^14^ Next, planar chiral ligands **L2** (entry 2) and **L3** (entry 3) were also employed, and somewhat lower yields and enantioselectivities were obtained compared with that of **L1** (entry 1). However, no reaction occurred when *t*-Bu-PHOX was used in place of the above planar chiral ligands (entry 4). After screening the effects of base and solvent on the reaction, it was found that the use of ferrocenyl ligand **L1** and 1,1,3,3-tetramethylguanidine (TMG) in a mixed solvent system of dioxane/DMSO (10/1, v/v) provided the desired product **4a** in 92% yield and 77% ee (Table 1, entry 1). Then, several additives, such as CsF, LiCl, LiBr and LiI were employed to improve the yield and enantioselectivity of the alkylated product (entries 5–8).10f,^15^ The reaction with LiI as an additive gave **4a** in 94% yield and 91% ee (entry 8). Next, allyl substrates bearing OCO_2_Me (**1b**) and OBoc (**1c**) as leaving groups were examined (entries 9 and 10). It was found that **1b** bearing OCO_2_Me as a leaving group provided better results (92% yield and 94% ee) than that of **1a** and **1c**. Finally, we lowered the reaction temperature in order to further improve the enantioselectivity. To our delight, the product **4a** was obtained in up to 96% yield and 95% ee when the reaction was carried out at 5 °C (entry 11). Further lowering the reaction temperature to 0 °C led to a sharp decline in yield because the solvent turned viscous at this temperature. Different substituents on the nitrogen atom of **2** were also examined and **2b** with two *n*-Bu groups gave the best results.^14^ In addition, since the use of LiI improved the enantioselectivitiy as mentioned above (entry 8) and because the use of LiI would most likely increase the activity of the chloroacetamide **2a**, we employed **2a** as a substrate again in place of **2b**. As expected, the desired product **4a** was obtained in excellent yield and enantioselectivity (entry 12). Therefore, **2a** was chosen for use in the subsequent reactions because it is less expensive and easy to access than **2b**.

**Table 1:** Optimization of the reaction conditions^*a*^

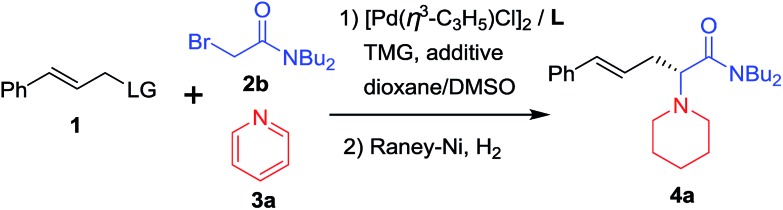						
Entry	L	LG of 1	Temp. (°C)	Additive	Yield^*b*^ (%)	ee^*c*^ (%)
1	**L1**	OAc	20	—	92	77
2	**L2**	OAc	20	—	90	41
3	**L3**	OAc	20	—	66	31
4	**L4**	OAc	20	—	NR	—
5	**L1**	OAc	20	CsF	95	41
6	**L1**	OAc	20	LiCl	93	55
7	**L1**	OAc	20	LiBr	93	71
8	**L1**	OAc	20	LiI	94	91
9	**L1**	OCO_2_Me	20	LiI	92	94
10	**L1**	OBoc	20	LiI	91	91
11	**L1**	OCO_2_Me	5	LiI	96	95
12^*d*^	**L1**	OCO_2_Me	5	LiI	96	95

^*a*^Reaction conditions: **1a** (0.1 mmol) with **2b** (0.2 mmol) and **3a** (0.5 mmol) in a mixed solvent of dioxane/DMSO (10/1, v/v) under nitrogen atmosphere with a catalytic system of [Pd(*η*^3^-C_3_H_5_)Cl]_2_ (2.5 mol%) and **L** (6.0 mol%) with TMG as a base (0.16 mmol) in the presence of an additive (0.1 mmol) at indicated temperature for 12 h; The pyridine quaternary salt was reduced by RANEY®Ni under hydrogen atmosphere.

^*b*^Isolated yields.

^*c*^Determined by HPLC using a chiral Daicel IC-3 column.

^*d*^Chloroacetamide **2a** was used.
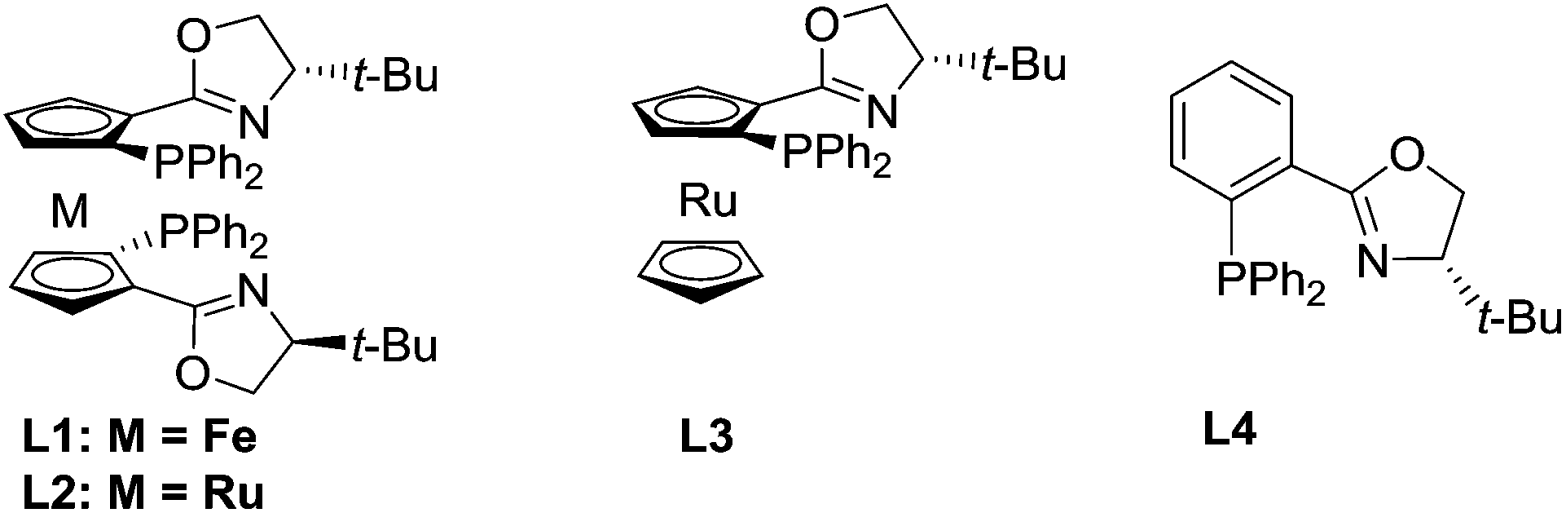

With the optimized reaction conditions in hand (Table 1, entry 9), substrates with different aryl substituents were explored (Scheme 2). First, cinnamyl carbonates bearing Me groups at the 2-, 3- and 4-positions of the phenyl ring were examined, and ees of approximately 90% were obtained for all substrates (**4b**, **4c** and **4d**). **4d** with an Me group at the 4-position of the phenyl ring provided the best enantioselectivity. When Me groups were replaced by OMe groups, nearly identical results were obtained and the substrate with an OMe group at the 4-position of the phenyl ring gave the best result (**4e**, **4f** and **4g**). Therefore, substrates with Et, *i*-Pr and *t*-Bu at 4-position of the phenyl ring were examined and all afforded satisfactory results (**4h**, **4i** and **4j**). Substrates bearing electron-withdrawing groups on the phenyl ring were next considered. When substrates with an F atom at the 2, 3- or 4-positions of the phenyl ring were used, the desired products were obtained in high yields and around 90% ees (**4k** and **4l***vs.***4m**). Similarly, **4m** with an F atom at the 4-position of the phenyl ring gave the best enantioselectivity. Therefore, substrates with electron-withdrawing groups, such as Cl, Br and CF_3_ at the 4-position of the phenyl ring, have been examined and all afforded promising results (**4n**, **4o** and **4p**). To our delight, **4p** bearing a CF_3_ group gave its corresponding product with 96% ee. When a Ph group was introduced, the reaction proceeded smoothly providing the corresponding product in 91% yield and 88% ee (**4q**). When the phenyl ring was replaced by a 2-naphthyl or 2-furyl group, the desired products were obtained in more than 90% yields and enantioselectivities for both substrates (**4r** and **4s**). An allylic substrate bearing 1,2-disubstituted groups was tested with the corresponding product **4t** being formed in 94% yield and 57% ee. Next, allyl carbonates without an Ar functional group or with alkyl groups instead of Ar groups were also subjected to the reaction conditions and the reductant was changed to NaBH_4_. The desired products bearing a tetrahydropyridine group (**5a–c**) were obtained in good yields and enantioselectivities. Two 1,1′-disubstituted allylic substrates bearing alkyl groups were also subjected to the reaction conditions, giving the desired products in moderate yields but with low ee (**5d** and **5e**). Finally, the *n*-Bu groups on the nitrogen atom of **2a** were replaced by two 4-pentenyl groups, with the desired product **5f** being obtained in excellent yield and enantioselectivity.

**Scheme 2:**
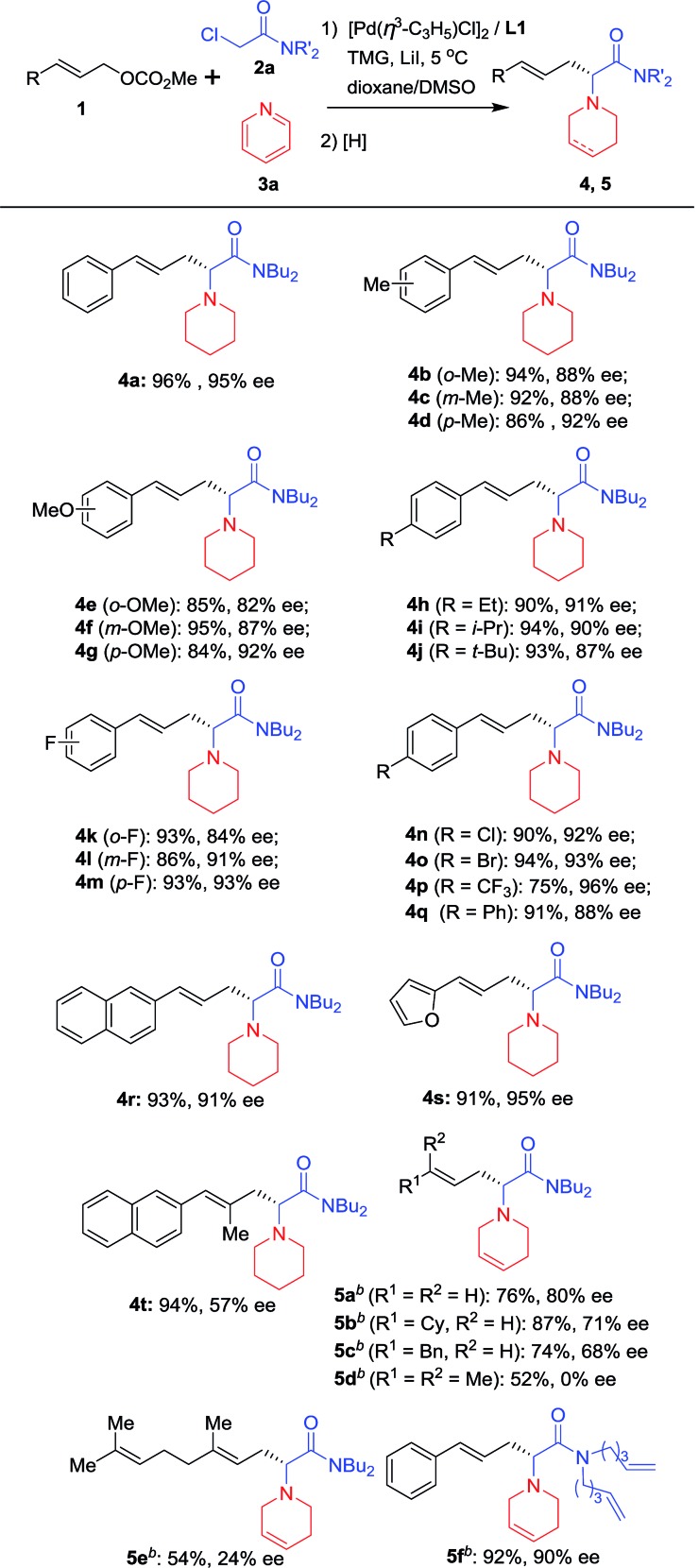
Scope of allylic substrates^*a*^. ^*a*^Using the optimal reaction conditions shown in Table 1. Isolated yields; ees were determined by HPLC using a chiral Daicel column. ^*b*^The pyridine quaternary salts were reduced with NaBH_4_.

To further explore the substrate scope, pyridine heterocycles **3** bearing different substituents were examined using NaBH_4_ as a reductant (Scheme 3). First, we found that when **3** possesses a Me group at the 4-position of the pyridine ring, the corresponding product is obtained in higher yield and enantioselectivity than when the Me group is present at the 3-position of the pyridine (**5g** and **5h**). Next, **3** bearing bulky alkyl or aryl groups substituted at the 4-position of the pyridine ring was used. The desired products were prepared in excellent yields and with around 90% ees (**5i–5l**). Finally, isoquinoline was used instead of pyridine and the corresponding dihydroisoquinoline derivative **5m** was obtained in good yield and enantioselectivity. **5m** contains a dihydroisoquinoline skeleton which is an important structural motif in alkaloid and biological molecules.^16^

**Scheme 3:**
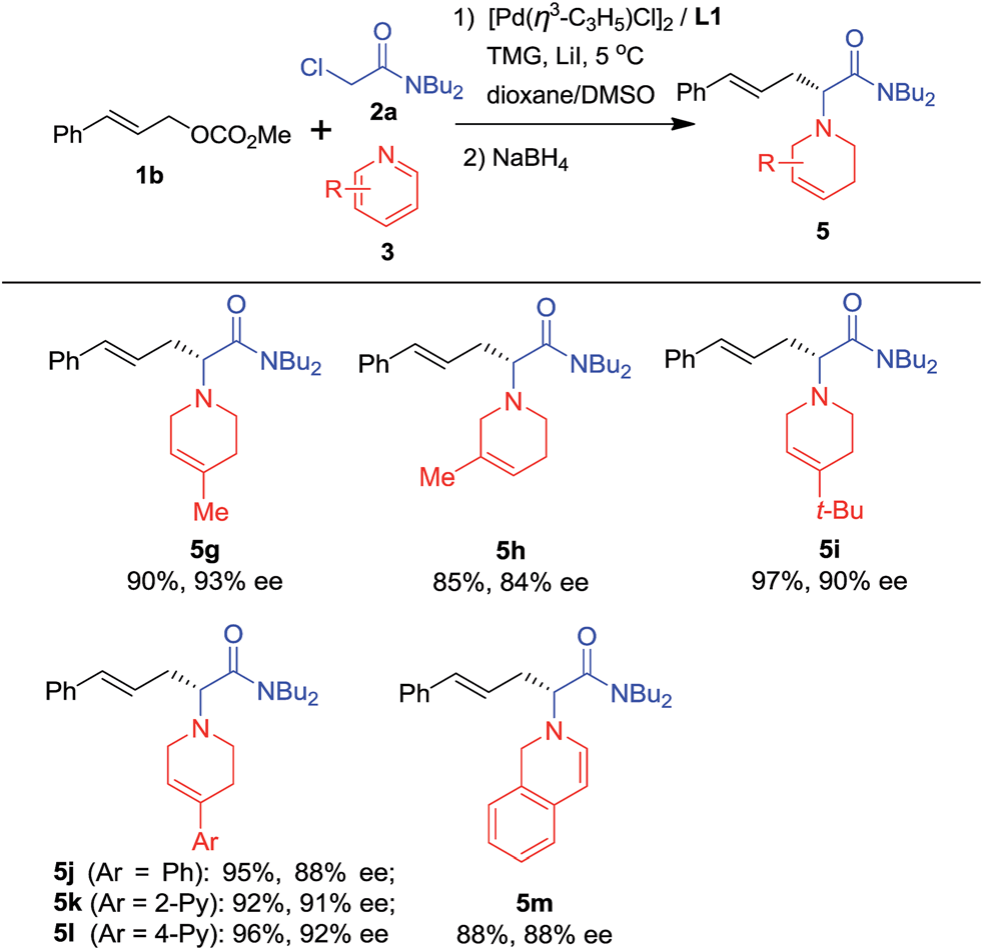
Scope of pyridines^*a*^. ^*a*^Using the optimal reaction conditions shown in Table 1. The pyridine quaternary salt was reduced with NaBH_4_. Isolated yields; ees were determined by HPLC using a chiral Daicel column.

To prove the utility of this new synthetic methodology, product **4a** was reduced with LiAlH_4_ to provide chiral vicinal diamine **6** in excellent yield (Scheme 4, eqn (1)). The hydrogenation of the double bond of the allylic motif in **4q** gave the product **7** in 83% yield without any loss in enantioselectivity (Scheme 4, eqn (2)). Derivatizations of **5f***via* ring closing metathesis were conducted, forming the corresponding macrocyclic amino acetamide **8** in excellent yield without any loss in ee (Scheme 4, eqn (3)).^17^ Product **5j**, with a phenyl group at the tetrahydropyridine ring, was oxidized to give the lactam **9** in excellent yield and with complete retention of configuration (Scheme 4, eqn (4)).^18^

**Scheme 4:**
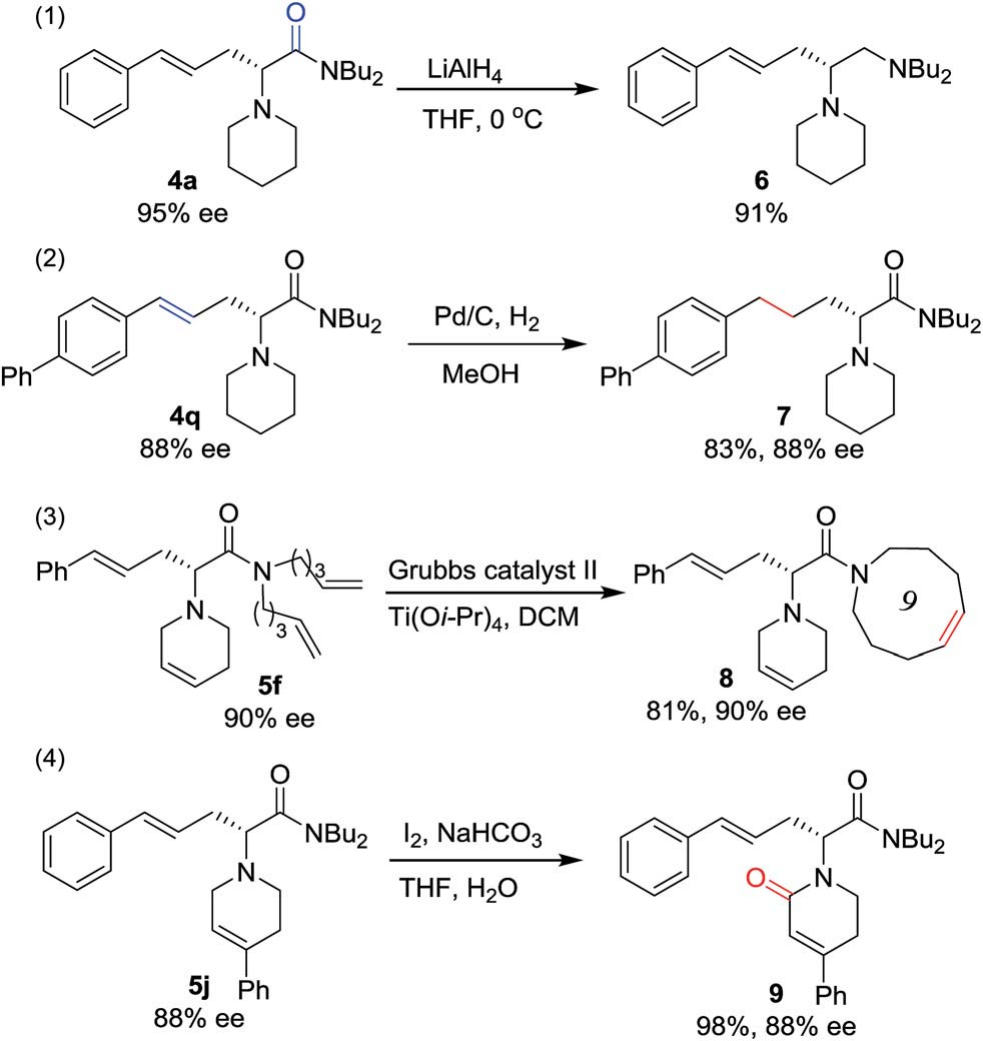
The transformation of **4** and **5**.

Reaction with chloroacetamide **2c** under standard conditions was also conducted. After removal of the DMB group, product **10** was obtained in 92% yield and 90% ee. Further hydrolysis of **10** under mild conditions^19^ gave the unnatural chiral amino acid (*R*)-**11**^20^ in good overall yield (Scheme 5, top). **10** was also treated with Lawesson's reagent and the corresponding amino thioacetamide derivative **12** was formed in 31% yield and 88% ee (Scheme 5, top). Unnatural amino acids are often used for polypeptide drug candidate optimization since the peptide bonds consisting of unnatural amino acids are more protease-resistant, which increases stability under physiological conditions and changes the log *D* value.^21^ Thus, bromoacetyl protected l-proline allyl ester **13** was prepared and applied in the asymmetric intramolecular allylic substitution cascade, affording the desired dipeptide **14** in high yield and diastereoselectivity (Scheme 5, bottom).

**Scheme 5:**
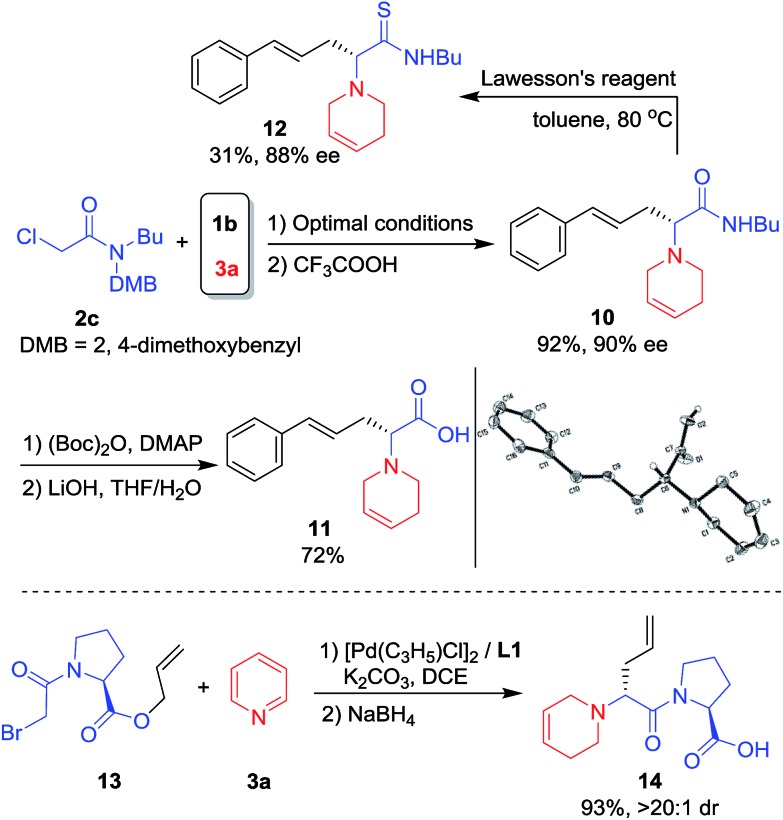
The synthesis of an unnatural amino acid and dipeptide.

## Conclusions

In summary, α-(pyridin-1-yl)-acetamides formed *in situ* have been used as nucleophiles in a Pd-catalyzed asymmetric allylic substitution cascade for the one-pot construction of chiral piperidine-based amino amides in high yields and with up to 96% ee. Using our methodology, several types of unnatural chiral amino acids and dipeptides containing piperidine or tetrahydropyridine substituents, which play an important role in natural products and medicines, have been prepared with excellent asymmetric behaviour.

## Conflicts of interest

There are no conflicts of interest to declare.

## Supplementary Material

Supplementary information

Crystal structure data
