# Supplementary material for: Pd-catalyzed asymmetric allylic substitution cascade using α-(pyridin-1-yl)-acetamides formed in situ as nucleophiles
Source: Chem Sci. 2018 Dec 4;10(6):1767–72. doi: 10.1039/c8sc04626c (PMC6369409; doi:10.1039/c8sc04626c)
Supplement: Supplementary file 1 [file SC-010-C8SC04626C-s001.pdf]

Electronic Supplementary Material (ESI) for Chemical Science.
This journal is © The Royal Society of Chemistry 2018

Supporting Information

Pd-Catalyzed asymmetric allylic substitution cascade using α-(pyridin-1-yl)-acetamides formed *in situ* as nucleophiles

Kun Yao,[a] Qianjia Yuan,[b] Xingxin Qu,[a] Yangang Liu,[a] Delong Liu,*[,a] and Wanbin Zhang*[a,b]

[a] Shanghai Key Laboratory for Molecular Engineering of Chiral Drugs, School of Pharmacy and

[b]School of Chemistry and Chemical Engineering, Shanghai Jiao Tong University, 800 Dongchuan Road, Shanghai 200240, P. R. China

Fax: (+)-86-21-54743265; Phone: (+)-86-21-54743265; E-mail: dlliu@sjtu.edu.cn; wanbin@sjtu.edu.cn

Contents

1. General Information S2
2. Preparation of 2-Haloacetamides (**2**) S2
3. Optimization of the Reaction Conditions S4
4. General Procedure: Pd-Catalyzed Three-Component Allylic Substitution S6
5. Derivatization of **4a, 4q, 5f, 5j** and **10** S19
6. Synthesis of Dipeptide and Unnatural Amino Acid S21
7. References S23
8. NMR Spectra S24
9. HPLC Data S76

1. General Information

All the reactions were monitored by TLC using UV light to visualize the course of reaction. Anhydrous THF, DME, Et_2O, 1,4-dioxane and toluene were prepared by distillation over sodium-benzophenone prior to use. ^1H, ^{19}F and ^{13}C NMR spectra were obtained using a Varian MERCURY plus-400 or Bruker 500 spectrometer with TMS as an internal standard. HRMS was performed on a Bruck solariX FTICR Mass Spectrometer at the Instrumental Analysis Center of Shanghai Jiao Tong University. Melting points were measured with SGW X-4 micro melting point apparatus. Cinnamyl carbonates **1** were prepared according to literature procedures.[1] All commercially available reagents were used as received.

2. Preparation of 2-Haloacetamides (2)

Preparation of 2-haloacetamides 2: To a solution of corresponding amine (10 mmol), triethylamine (2.02 g, 20 mmol) in DCM (50 mL) at 0 °C was added chloroacetyl chloride or bromoacetyl bromide (10 mmol) dropwise and stirred for 1 h. The solution was slowly warmed to room temperature and stirred overnight. The reaction mixture was diluted with DCM (100 mL), washed with brine, dried over Na_2SO_4 and then concentrated in vacuo. The residue was purified by column chromatography (PE/EtOAc = 10/1) to give the desired product. **2a**, **2b**, **2aa**, **2ab**, **2ac** and **2ad** were synthesized with this procedure and was consistent with the reported spectra[2]. The data of **2ae** and **2d** were summarized below.

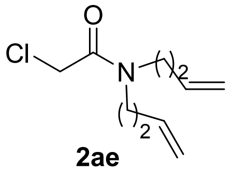

2ae

***N,N*-Di(but-3-en-1-yl)-2-chloroacetamide (2ae):** Yellow oil (1.68 g, 83%). ^1H NMR (400 MHz, $CDCl_3$): δ 5.86–5.65 (m, 2H), 5.19–4.96 (m, 4H), 3.82 (s, 2H), 3.46–3.21 (m, 4H), 2.44–2.19 (m, 4H); ^{13}C NMR (100 MHz, $CDCl_3$): δ 166.8, 134.8, 133.7, 118.2, 117.0, 48.5, 45.7, 33.1, 31.5, 26.2; IR (KBr) cm^{-1}: 3426, 2978, 1643, 1461, 1256, 918; HRMS (APCI) $[M+H]^+$ calcd 202.0993, found 202.0999.

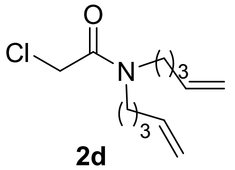

2d

2-Chloro-*N,N*-di(pent-4-en-1-yl)acetamide (2d): Yellow oil (1.72 g, 75%). ^1H NMR (400 MHz, $CDCl_3$): δ 5.86–5.69 (m, 2H), 5.12–4.88 (m, 4H), 4.03 (s, 2H), 3.39–3.18 (m, 4H), 2.15–1.95 (m, 4H), 1.75–1.59 (m, 4H); ^{13}C NMR (100 MHz, $CDCl_3$): δ 166.3, 137.9, 137.1, 116.1, 115.3, 47.8, 45.9, 41.5, 31.2, 30.9, 28.2, 26.6; IR (KBr) cm^{-1}: 3453, 2976, 1651, 1462, 1432, 912; HRMS (APCI) $[M+H]^+$ calcd 230.1306, found 230.1316.

Preparation of 2af:[3] A solution of *N*-butylidenebutan-1-amine (0.64 g, 5 mmol) in THF (10 mL) was stirred in a round bottom flask under a nitrogen atmosphere at room temperature. Then chloroacetyl chloride (0.57 g, 5 mmol) was added dropwise using a syringe. The mixture was stirred for 30 min at room temperature and then refluxed for a further 2 h. The reaction mixture

was concentrated in vacuo. The residue was purified by column chromatography (PE/EtOAc = 20/1) to give **2af** as a yellow oil.

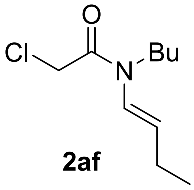

2af

***N*-(But-1-en-1-yl)-*N*-butyl-2-chloroacetamide (2af):** Yellow oil (0.78 g, 76%). ^{1}H NMR (400 MHz, CDCl$_3$, major rotamer): δ 6.36 (dt, J = 14.0, 1.6 Hz, 1H), 5.33–5.22 (m, 1H), 4.14 (s, 2H), 3.57 (t, J = 7.6 Hz, 2H), 2.15–2.04 (m, 2H), 1.40–1.25 (m, 4H), 1.10–0.92 (m, 6H); ^{1}H NMR (400 MHz, CDCl$_3$, minor rotamer): δ 7.07 (d, J = 14.0 Hz, 1H), 5.19–5.10 (m, 1H), 4.13 (s, 2H), 3.49 (t, J = 8.0 Hz, 2H), 2.15–2.04 (m, 2H), 1.40–1.25 (m, 4H), 1.10–0.92 (m, 6H); ^{13}C NMR (100 MHz, CDCl$_3$, major rotamer): δ 167.8, 126.1, 119.8, 44.5, 41.8, 29.1, 23.8, 20.3, 14.4, 14.0; ^{13}C NMR (100 MHz, CDCl$_3$, minor rotamer): δ 167.3, 124.9, 115.5, 45.5, 41.4, 30.3, 22.9, 20.8, 14.7, 14.1; IR (KBr) cm^{-1}: 3427, 2934, 1666, 1441, 1260, 912; HRMS (APCI) [M+H]$^+$ calcd 204.1150, found 204.1154.

Preparation of 2c and 11: To a solution of *N*-(2,4-dimethoxybenzyl)butan-1-amine or *L*-proline allyl ester hydrochloride (10 mmol) in water (5 mL) at 0 °C was added NaHCO$_3$ (2.52 g, 30 mmol) and the mixture was stirred for 10 min. Then, a solution of chloroacetyl chloride or bromoacetyl bromide (10 mmol) in toluene (5 mL) was added dropwise. The reaction was slowly warmed to room temperature and stirred for another 2 h. The reaction mixture was diluted with DCM (100 mL), washed with brine, dried over Na$_2$SO$_4$ and then concentrated in vacuo. The residue was purified by column chromatography to give the corresponding product.

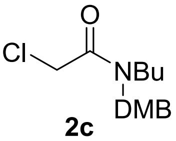

2c

***N*-Butyl-2-chloro-*N*-(2,4-dimethoxybenzyl)acetamide (2c):** Yellow oil (1.94 g, 65%). ^{1}H NMR (400 MHz, CDCl$_3$, mixture of two rotamers): δ 7.14 (d, J = 8.0 Hz, 0.3H), 6.94 (d, J = 8.0 Hz, 0.7H), 6.50–6.34 (m, 2H), 4.54 (s, 0.7H), 4.41 (s, 1.3H), 4.15 (s, 1.3H), 4.09 (s, 0.7H), 3.87–3.65 (m, 6H), 3.33–3.15 (m, 2H), 1.63–1.38 (m, 2H), 1.21–1.30 (m, 2H), 0.95–0.78 (m, 3H); ^{13}C NMR (100 MHz, CDCl$_3$, mixture of two rotamers): δ 166.9, 166.6, 161.0, 160.5, 158.6, 158.5, 130.5, 128.8, 117.6, 116.6, 104.5, 104.2, 98.9, 98.5, 60.5, 59.5, 55.6, 55.4, 47.5, 47.2, 45.9, 42.8, 41.9, 41.5, 31.0, 29.4, 21.2, 20.3, 14.0, 13.9; IR (KBr) cm^{-1}: 3011, 1749, 1670, 1653, 922; HRMS (APCI) [M+H]$^+$ calcd 300.1361, found 300.1378.

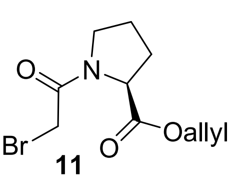

11

(*S*)-Allyl 1-(2-bromoacetyl)pyrrolidine-2-carboxylate (11): Yellow oil (2.41 g, 87%). ^{1}H NMR (500 MHz, CDCl$_3$, mixture of two rotamers): δ 6.06–5.79 (m, 1H), 5.47–5.15 (m, 2H), 4.74–4.49 (m, 3H), 4.05–3.51 (m, 4H), 2.39–1.86 (m, 4H); ^{13}C NMR (126 MHz, CDCl$_3$, major rotamer): δ 171.4, 165.3, 131.8, 118.5, 65.8, 59.3, 47.5, 29.2, 26.9, 24.9; ^{13}C NMR (126 MHz,

CDCl$_3$, minor rotamer): δ 171.2, 165.5, 131.2, 119.5, 66.4, 59.8, 47.0, 31.2, 27.0, 22.4; IR (KBr) cm^{-1}: 2958, 1743, 1648, 1418, 913; HRMS (APCI) $[M+H]^+$ calcd 276.0230, found 276.0256.

3. Optimization of the Reaction Conditions

Our study began with the reaction of cinnamyl acetate (**1a**), 2-chloro-*N,N*-dibutylacetamide (**2a**) and pyridine (**3a**) using a catalytic system consisting of $[Pd(\eta^3\text{-}C_3H_5)Cl]_2$ and a planar chiral phosphino-oxazoline ligand **L1** under a nitrogen atmosphere at room temperature for 12 h (Table S1). Unfortunately, no reaction occurred possible due to the low reaction activity for chloroacetamide. Then, 2-bromo-*N,N*-dibutylacetamide (**2b)** was employed instead of **2a** in the above reaction (entry 1). To our delight, the reaction processed smoothly with the desired product **4a** being obtained in 92% yield albeit with low enantioselectivity (entry 2). Subsequently, we examined the effect of solvent on the reaction. It was found that the reaction in protic alcohol solvents presented high yields but with low enantioselectivities (entries 1–4). Therefore, aprotic solvents such as toluene, THF, dioxane was examined and only trace amount of products were formed but with around 40% ee (entries 6–7). No product was observed at all when the reaction was carried out in toluene (entry 5). When MeCN was used, high yield with a little lower enantioselectivitiy were obtained (entry 8). We envisaged that the low yields might be attributed to low solubility of pyridine quaternary salt intermediates in ether or hydrocarbon solvents. So, some large-polar solvents were added to the above reaction as co-solvents. We were pleased to find that these co-solvents promoted the reaction efficiently (entries 9–11) and a mixed solvent of dioxane and DMSO (10/1, *v*/*v*) was found to be the best one (entry 10). Several bases were examined and 1,1,3,3-tetramethylguanidine (TMG) was undoubtedly the best choice (entries 12–16).

Table S1 The effect of solvent and base on the reaction[a]

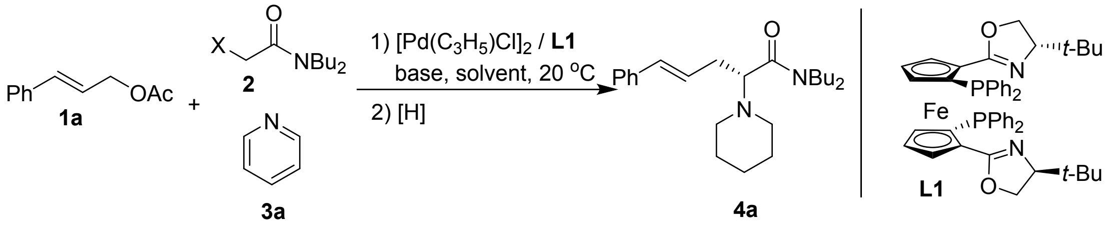

Entry	X	Solvent	Base	Yield (%)[b]	Ee (%)[c]
1	Cl	EtOH	TMG	NP	-
2	Br	EtOH	TMG	92	<10
3	Br	*n*-PrOH	TMG	95	<10
4	Br	*i*-PrOH	TMG	92	<10
5	Br	toluene	TMG	NP	-
6	Br	THF	TMG	trace	20
7	Br	dioxane	TMG	trace	43
8	Br	MeCN	TMG	91	41
9	Br	dioxane[d]	TMG	71	60
10	Br	dioxane[e]	TMG	92	77
11	Br	dioxane[f]	TMG	58	55
12	Br	dioxane[e]	Et_3N	NP	-

13	Br	dioxane[e]	Na_2CO_3	NP	-
14	Br	dioxane[e]	Cs_2CO_3	72	<10
15	Br	dioxane[e]	*t*-BuONa	NP	-
16	Br	dioxane[e]	DBU	88	24

[a] Reaction conditions: **1a** (0.1 mmol) with **2a** (0.2 mmol) and **3a** (0.5 mmol) under nitrogen atmosphere with a catalytic system of $[Pd(\eta^3\text{-}C_3H_5)Cl]_2$ (2.5 mol%) and **L*** (6 mol%) in the presence of a base (0.16 mmol) in an indicated solvent (2 mL) at 20 °C for 12 h; The pyridine quaternary salt was reduced with Raney-Ni under H_2 atomosphere. [b] Isolated yield. [c] Determined by HPLC using a chiral Daicel column. [d] MeCN was added as a co-solvent. [e] DMSO was added as a co-solvent. [f] DMF was added as a co-solvent

Then, **2** with different *N*-substituents were taken into consideration (Table S2). Linear alkyl groups, such as Et, Pr, pentyl could give higher yields but with somewhat lower enantioselectivities (**5aa**, **5ab** and **5ad**) than that of Bu group (**4a**, 96% yield and 95% ee). When *i*-Pr group adopted, only trace amount of product was obtained (**5ac**). Linear unsaturated groups with different steric hindrance were subjected to the reaction, no better results were obtained (**5ae** and **5b**). Replacing one Bu group on the nitrogen atom of **2a** with butenyl group resulted in a dramatically decrease of enantioselectivity (**5af**). When DMB (2,4-dimethoxybenzyl) was used instead of Bu group on the nitrogen atom of **2a**, the desired product **5ag** was obtained in 92% yield and 90% ee**.**

Table S2 Screen of **2** with different *N*-substituents[a]

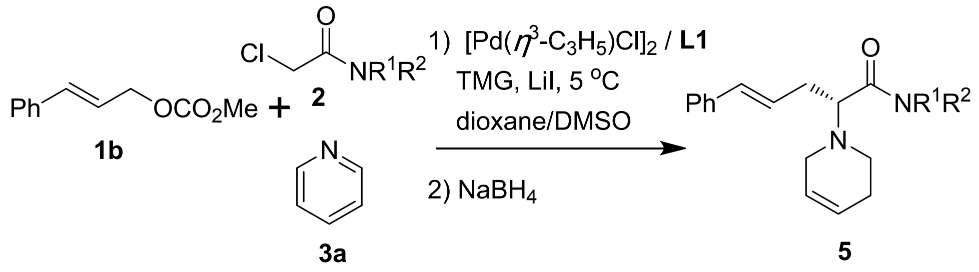

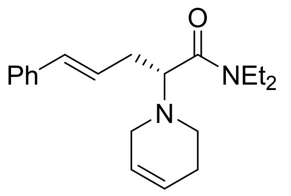

5aa
91%, 55% ee

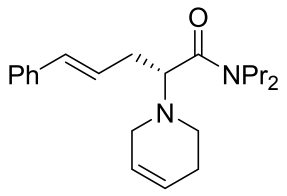

5ab
93%, 83%ee

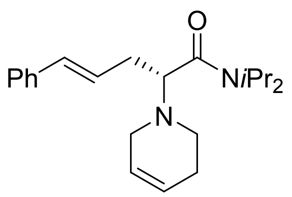

5ac
trace

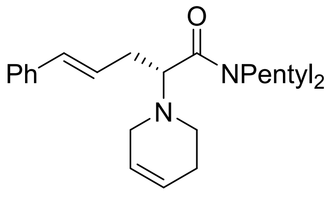

5ad
90%, 81%ee

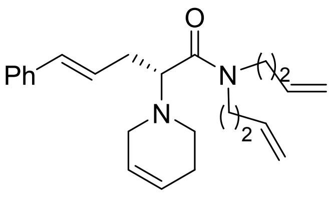

5ae
92%, 69% ee

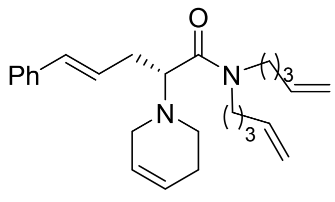

5f
92%, 90% ee

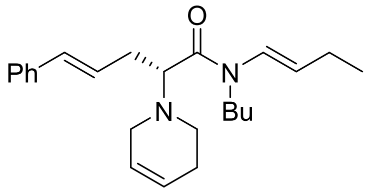

5af
92%, 40% ee

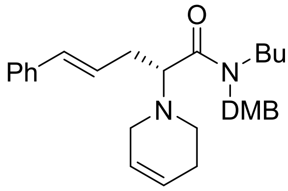

5ag[b]
92%, 90%ee

[a] Reaction conditions: **1a** (0.1 mmol) with **2a** (0.2 mmol) and **3a** (0.5 mmol) in a mixed solvent of dioxane/DMSO (10/1, *v*/*v*, 2mL) under nitrogen atmosphere with a catalytic system of $[Pd(\eta^3-C_3H_5)Cl]_2$ (2.5 mol%) and **L*** (6.0 mol%) with 1,1,3,3-Tetramethylguanidine (TMG) as a base (0.16 mmol) in the presence of LiI (0.1 mmol) at 5 °C for 12 h; The pyridine quaternary salt was reduced by $NaBH_4$; Isolated yields; ees were determined by HPLC using a chiral Daicel column. [b] Data and spectra were collected after removing the DMB group with TFA, see compound **10**.

4. General Procedure: Pd-Catalyzed Three-Component Allylic Substitution

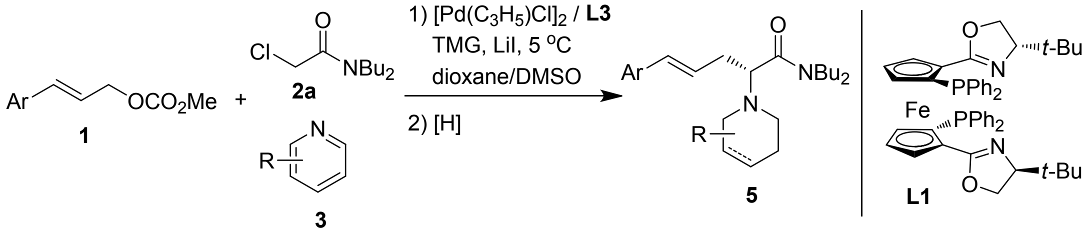

To an oven-dried glassware were added $[Pd(C_3H_5)Cl]_2$ (0.9 mg, 2.5 mol%), **L1** (4.8 mg, 5.5 mol%) and anhydrous dioxane (1.0 mL). To another dry glassware were added **2** (0.2 mmol), **3** (0.5 mmol), ultra-dry LiI (0.1 mmol, 13.3 mg) and anhydrous dioxane (1.0 mL). Then the first glassware was stirred at RT and second glassware was stirred at 100 °C under a nitrogen atmosphere for 1 h. Allylic carbonate **1** (0.1 mmol) was added to the first glassware. DMSO (0.2 mL) was added to the second glassware followed by 1,1,3,3-Tetramethylguanidine (TMG, 0.16 mmol). The solution in the first glassware was added dropwise to another one. The reaction was stirred at 5 °C for 12 h. The product was reduced *in situ* by one of the following methods. (1) Reduced by H_2: Raney-Ni (water slurry, 50 μm, 0.2 mL) was added to the reaction mixture and the mixture was stirred at H_2 atmosphere (10 bar) for 1 h. The reaction mixture was filtrated, diluted with EtOAc, washed with brine, dried over anhydrous Na_2SO_4 and then concentrated in vacuo. The residue was then purified by flash column chromatography (PE/EtOAc = 10/1) to give the corresponding products. (2) Reduced with $NaBH_4$: The solvent was removed in vacuo. MeOH (2 mL) was added and the resulting mixture was cooled to –20 °C quickly. Then, $NaBH_4$ (0.5 mmol) was added in one portion and the reaction was stirred for 2 h. The reaction mixture was diluted with EtOAc, washed with brine, dried over anhydrous Na_2SO_4 and then concentrated in vacuo. The residue was purified by flash column chromatography (PE/EtOAc = 10/1) to give the corresponding products. (3) Reduced with $NaBH_4$ for alkyl substituted substrates（**5a**, **5b**, **5c**, **5d** and **5e**）: The solvent was removed in vacuo. MeOH (2 mL) was added and the resulting mixture was cooled to –40 °C quickly. Then, $NaBH_4$ (0.5 mmol) was added in one portion and the reaction was stirred for 2 h. The reaction mixture was diluted with EtOAc, washed with brine, dried over anhydrous Na_2SO_4 and then concentrated in vacuo. The residue was purified by flash column

chromatography (PE/EtOAc = 10/1) to give the corresponding products.

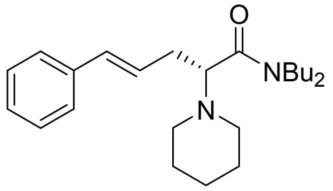

(*E*)-*N*,*N*-Dibutyl-5-phenyl-2-(piperidin-1-yl)pent-4-enamide (4a): Yellow oil (35.6 mg, 96%). $[\alpha]_D^{25}$ = +59.9 (*c* 0.18, acetone); ^{1}H NMR (400 MHz, $CDCl_3$): δ 7.33–7.21 (m, 4H), 7.20–7.10 (m, 1H), 6.41 (d, J = 16 Hz, 1H), 6.14 (dt, J = 15.6, 7.6 Hz 1H), 3.51–3.33 (m, 3H), 3.22–3.00 (m, 2H), 2.83–2.72 (m, 1H), 2.71–2.58 (m, 2H), 2.53–2.32 (m, 3H), 1.64–1.30 (m, 10H), 1.33–1.19 (m, 4H), 0.86 (q, J = 7.2 Hz 6H); ^{13}C NMR (100 MHz, $CDCl_3$): δ 170.5, 137.7, 131.6, 128.4, 128.2, 126.8, 125.9, 65.8, 50.3, 47.4, 45.6, 31.5, 29.8, 28.9, 26.6, 24.5, 20.3, 20.2, 13.9, 13.8; IR (KBr) cm^{-1}: 2976, 2905, 1671, 1456, 1382, 879; HRMS (APCI) $[M+H]^+$ calcd 371.3057, found 371.3088; Daicel Chiralpak IC-3 column, *n*-hexane/*i*-PrOH = 90/10, 0.8 mL/min, 254 nm: t_{major} = 24.379 min, t_{minor} = 18.314 min.

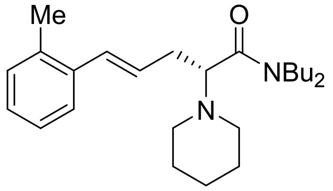

(*E*)-*N*,*N*-Dibutyl-2-(piperidin-1-yl)-5-(o-tolyl)pent-4-enamide (4b): Yellow oil (36.2 mg, 94%). $[\alpha]_D^{25}$ = +45.9 (*c* 0.22, acetone); ^{1}H NMR (400 MHz, $CDCl_3$): δ 7.35 (d, J = 5.6 Hz, 1H), 7.13–7.06 (m, 3H), 6.66–6.54 (d, J = 15.6 Hz, 1H), 6.03 (dt, J = 15.6, 7.6 Hz 1H), 3.54–3.34 (m, 3H), 3.19–3.05 (m, 2H), 2.88–2.73 (m, 1H), 2.72–2.59 (m, 2H), 2.57–2.40 (m, 3H), 2.29 (s, 3H), 1.52–1.18 (m, 14H), 0.87 (t, J = 7.2 Hz, 6H); ^{13}C NMR (100 MHz, $CDCl_3$): δ 170.5, 136.8, 134.9, 130.1, 129.6, 126.8, 125.9, 125.4, 109.9, 65.8, 50.4, 47.4, 45.6, 31.6, 29.8, 29.3, 26.6, 24.5, 20.3, 20.2, 19.8, 13.9, 13.8; IR (KBr) cm^{-1}: 2971, 2849, 1660, 1507, 938; HRMS (APCI) $[M+H]^+$ calcd 385.3213, found 385.3230; Daicel Chiralpak IC-3 column, *n*-hexane/*i*-PrOH = 95/5, 0.8 mL/min, 254 nm: t_{major} = 25.141 min, t_{minor} = 14.969 min.

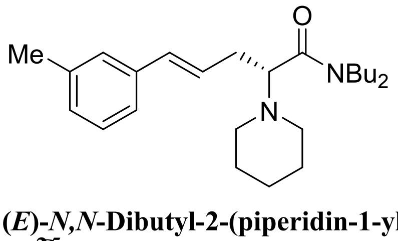

(*E*)-*N*,*N*-Dibutyl-2-(piperidin-1-yl)-5-(m-tolyl)pent-4-enamide (4c): Yellow oil (35.3 mg, 92%); $[\alpha]_D^{25}$ = +35.5 (*c* 0.28, acetone); ^{1}H NMR (400 MHz, $CDCl_3$): δ 7.19–7.05 (m, 3H), 6.98 (d, J = 6.8 Hz, 1H), 6.37 (d, J = 16.4 Hz, 1H), 6.18–6.07 (m, 1H), 3.52–3.34 (m, 3H), 3.19–3.05 (m, 2H), 2.80–2.71 (m, 1H), 2.70–2.56 (m, 2H), 2.56–2.35 (m, 3H), 2.30 (s, 3H), 1.56–1.19 (m, 14H), 0.88 (q, J = 6.8 Hz, 6H); ^{13}C NMR (100 MHz, $CDCl_3$): δ 170.5, 137.8, 137.6, 131.7, 128.2, 128.1, 127.5, 126.6, 123.1, 65.9, 50.3, 47.4, 45.6, 31.5, 29.7, 28.9, 26.5, 24.5, 21.3, 20.2, 20.1, 13.9, 13.8; IR (KBr) cm^{-1}: 2978, 2906, 1716, 1418, 668; HRMS (APCI) $[M+H]^+$ calcd 385.3213, found

385.3221 Daicel Chiralpak IC-3 column, *n*-hexane/*i*-PrOH = 95/5, 0.8 mL/min, 254 nm: t_{major} = 19.604 min, t_{minor} = 16.423 min.

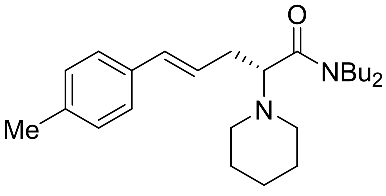

(*E*)-*N,N*-Dibutyl-2-(piperidin-1-yl)-5-(p-tolyl)pent-4-enamide (4d): Yellow oil (33.2 mg, 86%). $[\alpha]^{25}$ = +48.4 (*c* 0.33, acetone); ^{1}H NMR (400 MHz, $CDCl_3$): δ 7.18 (d, *J* = 8 Hz, 2H), 7.03 (d, *J* = 7.6 Hz, 2H), 6.41–6.31 (d, *J* = 16 Hz, 1H), 6.16–6.01 (m, 1H), 3.52–3.34 (m, 3H), 3.22–3.04 (m, 2H), 2.84–2.71 (m, 1H), 2.70–2.58 (m, 2H), 2.59–2.35 (m, 3H), 2.29 (s, 3H), 1.55–1.11 (m, 14H), 0.85 (q, *J* = 6.8 Hz, 6H); ^{13}C NMR (100 MHz, $CDCl_3$): δ170.5, 136.4, 134.9, 131.4, 129.0, 127.2, 125.8, 65.8, 50.3, 47.3, 45.6, 31.5, 29.7, 28.9, 26.5, 24.5, 21.1, 20.2, 20.1, 13.9, 13.8; IR (KBr) cm^{-1}: 2925, 1733, 1261, 1045, 879; HRMS (APCI) $[M+H]^+$ calcd 385.3213, found 385.3223; Daicel Chiralpak IC-3 column, *n*-hexane/*i*-PrOH = 95/5, 0.8 mL/min, 254 nm: t_{major} = 27.475 min, t_{minor} = 21.027 min.

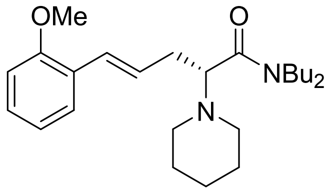

(*E*)-*N,N*-Dibutyl-5-(2-methoxyphenyl)-2-(piperidin-1-yl)pent-4-enamide (4e): Yellow oil (34.1 mg, 85%). $[\alpha]^{25}$ = +68.2 (*c* 0.18, acetone); ^{1}H NMR (400 MHz, $CDCl_3$): δ 7.35 (d, *J* = 7.6 Hz, 1H), 7.13 (t, *J* = 8 Hz, 1H), 6.90–6.76 (m, 2H), 6.70 (d, *J* = 16 Hz, 1H), 6.20–6.07 (m, 1H), 3.81 (s, 3H), 3.53–3.32 (m, 3H), 3.21–3.05 (m, 2H), 2.82–2.71 (m, 1H), 2.72–2.58 (m, 2H), 2.57–2.37 (m, 3H), 1.57–1.17 (m, 14H), 0.96–0.79 (m, 6H); ^{13}C NMR (100 MHz, $CDCl_3$): δ 170.7, 156.1, 128.9, 127.8, 126.7, 126.3, 125.9, 120.5, 110.6, 65.9, 55.3, 50.3, 47.4, 45.6, 31.5, 29.7, 29.3, 26.5, 24.5, 20.2, 20.1, 13.9, 13.8; IR (KBr) cm^{-1}: 2978, 2360, 1716, 1507, 1086, 1046, 879; HRMS (APCI) $[M+H]^+$ calcd 401.3163, found 401.3173; Daicel Chiralpak IC-3 column, *n*-hexane/*i*-PrOH = 85/15, 0.8 mL/min, 254 nm: t_{major} = 30.257 min, t_{minor} = 14.089 min.

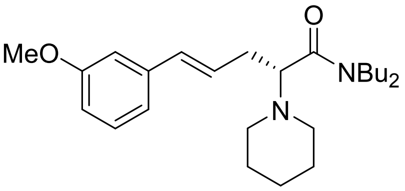

(*E*)-*N,N*-Dibutyl-5-(3-methoxyphenyl)-2-(piperidin-1-yl)pent-4-enamide (4f): Yellow oil (38.2 mg, 95%). $[\alpha]^{25}$ = +44.9 (*c* 0.25, acetone); ^{1}H NMR (400 MHz, $CDCl_3$): δ 7.16 (t, *J* = 8 Hz, 1H), 6.88 (d, *J* = 7.6 Hz, 1H), 6.84–6.80 (m, 1H), 6.71 (dd, *J* = 8.4, 2.4 Hz, 1H), 6.37 (d, *J* = 16 Hz, 1H), 6.19–6.07 (m, 1H), 3.77 (s, 3H), 3.51–3.35 (m, 3H), 3.20–3.04 (m, 2H), 2.83–2.70 (m, 1H), 2.71–2.56 (m, 2H), 2.53–2.36 (m, 3H), 1.60–1.17 (m, 14H), 0.95–0.82 (m, 6H); ^{13}C NMR (100 MHz, $CDCl_3$): δ 170.4, 159.7, 139.1, 131.5, 129.3, 128.7, 118.6, 112.5, 111.2, 65.8, 55.1,

50.3, 47.3, 45.6, 31.5, 29.7, 28.8, 26.5, 24.5, 20.2, 20.1, 13.9, 13.8; IR (KBr) cm^{-1}: 2978, 1716, 1558, 1261, 1085, 879; HRMS (APCI) $[M+H]^+$ calcd 401.3163, found 401.3168; Daicel Chiralpak IC-3 column, *n*-hexane/*i*-PrOH = 90/10, 0.8 mL/min, 254 nm: t_{major} = 23.437 min, t_{minor} = 19.105 min.

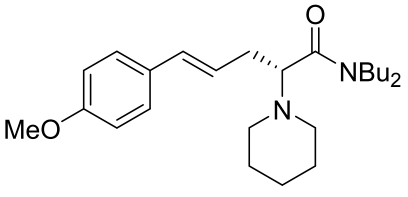

(*E*)-*N*,*N*-Dibutyl-5-(4-methoxyphenyl)-2-(piperidin-1-yl)pent-4-enamide (4g): Yellow oil (33.5 mg, 84%). $[\alpha]^{25}$ = +46.6 (*c* 0.32, acetone); ^{1}H NMR (400 MHz, $CDCl_3$): δ 7.22 (d, *J* = 8.8 Hz, 2H), 6.79 (d, *J* = 8.4 Hz, 2H), 6.34 (d, *J* = 16.0 Hz, 1H), 6.04–5.92 (m, 1H), 3.77 (s, 3H), 3.56–3.33 (m, 3H), 3.19–3.04 (m, 2H), 2.67 (dd, *J* = 60.1, 42.9 Hz, 6H), 1.60–1.16 (m, 14H), 0.96–0.78 (m, 6H); ^{13}C NMR (100 MHz, $CDCl_3$): δ 170.6, 158.6, 131.0, 130.5, 129.2, 127.0, 126.0, 113.8, 113.6, 65.8, 55.2, 50.3, 47.4, 45.6, 31.5, 29.7, 29.0, 26.5, 24.5, 20.2, 20.1, 13.9, 13.8; IR (KBr) cm^{-1}: 2979, 1716, 1558, 879; HRMS (APCI) $[M+H]^+$ calcd 401.3163, found 401.3173; Daicel Chiralpak IC-3 column, *n*-hexane/*i*-PrOH = 90/10, 0.8 mL/min, 254 nm: t_{major} = 21.605 min, t_{minor} = 21.077 min.

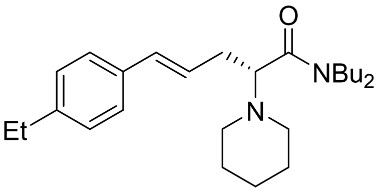

(*E*)-*N*,*N*-Dibutyl-5-(4-ethylphenyl)-2-(piperidin-1-yl)pent-4-enamide (4h): Yellow oil (35.9 mg, 90%). $[\alpha]^{25}$ = +69.2 (*c* 0.20, acetone); ^{1}H NMR (400 MHz, $CDCl_3$): δ 7.21 (d, *J* = 8.0 Hz, 2H), 7.08 (d, *J* = 8.0 Hz, 2H), 6.37 (d, *J* = 16.0 Hz, 1H), 6.14–6.03 (m, 1H), 3.52–3.34 (m, 3H), 3.20–3.04 (m, 2H), 2.79–2.71 (m, 1H), 2.70–2.52 (m, 4H), 2.53–2.34 (m, 3H), 1.32–1.14 (m, 17H), 0.89–0.82 (m, 6H); ^{13}C NMR (100 MHz, $CDCl_3$): δ 170.6, 142.9, 135.1, 131.5, 127.8, 127.3, 125.9, 65.8, 50.3, 47.3, 45.6, 31.5, 29.7, 28.9, 28.5, 26.5, 24.5, 20.3, 20.2, 15.5, 13.9, 13.8; IR (KBr) cm^{-1}: 2934, 1716, 1558, 1456, 880; HRMS (APCI) $[M+H]^+$ calcd 399.3370, found 399.3380; Daicel Chiralpak IC-3 column, *n*-hexane/*i*-PrOH = 95/5, 0.8 mL/min, 254 nm: t_{major} = 21.694 min, t_{minor} = 19.572 min.

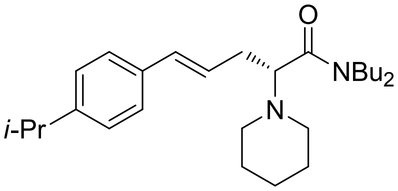

(*E*)-*N*,*N*-Dibutyl-5-(4-isopropylphenyl)-2-(piperidin-1-yl)pent-4-enamide (4i): Yellow oil (38.6 mg, 94%). $[\alpha]^{25}$ = +71.9 (*c* 0.22, acetone); ^{1}H NMR (400 MHz, $CDCl_3$): δ 7.23 (d, *J* = 7.6 Hz, 2H), 7.11 (d, *J* = 7.6 Hz, 2H), 6.37 (d, *J* = 16.0 Hz, 1H), 6.16–6.02 (m, 1H), 3.49–3.35 (m, 3H), 3.21–3.03 (m, 2H), 2.96–2.57 (m, 4H), 2.59–2.32 (m, 3H), 1.66–1.10 (m, 20H), 0.98–0.79

(m, 6H); ^{13}C NMR (100 MHz, $CDCl_3$): δ 170.6, 147.6, 135.3, 131.5, 127.4, 126.4, 125.9, 65.9, 50.4, 47.4, 45.6, 33.7, 31.5, 29.8, 28.9, 26.6, 24.5, 23.9, 20.3, 20.2, 13.8, 13.8.; IR (KBr) cm^{-1}: 2979, 1684, 1507, 1146, 879; HRMS (APCI) $[M+H]^+$ calcd 413.3526, found 413.3538; Daicel Chiralpak IC-3 column, *n*-hexane/*i*-PrOH = 95/5, 0.8 mL/min, 254 nm: t_{major} = 16.575 min, t_{minor} = 18.078 min.

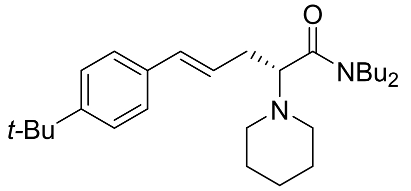

(*E*)-*N,N*-Dibutyl-5-(4-(tert-butyl)phenyl)-2-(piperidin-1-yl)pent-4-enamide (4j): Yellow oil (39.7 mg, 93%). $[\alpha]_D^{20}$ = +37.2 (*c* 0.20, acetone); ^{1}H NMR (400 MHz, $CDCl_3$): δ 7.32–7.18 (m, 4H), 6.38 (d, J = 15.6 Hz, 1H), 6.16–6.05 (m, 1H), 3.53–3.34 (m, 3H), 3.18–3.05 (m, 2H), 2.80–2.71 (m, 1H), 2.71–2.59 (m, 2H), 2.54–2.36 (m, 3H), 1.51–1.14 (m, 23H), 0.87 (q, J = 7.2 Hz, 6H); ^{13}C NMR (100 MHz, $CDCl_3$): δ 170.5, 149.8, 134.9, 131.3, 127.4, 125.6, 125.2, 65.9, 50.3, 47.3, 45.6, 34.4, 31.5, 31.2, 29.7, 28.9, 26.5, 24.50, 20.2, 20.1, 13.9, 13.8; IR (KBr) cm^{-1}: 2977, 1653, 1418, 1046, 879; HRMS (APCI) $[M+H]^+$ calcd 427.3683, found 427.3685; Daicel Chiralpak IC-3 column, *n*-hexane/*i*-PrOH = 95/5, 0.8 mL/min, 254 nm: t_{major} = 13.086 min, t_{minor} = 17.201 min.

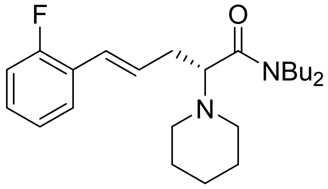

(*E*)-*N,N*-Dibutyl-5-(2-fluorophenyl)-2-(piperidin-1-yl)pent-4-enamide (4k): Yellow oil (36.1 mg, 93%). $[\alpha]_D^{20}$ = +50.6 (*c* 0.25, acetone); ^{1}H NMR (400 MHz, $CDCl_3$): δ 7.37 (t, J = 8.0 Hz, 1H), 7.17–7.07 (m, 1H), 7.05–6.89 (m, 2H), 6.55 (d, J = 16.0 Hz, 1H), 6.30–6.16 (m, 1H), 3.56–3.29 (m, 3H), 3.20–3.05 (m, 2H), 2.85–2.74 (m, 1H), 2.74–2.57 (m, 2H), 2.57–2.32 (m, 3H), 1.63–1.16 (m, 14H), 0.87 (t, J = 7.2 Hz, 6H); ^{13}C NMR (100 MHz, $CDCl_3$): δ 170.5, 159.9 ($J_{C\text{-}F}$ = 250 Hz), 131.2 ($J_{C\text{-}F}$ = 2 Hz), 128.0 ($J_{C\text{-}F}$ = 10 Hz), 127.0 ($J_{C\text{-}F}$ = 2 Hz), 125.4 ($J_{C\text{-}F}$ = 10 Hz), 123.9 ($J_{C\text{-}F}$ = 4 Hz), 123.8 ($J_{C\text{-}F}$ = 4 Hz), 115.5 ($J_{C\text{-}F}$ = 22 Hz), 65.9, 50.3, 47.4, 45.6, 31.5, 29.7, 29.1, 26.5, 24.4, 20.3, 20.2, 13.9, 13.8; ^{19}F NMR (376 MHz, $CDCl_3$): δ −118.9; IR (KBr) cm^{-1}: 2979, 1773, 1559, 1419, 1045, 879; HRMS (APCI) $[M+H]^+$ calcd 389.2963, found 389.2966; Daicel Chiralpak IC-3 column, *n*-hexane/*i*-PrOH = 95/5, 0.8 mL/min, 254 nm: t_{major} = 13.455 min, t_{minor} = 11.373 min.

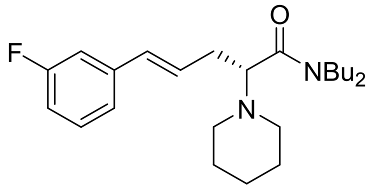

(*E*)-*N,N*-Dibutyl-5-(3-fluorophenyl)-2-(piperidin-1-yl)pent-4-enamide (4l): Yellow oil (33.5 mg, 86%). $[\alpha]_D^{20}$ = +69.2 (*c* 0.26, acetone); ^{1}H NMR (400 MHz, $CDCl_3$): δ 7.19 (q, J = 7.2 Hz,

1H), 7.03 (d, J = 7.6 Hz, 1H), 6.98 (d, J = 10.4 Hz, 1H), 6.84 (t, J = 9.2 Hz, 1H), 6.36 (d, J = 16.0 Hz, 1H), 6.21–6.09 (m, 1H), 3.56–3.33 (m, 3H), 3.21–3.04 (m, 2H), 2.82–2.72 (m, 1H), 2.69–2.54 (m, 2H), 2.55–2.30 (m, 3H), 1.58–1.19 (m, 14H), 0.96–0.78 (m, 6H), 1.63–1.16 (m, 14H), 0.87 (t, J = 7.2 Hz, 6H); ^{13}C NMR (100 MHz, $CDCl_3$): δ 170.4, 163.0 ($J_{C\text{-}F}$ = 240 Hz), 140.1 ($J_{C\text{-}F}$ = 10 Hz), 130.6 ($J_{C\text{-}F}$ = 2 Hz), 129.9, 129.7 ($J_{C\text{-}F}$ = 10 Hz), 121.8 ($J_{C\text{-}F}$ = 2 Hz), 113.5 ($J_{C\text{-}F}$ = 8 Hz), 112.3 ($J_{C\text{-}F}$ = 8 Hz), 65.7, 50.3, 47.3, 45.5, 31.5, 29.7, 28.6, 26.5, 24.4, 20.2, 20.1, 13.9, 13.8; ^{19}F NMR (376 MHz, $CDCl_3$): δ −113.9; IR (KBr) cm^{-1}: 2978, 1653, 1540, 1374, 879, 668; HRMS (APCI) $[M+H]^+$ calcd 389.2963, found 389.2967; Daicel Chiralpak IC-3 column, *n*-hexane/*i*-PrOH = 95/5, 0.8 mL/min, 254 nm: t_{major} = 15.438 min, t_{minor} = 12.776 min.

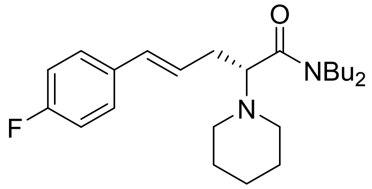

(*E*)-*N,N*-Dibutyl-5-(4-fluorophenyl)-2-(piperidin-1-yl)pent-4-enamide (4m): Yellow oil (36.1 mg, 93%). $[\alpha]_D^{20}$ = +66.2 (c 0.16, acetone); ^{1}H NMR (400 MHz, $CDCl_3$): δ 7.24 (dd, J = 8.4, 5.2 Hz, 2H), 6.93 (t, J = 8.4 Hz, 2H), 6.36 (d, J = 16.0 Hz, 1H), 6.10–5.97 (m, 1H), 3.54–3.30 (m, 3H), 3.20–3.04 (m, 2H), 2.83–2.72 (m, 1H), 2.72–2.57 (m, 2H), 2.55–2.31 (m, 3H), 1.70–1.13 (m, 14H), 0.98–0.77 (m, 6H); ^{13}C NMR (100 MHz, $CDCl_3$): δ 170.4, 161.8 ($J_{C\text{-}F}$ = 240 Hz), 133.8 ($J_{C\text{-}F}$ = 4 Hz), 130.4, 128.0 ($J_{C\text{-}F}$ = 2 Hz), 127.3 ($J_{C\text{-}F}$ = 8 Hz), 115.2 ($J_{C\text{-}F}$ = 22 Hz), 65.7, 50.2, 47.3, 45.5, 31.5, 29.7, 28.7, 26.5, 24.4, 20.2, 20.1, 13.9, 13.8; ^{19}F NMR (376 MHz, $CDCl_3$): δ −115.7; IR (KBr) cm^{-1}: 2979, 2929, 1684, 1362, 879, 517; HRMS (APCI) $[M+H]^+$ calcd 389.2963, found 389.2960; Daicel Chiralpak IC-3 column, *n*-hexane/*i*-PrOH = 95/5, 0.8 mL/min, 254 nm: t_{major} = 15.077 min, t_{minor} = 13.774 min.

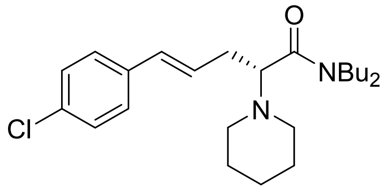

(*E*)-*N,N*-Dibutyl-5-(4-chlorophenyl)-2-(piperidin-1-yl)pent-4-enamide (4n): Yellow oil (36.5 mg, 90%). $[\alpha]_D^{20}$ = +48.9 (c 0.4, acetone); ^{1}H NMR (400 MHz, $CDCl_3$): δ 7.23–7.18 (m, 4H), 6.36 (d, J = 14.8 Hz, 1H), 6.20–6.01 (m, 1H), 3.60–3.25 (m, 3H), 3.21–3.07 (m, 2H), 2.92–2.25 (m, 6H), 1.53–1.19 (m, 14H), 0.91–0.84 (m, 6H); ^{13}C NMR (100 MHz, $CDCl_3$): δ 170.4, 136.2, 132.3, 130.4, 129.1, 128.5, 127.1, 65.7, 50.3, 47.3, 45.6, 31.5, 29.7, 26.5, 24.5, 22.7, 20.3, 20.2, 13.9, 13.8; IR (KBr) cm^{-1}: 2978, 2927, 1684, 1362, 879, 568; HRMS (APCI) $[M+H]^+$ calcd 405.2667, found 405.2668; Daicel Chiralpak IC-3 column, *n*-hexane/*i*-PrOH = 95/5, 0.4 mL/min, 254 nm: t_{major} = 38.173 min, t_{minor} = 22.855 min.

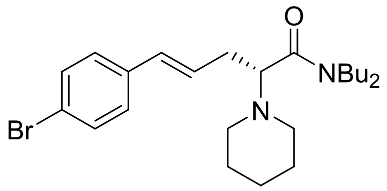

(*E*)-*N,N*-Dibutyl-5-(4-bromophenyl)-2-(piperidin-1-yl)pent-4-enamide (4o): Yellow oil (42.2 mg, 94%). $[\alpha]_D^{20}$ = +29.9 (*c* 0.36, acetone); ^{1}H NMR (400 MHz, $CDCl_3$): δ 7.36 (d, J = 8.4 Hz, 2H), 7.15 (d, J = 8.4 Hz, 2H), 6.34 (d, J = 16.0 Hz, 1H), 6.13 (dt, J = 15.2, 7.6 Hz, 1H), 3.55–3.33 (m, 3H), 3.20–3.04 (m, 2H), 2.82–2.73 (m, 1H), 2.71–2.57 (m, 2H), 2.56–2.36 (m, 3H), 1.57–1.20 (m, 14H), 0.93–0.81 (m, 6H); ^{13}C NMR (100 MHz, $CDCl_3$): δ 170.4, 136.6, 131.4, 130.5, 129.3, 127.4, 120.4, 65.7, 50.3, 47.3, 45.5, 31.5, 29.7, 28.7, 26.5, 24.4, 20.2, 20.1, 13.9, 13.8; IR (KBr) cm^{-1}: 2977, 2928, 1683, 1066, 879, 688; HRMS (APCI) $[M+H]^+$ calcd 449.2162, found 449.2164; Daicel Chiralpak IC-3 column, *n*-hexane/*i*-PrOH = 95/5, 0.8 mL/min, 254 nm: t_{major} = 25.302 min, t_{minor} = 17.077 min.

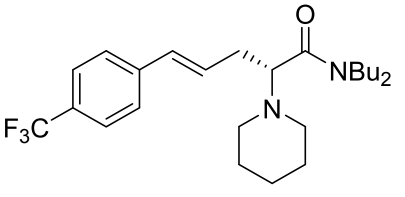

(*E*)-*N,N*-Dibutyl-2-(piperidin-1-yl)-5-(4-(trifluoromethyl)phenyl)pent-4-enamide (4p): Yellow oil (33.0 mg, 75%). $[\alpha]_D^{20}$ = +73.3 (*c* 0.24, acetone); ^{1}H NMR (400 MHz, $CDCl_3$): δ 7.51 (d, J = 8.4 Hz, 2H), 7.39 (d, J = 8.4 Hz, 2H), 6.45 (d, J = 16.0 Hz, 1H), 6.33–6.23 (m, 1H), 3.57–3.36 (m, 3H), 3.19–3.06 (m, 2H), 2.88–2.79 (m, 1H), 2.76–2.62 (m, 2H), 2.61–2.39 (m, 3H), 1.54–1.18 (m, 14H), 0.92–0.85 (m, 6H); ^{13}C NMR (100 MHz, $CDCl_3$): δ 170.3, 141.1, 131.4, 127.9 ($J_{C\text{-}F}$ = 190 Hz), 130.4, 126.0, 125.3 (q, $J_{C\text{-}F}$ = 4 Hz), 65.8, 50.3, 47.3, 45.5, 31.5, 29.7, 28.6, 26.5, 24.4, 20.2, 20.1, 13.9, 13.8; ^{19}F NMR (376 MHz, $CDCl_3$): δ –62.4; IR (KBr) cm^{-1}: 2978, 2923, 1670, 1653, 879; HRMS (APCI) $[M+H]^+$ calcd 439.2931, found 439.2928; Daicel Chiralpak AD-H column, *n*-hexane/*i*-PrOH = 99.5/0.5, 0.8 mL/min, 254 nm: t_{major} = 27.012 min, t_{minor} = 15.523 min.

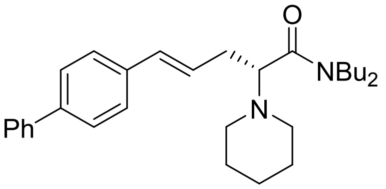

(*E*)-5-([1,1'-Biphenyl]-4-yl)-*N,N*-dibutyl-2-(piperidin-1-yl)pent-4-enamide (4q): Yellow oil (40.7 mg, 91%). $[\alpha]_D^{20}$ = +49.9 (*c* 0.25, acetone); ^{1}H NMR (400 MHz, $CDCl_3$): δ 7.57 (d, J = 8.4 Hz, 2H), 7.50 (d, J = 8.4 Hz, 2H), 7.45–7.20 (m, 5H), 6.45 (d, J = 16.0 Hz, 1H), 6.31–6.08 (m, 1H), 3.62–3.30 (m, 3H), 3.24–3.01 (m, 2H), 2.90–2.75 (m, 1H), 2.75–2.59 (m, 2H), 2.57–2.26 (m, 3H), 1.38–1.18 (m, 14H), 0.85–0.94 (m, 6H); ^{13}C NMR (100 MHz, $CDCl_3$): δ 170.5, 140.8, 139.5, 136.7, 131.2, 128.7, 128.5, 127.1, 127.0, 126.8, 126.3, 65.8, 50.3, 47.4, 45.6, 31.5, 29.6, 28.9, 26.5, 24.5, 20.3, 20.2, 13.9, 13.8; IR (KBr) cm^{-1}: 1748, 1540, 1472, 912; HRMS (APCI) $[M+H]^+$ calcd 447.3370, found 447.3380; Daicel Chiralpak IC-3 column, *n*-hexane/*i*-PrOH = 95/5, 0.8 mL/min, 254 nm: t_{major} = 27.566 min, t_{minor} = 24.641 min.

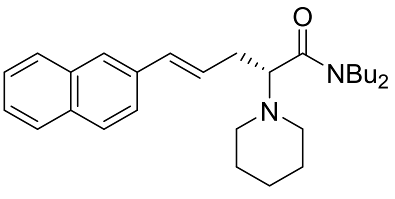

(*E*)-*N*,*N*-Dibutyl-5-(naphthalen-2-yl)-2-(piperidin-1-yl)pent-4-enamide (4r): Yellow oil (39.2 mg, 93%). $[\alpha]_D^{20}$ = +61.4 (*c* 0.11, acetone); ^{1}H NMR (400 MHz, $CDCl_3$): δ 7.88–7.59 (m, 4H), 7.52 (d, J = 8.8 Hz, 1H), 7.39 (p, J = 7.2 Hz, 2H), 6.57 (d, J = 15.6 Hz, 1H), 6.36–6.22 (m, 1H), 3.62–3.29 (m, 3H), 3.23–3.05 (m, 2H), 2.91–2.74 (m, 1H), 2.78–2.59 (m, 2H), 2.60–2.27 (m, 3H), 1.60–1.16 (m, 14H), 0.97–0.75 (m, 6H); ^{13}C NMR (100 MHz, $CDCl_3$): δ 170.6, 135.2, 133.6, 132.6, 131.7, 128.8, 127.9, 127.7, 127.5, 126.0, 125.4, 125.3, 123.5, 65.8, 50.3, 47.3, 45.6, 31.5, 29.7, 29.0, 26.5, 24.5, 20.2, 20.1, 13.9, 13.8; IR (KBr) cm^{-1}: 1558, 962, 688; $[M+H]^+$ calcd 421.3213, found 421.3216; Daicel Chiralpak AD-H column, *n*-hexane/*i*-PrOH = 99/1, 1 mL/min, 254 nm: t_{major} = 19.661 min, t_{minor} = 15.967 min.

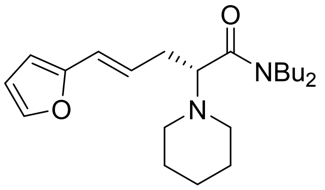

(*E*)-*N*,*N*-Dibutyl-5-(furan-2-yl)-2-(piperidin-1-yl)pent-4-enamide (4s): Yellow oil (32.9 mg, 91%). $[\alpha]_D^{20}$ = +40.7 (*c* 0.21, acetone); ^{1}H NMR (400 MHz, $CDCl_3$): δ 7.25 (s, 1H), 6.32–6.25 (m, 1H), 6.22 (d, J = 16.0 Hz, 1H), 6.11–5.97 (m, 2H), 3.52–3.32 (m, 3H), 3.20–3.03 (m, 2H), 2.79–2.68 (m, 1H), 2.68–2.52 (m, 2H), 2.54–2.31 (m, 3H), 1.56–1.14 (m, 14H), 0.94–0.80 (m, 6H); ^{13}C NMR (100 MHz, $CDCl_3$): δ 170.4, 153.1, 141.2, 127.2, 120.3, 110.9, 106.1, 65.5, 50.2, 47.4, 45.6, 31.5, 29.7, 28.7, 26.5, 24.4, 20.2, 20.1, 13.9, 13.8; IR (KBr) cm^{-1}: 2975, 2926, 1683, 1653, 1048, 880; HRMS (APCI) $[M+H]^+$ calcd 361.2850, found 361.2853; Daicel Chiralpak IC-3 column, *n*-hexane/*i*-PrOH = 95/5, 0.8 mL/min, 254 nm: t_{major} = 43.549 min, t_{minor} = 30.185 min.

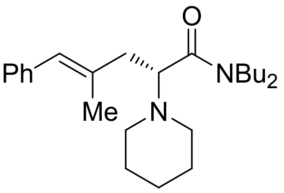

(*E*)-*N*,*N*-Dibutyl-4-methyl-5-phenyl-2-(piperidin-1-yl)pent-4-enamide (4t): Yellow oil (36.1 mg, 94%). $[\alpha]_D^{20}$ = +45.9 (*c* 0.60, acetone); ^{1}H NMR (400 MHz, $CDCl_3$): δ 7.33–7.24 (m, 2H), 7.22–7.09 (m, 3H), 6.30 (s, 1H), 3.65–3.55 (m, 1H), 3.54–3.36 (m, 2H), 3.26–3.05 (m, 2H), 2.86–2.64 (m, 3H), 2.61–2.37 (m, 3H), 1.88 (s, 3H), 1.58–1.20 (m, 14H), 0.95–0.78 (m, 6H); ^{13}C NMR (100 MHz, $CDCl_3$): δ 170.6, 138.5, 136.5, 128.7, 127.9, 127.1, 125.8, 63.9, 50.3, 47.5, 45.8, 36.1, 31.6, 29.8, 26.5, 24.5, 20.3, 20.2, 18.5, 13.9, 13.8; IR (KBr) cm^{-1}: 2973, 2925, 1677, 1250, 819; HRMS (APCI) $[M+H]^+$ calcd 385.3213, found 385.3213; Daicel Chiralpak IC-3 column, *n*-hexane/*i*-PrOH = 97/3, 0.8 mL/min, 254 nm: t_{major} = 16.562 min, t_{minor} = 13.513 min.

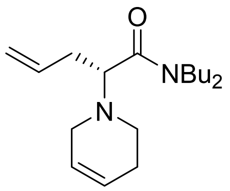

N,N-Dibutyl-2-(5,6-dihydropyridin-1(2H)-yl)pent-4-enamide (5a): Yellow oil (22.2 mg, 76%). $[\alpha]^{20}_{D}$ = +25.0 (c 0.26, acetone); ^{1}H NMR (400 MHz, $CDCl_3$): δ 5.81–5.61 (m, 3H), 5.11–4.94 (m, 2H), 3.57–3.29 (m, 3H), 3.24–3.01 (m, 4H), 2.84–2.55 (m, 3H), 2.39–2.24 (m, 1H), 2.18–1.94 (m, 2H), 1.57–1.16 (m, 8H), 0.89 (q, J = 6.8 Hz, 6H); ^{13}C NMR (100 MHz, $CDCl_3$): δ 170.4, 136.1, 125.6, 125.0, 116.5, 64.0, 48.7, 47.4, 45.7, 45.4, 31.5, 29.8, 29.7, 26.8, 20.2, 20.1, 13.9, 13.8; IR (KBr) cm^{-1}: 2978, 1698, 1558, 1046, 879; HRMS (APCI) $[M+H]^+$ calcd 293.2587, found 293.2605; Daicel Chiralpak IC-3 column, n-hexane/i-PrOH = 95/5, 0.8 mL/min, 210 nm: t_{major} = 22.716 min, t_{minor} = 23.954 min.

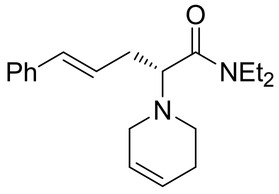

(E)-2-(5,6-Dihydropyridin-1(2H)-yl)-N,N-diethyl-5-phenylpent-4-enamide (5aa): Yellow oil (28.4 mg, 91%). ^{1}H NMR (400 MHz, $CDCl_3$): δ 7.35–7.13 (m, 5H), 6.44 (d, J = 16.0 Hz, 1H), 6.25–6.09 (m, 1H), 5.79–5.56 (m, 2H), 3.63–3.51 (m, 1H), 3.51–3.35 (m, 2H), 3.36–3.17 (m, 3H), 3.17–3.06 (m, 1H), 2.91–2.65 (m, 3H), 2.57–2.45 (m, 1H), 2.20–2.07 (m, 2H), 1.24–1.02 (m, 6H); ^{13}C NMR (100 MHz, $CDCl_3$): δ 170.2, 137.5, 132.0, 128.4, 127.6, 126.9, 126.0, 125.5, 125.1, 64.4, 48.7, 45.8, 41.9, 40.4, 29.7, 26.8, 14.6, 13.0; IR (KBr) cm^{-1}: 2918, 2848, 1636, 694; HRMS (APCI) $[M+H]^+$ calcd 313.2274, found 313.2273.

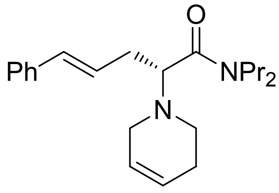

(E)-2-(5,6-Dihydropyridin-1(2H)-yl)-5-phenyl-N,N-dipropylpent-4-enamide (5ab): Yellow oil (31.6 mg, 93%). ^{1}H NMR (400 MHz, $CDCl_3$): δ 7.32–7.17 (m, 5H), 6.43 (d, J = 15.6 Hz, 1H), 6.26–6.08 (m, 1H), 5.78–5.56 (m, 2H), 3.64–3.53 (m, 1H), 3.46–3.34 (m, 2H), 3.31–3.22 (m, 1H), 3.21–3.06 (m, 3H), 2.91–2.66 (m, 3H), 2.56–2.44 (m, 1H), 2.16–2.07 (m, 2H), 1.61–1.45 (m, 4H), 0.89–0.81 (m, 6H); ^{13}C NMR (100 MHz, $CDCl_3$): δ 170.6, 137.6, 131.9, 128.4, 127.8, 126.9, 126.0, 125.6, 125.1, 64.4, 49.5, 48.8, 47.7, 45.6, 30.2, 29.4, 26.8, 22.6, 20.9, 11.4; IR (KBr) cm^{-1}: 3236, 2853, 1636, 1384; HRMS (APCI) $[M+H]^+$ calcd 341.2587, found 341.2589.

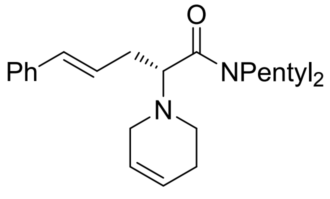

(E)-2-(5,6-Dihydropyridin-1(2H)-yl)-N,N-dipentyl-5-phenylpent-4-enamide (5ad): Yellow oil

(35.6 mg, 90%). ^{1}H NMR (400 MHz, $CDCl_3$): δ 7.38–7.11 (m, 5H), 6.43 (d, J = 16.4 Hz, 1H), 6.23–6.09 (m, 1H), 5.77–5.55 (m, 2H), 3.62–3.51 (m, 1H), 3.49–3.34 (m, 2H), 3.35–3.07 (m, 4H), 2.91–2.62 (m, 3H), 2.54–2.43 (m, 1H), 2.19–2.03 (m, 2H), 1.45–1.16 (m, 12H), 0.92–0.76 (m, 6H); ^{13}C NMR (100 MHz, $CDCl_3$): δ170.4, 137.6, 132.0, 128.4, 127.8, 126.9, 126.0, 125.6, 125.1, 64.4, 48.8, 47.8, 46.0, 45.6, 29.4, 29.2, 29.18, 29.15, 27.4, 26.8, 22.4, 22.4, 14.0, 13.9; IR (KBr) cm^{-1}: 2929, 1631, 1507, 1456, 1265, 965, 879, 784; HRMS (APCI) $[M+H]^+$ calcd 397.3213, found 397.3211.

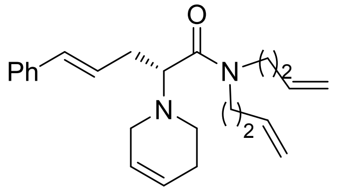

(*E*)-*N,N*-Di(but-3-en-1-yl)-2-(5,6-dihydropyridin-1(*2H*)-yl)-5-phenylpent-4-enamide (5ad): Yellow oil (33.4 mg, 92%). ^{1}H NMR (400 MHz, $CDCl_3$): δ 7.34–7.14 (m, 5H), 6.44 (d, J = 15.6 Hz, 1H), 6.24–6.09 (m, 1H), 5.85–5.59 (m, 4H), 5.16–4.84 (m, 4H), 3.65–3.43 (m, 3H), 3.36–3.17 (m, 3H), 3.16–3.05 (m, 1H), 2.92–2.76 (m, 2H), 2.75–2.62 (m, 1H), 2.53–2.41 (m, 1H), 2.38–2.23 (m, 4H), 2.17–2.06 (m, 2H); ^{13}C NMR (100 MHz, $CDCl_3$): δ 170.6, 137.6, 135.6, 134.7, 132.0, 128.4, 127.8, 126.9, 126.0, 125.5, 125.1, 117.1, 116.4, 64.8, 48.6, 47.4, 45.7, 45.6, 33.6, 32.2, 28.7, 26.8; IR (KBr) cm^{-1}: 2920, 2852, 1637, 1490, 1275, 748; HRMS (APCI) $[M+H]^+$ calcd 365.2587, found 365.2586.

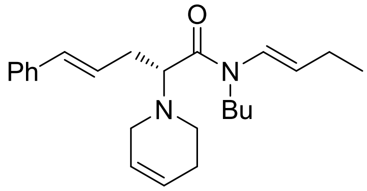

(*E*)-*N*-(But-1-en-1-yl)-*N*-butyl-2-(5,6-dihydropyridin-1(*2H*)-yl)-5-phenylpent-4-enamide (5af): Yellow oil (33.6 mg, 92%,). ^{1}H NMR (400 MHz, $CDCl_3$, mixture of two rotamers): δ 7.42–7.12 (m, 5.34H), 6.71 (d, J = 14.4 Hz, 0.66H), 6.43 (d, J = 15.6 Hz, 1H), 6.22–6.08 (m, 1H), 5.78–5.60 (m, 2H), 5.23–5.12 (m, 0.66H), 5.12–4.99 (m, 0.34H), 3.78–3.53 (m, 3H), 3.24 (d, J = 16.0 Hz, 1H), 3.12 (d, J = 16.0 Hz, 1H), 2.97–2.55 (m, 4H), 2.16–2.04 (m, 4H), 1.35–1.22 (m, 4H), 1.06–0.96 (m, 3H), 0.91–0.85 (m, 3H); ^{13}C NMR (100 MHz, $CDCl_3$): δ 169.7, 137.7, 132.3, 128.6, 127.5, 127.3, 127.2, 126.2, 125.7, 125.3, 117.0, 65.3, 48.8, 46.1, 44.1, 30.1, 29.4, 27.0, 23.8, 20.4, 14.4, 14.1; IR (KBr) cm^{-1}: 3290, 1644, 1265, 740; HRMS (APCI) $[M+H]^+$ calcd 367.2744, found 367.2740.

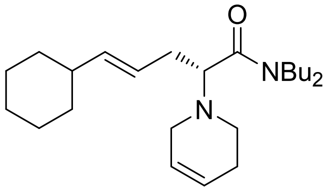

(*E*)-*N,N*-Dibutyl-5-cyclohexyl-2-(5,6-dihydropyridin-1(*2H*)-yl)pent-4-enamide (5b): Yellow oil (32.6 mg, 87%). $[\alpha]^{20}_{D}$ = +31.3 (c 0.60, acetone); ^{1}H NMR (400 MHz, $CDCl_3$): δ 5.74–5.57 (m, 2H), 5.46–5.36 (m, 1H), 5.34–5.21 (m, 1H), 3.52–3.30 (m, 3H), 3.29–2.98 (m, 4H), 2.81–2.53 (m, 3H), 2.30–2.18 (m, 1H), 2.14–2.01 (m, 2H), 1.93–1.79 (m, 1H), 1.68–1.03 (m, 18H), 0.96–

0.85 (m, 6H); ^{13}C NMR (100 MHz, $CDCl_3$): δ 170.8, 138.9, 125.7, 125.1, 124.4, 64.4, 49.0, 47.6, 45.9, 45.5, 40.7, 33.02, 33.0, 31.7, 29.9, 29.4, 26.8, 26.2, 26.0, 20.2, 13.9, 13.8; IR (KBr) cm^{-1}: 2919, 2849, 1634, 1558, 1506, 914; HRMS (APCI) $[M+H]^+$ calcd 375.3370, found 375.3379; Daicel Chiralpak IC-3 column, *n*-hexane/*i*-PrOH = 98/2, 0.8 mL/min, 210 nm: t_{major} = 29.191 min, t_{minor} = 33.756 min.

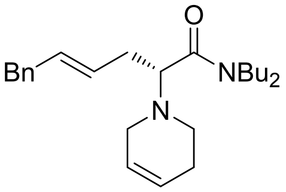

(*E*)-*N,N*-Dibutyl-2-(5,6-dihydropyridin-1(*2H*)-yl)-6-phenylhex-4-enamide (5c): Yellow oil (28.4 mg, 74%). $[\alpha]_D^{20}$ = +10.8 (*c* 0.80, acetone); ^{1}H NMR (400 MHz, $CDCl_3$): δ 7.25 (t, *J* = 8.0 Hz, 2H), 7.18 (d, *J* = 7.2 Hz, 1H), 7.16–7.11 (m, 2H), 5.77–5.58 (m, 3H), 5.51–5.39 (m, 1H), 3.52–3.34 (m, 3H), 3.30 (d, *J* = 6.8 Hz, 2H), 3.27–3.04 (m, 4H), 2.83–2.72 (m, 1H), 2.71–2.60 (m, 2H), 2.38–2.25 (m, 1H), 2.14–2.04 (m, 2H), 1.54–1.38 (m, 4H), 1.35–1.27 (m, 4H), 0.96–0.88 (m, 6H); ^{13}C NMR (100 MHz, $CDCl_3$): δ 170.8, 141.0, 131.6, 129.0, 128.7, 128.5, 126.1, 125.9, 125.3, 64.5, 49.1, 47.8, 46.1, 45.8, 39.3, 31.9, 30.1, 29.1, 27.1, 20.5, 14.2, 14.1; IR (KBr) cm^{-1}: 2956, 2920, 1646, 1558, 1265, 969; HRMS (APCI) $[M+H]^+$ calcd 383.3057, found 383.3059; Daicel Chiralpak IC-3 column, *n*-hexane/*i*-PrOH = 98/2, 0.8 mL/min, 230 nm: t_{major} = 31.521 min, t_{minor} = 28.512 min.

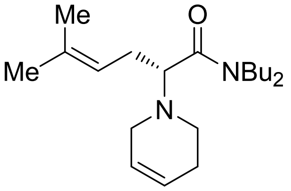

(*E*)-*N,N*-Dibutyl-2-(5,6-dihydropyridin-1(*2H*)-yl)-5-methylhex-4-enamide (5d): Yellow oil (16.8 mg, 52%). ^{1}H NMR (400 MHz, $CDCl_3$): δ 5.72–5.55 (m, 2H), 5.07–4.98 (m, 1H), 3.49–3.32 (m, 3H), 3.26–2.98 (m, 4H), 2.80–2.61 (m, 2H), 2.59–2.46 (m, 1H), 2.34–2.23 (m, 1H), 2.12–1.98 (m, 2H), 1.62 (s, 3H), 1.59 (s, 3H), 1.52–1.39 (m, 4H), 1.32–1.23 (m, 4H), 0.92–0.83 (m, 6H); ^{13}C NMR (100 MHz, $CDCl_3$): δ 170.9, 133.1, 125.7, 125.1, 121.3, 63.8, 48.9, 47.5, 45.8, 45.5, 31.5, 29.8, 26.8, 25.7, 24.8, 20.2, 20.1, 17.7, 13.9, 13.8; IR (KBr) cm^{-1}: 2919, 2849, 1634, 1507, 895, 739; HRMS (APCI) $[M+H]^+$ calcd 321.2900, found 321.2906.

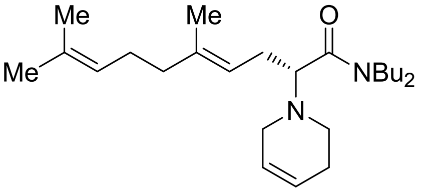

(*E*)-*N,N*-Dibutyl-2-(5,6-dihydropyridin-1(*2H*)-yl)-5,9-dimethyldeca-4,8-dienamide (5e): Yellow oil (21.2 mg, 54%). $[\alpha]_D^{20}$ = +10.7 (*c* 0.50, acetone); ^{1}H NMR (400 MHz, $CDCl_3$): δ 5.74–5.59 (m, 2H), 5.13–5.01 (m, 2H), 3.52–3.33 (m, 3H), 3.31–3.01 (m, 4H), 2.84–2.74 (m, 1H), 2.73–2.63 (m, 1H), 2.62–2.51 (m, 1H), 2.39–2.29 (m, 1H), 2.15–2.06 (m, 2H), 2.06–1.98 (m, 2H), 1.98–1.90 (m, 2H), 1.65 (s, 3H), 1.62 (s, 3H), 1.57 (s, 3H), 1.55–1.39 (m, 4H), 1.34–1.25 (m, 4H),

0.98–0.78 (m, 6H); ^{13}C NMR (100 MHz, $CDCl_3$): δ 170.9, 136.9, 131.3, 125.7, 125.1, 124.3, 121.1, 63.8, 48.9, 47.6, 45.9, 45.6, 39.8, 31.6, 29.9, 26.8, 26.7, 25.7, 24.7, 20. 3, 20.2, 17.6, 16.1, 13.9, 13.8; IR (KBr) cm^{-1}: 2958, 2927, 1646, 1558, 1265, 969; HRMS (APCI) $[M+H]^+$ calcd 389.3526, found 389.3531; Daicel Chiralpak IC-3 column, *n*-hexane/*i*-PrOH = 97/3, 0.8 mL/min, 210 nm: t_{major} = 13.680 min, t_{minor} = 11.816 min.

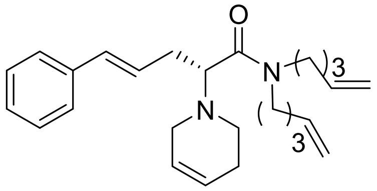

(*E*)-2-(5,6-Dihydropyridin-1(2*H*)-yl)-*N*,*N*-di(pent-4-en-1-yl)-5-phenylpent-4-enamide (5f):

Yellow oil (36.3 mg, 92%). $[\alpha]_D^{20}$ = +25.3 (*c* 0.15, acetone); ^1H NMR (400 MHz, $CDCl_3$): δ 7.35–7.08 (m, 5H), 6.42 (d, *J* = 15.6 Hz, 1H), 6.25–6.07 (m, 1H), 5.96–5.55 (m, 4H), 5.16–4.84 (m, 4H), 3.62–3.50 (m, 1H), 3.50–3.32 (m, 2H), 3.30–2.99 (m, 4H), 2.91–2.74 (m, 2H), 2.75–2.63 (m, 1H), 2.54–2.38 (m, 1H), 2.22–1.79 (m, 6H), 1.74–1.44 (m, 4H); ^{13}C NMR (100 MHz, $CDCl_3$): δ 170.5, 138.0, 137.5, 137.4, 132.0, 128.4, 127.7, 126.9, 125.9, 125.5, 125.1, 115.4, 114.8, 64.5, 48.7, 47.2, 45.6, 45.5, 31.1, 31.0, 28.9, 28.3, 26.9, 26.8; IR (KBr) cm^{-1}: 2977, 2927, 1717, 1507, 880, 668; HRMS (APCI) $[M+H]^+$ calcd 393.2900, found 393.2924; Daicel Chiralpak AD-H column, *n*-hexane/*i*-PrOH = 98/2, 1 mL/min, 254 nm: t_{major} = 15.029 min, t_{minor} = 11.897 min.

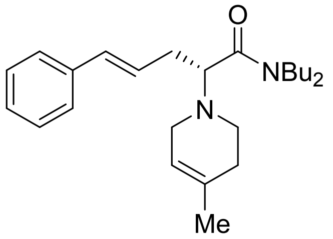

(*E*)-*N*,*N*-Dibutyl-2-(4-methyl-5,6-dihydropyridin-1(2*H*)-yl)-5-phenylpent-4-enamide (5g):

Yellow oil (34.5 mg, 90%). $[\alpha]_D^{20}$ = +29.3 (*c* 0.22, acetone); ^1H NMR (400 MHz, $CDCl_3$): δ 7.31–7.21 (m, 4H), 7.20–7.12 (m, 1H), 6.42 (d, *J* = 15.6 Hz, 1H), 6.20–6.08 (m, 1H), 5.37–5.28 (m, 1H), 3.69–3.48 (m, 1H), 3.52–3.34 (m, 2H), 3.33–2.93 (m, 4H), 2.91–2.61 (m, 3H), 2.57–2.40 (m, 1H), 2.13–1.89 (m, 2H), 1.65 (s, 3H), 1.54–1.19 (m, 8H), 0.88 (t, *J* = 7.2 Hz, 3H), 0.83 (t, *J* = 7.2 Hz, 3H); ^{13}C NMR (100 MHz, $CDCl_3$): δ 170.7, 137.8, 132.8, 132.1, 128.6, 128.0, 127.1, 126.2, 119.6, 67.3, 64.3, 49.1, 47.8, 46.1, 45.8, 31.8, 31.7, 30.1, 29.9, 23.2, 20.4, 14.1, 14.0; IR (KBr) cm^{-1}: 2977, 2927, 1684, 1653, 879, 668; HRMS (APCI) $[M+H]^+$ calcd 383.3057, found 383.3068; Daicel Chiralpak IC-3 column, *n*-hexane/*i*-PrOH = 95/5, 0.8 mL/min, 254 nm: t_{major} = 26.938 min, t_{minor} = 30.912 min.

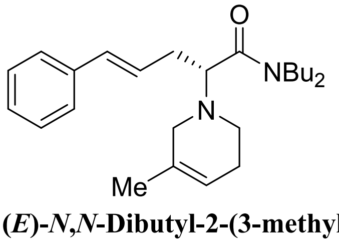

(*E*)-*N*,*N*-Dibutyl-2-(3-methyl-5,6-dihydropyridin-1(2*H*)-yl)-5-phenylpent-4-enamide (5h):

Yellow oil (32.5 mg, 85%). $[\alpha]_D^{20}$ = +64.9 (*c* 0.30, acetone); ^1H NMR (400 MHz, $CDCl_3$): δ

7.36–7.28 (m, 4H), 7.24–7.16 (m, 1H), 6.47 (d, *J* = 15.6 Hz, 1H), 6.27–6.14 (m, 1H), 5.50–5.38 (m, 1H), 3.62 (dd, *J* = 9.6, 4.0 Hz, 1H), 3.51–3.39 (m, 2H), 3.25–3.12 (m, 3H), 3.05–2.96 (m, 1H), 2.93–2.84 (m, 1H), 2.83–2.75 (m, 1H), 2.73–2.64 (m, 1H), 2.58–2.48 (m, 1H), 2.14–2.05 (m, 2H), 1.66 (s, 3H), 1.49–1.31 (m, 8H), 0.93–0.87 (m, 6H); ^{13}C NMR (100 MHz, CDCl$_3$): δ 170.5, 137.6, 132.5, 132.0, 128.4, 127.7, 126.9, 126.0, 119.4, 64.3, 52.8, 47.6, 45.9, 45.7, 31.6, 29.9, 29.7, 26.5, 22.7, 21.0, 20.3, 13.9, 13.8; IR (KBr) cm^{-1}: 2978, 1684, 1653, 1338, 879, 668; HRMS (APCI) [M+H]$^+$ calcd 383.3057, found 383.3074; Daicel Chiralpak IC-3 column, *n*-hexane/*i*-PrOH = 95/5, 0.8 mL/min, 254 nm: t_{major} = 35.416 min, t_{minor} = 30.117 min.

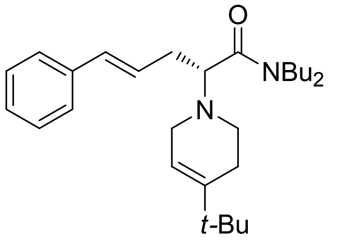

(*E*)-*N*,*N*-Dibutyl-2-(4-(tert-butyl)-5,6-dihydropyridin-1(2*H*)-yl)-5-phenylpent-4-enamide (5i):

Yellow oil (41.2 mg, 97%). $[\alpha]^{20}_{D}$ = +54.8 (*c* 0.25, acetone); ^{1}H NMR (400 MHz, CDCl$_3$): δ 7.27–7.20 (m, 4H), 7.19–7.09 (m, 1H), 6.41 (d, *J* = 15.6 Hz, 1H), 6.21–6.08 (m, 1H), 5.42–5.28 (m, 1H), 3.62–3.52 (m, 1H), 3.38–3.08 (m, 6H), 2.91–2.65 (m, 3H), 2.57–2.39 (m, 1H), 2.19–2.00 (m, 2H), 1.50–1.25 (m, 8H), 0.99 (s, 9H), 0.89–0.82 (m, 6H); ^{13}C NMR (100 MHz, CDCl$_3$): δ 170.6, 144.1, 137.5, 131.9, 128.3, 127.7, 126.8, 125.9, 115.8, 64.0, 49.2, 47.6, 46.0, 45.8, 34.8, 31.5, 29.9, 29.8, 28.7, 26.0, 20.3, 20.2, 13.9, 13.8; IR (KBr) cm^{-1}: 2978, 2926, 1680, 1653, 879, 669; HRMS (APCI) [M+H]$^+$ calcd 425.3526; found 425.3529; Daicel Chiralpak IC-3 column, *n*-hexane/*i*-PrOH = 95/5, 0.8 mL/min, 254 nm: t_{major} = 33.248 min, t_{minor} = 29.290 min.

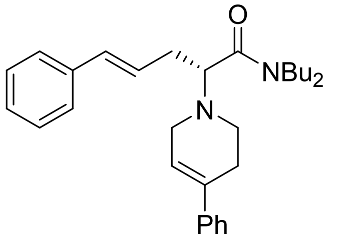

(*E*)-*N*,*N*-Dibutyl-5-phenyl-2-(4-phenyl-5,6-dihydropyridin-1(2*H*)-yl)pent-4-enamide (5j):

Yellow oil (42.3 mg, 95%). $[\alpha]^{20}_{D}$ = +46.6 (*c* 0.28, acetone); ^{1}H NMR (400 MHz, CDCl$_3$): δ 7.52–7.06 (m, 10H), 6.45 (d, *J* = 15.8 Hz, 1H), 6.24–6.12 (m, 1H), 6.10–5.96 (m, 1H), 3.70–3.60 (m, 1H), 3.53–3.07 (m, 6H), 3.06–2.74 (m, 3H), 2.65–2.37 (m, 3H), 1.54–1.21 (m, 8H), 0.89 (t, *J* = 7.6 Hz, 3H), 0.84 (t, *J* = 7.6 Hz, 3H); ^{13}C NMR (100 MHz, CDCl$_3$): δ 170.4, 140.8, 137.5, 134.8, 132.0, 128.4, 128.2, 127.5, 126.9, 126.8, 125.9, 124.7, 122.0, 64.0, 49.2, 47.6, 45.8, 45.8, 31.6, 29.8, 29.7, 29.6, 28.7, 20.2, 13.9, 13.8; IR (KBr) cm^{-1}: 2978, 2928, 1640, 1684, 1670, 689; HRMS (APCI) [M+H]$^+$ calcd 445.3213, found 445.3208; Daicel Chiralpak IC-3 column, *n*-hexane/*i*-PrOH = 95/5, 0.8 mL/min, 254 nm: t_{major} = 36.138 min, t_{minor} = 32.405 min.

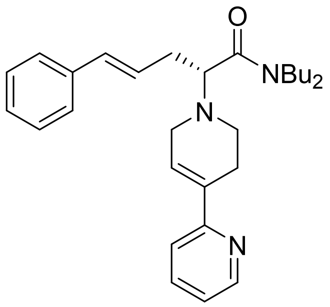

(*E*)-*N*,*N*-Dibutyl-2-(5',6'-dihydro-[2,4'-bipyridin]-1'(2'*H*)-yl)-5-phenylpent-4-enamide (5k):

Yellow oil (41.1 mg, 92%). $[\alpha]_D^{20}$ = +41.4 (*c* 0.16, acetone); ^{1}H NMR (400 MHz, $CDCl_3$): δ 8.58–8.49 (m, 1H), 7.60 (t, J = 8.0 Hz, 1H), 7.35–7.07 (m, 7H), 6.67–6.57 (m, 1H), 6.44 (d, J = 15.6 Hz, 1H), 6.24–6.10 (m, 1H), 3.70–3.59 (m, 1H), 3.58–3.31 (m, 4H), 3.22–3.06 (m, 2H), 3.04–2.79 (m, 3H), 2.68–2.43 (m, 3H), 1.54–1.26 (m, 8H), 0.88 (t, J = 7.2 Hz, 3H), 0.82 (t, J = 7.2 Hz, 3H); ^{13}C NMR (100 MHz, $CDCl_3$): δ 170.2, 157.4, 148.8, 137.5, 136.2, 134.7, 132.0, 128.3, 127.6, 126.9, 125.9, 125.7, 121.6, 118.8, 64.0, 49.0, 47.5, 45.9, 45.8, 31.6, 30.1, 29.6, 29.2, 27.3, 20.2, 13.9, 13.8; IR (KBr) cm^{-1}: 2978, 2924, 1684, 1670, 1046, 879; HRMS (APCI) $[M+H]^+$ calcd 446.3166, found 446.3179; Daicel Chiralpak AD-H column, *n*-hexane/*i*-PrOH = 98.5/1.5, 1 mL/min, 254 nm: t_{major} = 42.850 min, t_{minor} = 50.916 min.

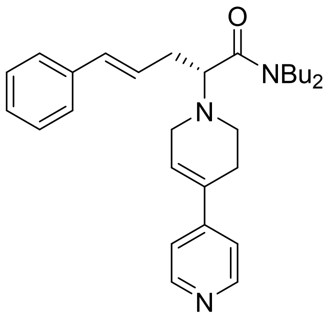

(*E*)-*N*,*N*-Dibutyl-2-(5,6-dihydro-[4,4'-bipyridin]-1(2*H*)-yl)-5-phenylpent-4-enamide (5l):

Yellow oil (42.8 mg, 96%). $[\alpha]_D^{20}$ = +31.1 (*c* 0.18, acetone); ^{1}H NMR (400 MHz, $CDCl_3$): δ 8.55 (d, J = 5.6 Hz, 2H), 7.35–7.15 (m, 7H), 6.49 (d, J = 15.6 Hz, 1H), 6.40–6.30 (m, 1H), 6.27–6.13 (m, 1H), 3.74–3.64 (m, 1H), 3.58–3.36 (m, 4H), 3.27–3.15 (m, 2H), 3.07–2.84 (m, 3H), 2.63–2.45 (m, 3H), 1.50–1.25 (m, 8H), 0.93 (t, J = 7.2 Hz, 3H), 0.88 (t, J = 7.2 Hz, 3H); ^{13}C NMR (100 MHz, $CDCl_3$): δ 170.3, 149.9, 147.7, 137.4, 132.8, 132.2, 128.4, 127.2, 127.0, 126.0, 125.9, 119.3, 63.7, 49.1, 47.6, 45.9, 45.6, 31.6, 29.9, 29.7, 29.7, 27.8, 20.2, 13.9, 13.8; IR (KBr) cm^{-1}: 2978, 1684, 1653, 1084, 879, 668; HRMS (APCI) $[M+H]^+$ calcd 446.3166, found 446.3156; Daicel Chiralpak AD-H column, *n*-hexane/*i*-PrOH = 95/5, 1 mL/min, 254 nm: t_{major} = 30.526 min, t_{minor} = 38.624 min.

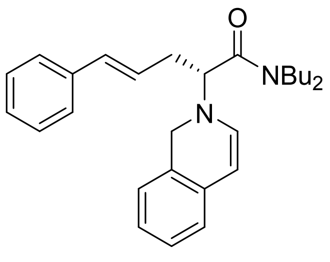

(*E*)-*N*,*N*-Dibutyl-2-(isoquinolin-2(1*H*)-yl)-5-phenylpent-4-enamide (5m): Yellow oil (36.9 mg, 88%). $[\alpha]_D^{20}$ = +44.6 (*c* 0.26, acetone); ^{1}H NMR (400 MHz, $CDCl_3$): δ 8.39 (d, *J* = 8.0 Hz, 1H), 7.60 (t, *J* = 7.2 Hz, 1H), 7.52–7.33 (m, 3H), 7.22–6.95 (m, 4H), 6.52 (d, *J* = 7.6 Hz, 1H), 6.45 (d, *J* = 15.6 Hz, 1H), 6.20–6.08 (m, 2H), 3.56–3.28 (m, 3H), 3.29–3.05 (m, 3H), 3.07–2.97 (m, 1H), 2.96–2.81 (m, 1H), 2.76–2.63 (m, 1H), 1.53–1.24 (m, 8H), 0.86 (t, *J* = 6.8 Hz, 3H), 0.72 (t, *J* = 6.8 Hz, 3H); ^{13}C NMR (100 MHz, $CDCl_3$): δ 168.7, 161.3, 137.0, 136.6, 133.7, 132.4, 128.3, 128.1, 128.0, 127.2, 126.8, 126.1, 125.9, 124.6, 106.7, 52.3, 47.5, 46.2, 36.1, 31.4, 31.2, 29.5, 20.1, 19.8, 13.7, 13.6; IR (KBr) cm^{-1}: 2978, 2928, 1698, 1684, 1085, 879; HRMS (APCI) $[M+H]^+$ calcd 417.2900, found 417.2897; Daicel Chiralpak IC-3 column, *n*-hexane/*i*-PrOH = 95/5, 0.8 mL/min, 254 nm: t_{major} = 17.766 min, t_{minor} = 26.716 min.

5. Derivatization of 4a, 4q, 5f, 5j and 10

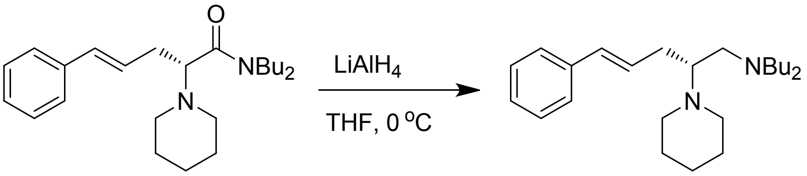

4a 95% ee → **6** 91%

To a solution of **4a** (37.1 mg, 0.1 mmol) in anhydrous THF (1.0 mL) under N_2 was added $LiAlH_4$ (16.0 mg, 0.4 mmol) at 0 °C and the mixture was stirred at RT overnight. The reaction mixture was quenched with ethanol, diluted with DCM, washed with brine, dried over Na_2SO_4 and concentrated in vacuo. The residue was purified by flash column chromatography to give the yellow oil **6** in 91% yield. The two enantiomers are difficult to be separated on several kinds of chiral column in HPLC analysis, the reported similar reactions showed no loss in ee.[4] $[\alpha]_D^{25}$ = +45.9 (*c* 0.3, acetone); ^{1}H NMR (400 MHz, $CDCl_3$): δ 7.34–7.17 (m, 5H), 6.41–6.24 (m, 2H), 2.78–2.09 (m, 13H), 1.56–1.24 (m, 14H), 0.87 (t, *J* = 7.2 Hz, 6H); ^{13}C NMR (100 MHz, $CDCl_3$): δ 138.0, 133.1, 130.3, 128.4, 126.6, 125.8, 63.6, 54.4, 50.1, 32.9, 29.6, 29.1, 26.6, 25.0, 20.7, 14.1; IR (KBr) cm^{-1}: 2978, 2928, 1748, 1489 1045, 879, 668; HRMS (APCI) $[M+H]^+$ calcd 357.3264, found 357.3275.

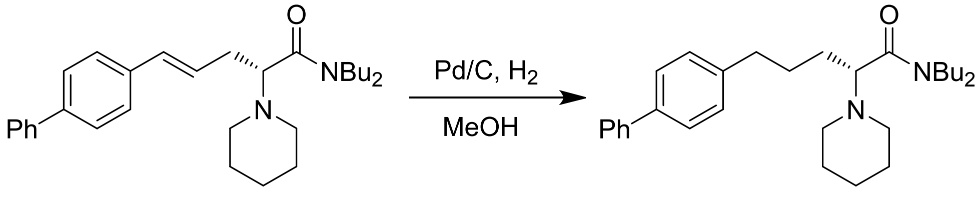

4q 88% ee → **7** 83%, 88% ee

To a solution of **4q** (44.7 mg, 0.1 mmol) in MeOH (2.0 mL) was added Pd/C (10%, 10 mg) and the mixture was stirred under H_2 atmosphere (20 bar) at room temperature for 20 h. The reaction mixture was diluted with DCM, filtered, dried over anhydrous Na_2SO_4 and concentrated in vacuo. The residue was purified by flash column chromatography to give **7** as a yellow oil (83%, 88% ee).

$[\alpha]^{25}$ = +8.3 (*c* 0.50, acetone); ^{1}H NMR (400 MHz, $CDCl_3$): *δ* 7.57 (d, *J* = 8.0 Hz, 2H), 7.50 (d, *J* = 8.0 Hz, 2H), 7.42 (t, *J* = 7.6 Hz, 2H), 7.34–7.29 (m, 1H), 7.25–7.20 (m, 2H), 3.56–3.39 (m, 2H), 3.38–3.25 (m, 1H), 3.21–3.04 (m, 2H), 2.80–2.52 (m, 4H), 2.55–2.36 (m, 2H), 1.64–1.25 (m, 18H), 0.98–0.83 (m, 6H); ^{13}C NMR (100 MHz, $CDCl_3$): *δ* 171.0, 141.6, 141.1, 138.6, 128.8, 128.7, 127.0, 126.9, 126.8, 65.0, 50.3, 47.3, 45.6, 35.8, 31.6, 29.8, 28.9, 26.5, 25.0, 24.5, 20.3, 20.2, 13.9; IR (KBr) cm^{-1}: 2920, 2850, 1635, 1558, 1471 1373, 1265, 667; HRMS (APCI) $[M+H]^+$ calcd 449.3526, found 449.3527; Daicel Chiralpak IC-3 column, *n*-hexane /*i*-PrOH = 95/5, 0.8 mL/min, 254 nm: t_{major} = 14.175 min, t_{minor} = 12.130 min.

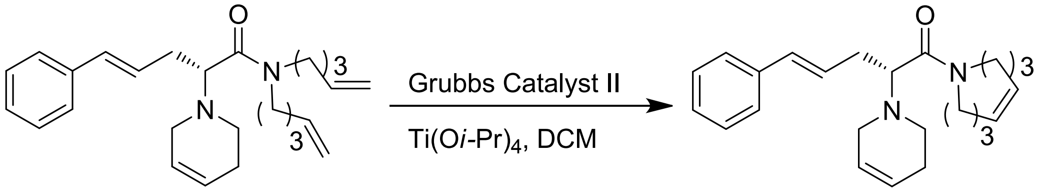

5f
90% ee

8
81%, 90% ee

To a solution of **5f** (39.5 mg, 0.1 mmol) in anhydrous DCM (20 mL) under N_2 was added fresh distilled Ti(O*i*-Pr)$_4$ (28.4 mg, 0.1 mmol) and stirred for 10 min, then Grubbs Catalyst II (8.5 mg, 0.01 mmol) was added. The resulting mixture was stirred under reflux for 10 h. The reaction mixture was concentrated in vacuo. The residue was purified by flash column chromatography to give the compound **8** (81%, 90% *ee* and E/Z = 2:1).[5] $[\alpha]^{20}$ = +10.0 (*c* 0.15, acetone); ^{1}H NMR (400 MHz, $CDCl_3$): *δ* 7.34–7.11 (m, 5H), 6.46 (d, *J* = 16.0 Hz, 1H), 6.26–6.07 (m, 1H), 5.81–5.62 (m, 4H), 4.10–4.02 (m, 2H), 3.55–3.30 (m, 7H), 2.28–1.71 (m, 12H); ^{13}C NMR (100 MHz, $CDCl_3$, major isomer): *δ* 170.8, 137.6, 132.0, 131.6, 128.4, 128.2, 128.1, 126.9, 125.9, 125.6, 125.0, 65.4, 53.4, 50.6, 48.3, 47.6, 45.9, 28.3, 27.8, 26.8, 25.9, 23.6; ^{13}C NMR (101 MHz, $CDCl_3$, minor isomer) *δ* 171.6, 137.5, 132.3, 130.0, 129.7, 127.2, 127.1, 127.0, 125.9, 125.6, 125.2, 63.7, 49.3, 48.7, 47.9, 47.3, 41.6, 30.2, 29.5, 26.9, 26.6, 23.2; IR (KBr) cm^{-1}: 2979, 1684, 1670, 1045, 879, 668; HRMS (APCI) $[M+H]^+$ calcd 365.2587, found 365.2591; Daicel Chiralpak AD-H column, *n*-hexane /*i*-PrOH = 98/2, 0.8 mL/min, 254 nm: t_{major} = 24.955 min, t_{minor} = 18.207 min.

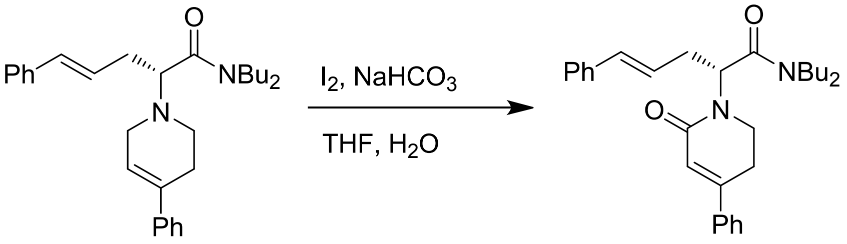

5j
88% ee

9
98%, 88% ee

To a solution of **5j** (44.5 mg, 0.1 mmol) in THF (10.0 mL) and H_2O (4 mL) was added $NaHCO_3$ (84 mg, 1 mmol) and I_2 (190 mg, 0.75 mmol). The mixture was stirred at RT for 12 h. The reaction mixture was quenched with saturated Na_2SO_3 and saturated $NaHCO_3$, extracted with DCM, dried over Na_2SO_4 and concentrated in vacuo. The residue was purified by flash column chromatography to give the compound **9** (98%, 88% *ee*).[6] $[\alpha]^{20}$ = +101.3 (*c* 0.3, acetone); ^{1}H

NMR (400 MHz, $CDCl_3$) δ 7.57–7.09 (m, 10H), 6.51 (d, J = 15.6 Hz, 1H), 6.30 (s, 1H), 6.22–6.06 (m, 1H), 5.66 (t, J = 7.2 Hz, 1H), 3.75–3.65 (m, 1H), 3.61–3.41 (m, 3H), 3.25–3.08 (m, 2H), 2.87–2.58 (m, 4H), 1.61–1.43 (m, 4H), 1.39–1.21 (m, 4H), 1.02–0.75 (m, 6H); ^{13}C NMR (101 MHz, $CDCl_3$) δ 169.8, 164.7, 150.3, 137.5, 137.4, 133.3, 129.8, 128.9, 128.7, 127.4, 126.4, 125.9, 119.7, 51.3, 47.5, 46.0, 40.6, 33.5, 31.7, 29.9, 27.0, 20.4, 20.2, 14.1, 14.1; IR (KBr) cm^{-1}: 2978, 2924, 1654, 1647 1374, 1045, 879, 668; HRMS (APCI) $[M+H]^+$ calcd 459.3006, found 459.3023; Daicel Chiralpak AD-H column, *n*-hexane /*i*-PrOH = 95/5, 1 mL/min, 254 nm: t_{major} = 22.878 min, t_{minor} = 40.089 min.

6. Synthesis of Dipeptide and Unnatural Amino Acid

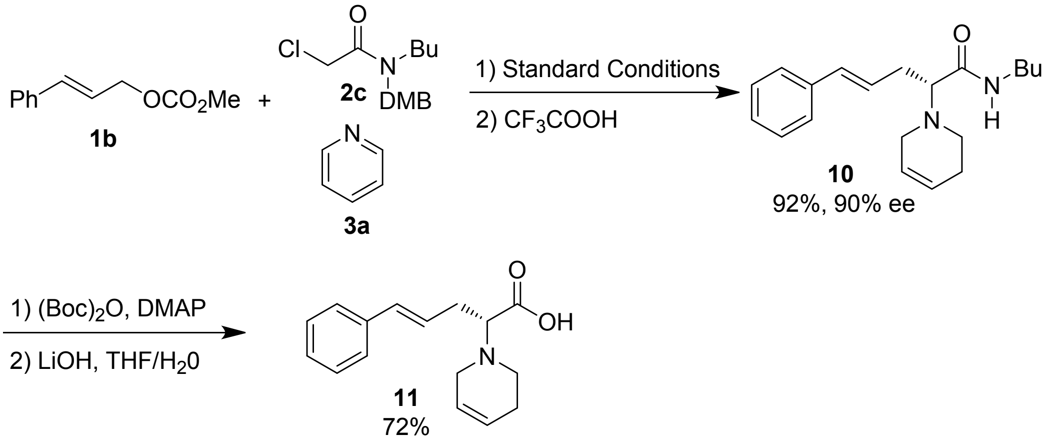

The reaction of **1b** (0.1 mmol), **2e** (0.2 mmol) and **3a** (0.5 mmol) was conducted using general procedure. The reaction mixture was filtrated through a short column of silica gel to remove DMSO and concentrated under vacuum. To the crude product was added TFA (2 mL) and the mixture was then stirred at 60 °C for 6 h. TFA was evaporate under vacuo and TEA (2 mL) was added. The mixture was stirred for further 0.5 h. The reaction mixture was diluted with DCM, the organic phase was washed with saturate $NaHCO_3$, dried over anhydrous Na_2SO_4 and concentrated in vacuo. The residue was purified by flash column chromatography (PE/EA = 5/1) to give **10** (92%, 90% *ee*). Mp: 39–40 °C. $[\alpha]_D^{20}$ = –14.9 (*c* 0.20, MeOH); ^1H NMR (400 MHz, $CDCl_3$) δ 7.40–7.08 (m, 6H), 6.44 (d, J = 15.6 Hz, 1H), 6.29 (dd, J = 15.6, 8.4 Hz, 1H), 5.82–5.61 (m, 2H), 3.40–2.96 (m, 5H), 2.87–2.73 (m, 1H), 2.73–2.49 (m, 3H), 2.24–2.04 (m, 2H), 1.51–1.37 (m, 2H), 1.31–1.22 (m, 2H), 0.92–0.79 (m, 3H); ^{13}C NMR (101 MHz, $CDCl_3$) δ 172.5, 137.6, 132.1, 128.6, 127.6, 127.2, 126.2, 125.5, 125.4, 69.1, 49.8, 47.7, 38.9, 32.0, 31.8, 26.7, 20.3, 13.9; IR (KBr) cm^{-1}: 2974, 1843, 1715, 1646, 1615, 1435, 766, 720; HRMS (APCI) $[M+H]^+$ calcd 313.2274, found 313.2291; Daicel Chiralpak OD-H column, *n*-hexane /*i*-PrOH = 95/5, 0.8 mL/min, 254 nm: t_{major} = 24.966 min, t_{minor} = 17.239 min.

10 (0.13 mmol) and DMAP (0.26 mmol) were dissolved in $(Boc)_2O$ (2 mL). The mixture was stirred at RT overnight before another portion of DMAP (0.13 mmol) and $(Boc)_2O$ (2 mL) were added and the mixture was stirred at 80 °C for another 2 h. The reaction mixture was directly purified by flash column chromatography (PE/EA = 20/1) to give Boc-protected **10**, which was dissolved in THF (3 mL) followed by the addition of water (1 mL) and LiOH (0.78 mmol). The mixture was stirred at RT for 2 days. The reaction was quenched with acetic acid, concentrated in vacuo. The residue was purified by flash column chromatography (DCM/MeOH = 15/1) to give

the compound **11** (72%). mp: 46–48 °C. $[\alpha]_D^{20}$ = –4.0 (*c* 0.2, MeOH); ^{1}H NMR (400 MHz, D_2O) δ 7.49–7.04 (m, 5H), 6.50 (d, *J* = 15.6 Hz, 1H), 6.15–6.00 (m, 1H), 5.91–5.76 (m, 1H), 5.65–5.48 (m, 1H), 3.79–3.49 (m, 3H), 3.42–3.10 (m, 2H), 2.88–2.62 (m, 2H), 2.46–2.15 (m, 2H); ^{13}C NMR (101 MHz, D_2O) δ 172.4, 136.7, 133.8, 128.8, 127.9, 126.3, 125.6, 123.0, 119.4, 68.7, 48.9, 48.7, 31.1, 22.3; IR (KBr) cm^{-1}: 3502, 1868, 1791, 1683, 1635, 1456, 457, 418; HRMS (APCI) $[M+H]^+$ calcd 258.1489; found 258.1501.

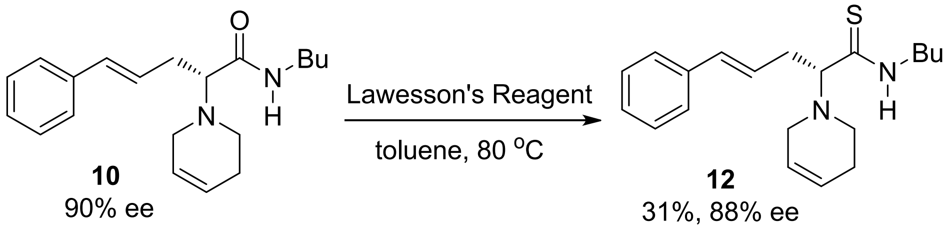

To a solution of **10** (31.2 mg, 0.1 mmol) in toluene (2.0 mL) was added Lawesson's reagent (80.8 mg, 0.2 mmol) under N_2 atmosphere and the mixture was stirred at 80 °C for 20 h. The reaction mixture was diluted with DCM, washed with saturated $NaHCO_3$, dried over anhydrous Na_2SO_4 and concentrated in vacuo. The residue was purified by flash column chromatography to give **12** as a yellow oil (31%, 88% ee). $[\alpha]_D^{25}$ = +22.9 (*c* 0.20, acetone); ^{1}H NMR (400 MHz, $CDCl_3$): δ 9.15 (s, 1H), 7.35–7.16 (m, 5H), 6.44 (d, *J* = 15.6 Hz, 1H), 6.21–6.04 (m, 1H), 5.87–5.72 (m, 1H), 5.73–5.61 (m, 1H), 3.73–3.48 (m, 3H), 3.43–3.19 (m, 1H), 3.15–2.93 (m, 2H), 2.89–2.53 (m, 3H), 2.27–2.02 (m, 2H), 1.63–1.46 (m, 2H), 1.40–1.26 (m, 2H), 0.85 (t, *J* = 7.2 Hz, 3H); ^{13}C NMR (100 MHz, $CDCl_3$): δ 202.0, 137.2, 132.8, 132.7, 128.5, 127.3, 126.1, 125.3, 125.1, 49.7, 49.6, 48.2, 44.8, 34.9, 30.1, 26.3, 20.3, 13.7; IR (KBr) cm^{-1}: 2920, 2850, 1683, 1635 1521, 1265, 895; HRMS (APCI) $[M+H]^+$ calcd 329.2046, found 329.2045; Daicel Chiralpak IC-3 column, *n*-hexane /*i*-PrOH = 95/5, 1 mL/min, 254 nm: t_{major} = 11.591 min, t_{minor} = 13.361 min.

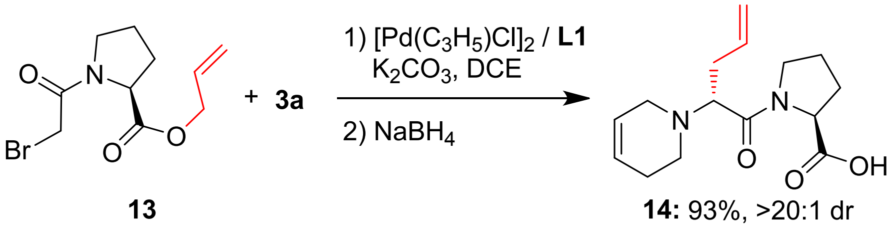

To an oven-dried glassware were added $[Pd(C_3H_5)Cl]_2$ (0.9 mg, 2.5 mol%), **L1** (4.8 mg, 5.5 mol%) and anhydrous DCE (1.0 mL). To another dry glassware were added **13** (0.1 mmol), **3a** (0.2 mmol), and anhydrous DCE (0.5 mL). Then the first glassware was stirred at RT and the second glassware was reflux under a nitrogen atmosphere for 1 h. After cooling to RT, anhydrous K_2CO_3 (0.1 mmol) was added to the second glassware. The solution in the first glassware was added dropwise to another one. The reaction was stirred at RT for 12 h. The solvent was removed in vacuo. THF (1 mL) was added and the resulting mixture was cooled to –20 °C. Then methanol (1 mL) was added and the mixture was stirred for 5 min. $NaBH_4$ (0.5 mmol) was added in one portion and the reaction was stirred for 2 h. The reaction was quenched with acetic acid, and then was concentrated in vacuo. The residue was purified by flash column chromatography to give the compound **14** (93%, >20:1 dr, determined by the ^{1}H-NMR of the crude reaction mixture). $[\alpha]_D^{20}$

= –18.0 (*c* 0.10, acetone); ^{1}H NMR (400 MHz, $CDCl_3$) δ 6.01–5.50 (m, 3H), 5.06 (d, *J* = 16.4 Hz, 1H), 4.98 (d, *J* = 9.6 Hz, 1H), 4.33–4.18 (m, 1H), 3.90–2.79 (m, 7H), 2.81–2.27 (m, 4H), 2.08–1.80 (m, 4H); ^{13}C NMR (101 MHz, $CDCl_3$) δ 179.1, 170.8, 135.8, 125.5, 125.1, 116.9, 65.6, 62.3, 49.0, 47.5, 46.0, 31.8, 29.3, 26.5, 25.1; IR (KBr) cm^{-1}: 2977, 2923, 1748, 1716 1271, 1045, 879; HRMS (APCI) $[M+H]^+$ calcd 279.1703, found 279.1716.

7. References

[1] X. Huo, J. Zhang, J. Fu, R. He and W. Zhang, *J. Am. Chem. Soc.*, 2018, **140**, 2080.

[2] J. D. Sivey and A. L. Roberts, *Environ. Sci. Technol.*, 2012, **46**, 2187.

[3] K. Moonen, C. V. Stevens, *Synthesis*, 2005, **20**, 3603.

[4] (a) K. Ishihara and K. Nakano, *J. Am. Chem. Soc*., 2005, **127**, 10504; (b) J. Roane, J. Wippich, S. D. Ramgren and M. J. Krische, *Org. Lett.*, 2017, **19**, 6634.

[5] Q. Yang, W.-J. Xiao and Z. Yu, *Org. Lett.*, 2005, **7**, 871.

[6] R. J. Griffiths, G. A. Burley and E. P. A. Talbot, *Org. Lett.*, 2017, **19**, 870.

8. NMR Spectra

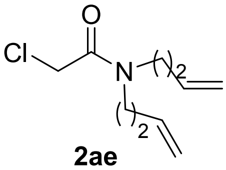

2ae

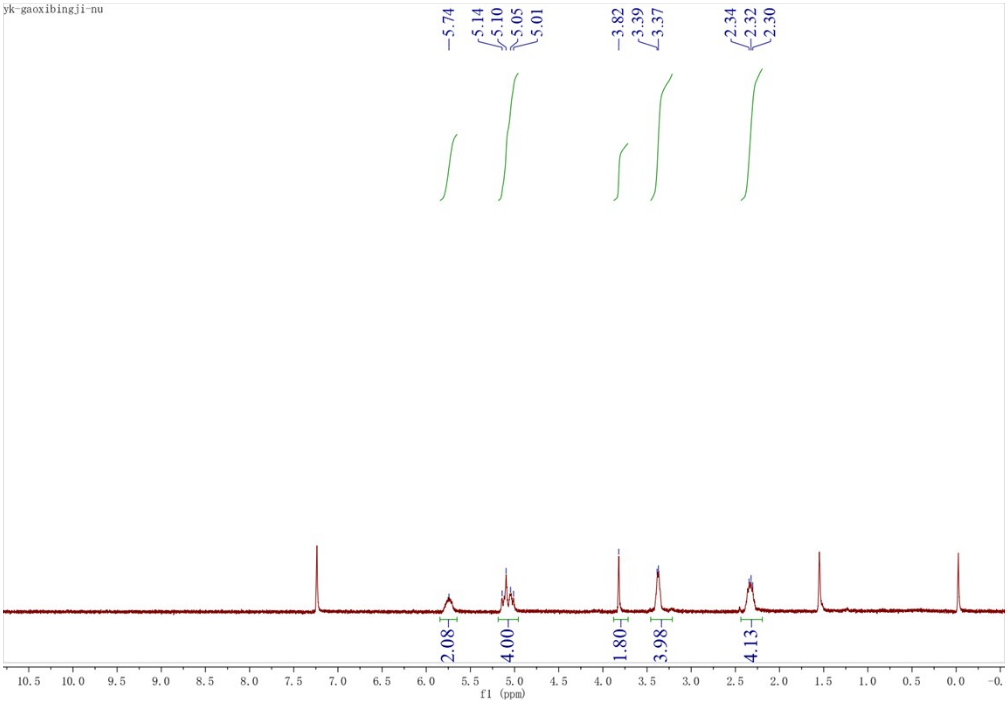

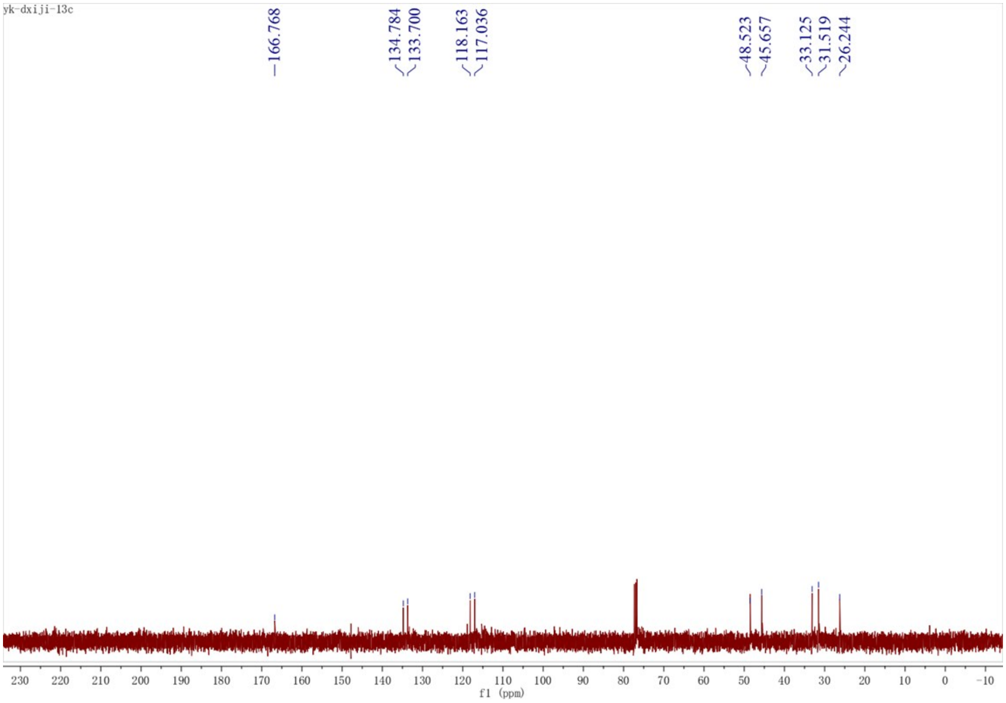

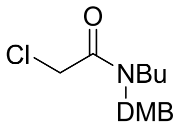
O
Cl
NBu
DMB

2c

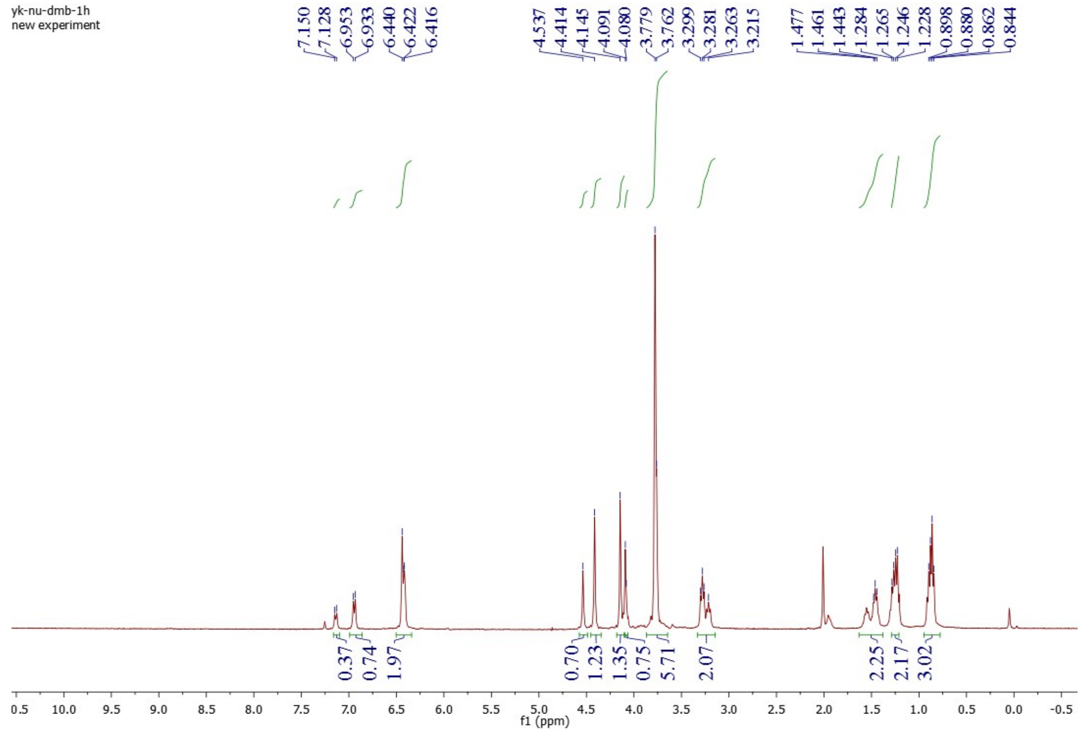
yk-nu-dmb-1h
new experiment
7.150
7.128
6.953
6.933
6.440
6.422
6.416
4.537
4.414
4.145
4.091
4.080
3.779
3.762
3.299
3.281
3.263
3.215
1.477
1.461
1.443
1.284
1.265
1.246
1.228
0.898
0.880
0.862
0.844
0.37
0.74
1.97
0.70
1.23
1.35
0.75
5.71
2.07
2.25
2.17
3.02
f1 (ppm)

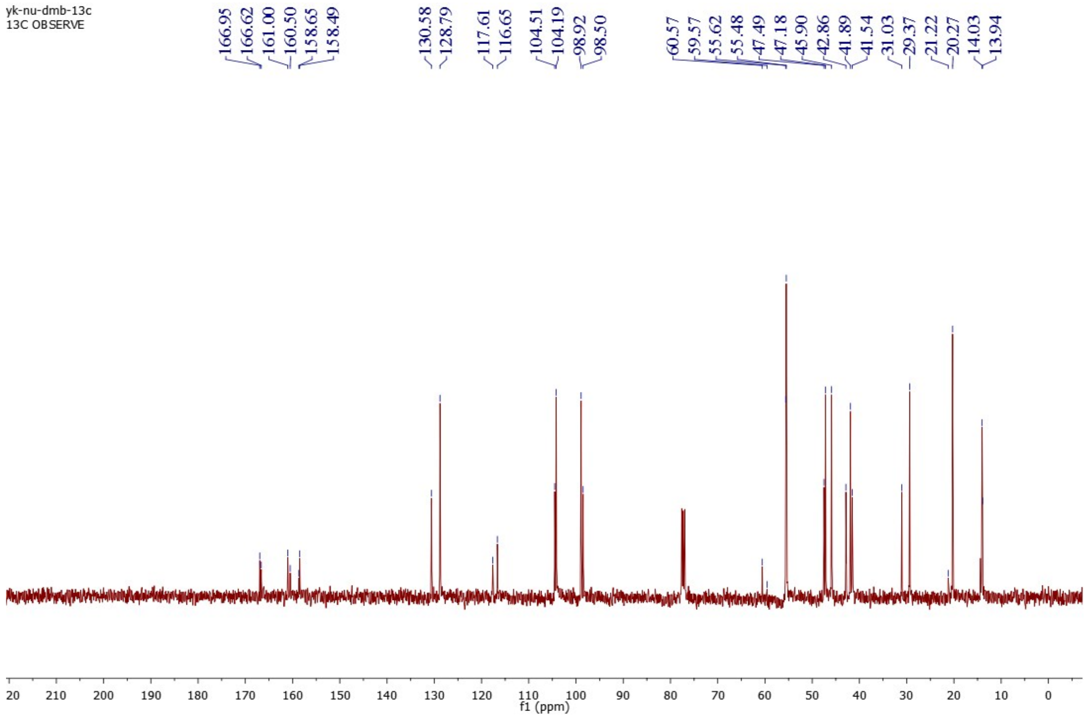
yk-nu-dmb-13c
13C OBSERVE
166.95
166.62
161.00
160.50
158.65
158.49
130.58
128.79
117.61
116.65
104.51
104.19
98.92
98.50
60.57
59.57
55.62
55.48
47.49
47.18
45.90
42.86
41.89
41.54
31.03
29.37
21.22
20.27
14.03
13.94
f1 (ppm)

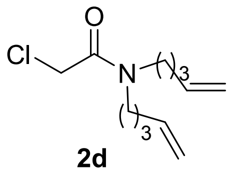

2d

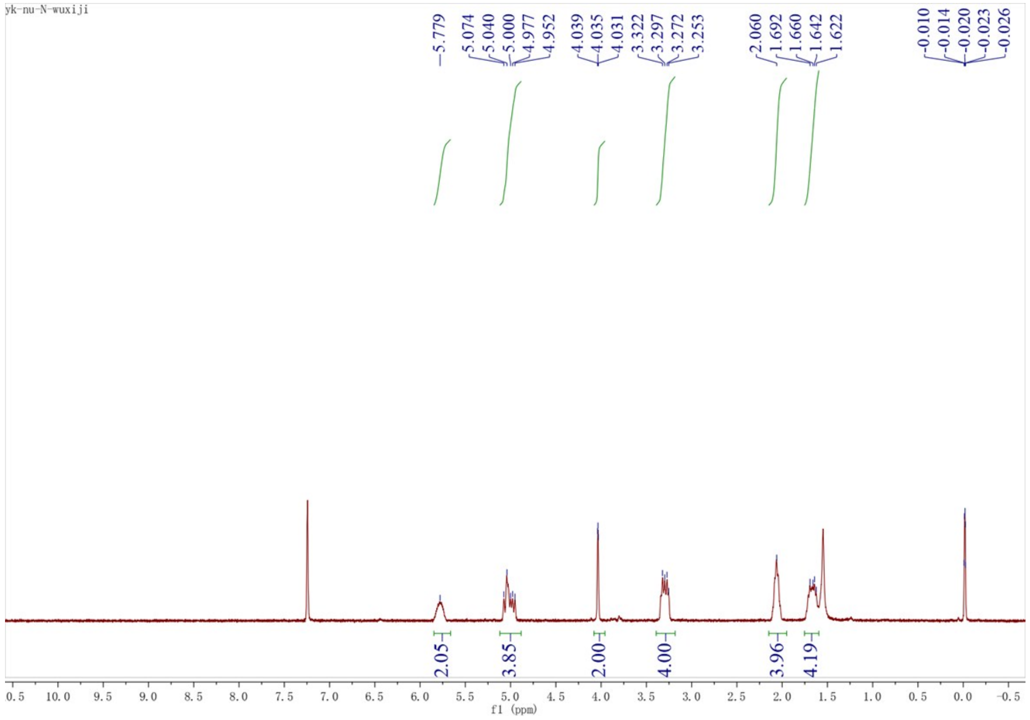
yk-nu-N-wuxiji
5.779
5.074
5.040
5.000
4.977
4.952
4.039
4.035
4.031
3.322
3.297
3.272
3.253
2.060
1.692
1.660
1.642
1.622
-0.010
-0.014
-0.020
-0.023
-0.026
2.05
3.85
2.00
4.00
3.96
4.19
f1 (ppm)

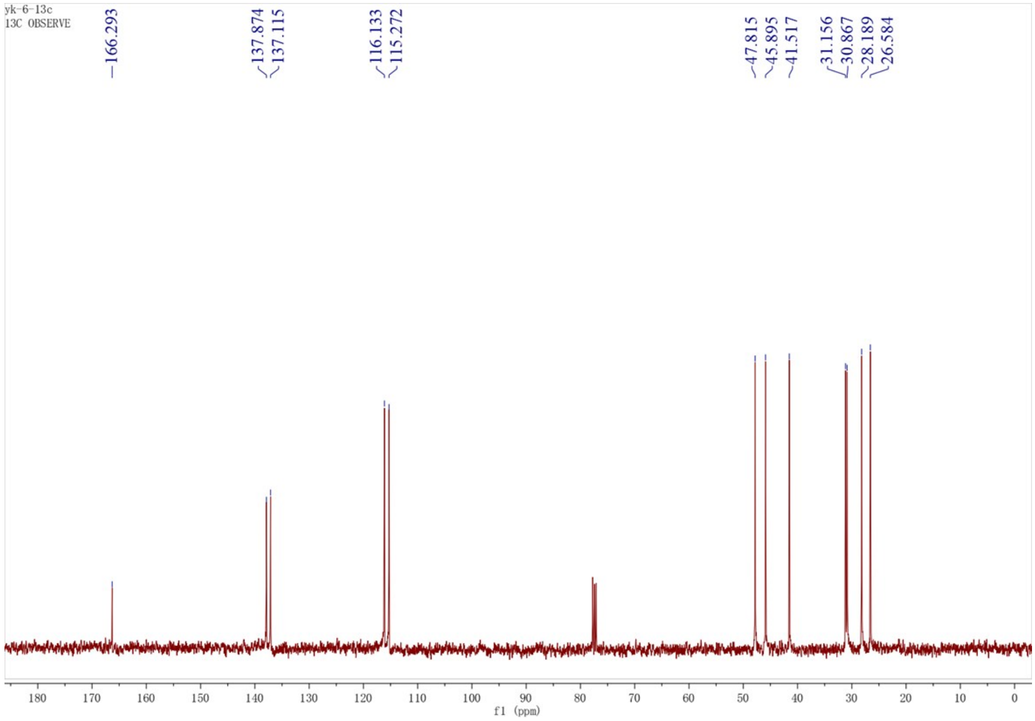
yk-6-13c
13C OBSERVE
166.293
137.874
137.115
116.133
115.272
47.815
45.895
41.517
31.156
30.867
28.189
26.584
f1 (ppm)

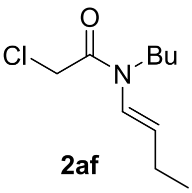

2af

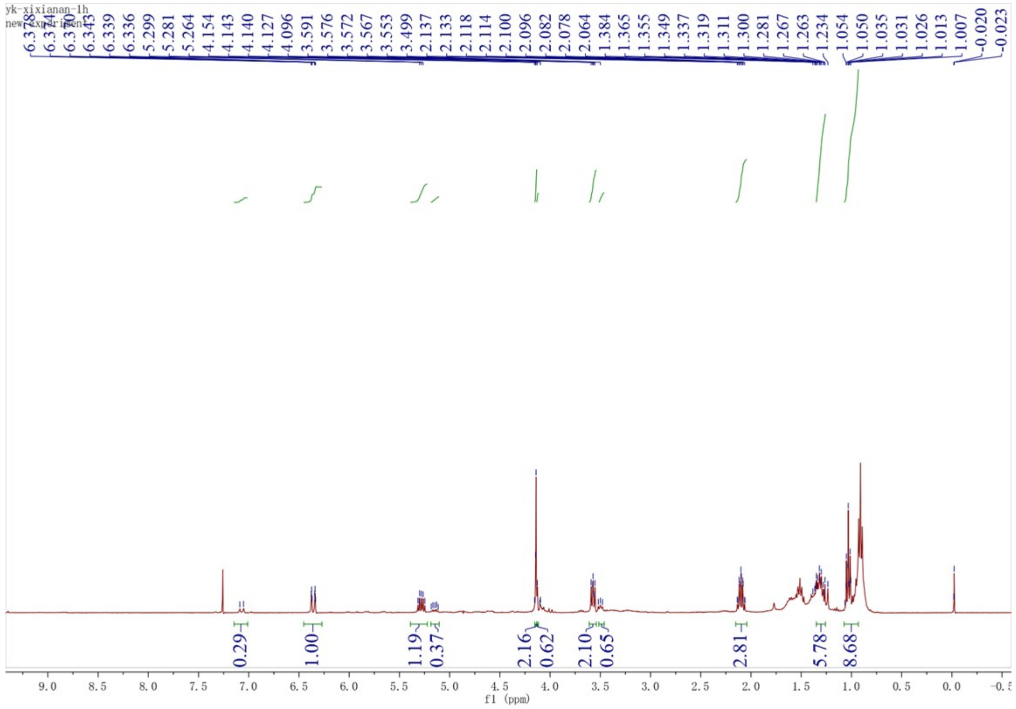

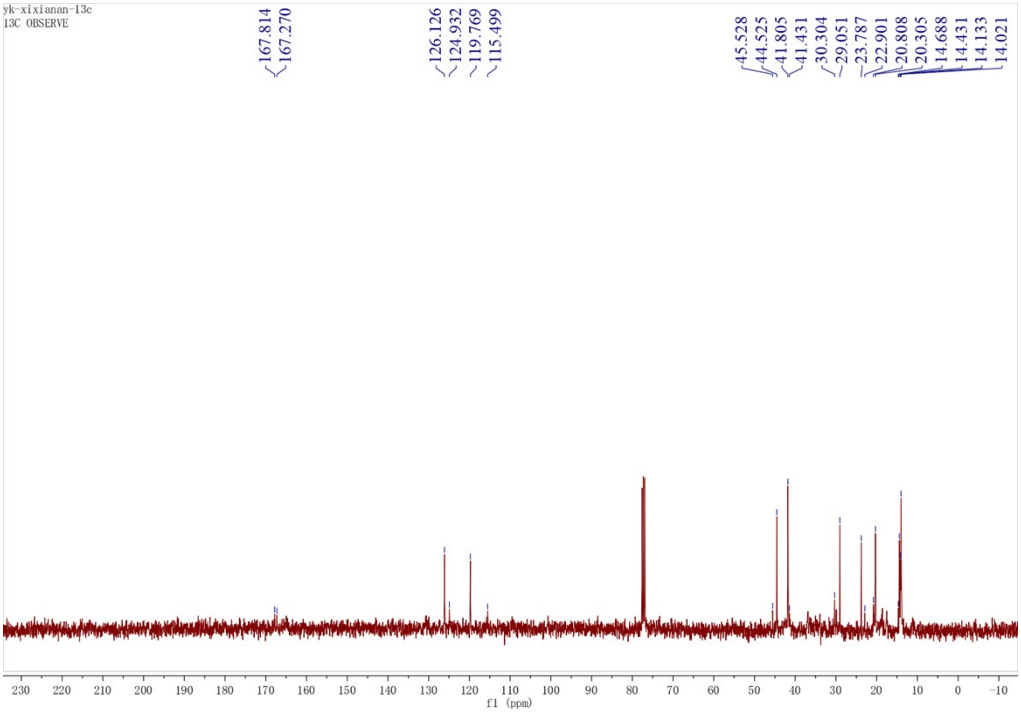

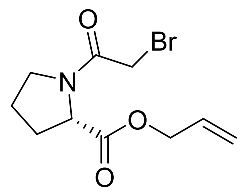
O
Br
N
O
O

13

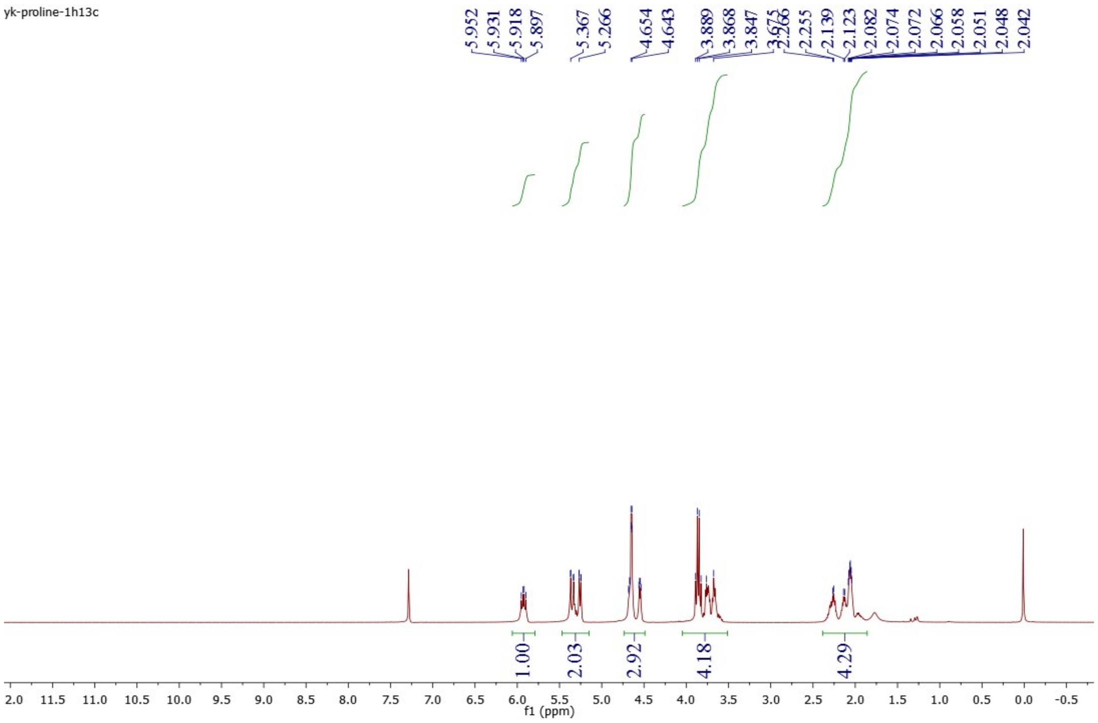
yk-proline-1h13c
5.952
5.931
5.918
5.897
5.367
5.266
4.654
4.643
3.889
3.868
3.847
3.675
2.266
2.255
2.139
2.123
2.082
2.074
2.072
2.066
2.058
2.051
2.048
2.042
1.00
2.03
2.92
4.18
4.29
f1 (ppm)

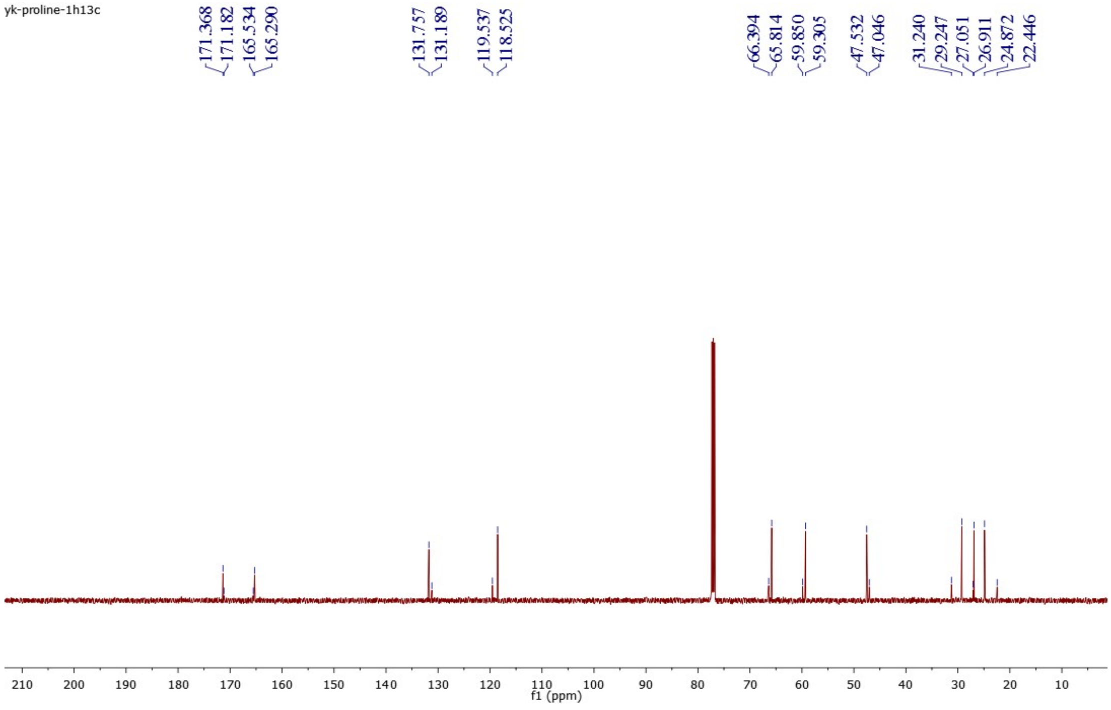
yk-proline-1h13c
171.368
171.182
165.534
165.290
131.757
131.189
119.537
118.525
66.394
65.814
59.850
59.305
47.532
47.046
31.240
29.247
27.051
26.911
24.872
22.446
f1 (ppm)

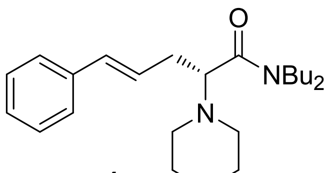

4a

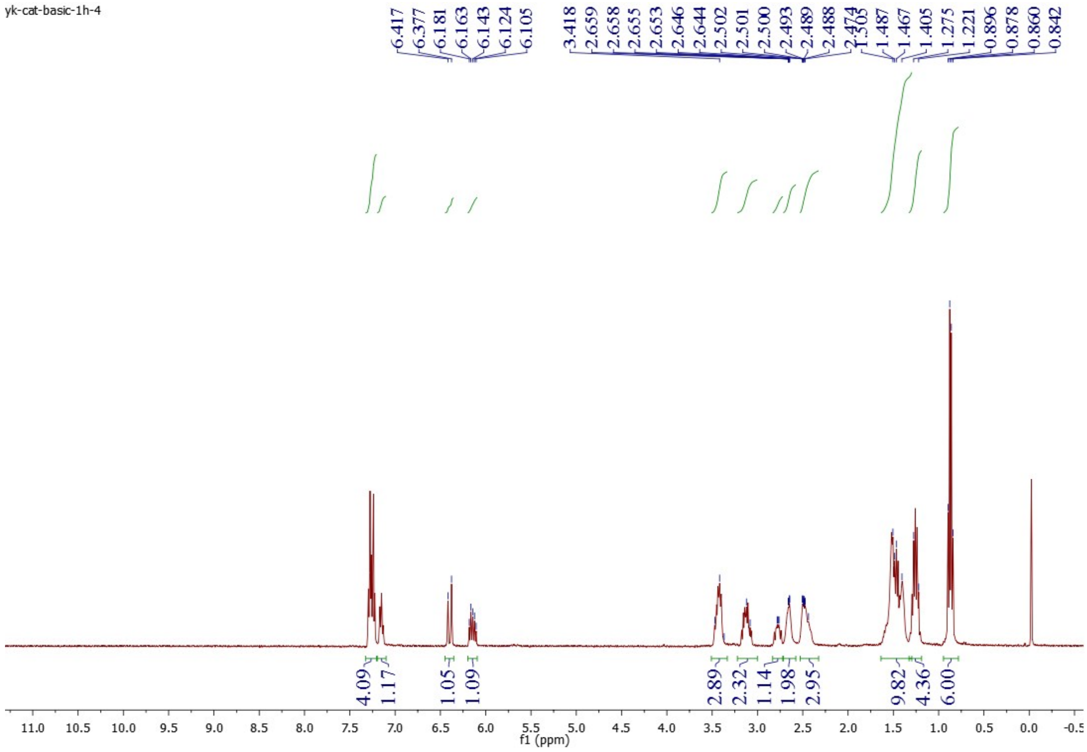
yk-cat-basic-1h-4
6.417
6.377
6.181
6.163
6.143
6.124
6.105
3.418
2.659
2.658
2.655
2.653
2.646
2.644
2.502
2.501
2.500
2.493
2.489
2.488
2.474
1.505
1.487
1.467
1.405
1.275
1.221
0.896
0.878
0.860
0.842
4.09
1.17
1.05
1.09
2.89
2.32
1.14
1.98
2.95
9.82
4.36
6.00
f1 (ppm)

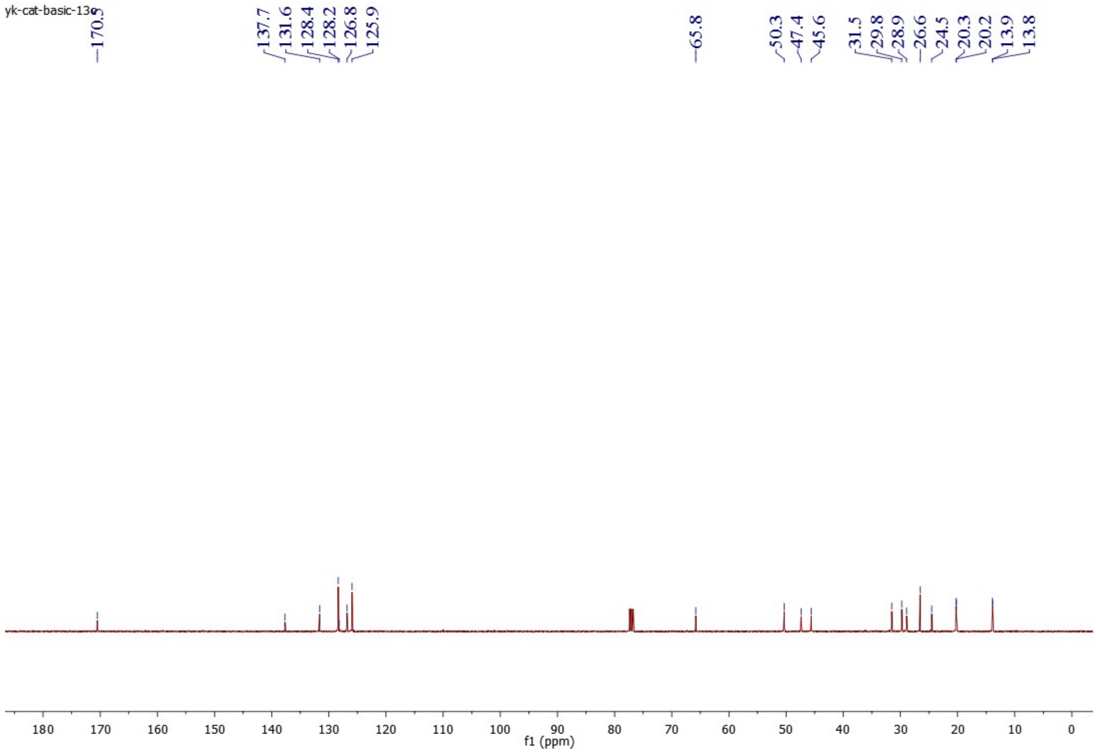
yk-cat-basic-13c
170.5
137.7
131.6
128.4
128.2
126.8
125.9
65.8
50.3
47.4
45.6
31.5
29.8
28.9
26.6
24.5
20.3
20.2
13.9
13.8
f1 (ppm)

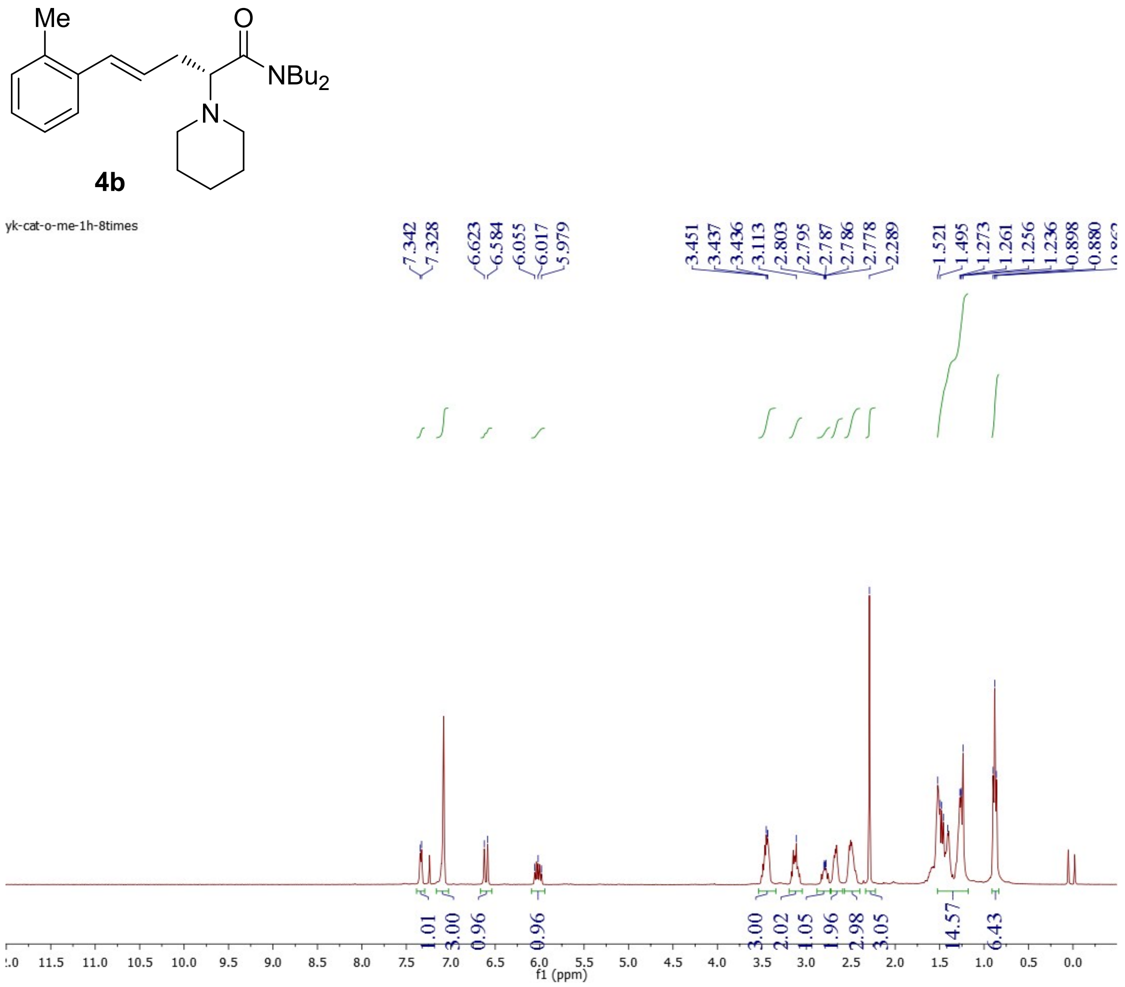
Me
O
NBu2
N
yk-cat-o-me-1h-8times
7.342
7.328
6.623
6.584
6.055
6.017
5.979
3.451
3.437
3.436
3.113
2.803
2.795
2.787
2.786
2.778
2.289
1.521
1.495
1.273
1.261
1.256
1.236
0.898
0.880
1.01
3.00
0.96
0.96
3.00
2.02
1.05
1.96
2.98
3.05
14.57
6.43
f1 (ppm)

4b

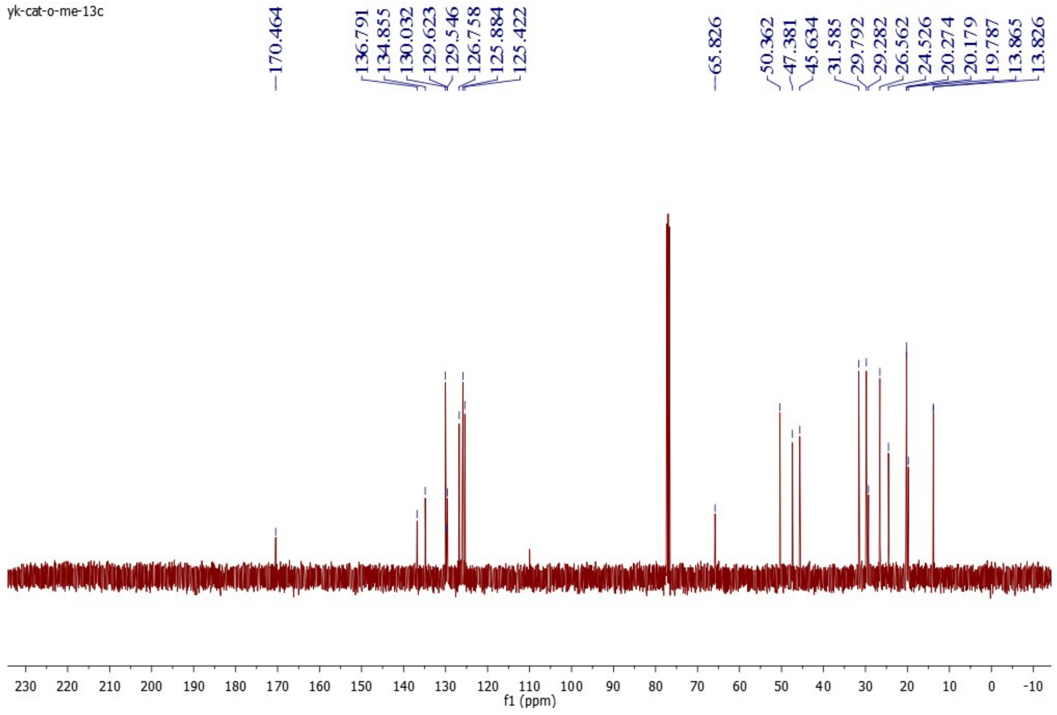
yk-cat-o-me-13c
170.464
136.791
134.855
130.032
129.623
129.546
126.758
125.884
125.422
65.826
50.362
47.381
45.634
31.585
29.792
29.282
26.562
24.526
20.274
20.179
19.787
13.865
13.826
f1 (ppm)

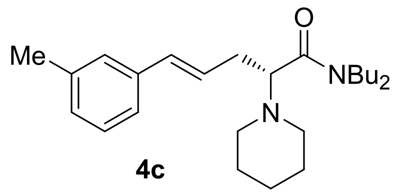
Me
O
NBu2
N

4c

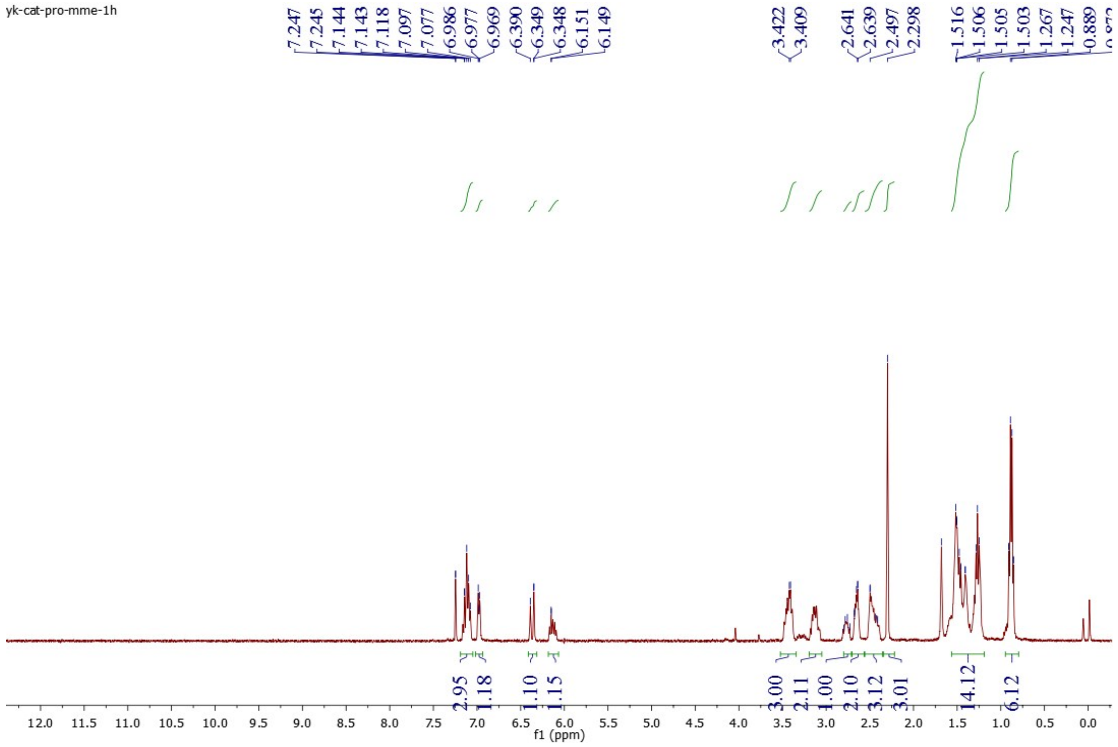
yk-cat-pro-mme-1h
7.247
7.245
7.144
7.143
7.118
7.097
7.077
6.986
6.977
6.969
6.390
6.349
6.348
6.151
6.149
3.422
3.409
2.641
2.639
2.497
2.298
1.516
1.506
1.505
1.503
1.267
1.247
0.889
[illegible]
2.95
1.18
1.10
1.15
3.00
2.11
1.00
2.10
3.12
3.01
14.12
6.12
f1 (ppm)

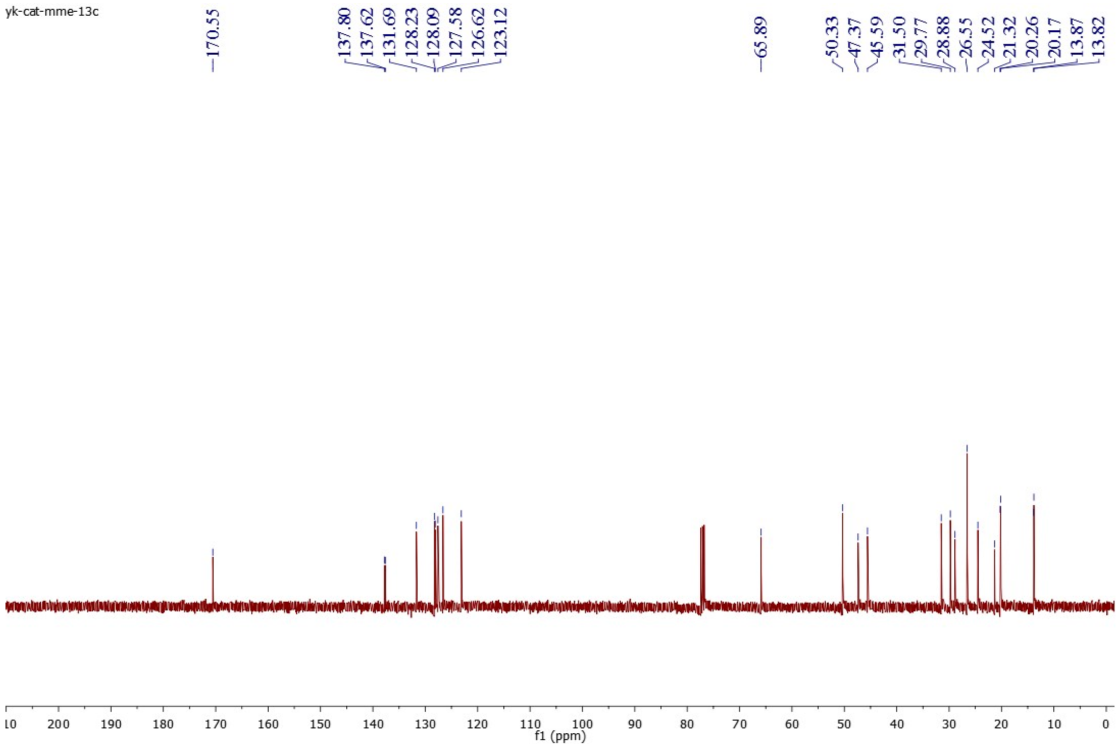
yk-cat-mme-13c
170.55
137.80
137.62
131.69
128.23
128.09
127.58
126.62
123.12
65.89
50.33
47.37
45.59
31.50
29.77
28.88
26.55
24.52
21.32
20.26
20.17
13.87
13.82
f1 (ppm)

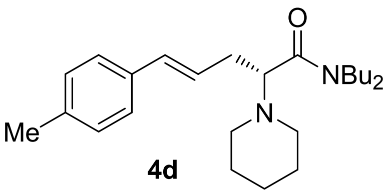
O
NBu2
N
Me

4d

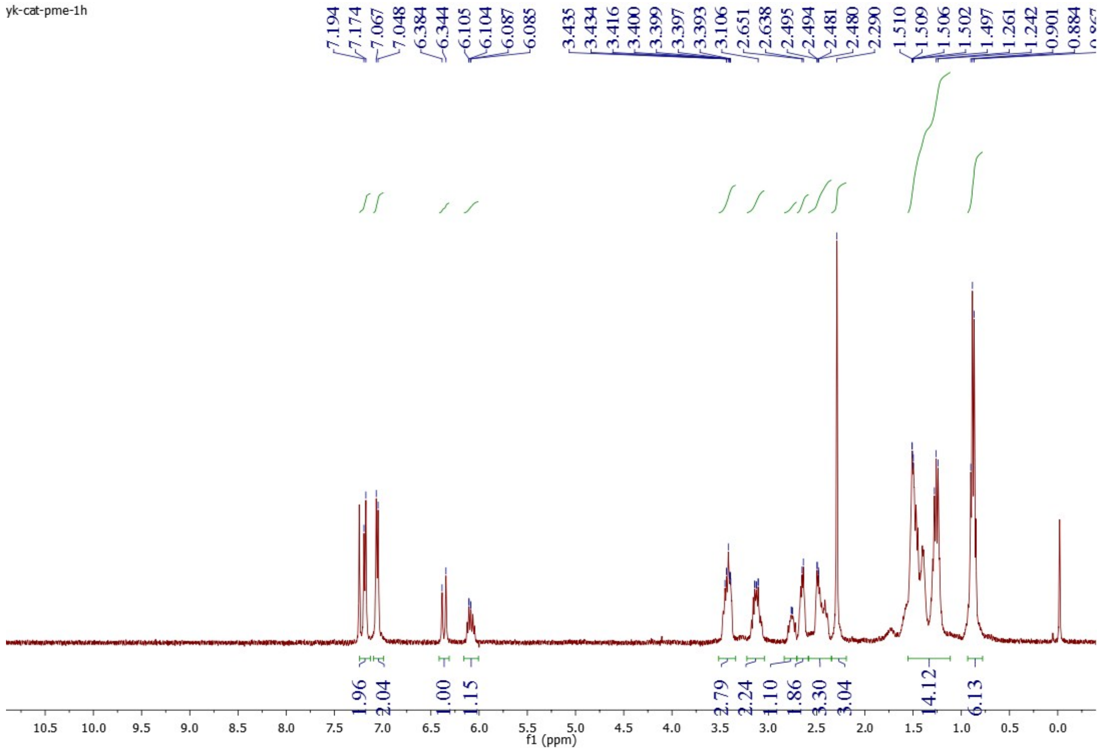
yk-cat-pme-1h
7.194
7.174
7.067
7.048
6.384
6.344
6.105
6.104
6.087
6.085
3.435
3.434
3.416
3.400
3.399
3.397
3.393
3.106
2.651
2.638
2.495
2.494
2.481
2.480
2.290
1.510
1.509
1.506
1.502
1.497
1.261
1.242
0.901
0.884
1.96
2.04
1.00
1.15
2.79
2.24
1.10
1.86
3.30
3.04
14.12
6.13
10.5 10.0 9.5 9.0 8.5 8.0 7.5 7.0 6.5 6.0 5.5 5.0 4.5 4.0 3.5 3.0 2.5 2.0 1.5 1.0 0.5 0.0
f1 (ppm)

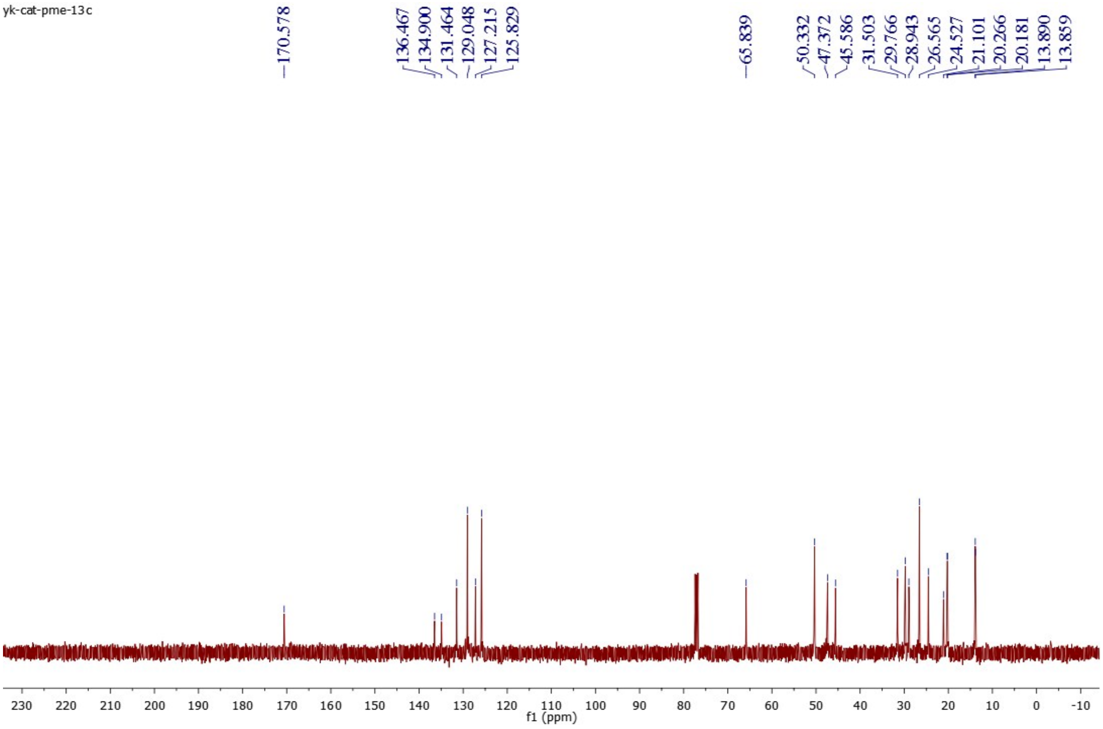
yk-cat-pme-13c
170.578
136.467
134.900
131.464
129.048
127.215
125.829
65.839
50.332
47.372
45.586
31.503
29.766
28.943
26.565
24.527
21.101
20.266
20.181
13.890
13.859
230 220 210 200 190 180 170 160 150 140 130 120 110 100 90 80 70 60 50 40 30 20 10 0 -10
f1 (ppm)

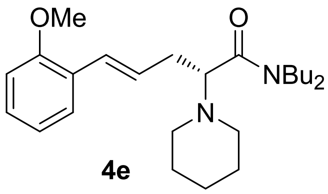
OMe
O
NBu2
N

4e

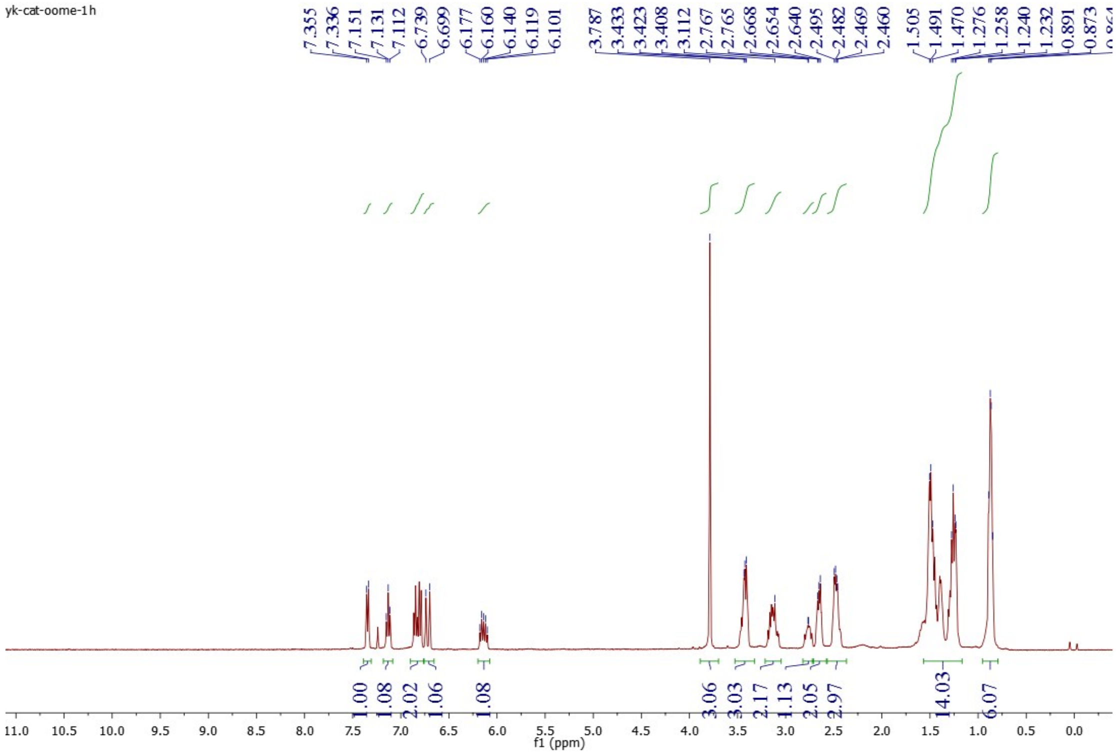
yk-cat-oome-1h
7.355
7.336
7.151
7.131
7.112
6.739
6.699
6.177
6.160
6.140
6.119
6.101
3.787
3.433
3.423
3.408
3.112
2.767
2.765
2.668
2.654
2.640
2.495
2.482
2.469
2.460
1.505
1.491
1.470
1.276
1.258
1.240
1.232
0.891
0.873
1.00
1.08
2.02
1.06
1.08
3.06
3.03
2.17
1.13
2.05
2.97
14.03
6.07
f1 (ppm)

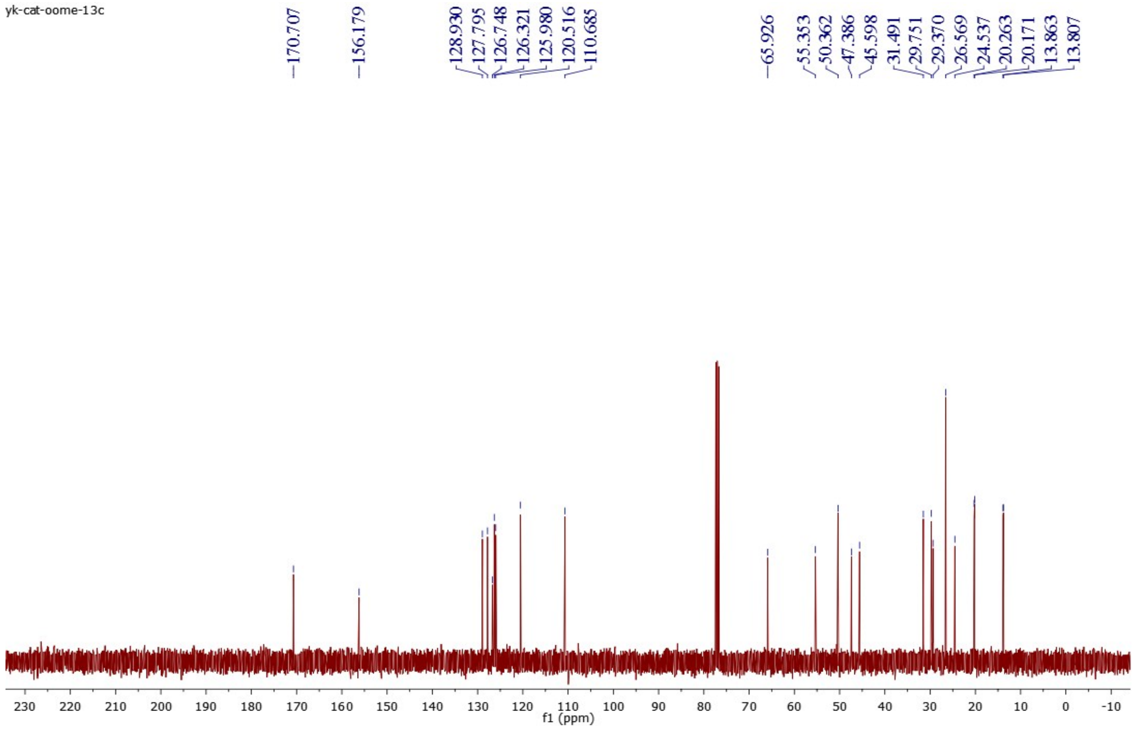
yk-cat-oome-13c
170.707
156.179
128.930
127.795
126.748
126.321
125.980
120.516
110.685
65.926
55.353
50.362
47.386
45.598
31.491
29.751
29.370
26.569
24.537
20.263
20.171
13.863
13.807
f1 (ppm)

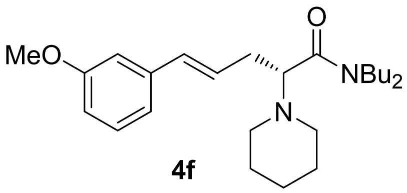
MeO
O
NBu2
N

4f

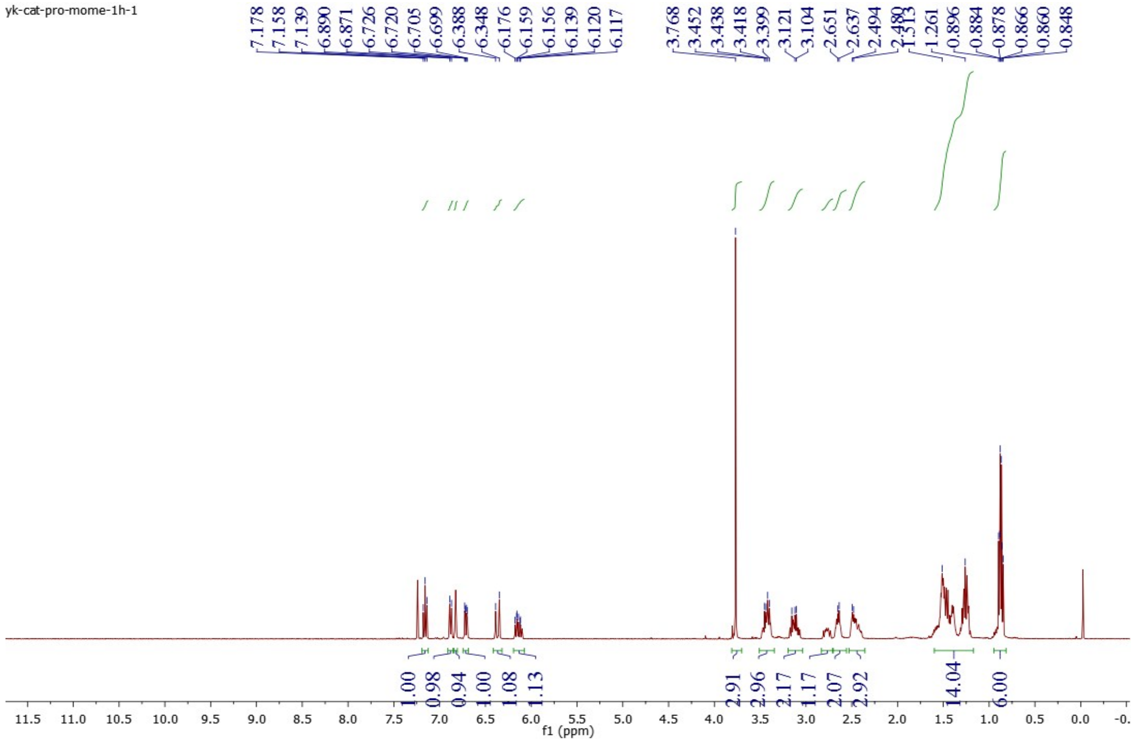
yk-cat-pro-mome-1h-1
7.178
7.158
7.139
6.890
6.871
6.726
6.720
6.705
6.699
6.388
6.348
6.176
6.159
6.156
6.139
6.120
6.117
3.768
3.452
3.438
3.418
3.399
3.121
3.104
2.651
2.637
2.494
2.480
1.513
1.261
0.896
0.884
0.878
0.866
0.860
0.848
1.00
0.98
0.94
1.00
1.08
1.13
2.91
2.96
2.17
1.17
2.07
2.92
14.04
6.00
f1 (ppm)

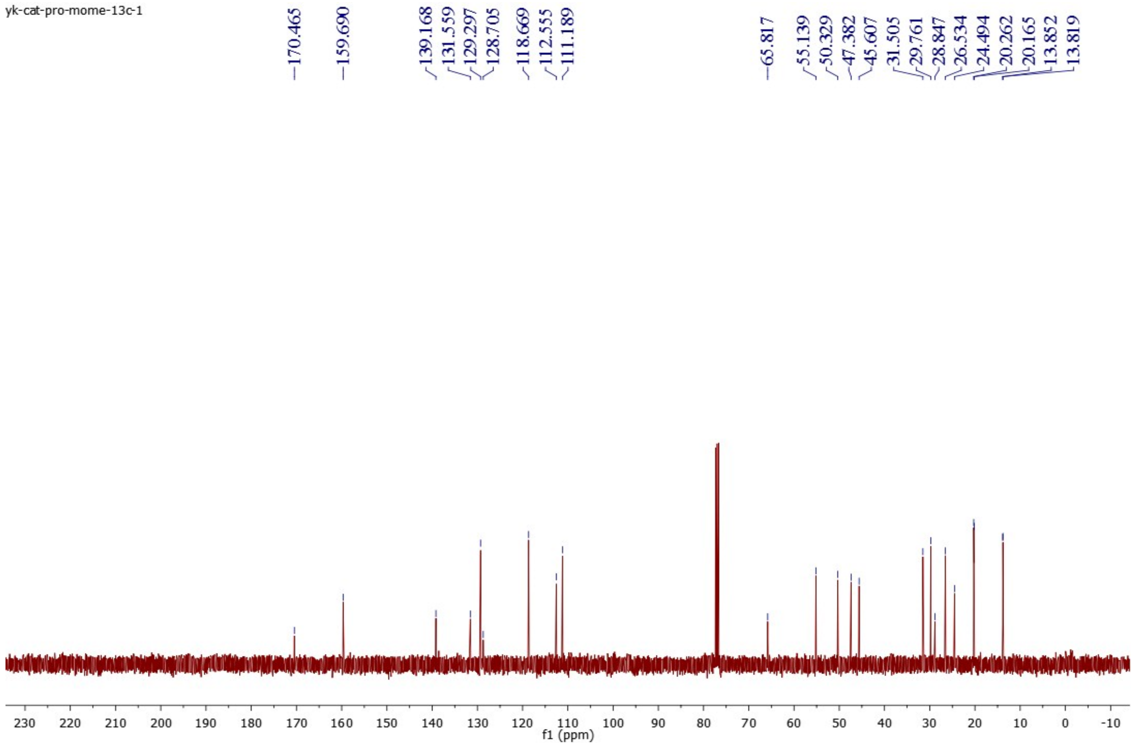
yk-cat-pro-mome-13c-1
170.465
159.690
139.168
131.559
129.297
128.705
118.669
112.555
111.189
65.817
55.139
50.329
47.382
45.607
31.505
29.761
28.847
26.534
24.494
20.262
20.165
13.852
13.819
f1 (ppm)

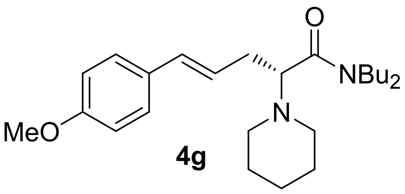

O
NBu2
MeO
N

4g

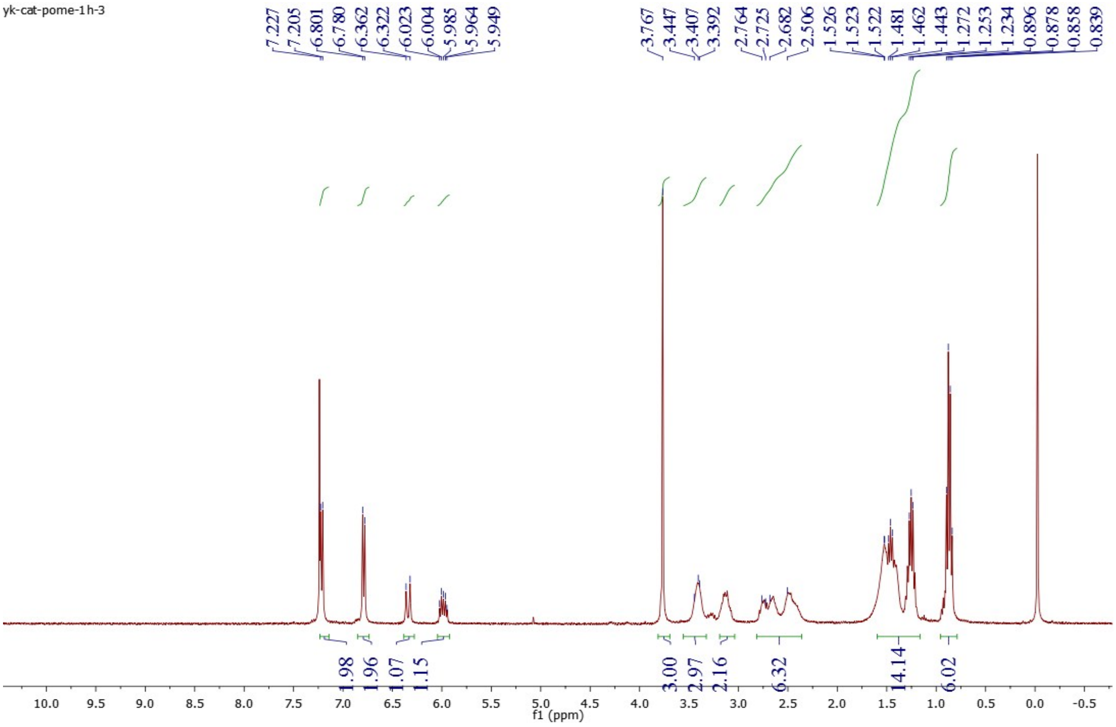

yk-cat-pome-1h-3
7.227
7.205
6.801
6.780
6.362
6.322
6.023
6.004
5.985
5.964
5.949
3.767
3.447
3.407
3.392
2.764
2.725
2.682
2.506
1.526
1.523
1.522
1.481
1.462
1.443
1.272
1.253
1.234
0.896
0.878
0.858
0.839
1.98
1.96
1.07
1.15
3.00
2.97
2.16
6.32
14.14
6.02
f1 (ppm)

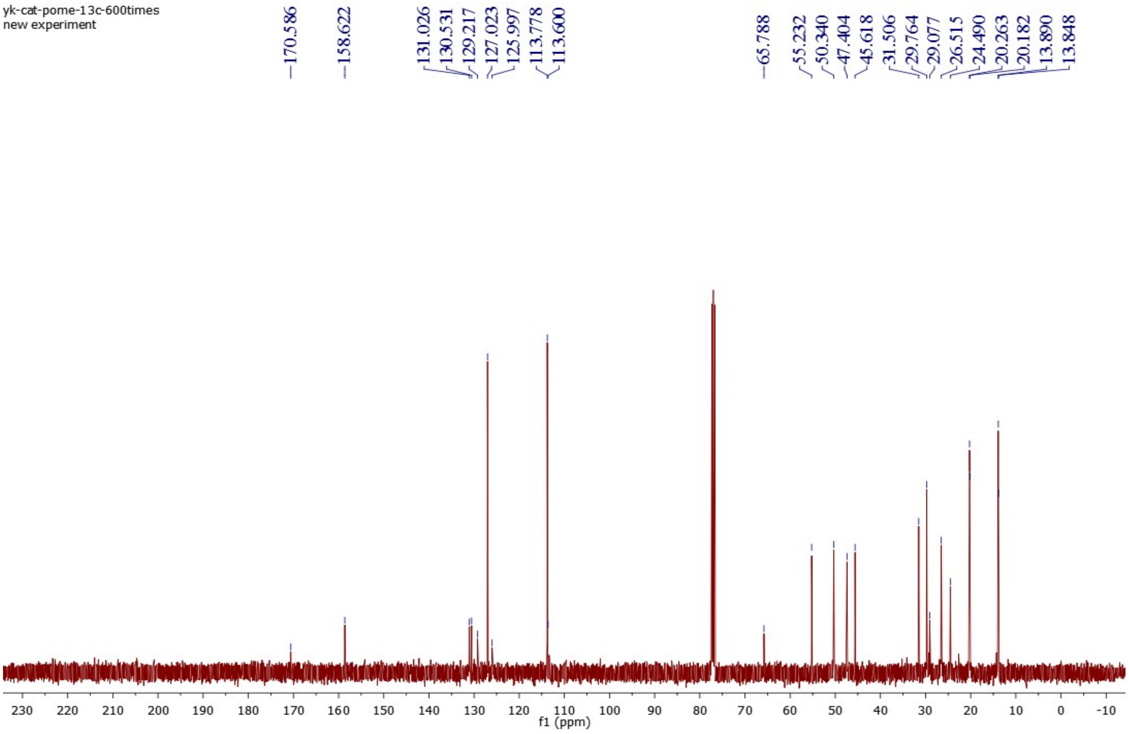

yk-cat-pome-13c-600times
new experiment
170.586
158.622
131.026
130.531
129.217
127.023
125.997
113.778
113.600
65.788
55.232
50.340
47.404
45.618
31.506
29.764
29.077
26.515
24.490
20.263
20.182
13.890
13.848
f1 (ppm)

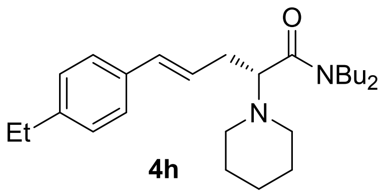
O
NBu2
N
Et

4h

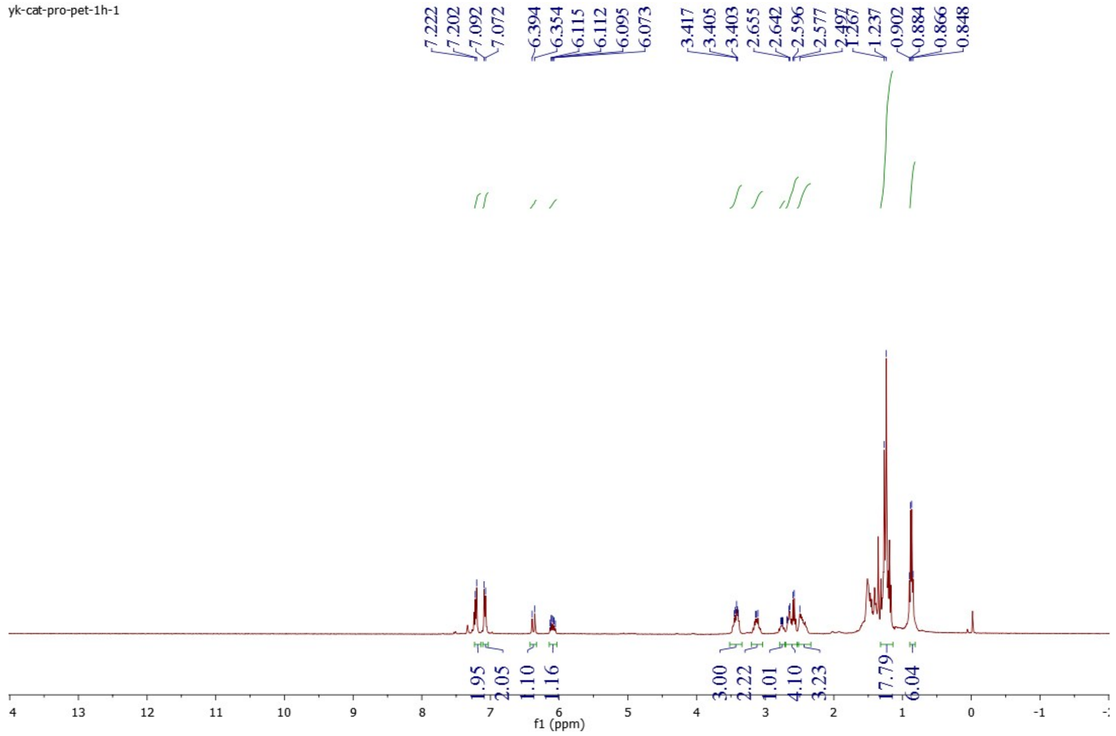
yk-cat-pro-pet-1h-1
7.222
7.202
7.092
7.072
6.394
6.354
6.115
6.112
6.095
6.073
3.417
3.405
3.403
2.655
2.642
2.596
2.577
2.497
1.267
1.237
0.902
0.884
0.866
0.848
1.95
2.05
1.10
1.16
3.00
2.22
1.01
4.10
3.23
17.79
6.04
f1 (ppm)

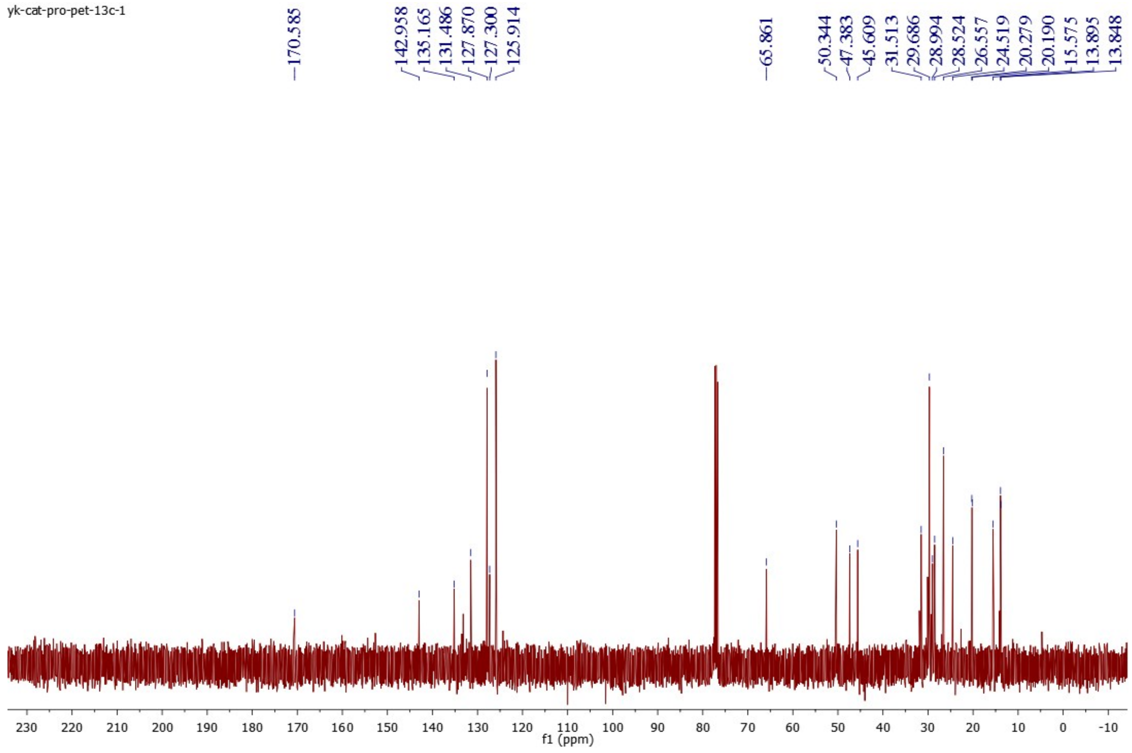
yk-cat-pro-pet-13c-1
170.585
142.958
135.165
131.486
127.870
127.300
125.914
65.861
50.344
47.383
45.609
31.513
29.686
28.994
28.524
26.557
24.519
20.279
20.190
15.575
13.895
13.848
f1 (ppm)

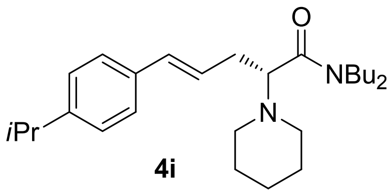

4i

yk-cat-pro-pipr-1h-2

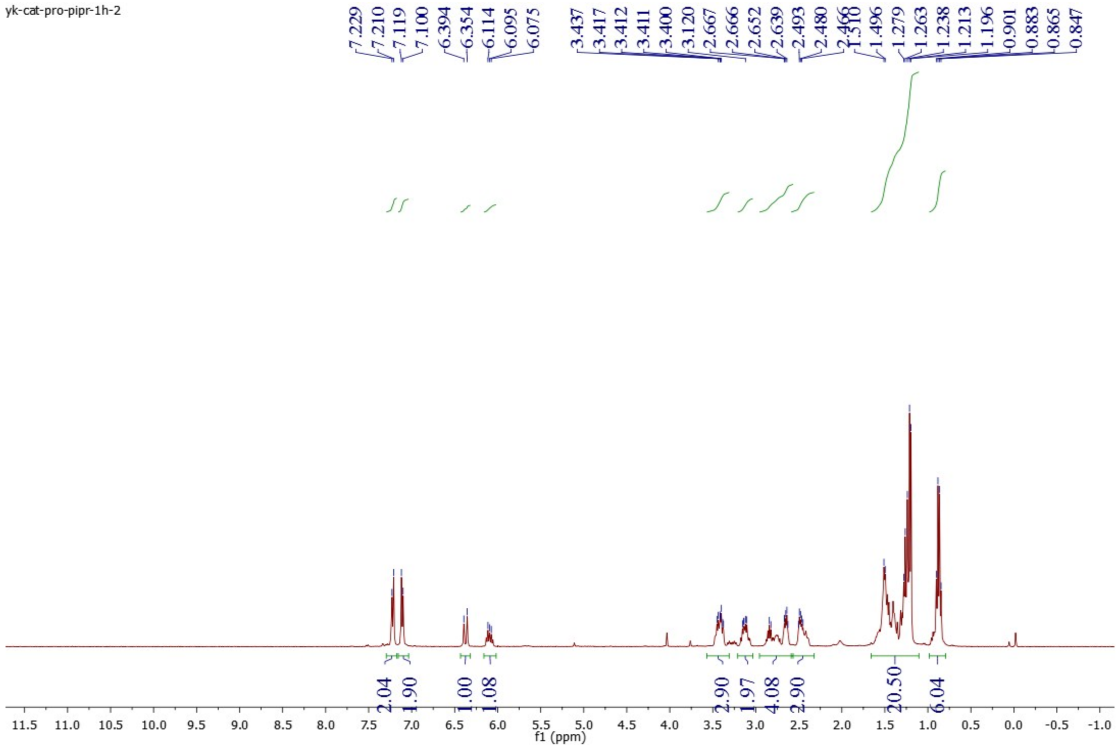

yk-cat-pro-pipr-13c-2

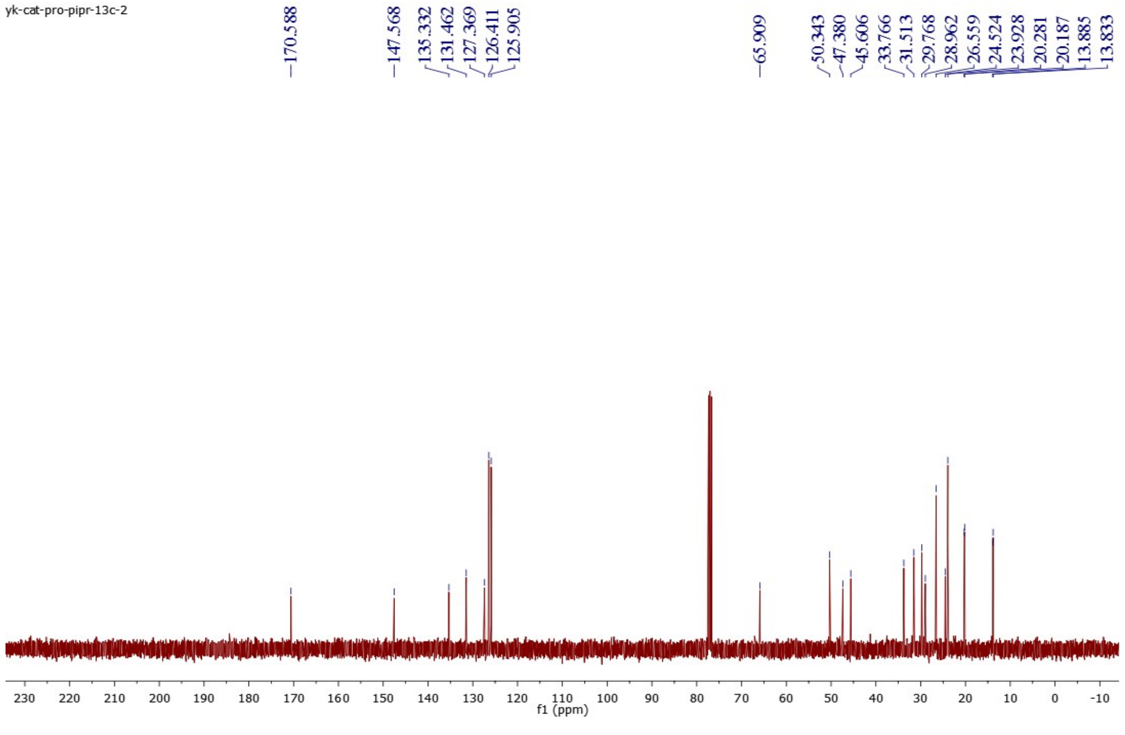

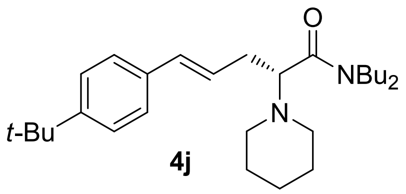
O
NBu2
N
t-Bu

4j

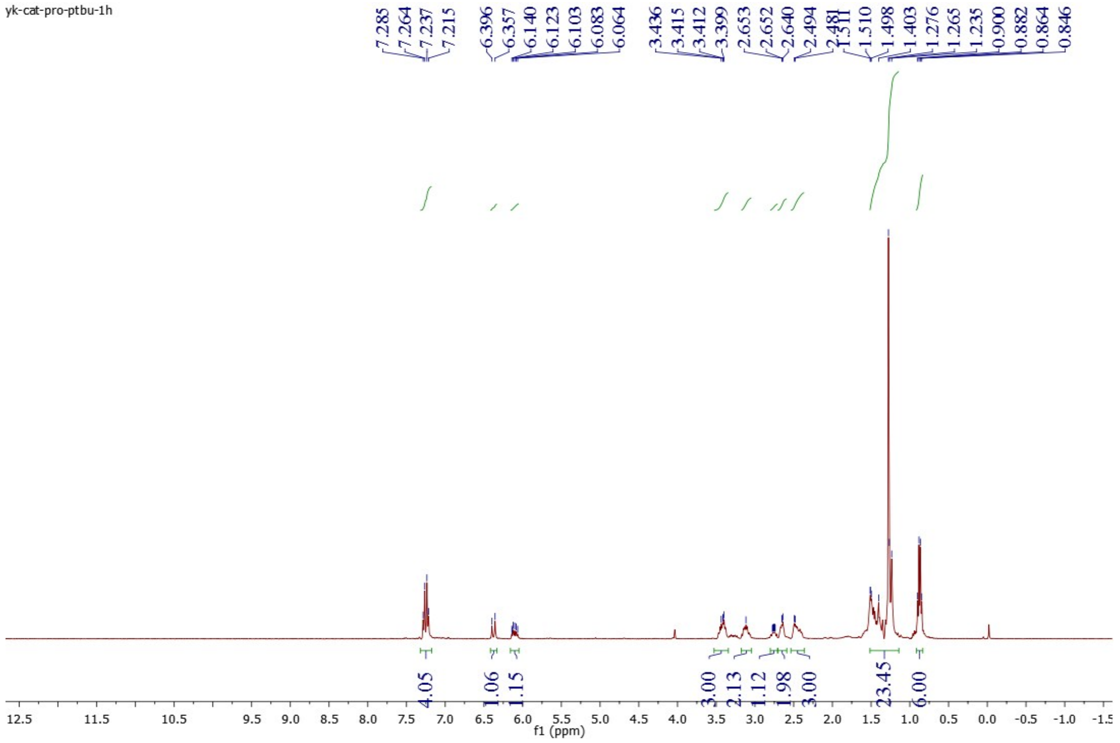
yk-cat-pro-ptbu-1h
7.285
7.264
7.237
7.215
6.396
6.357
6.140
6.123
6.103
6.083
6.064
3.436
3.415
3.412
3.399
2.653
2.652
2.640
2.494
2.481
1.511
1.510
1.498
1.403
1.276
1.265
1.235
0.900
0.882
0.864
0.846
4.05
1.06
1.15
3.00
2.13
1.12
1.98
3.00
23.45
6.00
f1 (ppm)

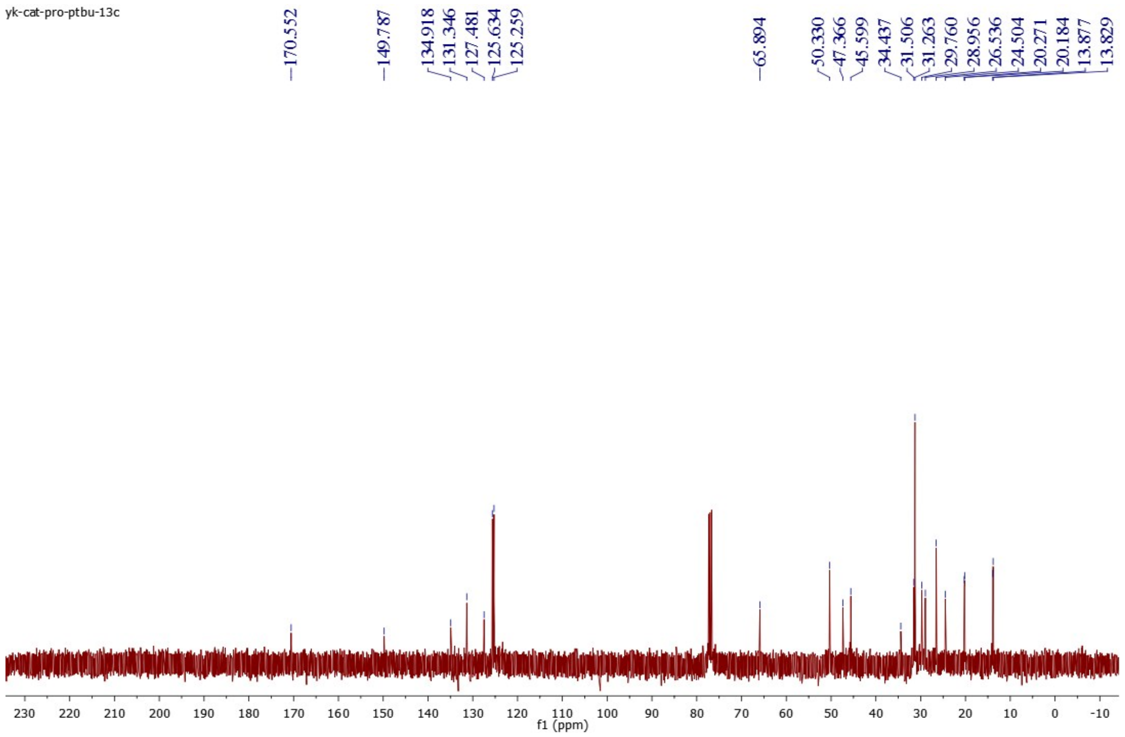
yk-cat-pro-ptbu-13c
170.552
149.787
134.918
131.346
127.481
125.634
125.259
65.894
50.330
47.366
45.599
34.437
31.506
31.263
29.760
28.956
26.536
24.504
20.271
20.184
13.877
13.829
f1 (ppm)

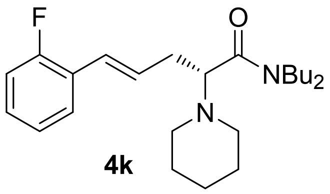
F
O
NBu2
N

4k

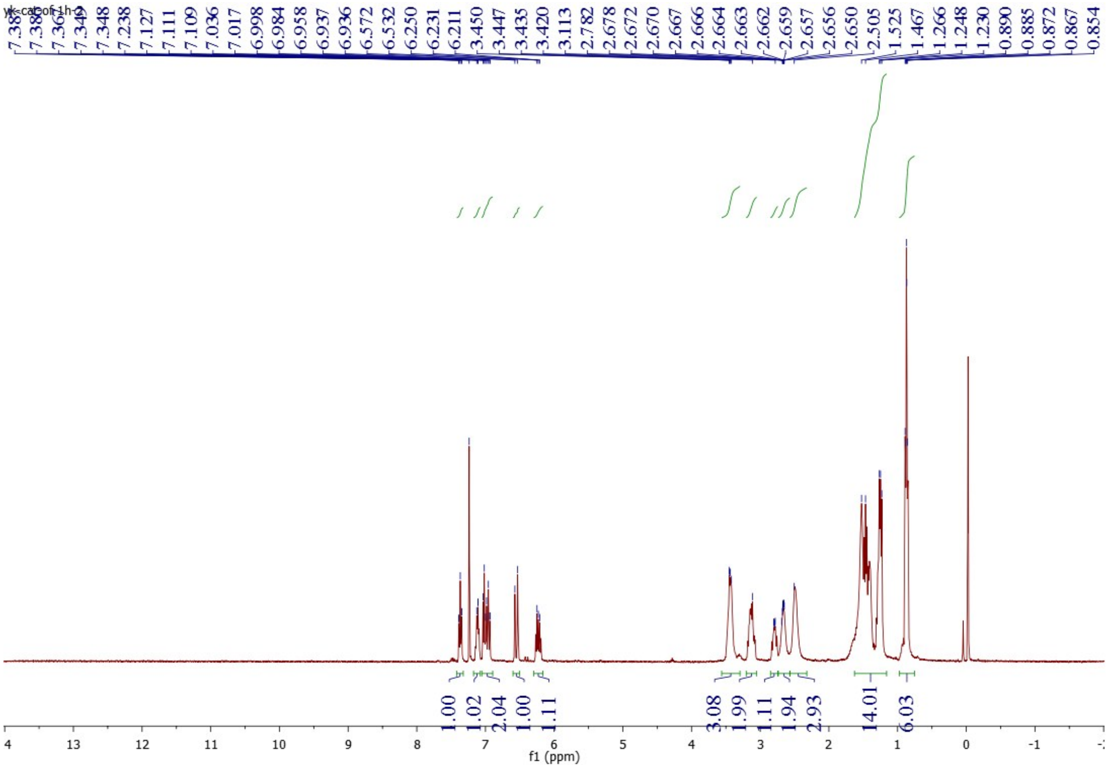
yk-cat-of-1h-2
7.348
7.238
7.127
7.111
7.109
7.036
7.017
6.998
6.984
6.958
6.937
6.936
6.572
6.532
6.250
6.231
6.211
3.450
3.447
3.435
3.420
3.113
2.782
2.678
2.672
2.670
2.667
2.666
2.664
2.663
2.662
2.659
2.657
2.656
2.650
2.505
1.525
1.467
1.266
1.248
1.230
0.890
0.885
0.872
0.867
0.854
1.00
1.02
2.04
1.00
1.11
3.08
1.99
1.11
1.94
2.93
14.01
6.03
f1 (ppm)

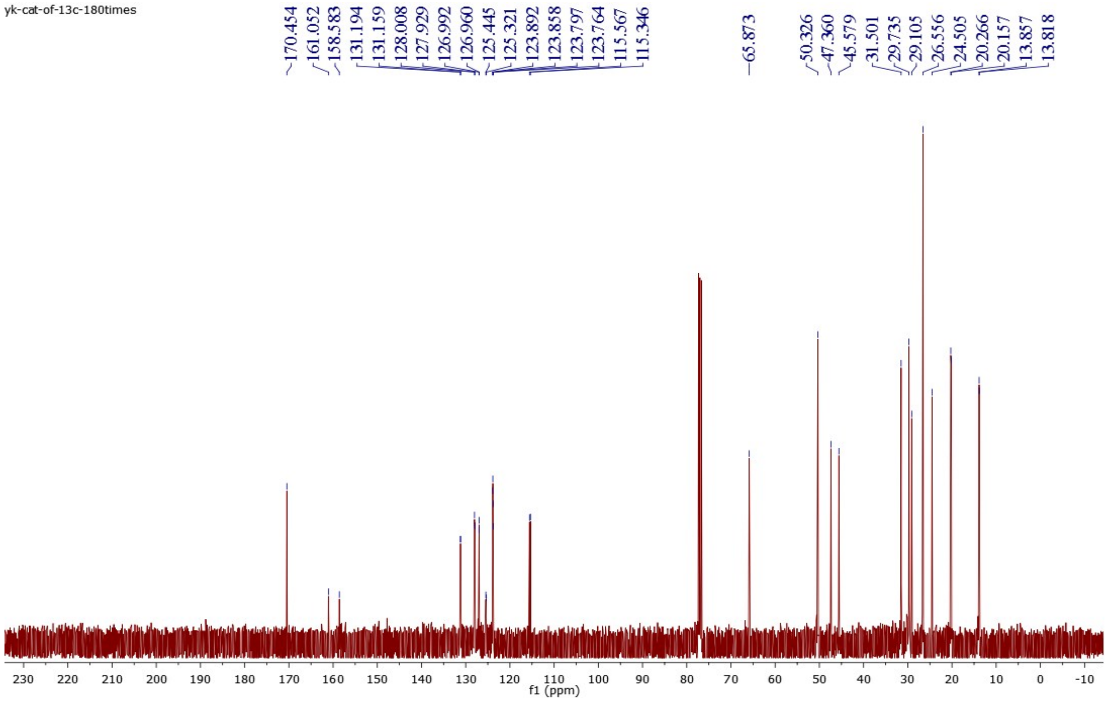
yk-cat-of-13c-180times
170.454
161.052
158.583
131.194
131.159
128.008
127.929
126.992
126.960
125.445
125.321
123.892
123.858
123.797
123.764
115.567
115.346
65.873
50.326
47.360
45.579
31.501
29.735
29.105
26.556
24.505
20.266
20.157
13.857
13.818
f1 (ppm)

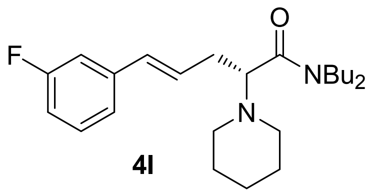
F
O
NBu2
N

4l

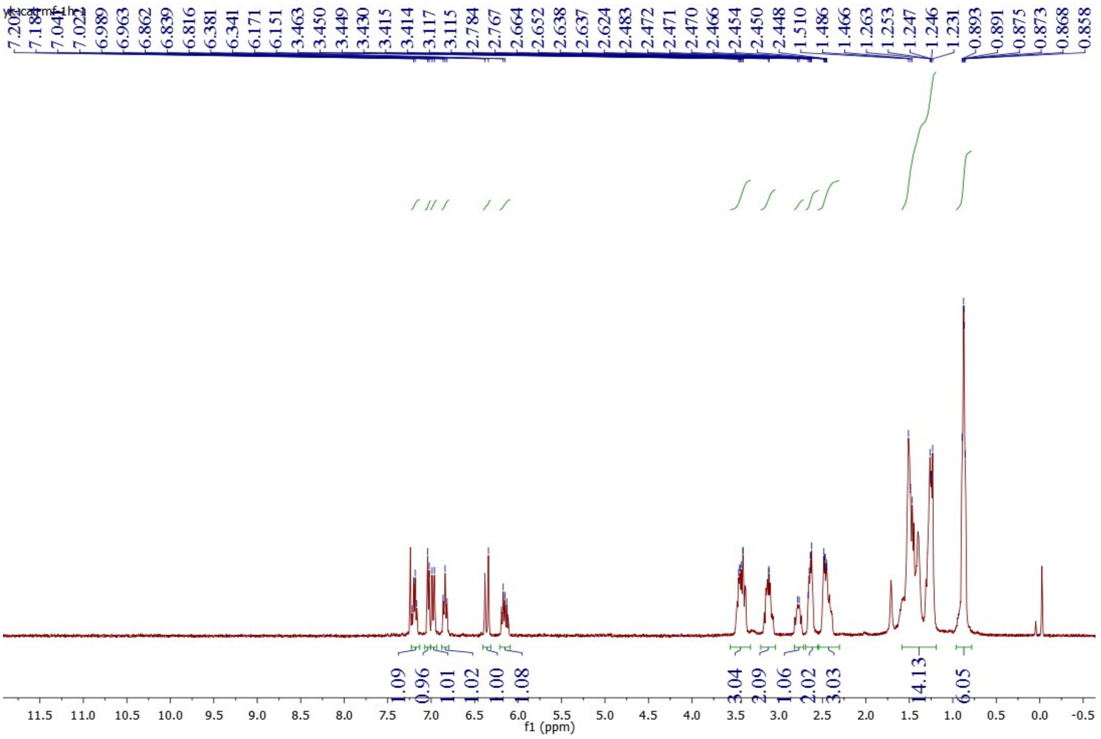
yk-cat-mf-1h-1
7.201
7.181
7.041
7.022
6.989
6.963
6.862
6.839
6.816
6.381
6.341
6.171
6.151
3.463
3.450
3.449
3.430
3.415
3.414
3.117
3.115
2.784
2.767
2.664
2.652
2.638
2.637
2.624
2.483
2.472
2.471
2.470
2.466
2.454
2.450
2.448
1.510
1.486
1.466
1.263
1.253
1.247
1.246
1.231
0.893
0.891
0.875
0.873
0.868
0.858
1.09
0.96
1.01
1.02
1.00
1.08
3.04
2.09
1.06
2.02
3.03
14.13
6.05
f1 (ppm)

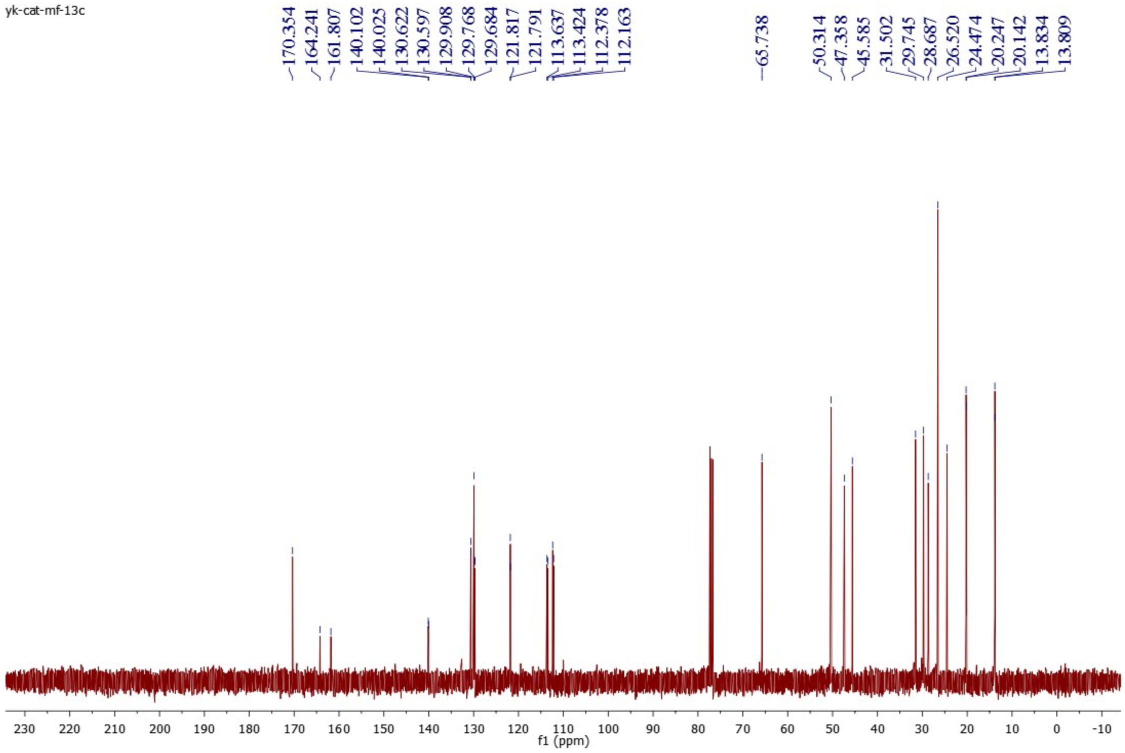
yk-cat-mf-13c
170.354
164.241
161.807
140.102
140.025
130.622
130.597
129.908
129.768
129.684
121.817
121.791
113.637
113.424
112.378
112.163
65.738
50.314
47.358
45.585
31.502
29.745
28.687
26.520
24.474
20.247
20.142
13.834
13.809
f1 (ppm)

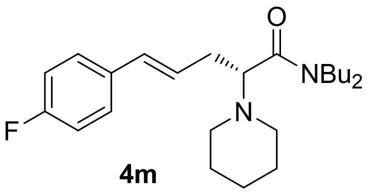

F
O
NBu2
N

4m

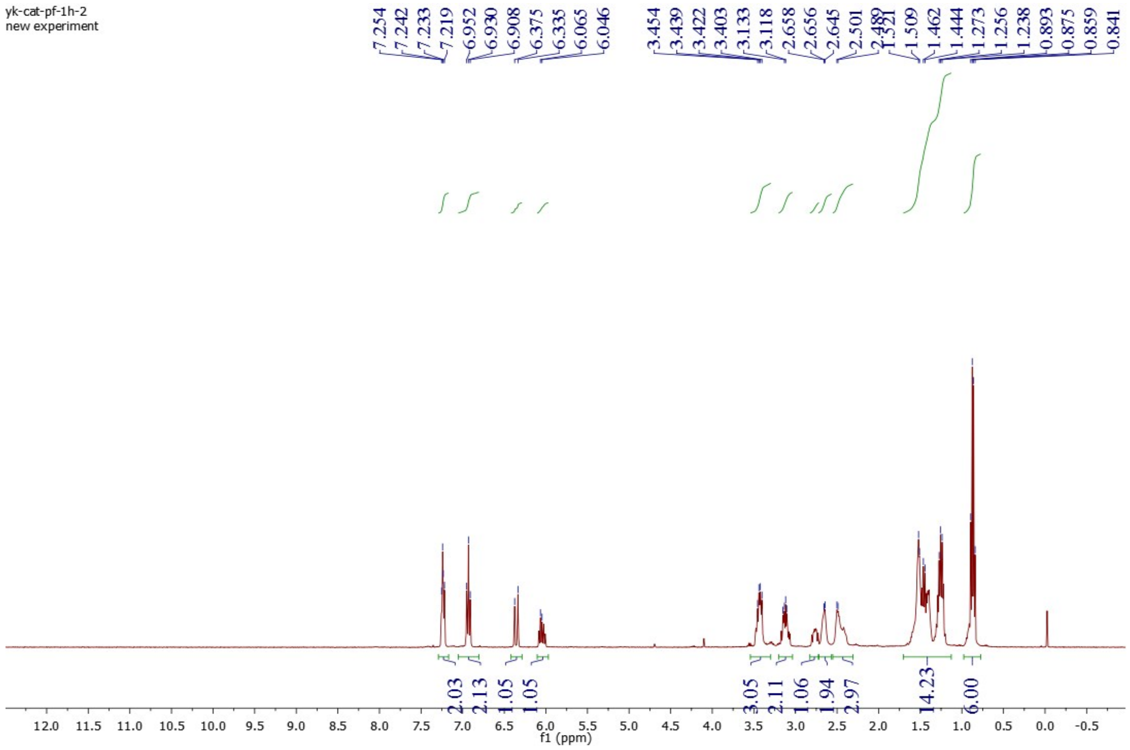

yk-cat-pf-1h-2
new experiment
7.254
7.242
7.233
7.219
6.952
6.930
6.908
6.375
6.335
6.065
6.046
3.454
3.439
3.422
3.403
3.133
3.118
2.658
2.656
2.645
2.501
2.489
1.521
1.509
1.462
1.444
1.273
1.256
1.238
0.893
0.875
0.859
0.841
2.03
2.13
1.05
1.05
3.05
2.11
1.06
1.94
2.97
14.23
6.00
12.0 11.5 11.0 10.5 10.0 9.5 9.0 8.5 8.0 7.5 7.0 6.5 6.0 5.5 5.0 4.5 4.0 3.5 3.0 2.5 2.0 1.5 1.0 0.5 0.0 -0.5
f1 (ppm)

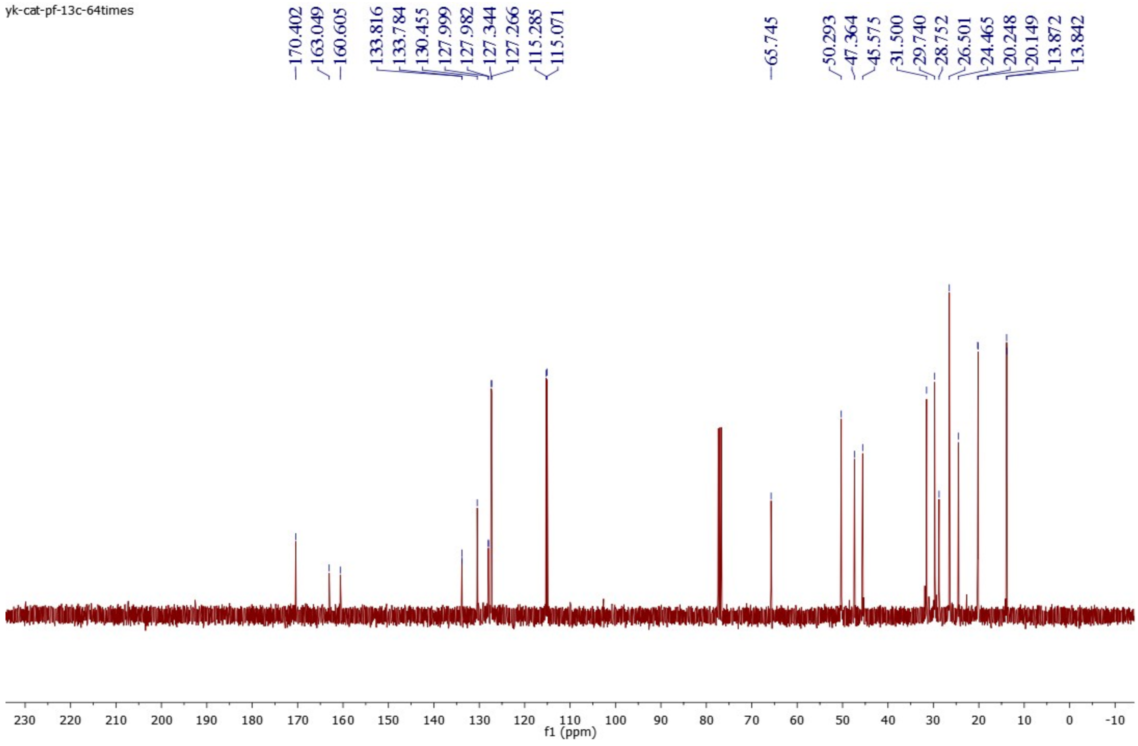

yk-cat-pf-13c-64times
170.402
163.049
160.605
133.816
133.784
130.455
127.999
127.982
127.344
127.266
115.285
115.071
65.745
50.293
47.364
45.575
31.500
29.740
28.752
26.501
24.465
20.248
20.149
13.872
13.842
230 220 210 200 190 180 170 160 150 140 130 120 110 100 90 80 70 60 50 40 30 20 10 0 -10
f1 (ppm)

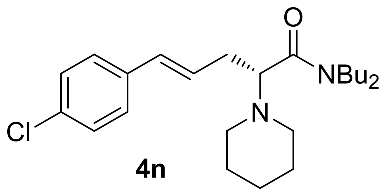

O
NBu2
N
Cl

4n

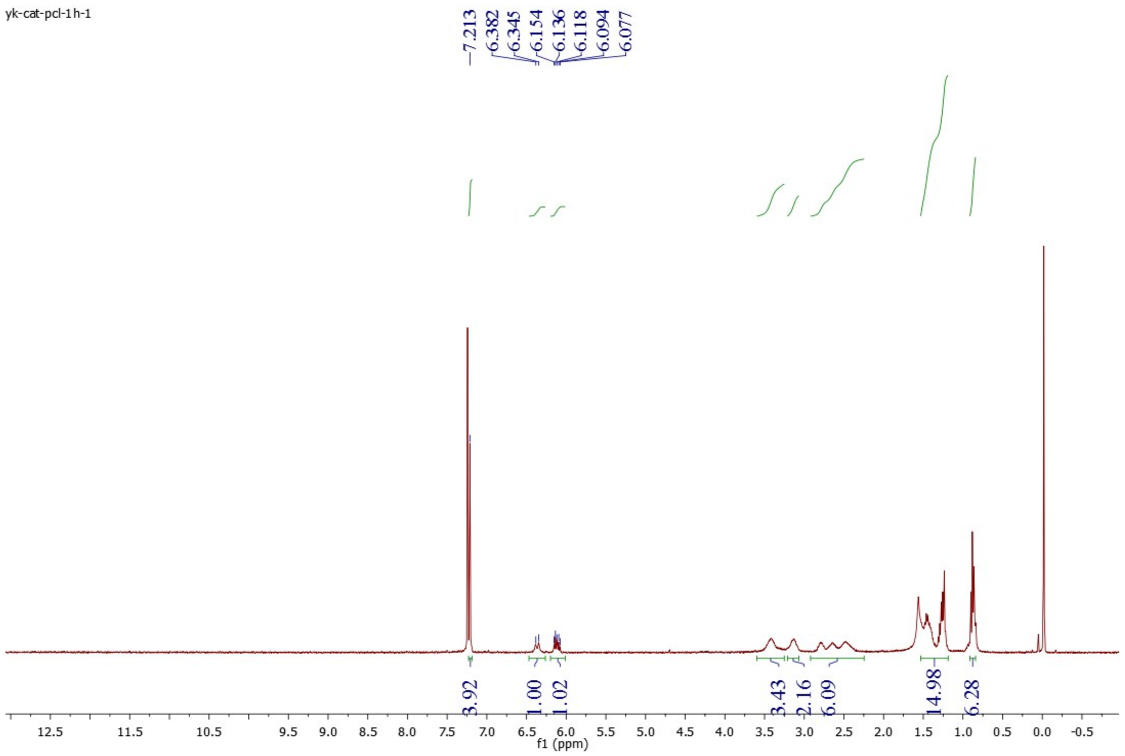

yk-cat-pcl-1h-1
7.213
6.382
6.345
6.154
6.136
6.118
6.094
6.077
3.92
1.00
1.02
3.43
2.16
6.09
14.98
6.28
12.5 11.5 10.5 9.5 9.0 8.5 8.0 7.5 7.0 6.5 6.0 5.5 5.0 4.5 4.0 3.5 3.0 2.5 2.0 1.5 1.0 0.5 0.0 -0.5
f1 (ppm)

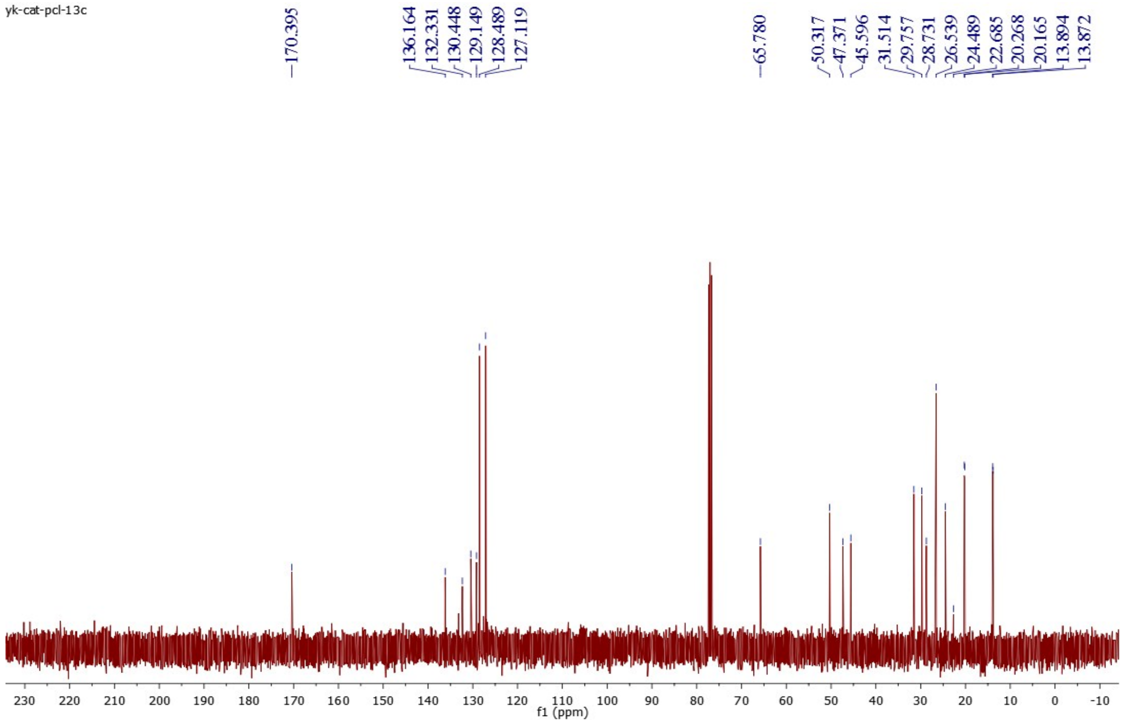

yk-cat-pcl-13c
170.395
136.164
132.331
130.448
129.149
128.489
127.119
65.780
50.317
47.371
45.596
31.514
29.757
28.731
26.539
24.489
22.685
20.268
20.165
13.894
13.872
230 220 210 200 190 180 170 160 150 140 130 120 110 100 90 80 70 60 50 40 30 20 10 0 -10
f1 (ppm)

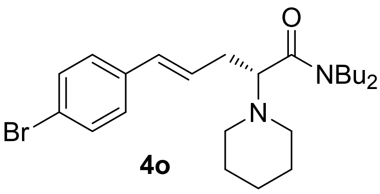
Br
O
NBu2
N

4o

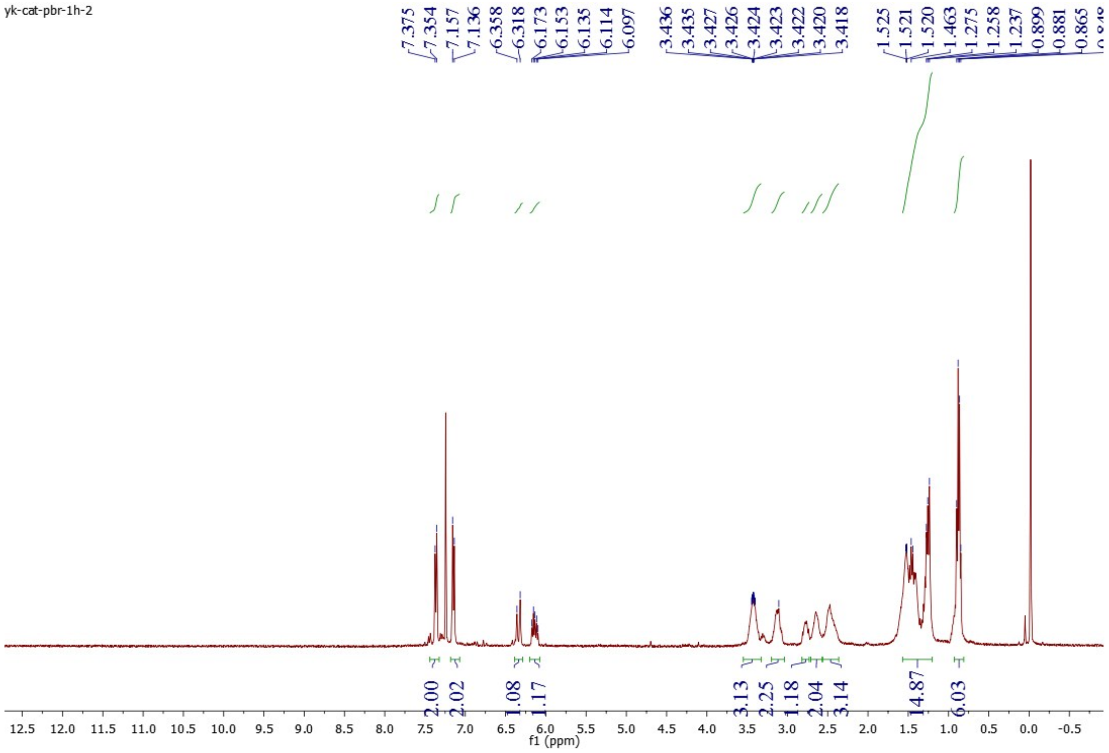
yk-cat-pbr-1h-2
7.375
7.354
7.157
7.136
6.358
6.318
6.173
6.153
6.135
6.114
6.097
3.436
3.435
3.427
3.426
3.424
3.423
3.422
3.420
3.418
1.525
1.521
1.520
1.463
1.275
1.258
1.237
0.899
0.881
0.865
2.00
2.02
1.08
1.17
3.13
2.25
1.18
2.04
3.14
14.87
6.03
12.5 12.0 11.5 11.0 10.5 10.0 9.5 9.0 8.5 8.0 7.5 7.0 6.5 6.0 5.5 5.0 4.5 4.0 3.5 3.0 2.5 2.0 1.5 1.0 0.5 0.0 -0.5
f1 (ppm)

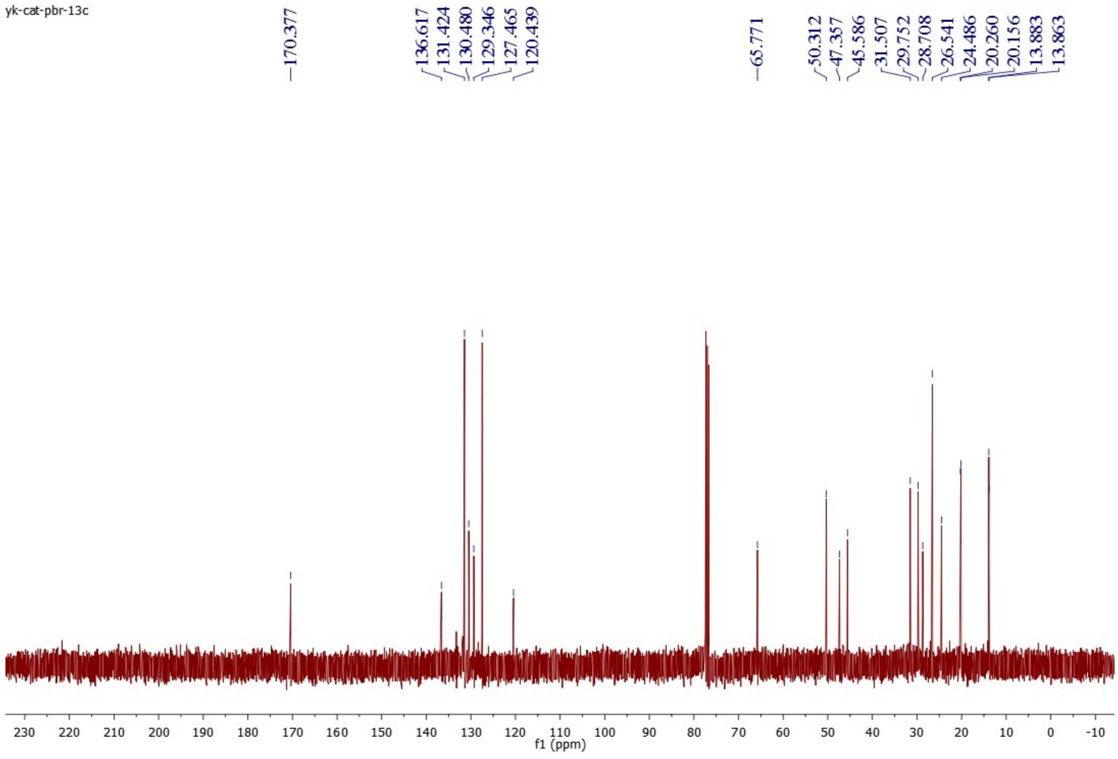
yk-cat-pbr-13c
170.377
136.617
131.424
130.480
129.346
127.465
120.439
65.771
50.312
47.357
45.586
31.507
29.752
28.708
26.541
24.486
20.260
20.156
13.883
13.863
230 220 210 200 190 180 170 160 150 140 130 120 110 100 90 80 70 60 50 40 30 20 10 0 -10
f1 (ppm)

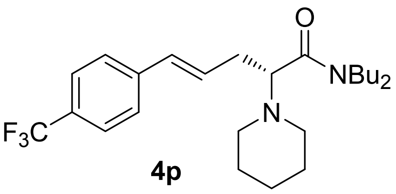
O
NBu2
N
F3C

4p

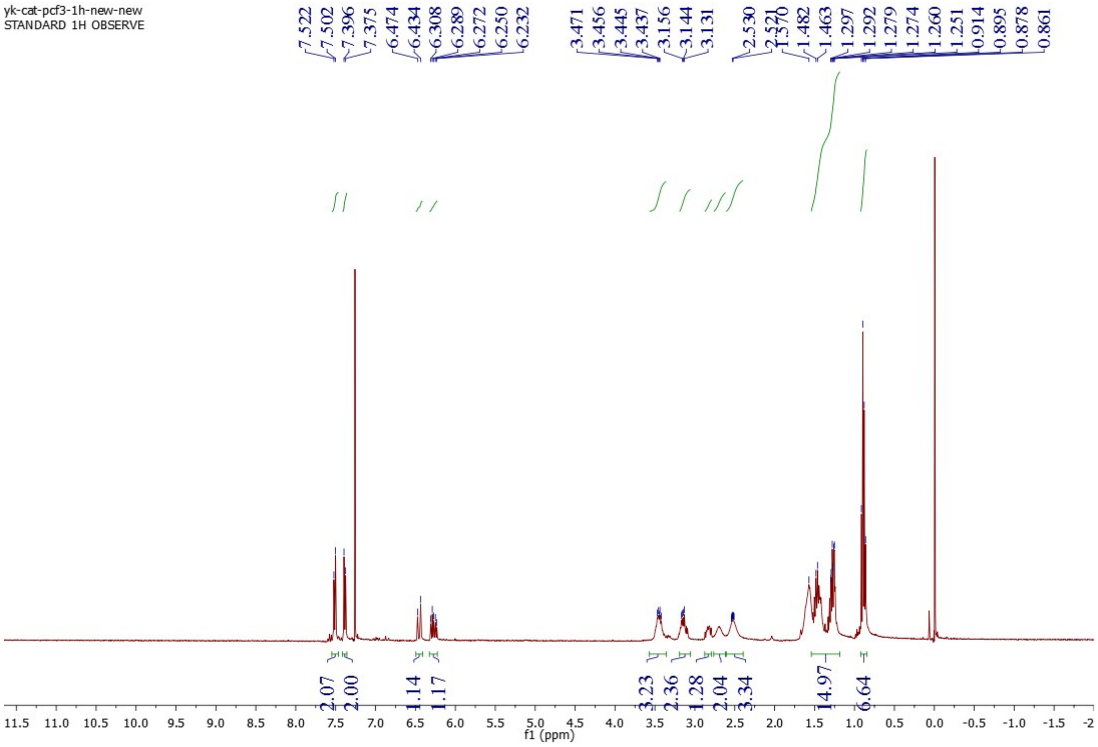
yk-cat-pcf3-1h-new-new
STANDARD 1H OBSERVE
7.522
7.502
7.396
7.375
6.474
6.434
6.308
6.289
6.272
6.250
6.232
3.471
3.456
3.445
3.437
3.156
3.144
3.131
2.530
2.521
1.570
1.482
1.463
1.297
1.292
1.279
1.274
1.260
1.251
0.914
0.895
0.878
0.861
2.07
2.00
1.14
1.17
3.23
2.36
1.28
2.04
3.34
14.97
6.64
11.5 11.0 10.5 10.0 9.5 9.0 8.5 8.0 7.5 7.0 6.5 6.0 5.5 5.0 4.5 4.0 3.5 3.0 2.5 2.0 1.5 1.0 0.5 0.0 -0.5 -1.0 -1.5 -2
f1 (ppm)

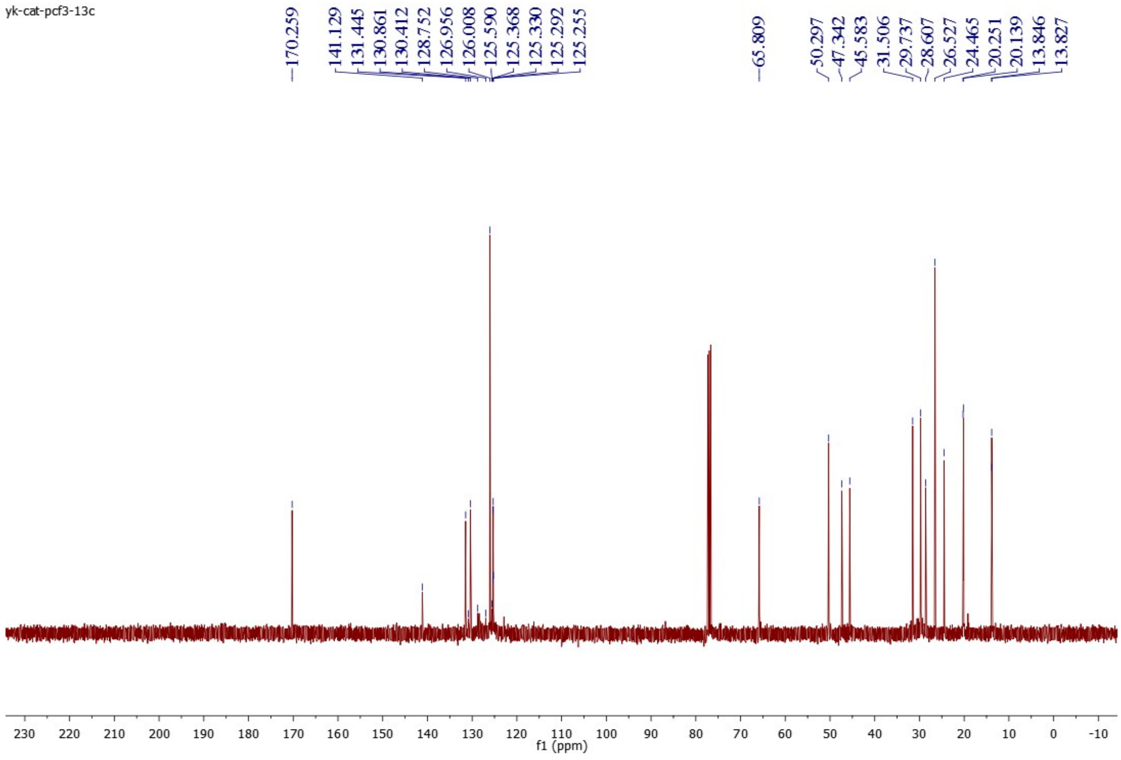
yk-cat-pcf3-13c
170.259
141.129
131.445
130.861
130.412
128.752
126.956
126.008
125.590
125.368
125.330
125.292
125.255
65.809
50.297
47.342
45.583
31.506
29.737
28.607
26.527
24.465
20.251
20.139
13.846
13.827
230 220 210 200 190 180 170 160 150 140 130 120 110 100 90 80 70 60 50 40 30 20 10 0 -10
f1 (ppm)

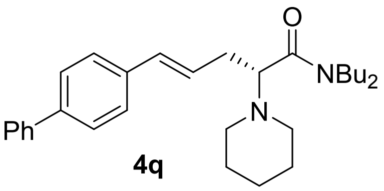

O
NBu2
N
Ph

4q

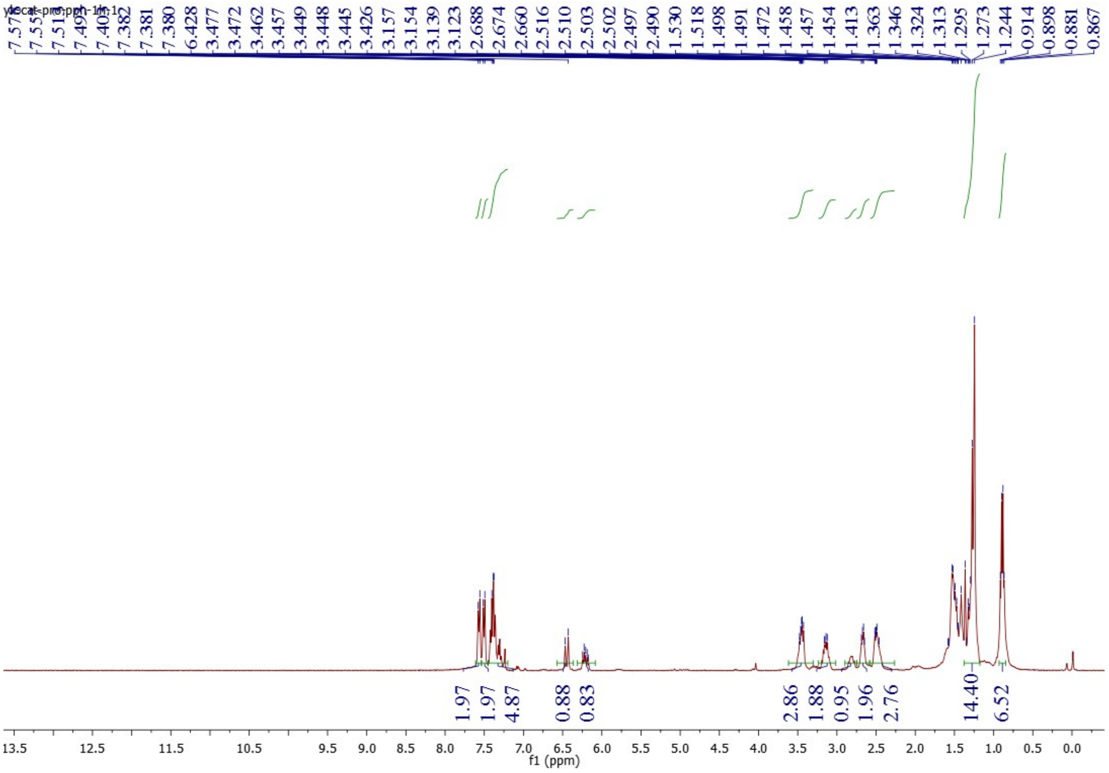

yk-cat-pro-pph-1h-1
7.495 7.403 7.382 7.381 7.380 6.428 3.477 3.472 3.462 3.457 3.449 3.448 3.445 3.426 3.157 3.154 3.139 3.123 2.688 2.674 2.660 2.516 2.510 2.503 2.502 2.497 2.490 1.530 1.518 1.498 1.491 1.472 1.458 1.457 1.454 1.413 1.363 1.346 1.324 1.313 1.295 1.273 1.244 0.914 0.898 0.881 0.867
1.97 1.97 4.87 0.88 0.83 2.86 1.88 0.95 1.96 2.76 14.40 6.52
13.5 12.5 11.5 10.5 9.5 9.0 8.5 8.0 7.5 7.0 6.5 6.0 5.5 5.0 4.5 4.0 3.5 3.0 2.5 2.0 1.5 1.0 0.5 0.0
f1 (ppm)

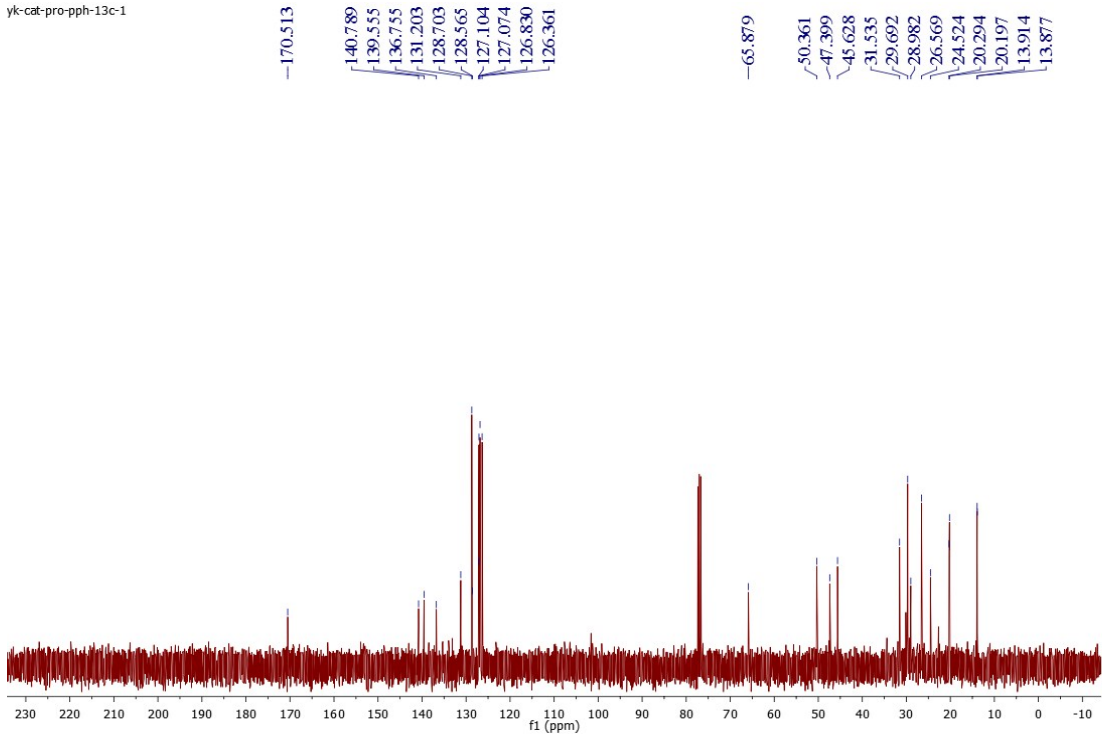

yk-cat-pro-pph-13c-1
170.513
140.789 139.555 136.755 131.203 128.703 128.565 127.104 127.074 126.830 126.361
65.879
50.361 47.399 45.628 31.535 29.692 28.982 26.569 24.524 20.294 20.197 13.914 13.877
230 220 210 200 190 180 170 160 150 140 130 120 110 100 90 80 70 60 50 40 30 20 10 0 -10
f1 (ppm)

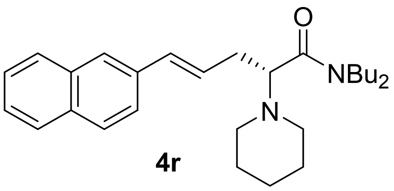
O
NBu2

4r

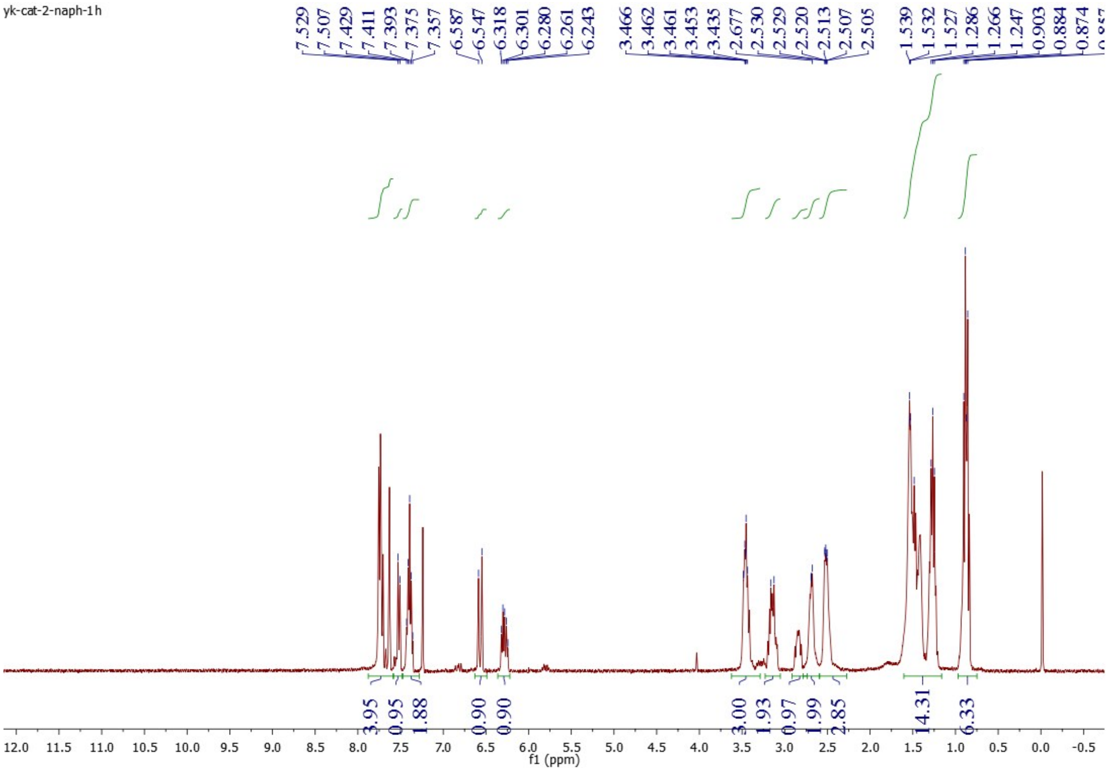
yk-cat-2-naph-1h
7.529
7.507
7.429
7.411
7.393
7.375
7.357
6.587
6.547
6.318
6.301
6.280
6.261
6.243
3.466
3.462
3.461
3.453
3.435
2.677
2.530
2.529
2.520
2.513
2.507
2.505
1.539
1.532
1.527
1.286
1.266
1.247
0.903
0.884
0.874
3.95
0.95
1.88
0.90
0.90
3.00
1.93
0.97
1.99
2.85
14.31
6.33
f1 (ppm)

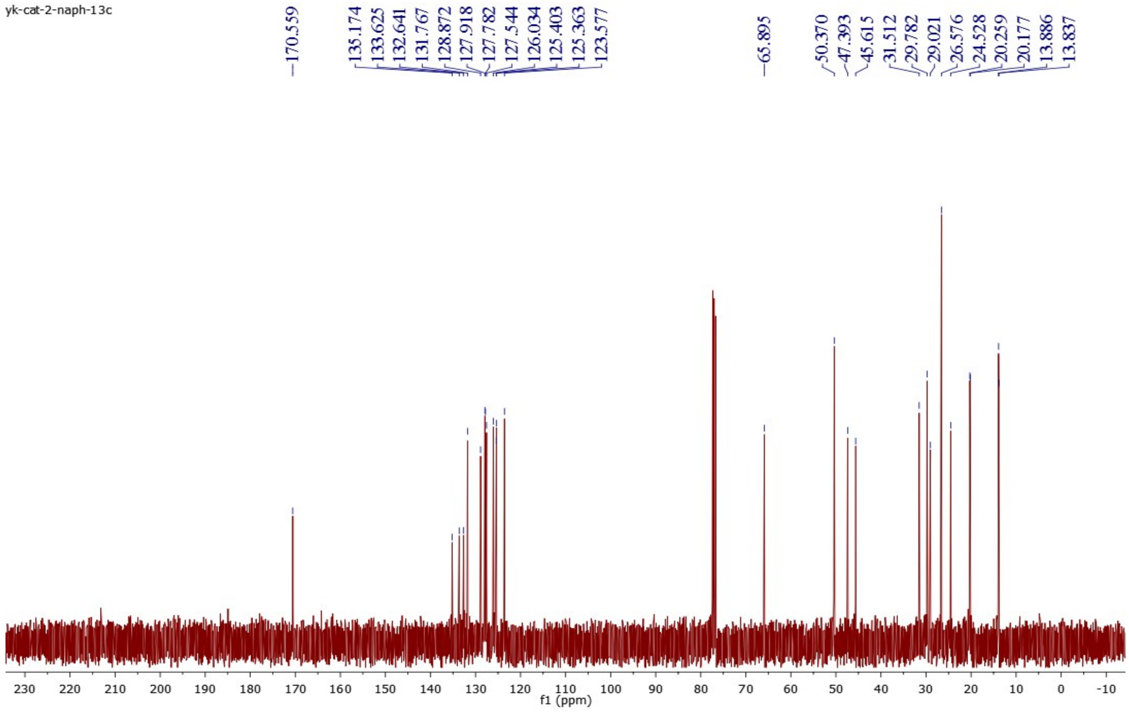
yk-cat-2-naph-13c
170.559
135.174
133.625
132.641
131.767
128.872
127.918
127.782
127.544
126.034
125.403
125.363
123.577
65.895
50.370
47.393
45.615
31.512
29.782
29.021
26.576
24.528
20.259
20.177
13.886
13.837
f1 (ppm)

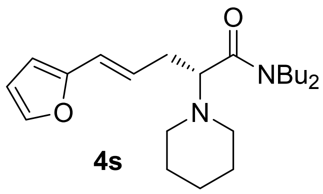

4s

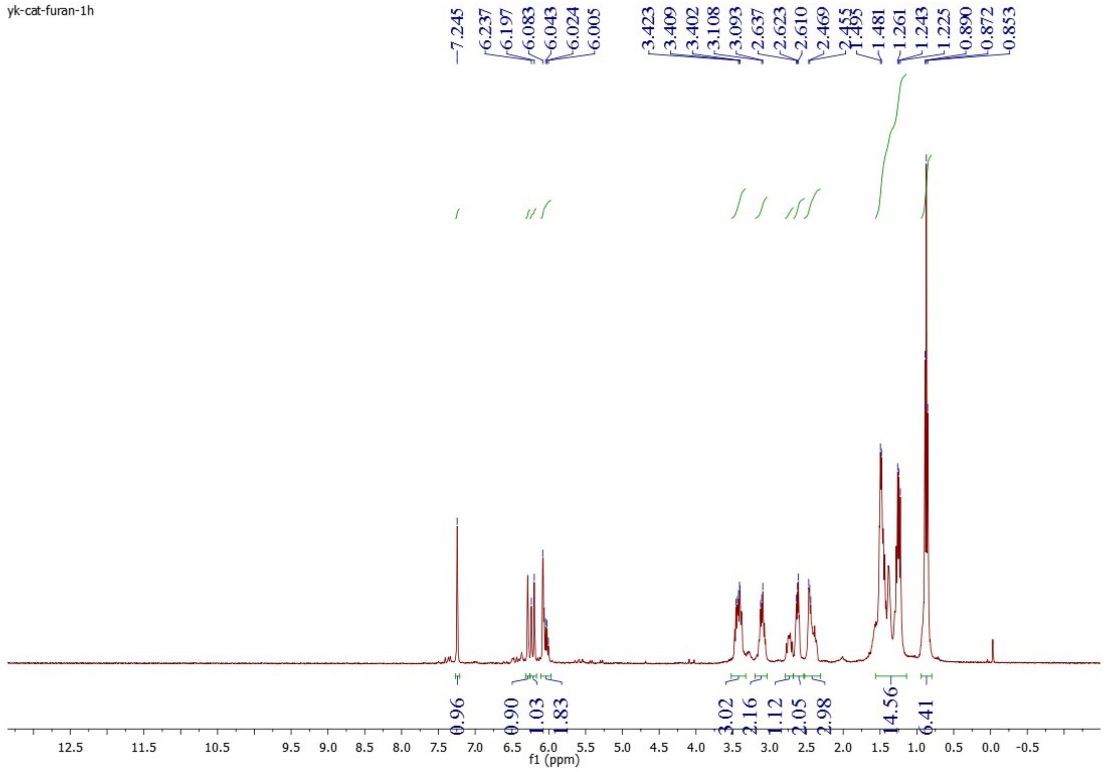

yk-cat-furan-1h
7.245
6.237
6.197
6.083
6.043
6.024
6.005
3.423
3.409
3.402
3.108
3.093
2.637
2.623
2.610
2.469
2.455
1.495
1.481
1.261
1.243
1.225
0.890
0.872
0.853
0.96
0.90
1.03
1.83
3.02
2.16
1.12
2.05
2.98
14.56
6.41
f1 (ppm)

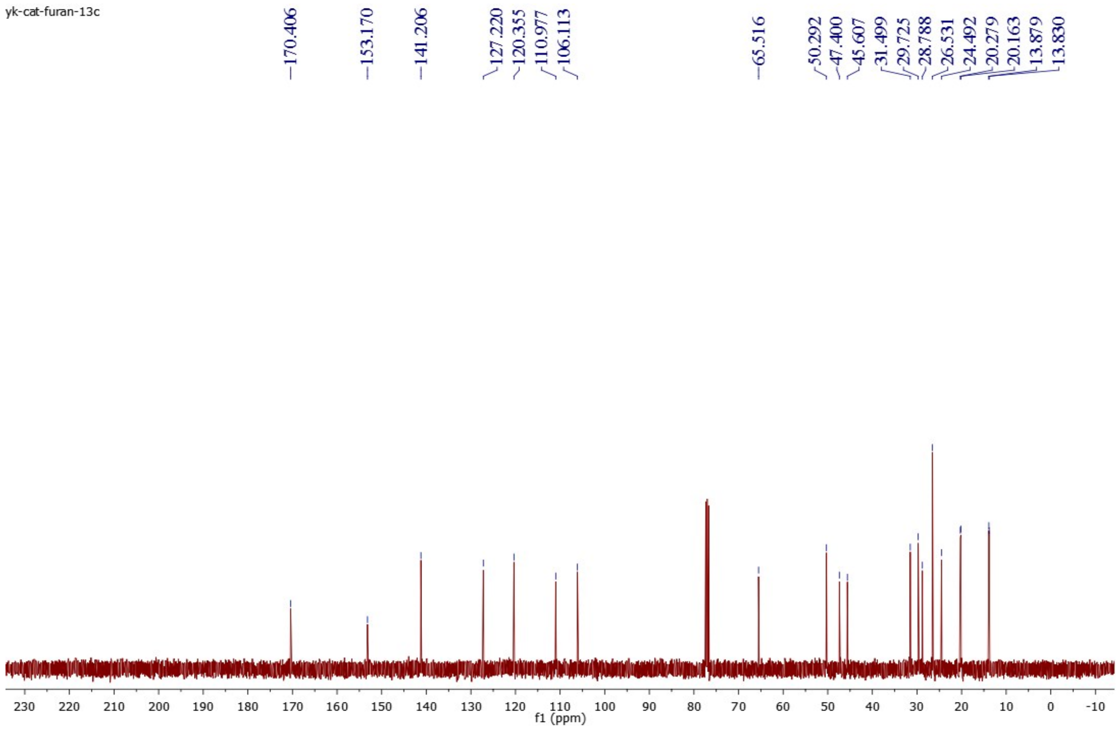

yk-cat-furan-13c
170.406
153.170
141.206
127.220
120.355
110.977
106.113
65.516
50.292
47.400
45.607
31.499
29.725
28.788
26.531
24.492
20.279
20.163
13.879
13.830
f1 (ppm)

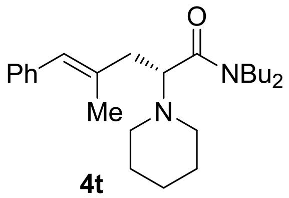
O
Ph
NBu2
Me
N

4t

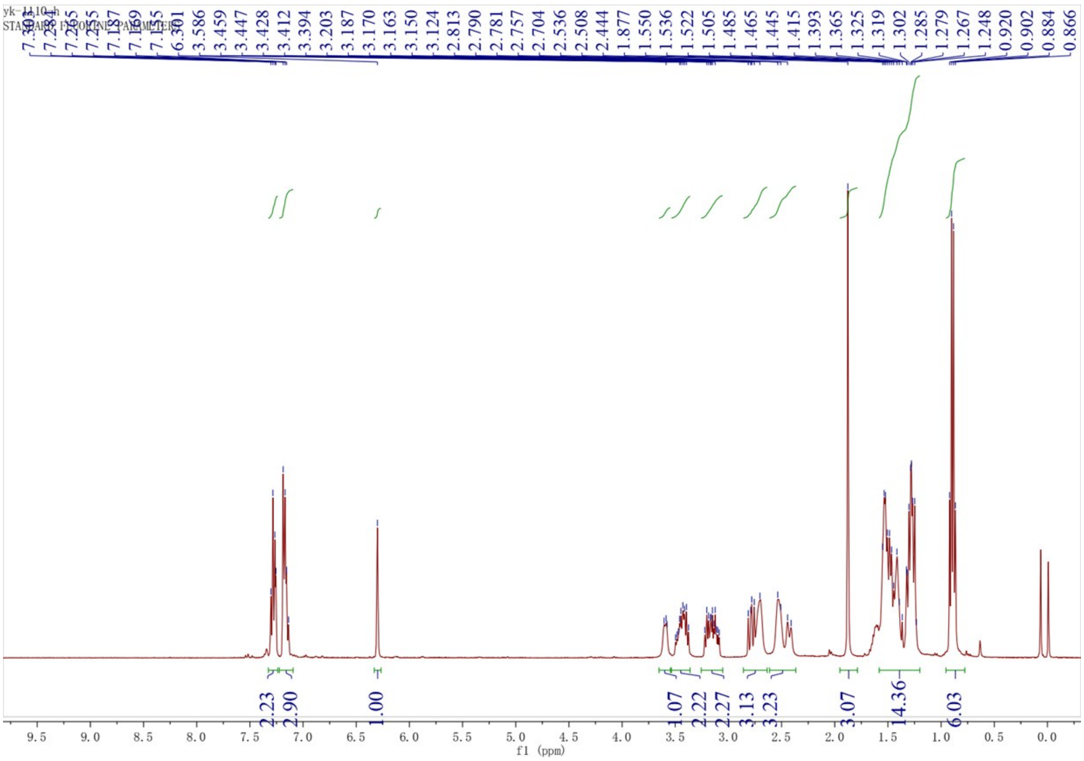
yk-1110-h
7.255
7.187
7.155
6.301
3.586
3.459
3.447
3.428
3.412
3.394
3.203
3.187
3.170
3.163
3.150
3.124
2.813
2.790
2.781
2.757
2.704
2.536
2.508
2.444
1.877
1.550
1.536
1.522
1.505
1.485
1.465
1.445
1.415
1.393
1.365
1.325
1.319
1.302
1.285
1.279
1.267
1.248
0.920
0.902
0.884
0.866
2.23
2.90
1.00
1.07
2.22
2.27
3.13
3.23
3.07
14.36
6.03
9.5 9.0 8.5 8.0 7.5 7.0 6.5 6.0 5.5 5.0 4.5 4.0 3.5 3.0 2.5 2.0 1.5 1.0 0.5 0.0
f1 (ppm)

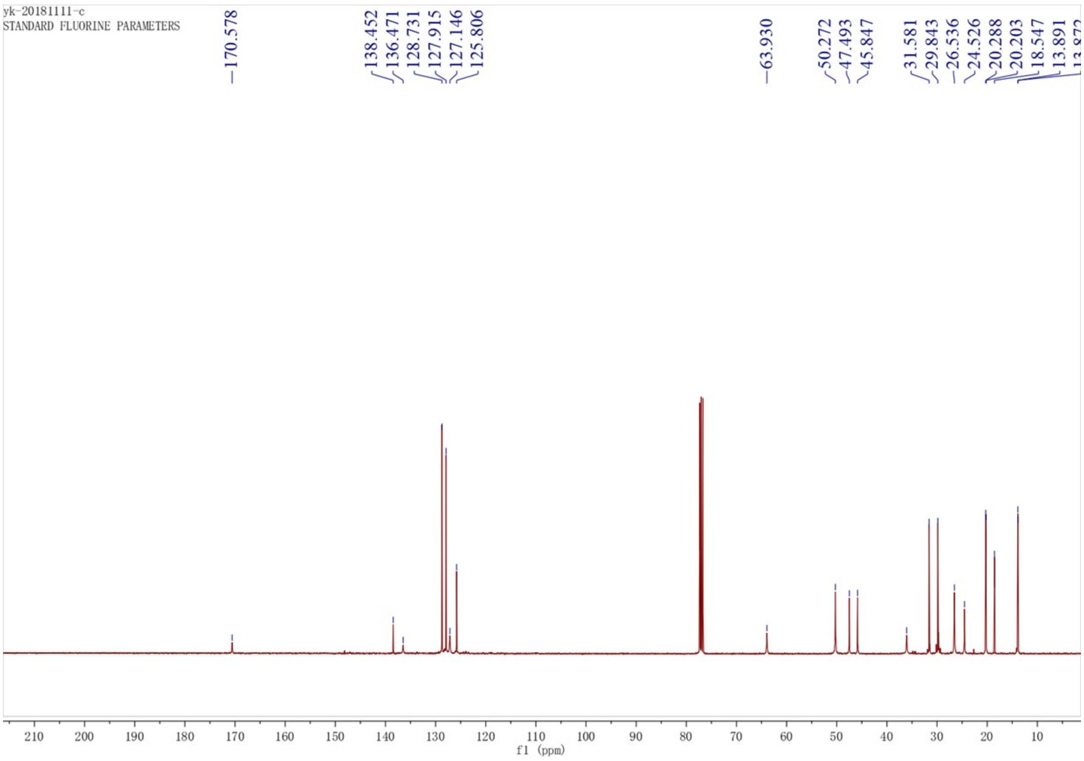
yk-20181111-c
STANDARD FLUORINE PARAMETERS
170.578
138.452
136.471
128.731
127.915
127.146
125.806
63.930
50.272
47.493
45.847
31.581
29.843
26.536
24.526
20.288
20.203
18.547
13.891
210 200 190 180 170 160 150 140 130 120 110 100 90 80 70 60 50 40 30 20 10
f1 (ppm)

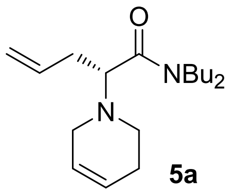

5a

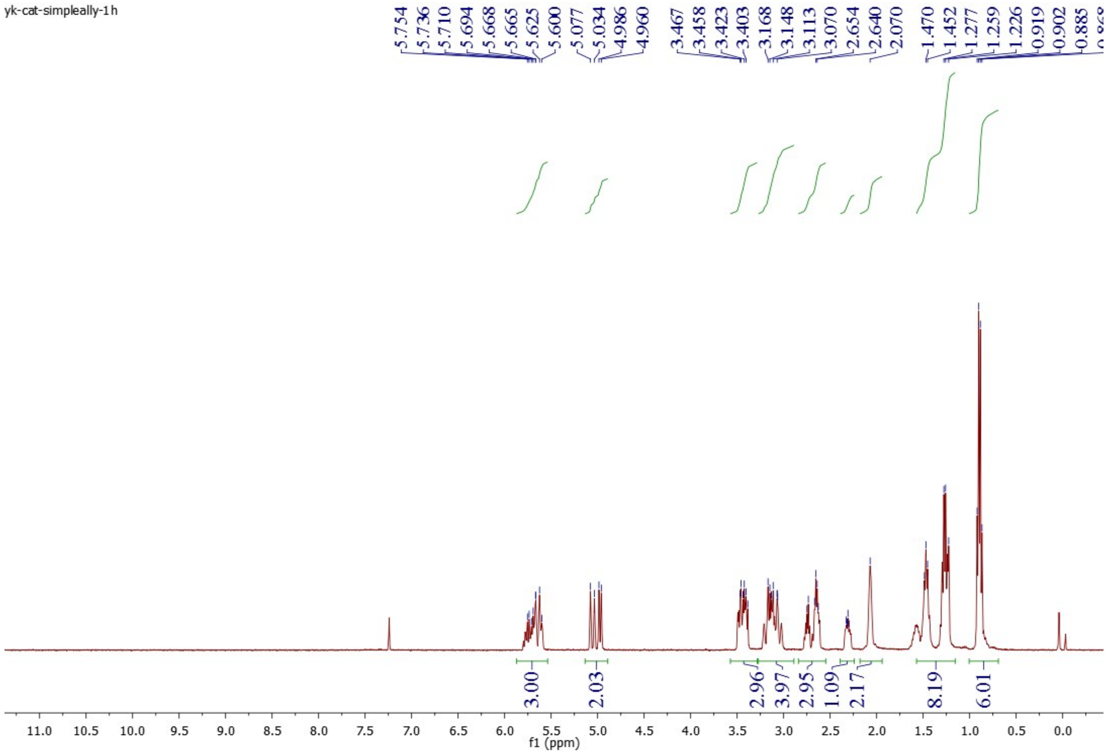
yk-cat-simpleally-1h
5.754
5.736
5.710
5.694
5.668
5.665
5.625
5.600
5.077
5.034
4.986
4.960
3.467
3.458
3.423
3.403
3.168
3.148
3.113
3.070
2.654
2.640
2.070
1.470
1.452
1.277
1.259
1.226
0.919
0.902
0.885
3.00
2.03
2.96
3.97
2.95
1.09
2.17
8.19
6.01
11.0 10.5 10.0 9.5 9.0 8.5 8.0 7.5 7.0 6.5 6.0 5.5 5.0 4.5 4.0 3.5 3.0 2.5 2.0 1.5 1.0 0.5 0.0
f1 (ppm)

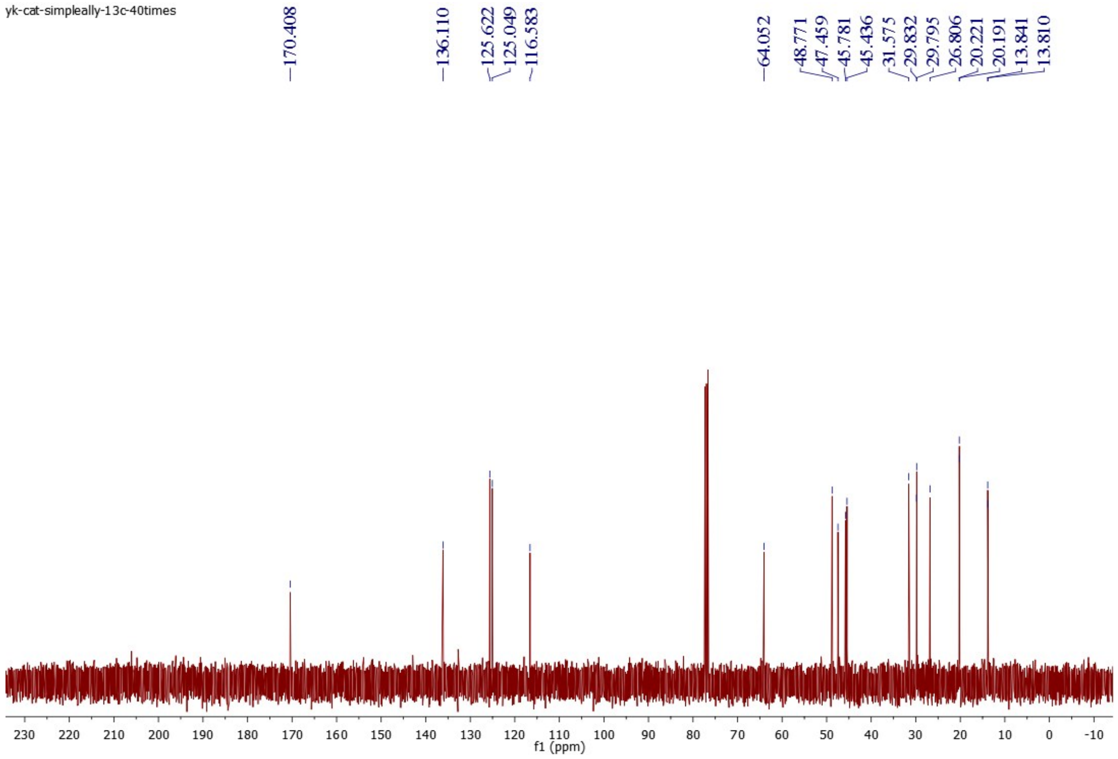
yk-cat-simpleally-13c-40times
170.408
136.110
125.622
125.049
116.583
64.052
48.771
47.459
45.781
45.436
31.575
29.832
29.795
26.806
20.221
20.191
13.841
13.810
230 220 210 200 190 180 170 160 150 140 130 120 110 100 90 80 70 60 50 40 30 20 10 0 -10
f1 (ppm)

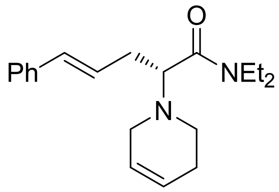
O
Ph
NEt2
N

5aa

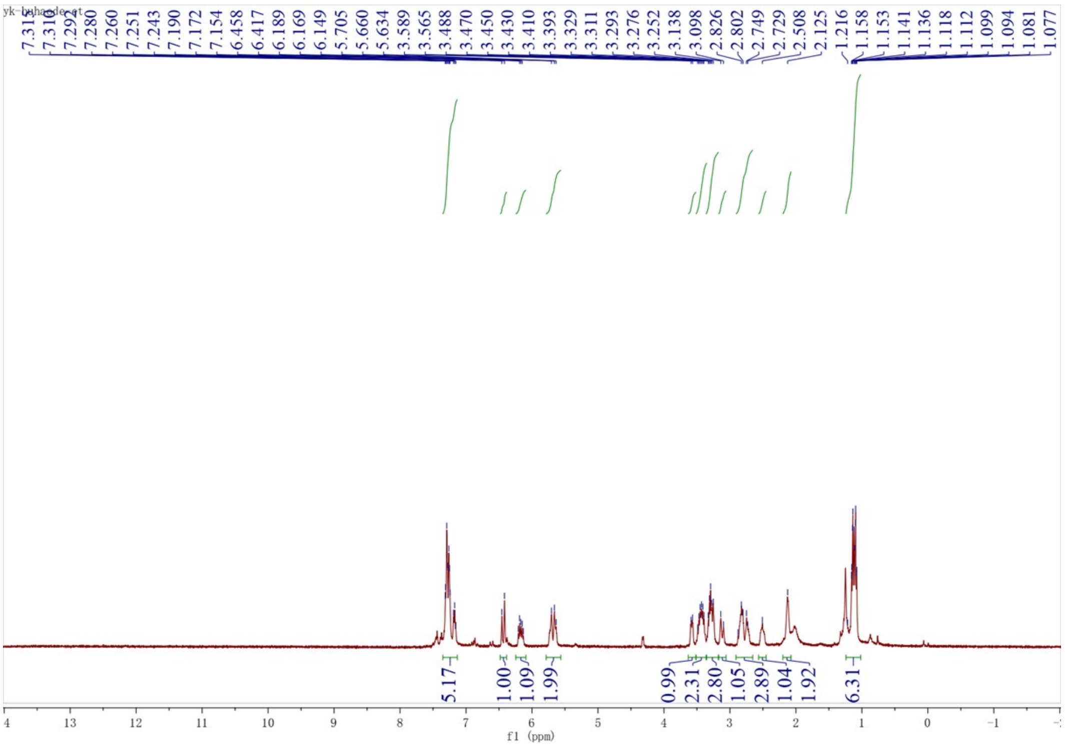
yk-buhaode-et
7.311
7.310
7.292
7.280
7.260
7.251
7.243
7.190
7.172
7.154
6.458
6.417
6.189
6.169
6.149
5.705
5.660
5.634
3.589
3.565
3.488
3.470
3.450
3.430
3.410
3.393
3.329
3.311
3.293
3.276
3.252
3.138
3.098
2.826
2.802
2.749
2.729
2.508
2.125
1.216
1.158
1.153
1.141
1.136
1.118
1.112
1.099
1.094
1.081
1.077
5.17
1.00
1.09
1.99
0.99
2.31
2.80
1.05
2.89
1.04
1.92
6.31
f1 (ppm)

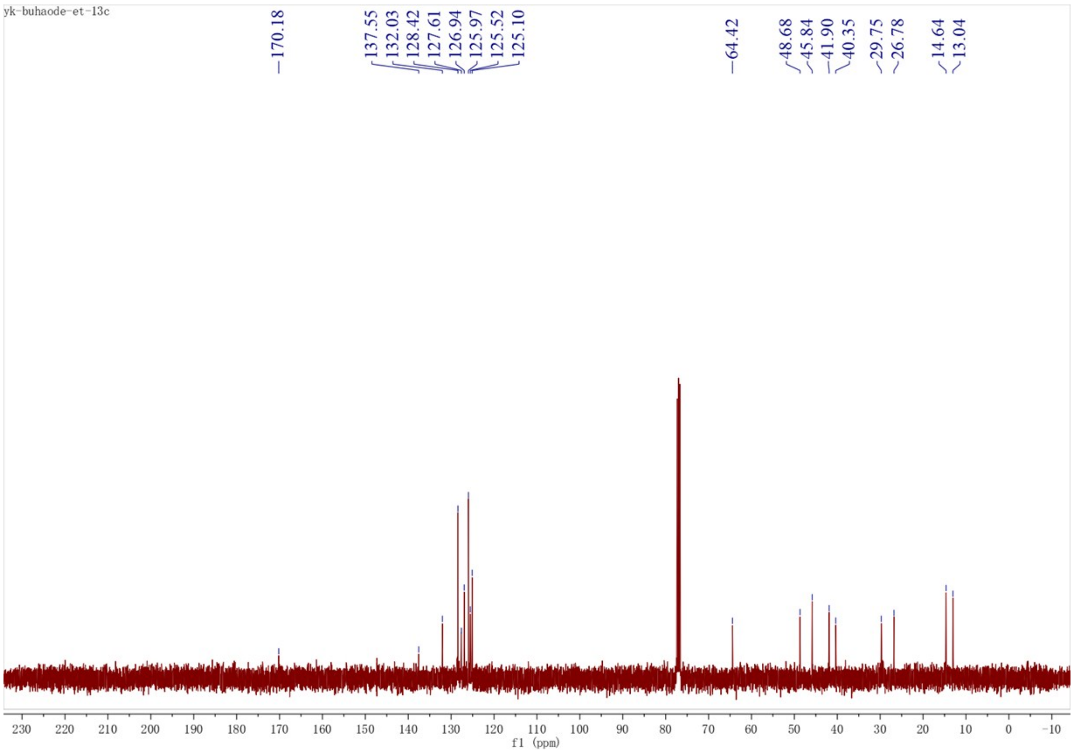
yk-buhaode-et-13c
170.18
137.55
132.03
128.42
127.61
126.94
125.97
125.52
125.10
64.42
48.68
45.84
41.90
40.35
29.75
26.78
14.64
13.04
f1 (ppm)

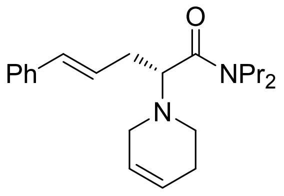

5ab

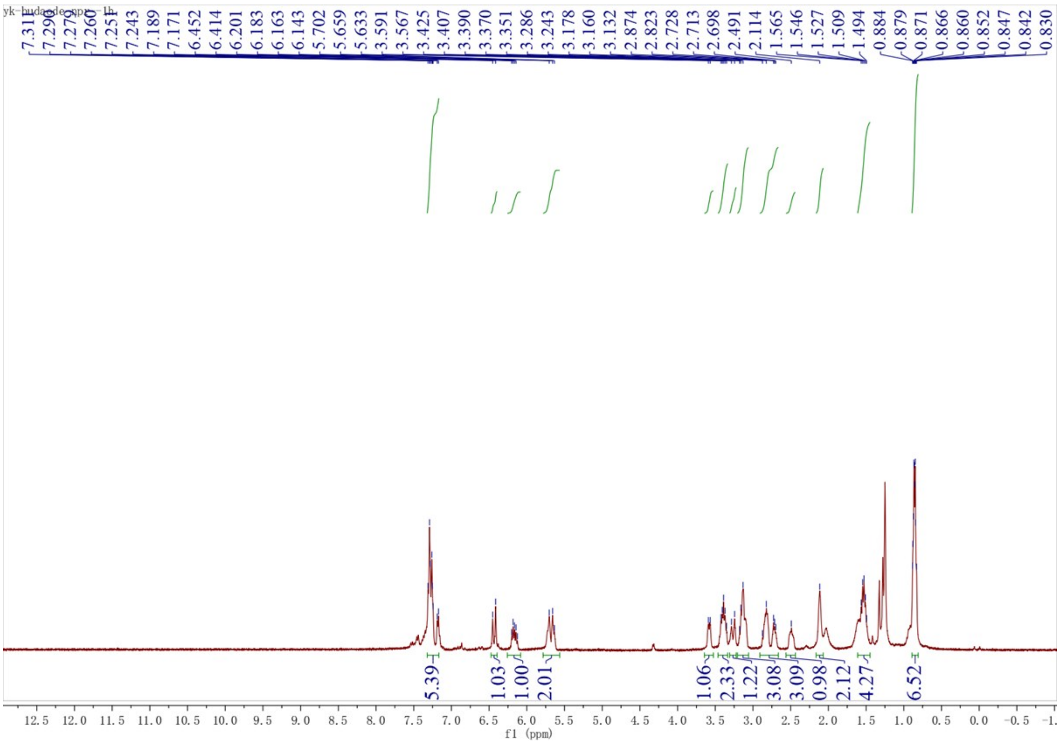

yk-budaode-npr--1h
7.311
7.290
7.273
7.260
7.251
7.243
7.189
7.171
6.452
6.414
6.201
6.183
6.163
6.143
5.702
5.659
5.633
3.591
3.567
3.425
3.407
3.390
3.370
3.351
3.286
3.243
3.178
3.160
3.132
2.874
2.823
2.728
2.713
2.698
2.491
2.114
1.565
1.546
1.527
1.509
1.494
0.884
0.879
0.871
0.866
0.860
0.852
0.847
0.842
0.830
5.39
1.03
1.00
2.01
1.06
2.33
1.22
3.08
3.09
0.98
2.12
4.27
6.52
f1 (ppm)

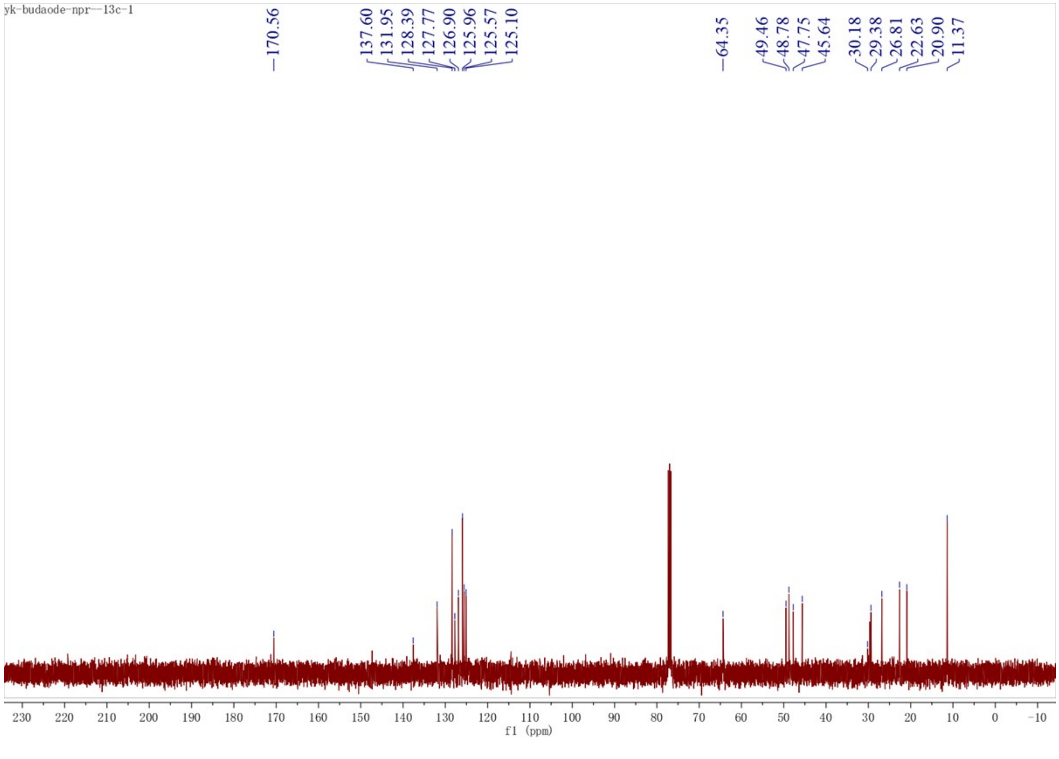

yk-budaode-npr--13c-1
170.56
137.60
131.95
128.39
127.77
126.90
125.96
125.57
125.10
64.35
49.46
48.78
47.75
45.64
30.18
29.38
26.81
22.63
20.90
11.37
f1 (ppm)

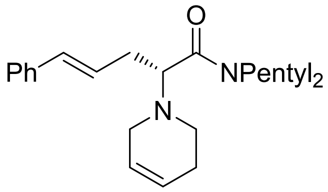

5ad

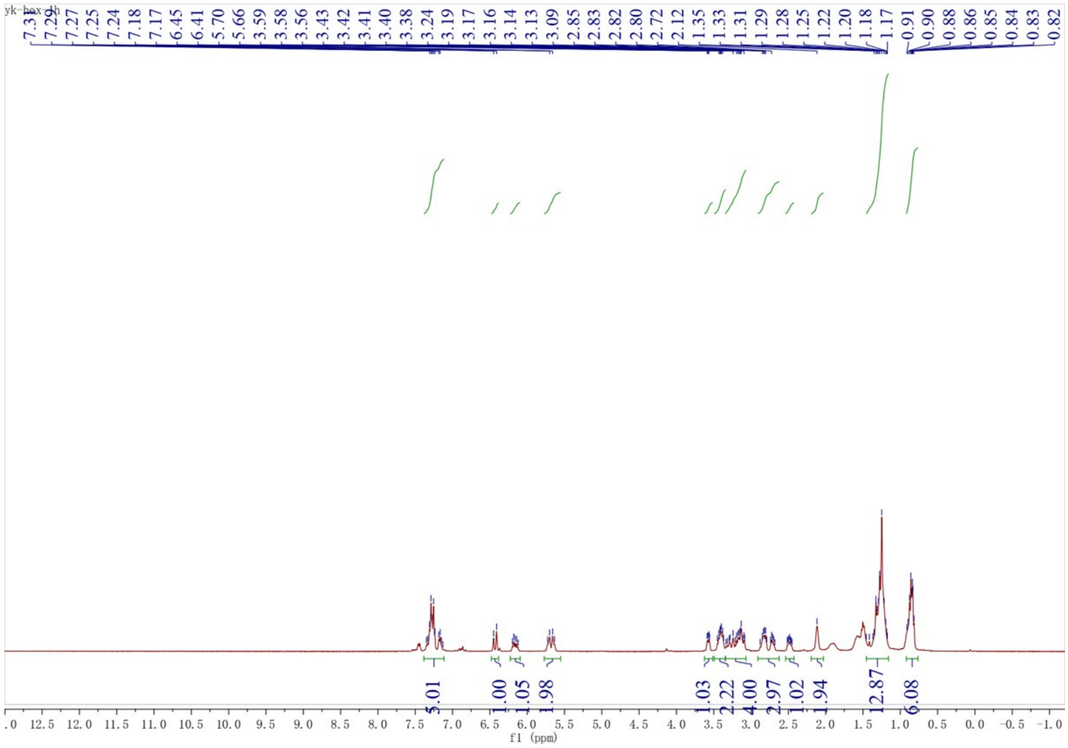
yk-hex-1h
7.31 7.29 7.27 7.25 7.24 7.18 7.17 6.45 6.41 5.70 5.66 3.59 3.58 3.56 3.43 3.42 3.41 3.40 3.38 3.24 3.19 3.17 3.16 3.14 3.13 3.09 2.85 2.83 2.82 2.80 2.72 2.12 1.35 1.33 1.31 1.29 1.28 1.25 1.22 1.20 1.18 1.17 0.91 0.90 0.88 0.86 0.85 0.84 0.83 0.82
5.01 1.00 1.05 1.98 1.03 2.22 4.00 2.97 1.02 1.94 12.87 6.08
f1 (ppm)

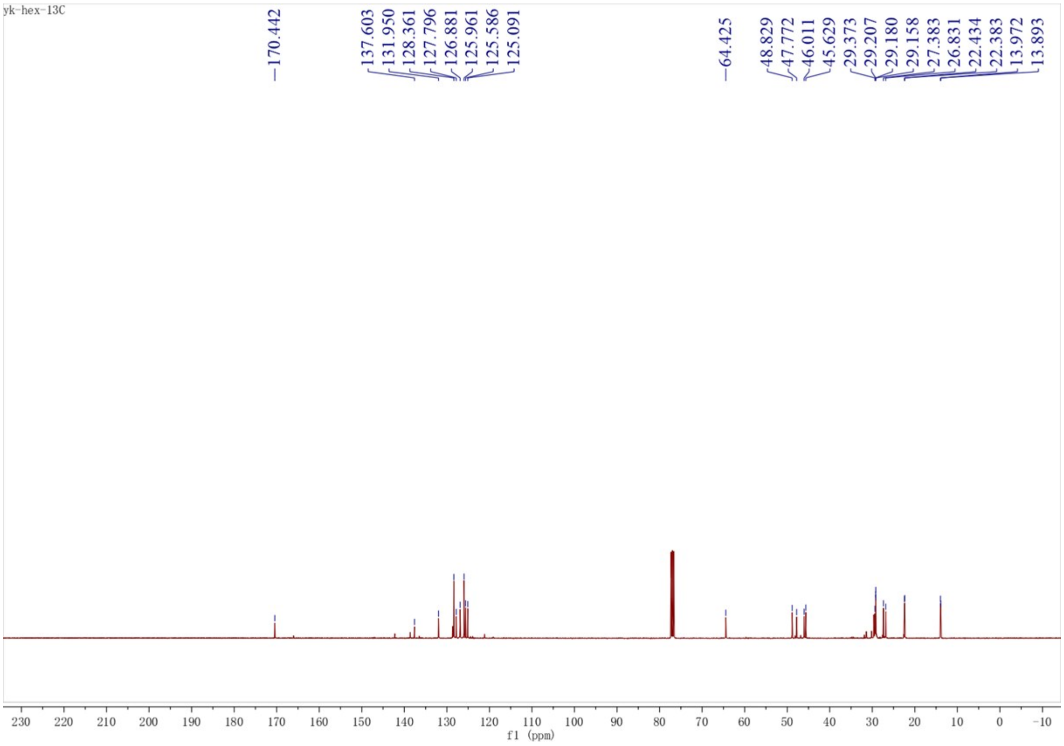
yk-hex-13C
170.442 137.603 131.950 128.361 127.796 126.881 125.961 125.586 125.091 64.425 48.829 47.772 46.011 45.629 29.373 29.207 29.180 29.158 27.383 26.831 22.434 22.383 13.972 13.893
f1 (ppm)

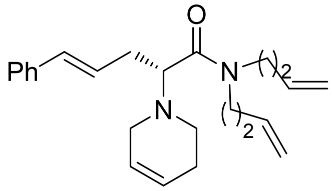

O
Ph
N
N
2
2

5ae

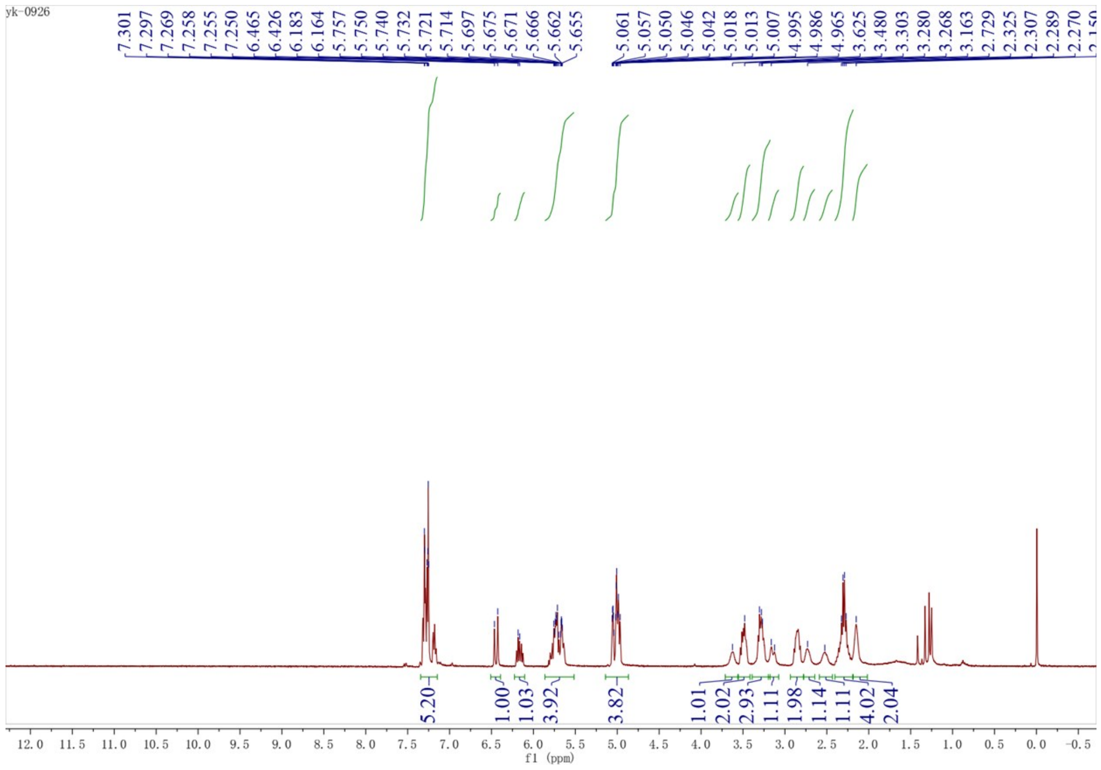

yk-0926
7.301
7.297
7.269
7.258
7.255
7.250
6.465
6.426
6.183
6.164
5.757
5.750
5.740
5.732
5.721
5.714
5.697
5.675
5.671
5.666
5.662
5.655
5.061
5.057
5.050
5.046
5.042
5.018
5.013
5.007
4.995
4.986
4.965
3.625
3.480
3.303
3.280
3.268
3.163
2.729
2.325
2.307
2.289
2.270
5.20
1.00
1.03
3.92
3.82
1.01
2.02
2.93
1.11
1.98
1.14
1.11
4.02
2.04
12.0 11.5 11.0 10.5 10.0 9.5 9.0 8.5 8.0 7.5 7.0 6.5 6.0 5.5 5.0 4.5 4.0 3.5 3.0 2.5 2.0 1.5 1.0 0.5 0.0 -0.5
f1 (ppm)

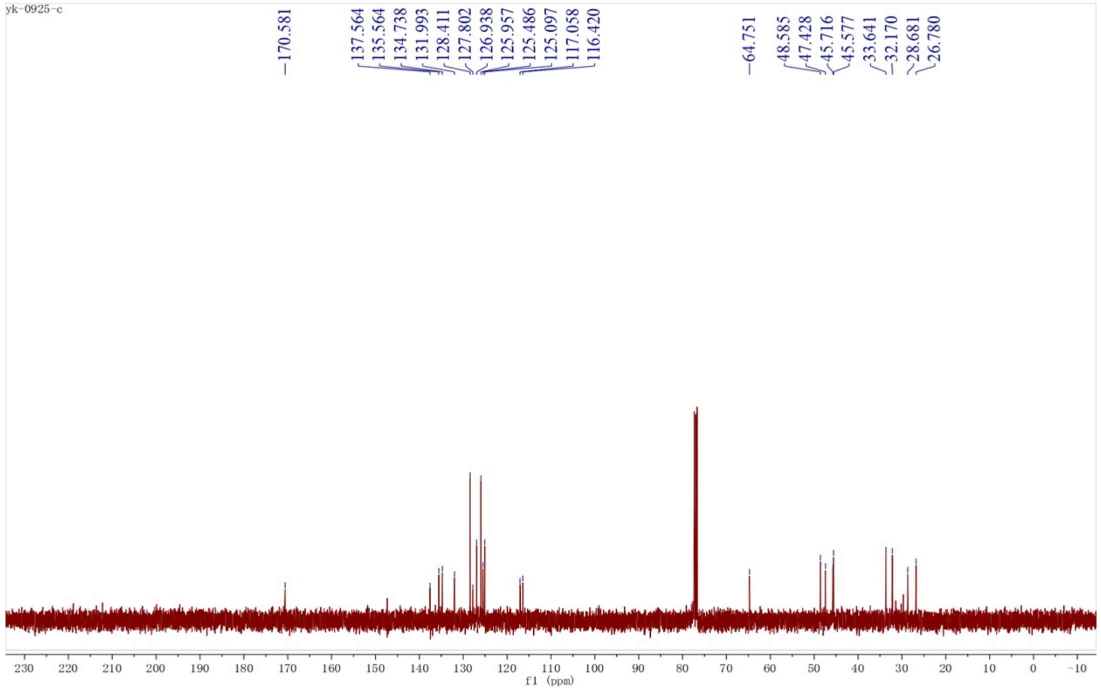

yk-0925-c
170.581
137.564
135.564
134.738
131.993
128.411
127.802
126.938
125.957
125.486
125.097
117.058
116.420
64.751
48.585
47.428
45.716
45.577
33.641
32.170
28.681
26.780
230 220 210 200 190 180 170 160 150 140 130 120 110 100 90 80 70 60 50 40 30 20 10 0 -10
f1 (ppm)

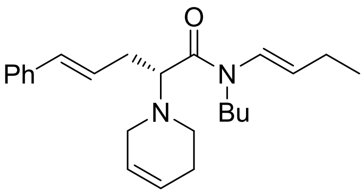

5af

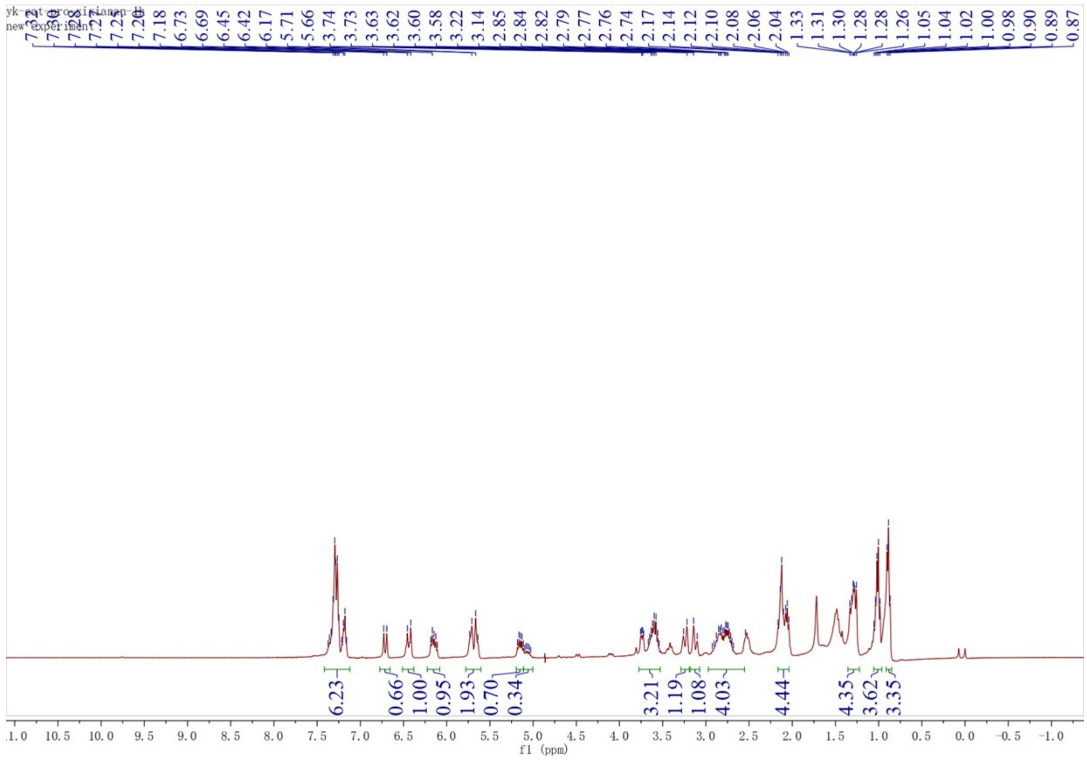

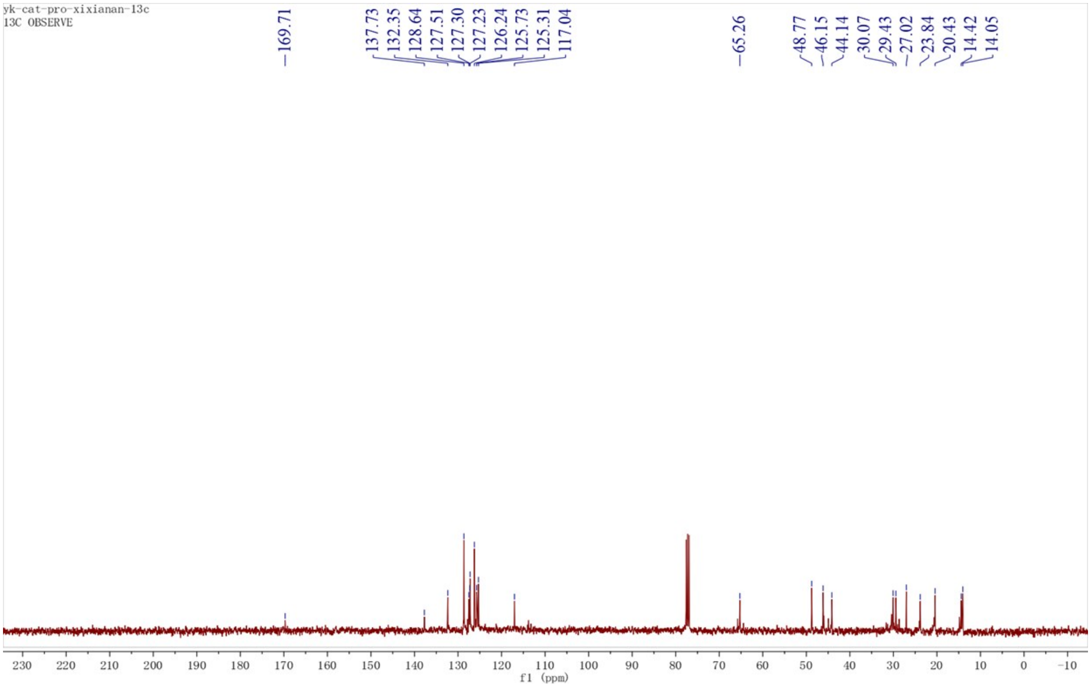

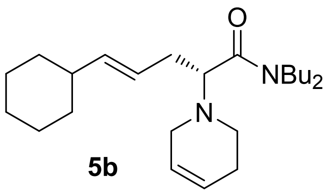
O
NBu2
N

5b

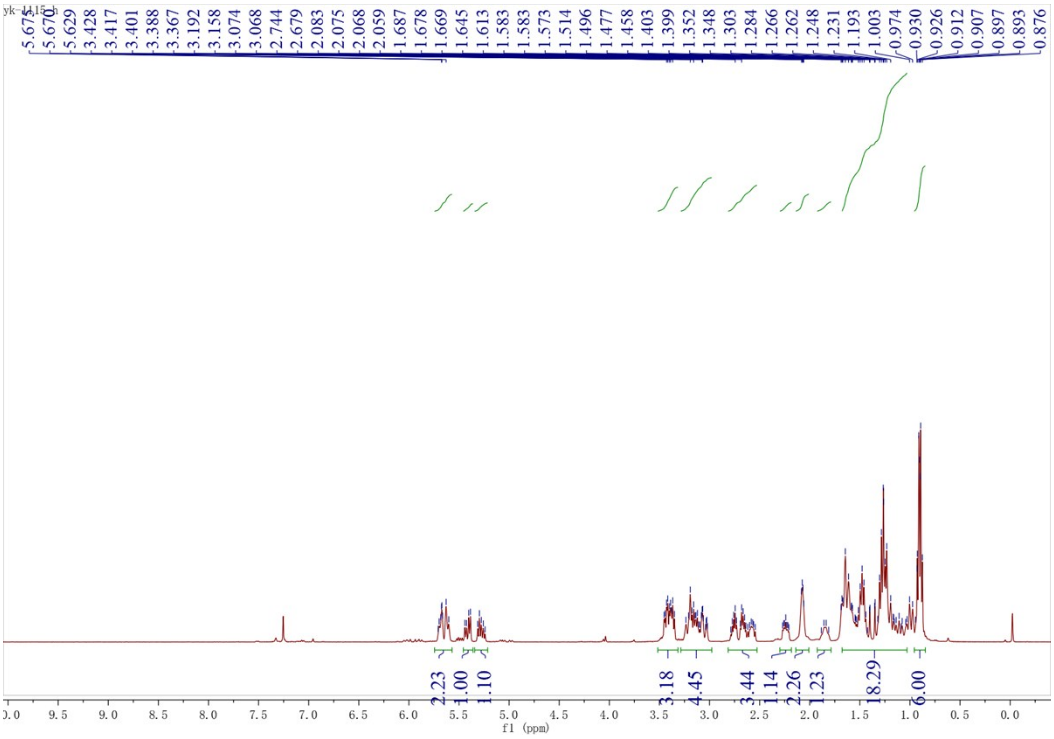
5.675
5.670
5.629
3.428
3.417
3.401
3.388
3.367
3.192
3.158
3.074
3.068
2.744
2.679
2.083
2.075
2.068
2.059
1.687
1.678
1.669
1.645
1.613
1.583
1.583
1.573
1.514
1.496
1.477
1.458
1.403
1.399
1.352
1.348
1.303
1.284
1.266
1.262
1.248
1.231
1.193
1.003
0.974
0.930
0.926
0.912
0.907
0.897
0.893
0.876
2.23
1.00
1.10
3.18
4.45
3.44
1.14
2.26
1.23
18.29
6.00
f1 (ppm)

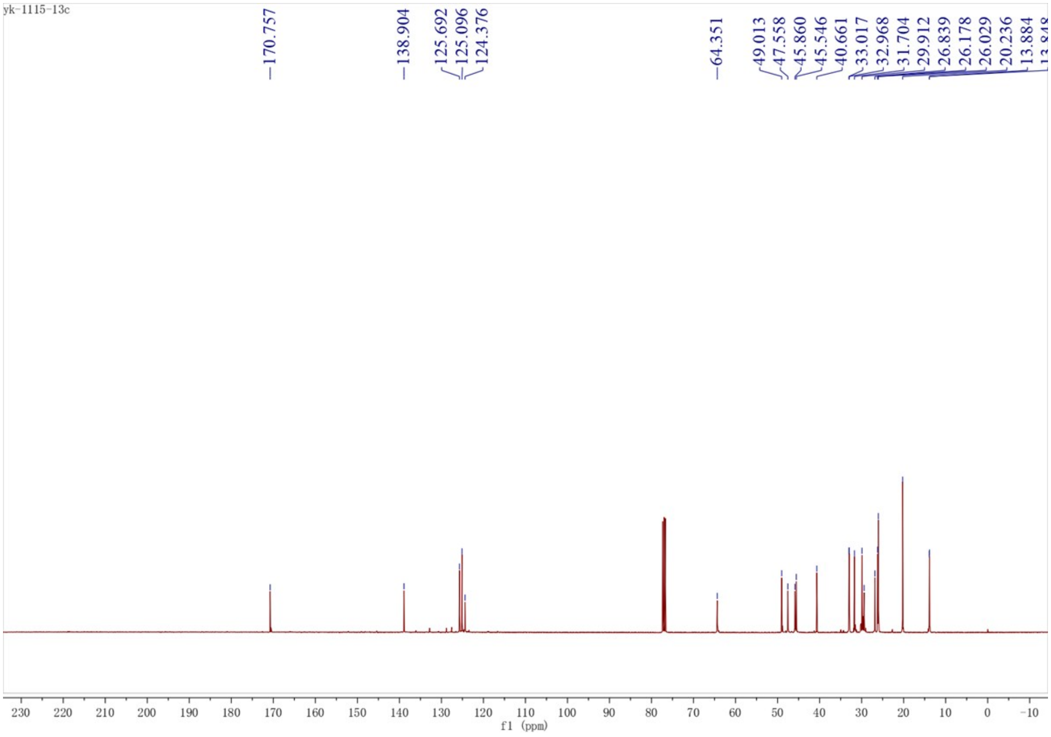
yk-1115-13c
170.757
138.904
125.692
125.096
124.376
64.351
49.013
47.558
45.860
45.546
40.661
33.017
32.968
31.704
29.912
26.839
26.178
26.029
20.236
13.884
f1 (ppm)

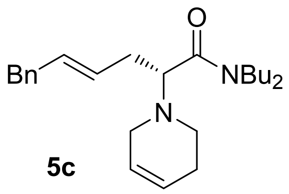

5c

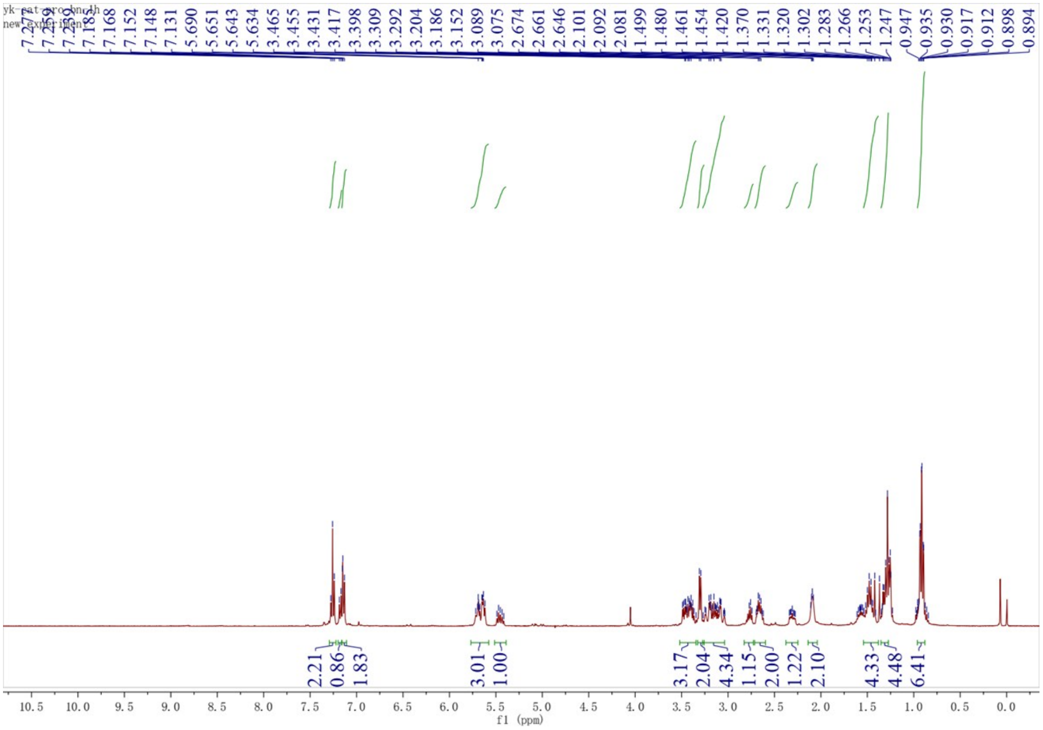
7.168
7.152
7.148
7.131
5.690
5.651
5.643
5.634
3.465
3.455
3.431
3.417
3.398
3.309
3.292
3.204
3.186
3.152
3.089
3.075
2.674
2.661
2.646
2.101
2.092
2.081
1.499
1.480
1.461
1.454
1.420
1.370
1.331
1.320
1.302
1.283
1.266
1.253
1.247
0.947
0.935
0.930
0.917
0.912
0.898
0.894
2.21
0.86
1.83
3.01
1.00
3.17
2.04
4.34
1.15
2.00
1.22
2.10
4.33
4.48
6.41
f1 (ppm)

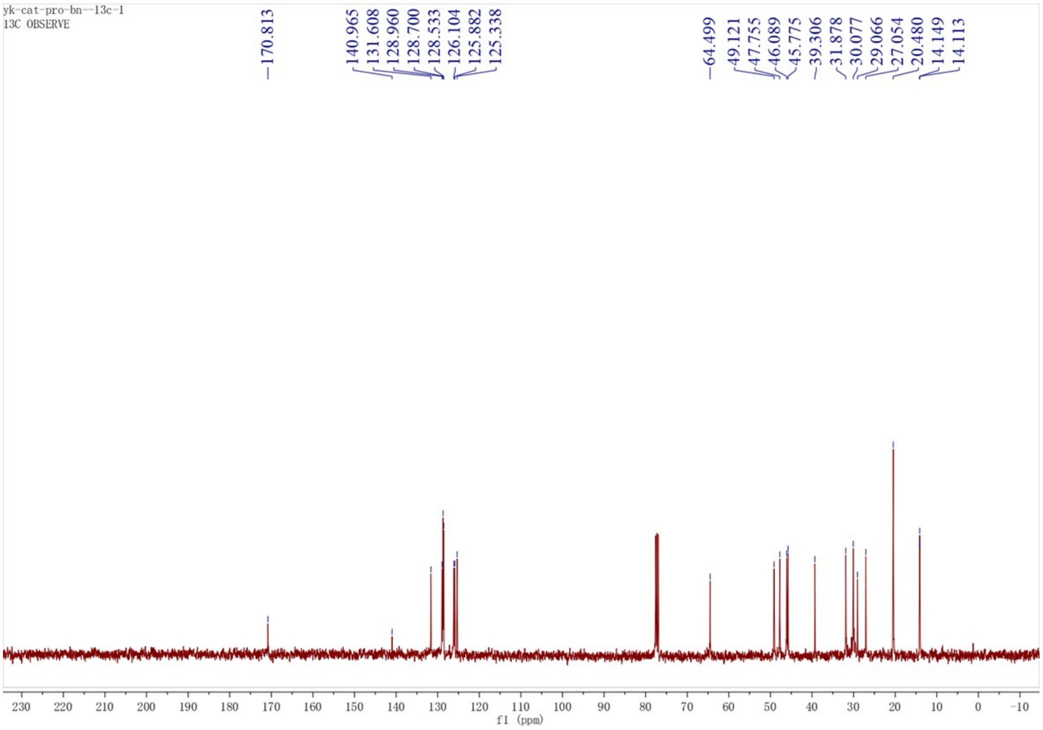
yk-cat-pro-bn--13c-1
13C OBSERVE
170.813
140.965
131.608
128.960
128.700
128.533
126.104
125.882
125.338
64.499
49.121
47.755
46.089
45.775
39.306
31.878
30.077
29.066
27.054
20.480
14.149
14.113
f1 (ppm)

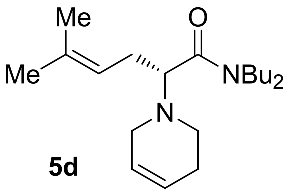
Me
Me
O
NBu2
N

5d

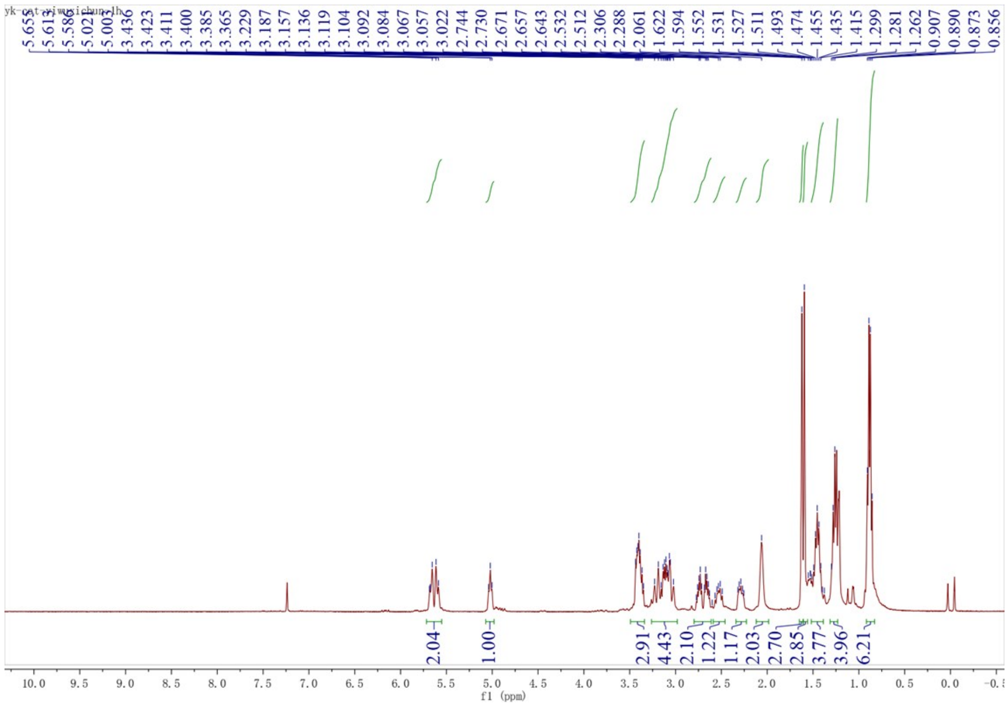
yk-cat-yiwuxichun-1h
5.655 5.613 5.586 5.024 5.003 3.436 3.423 3.411 3.400 3.385 3.365 3.229 3.187 3.157 3.136 3.119 3.104 3.092 3.084 3.067 3.057 3.022 2.744 2.730 2.671 2.657 2.643 2.532 2.512 2.306 2.288 2.061 1.622 1.594 1.552 1.531 1.527 1.511 1.493 1.474 1.455 1.435 1.415 1.299 1.281 1.262 0.907 0.890 0.873 0.856
2.04 1.00 2.91 4.43 2.10 1.22 1.17 2.03 2.70 2.85 3.77 3.96 6.21
f1 (ppm)

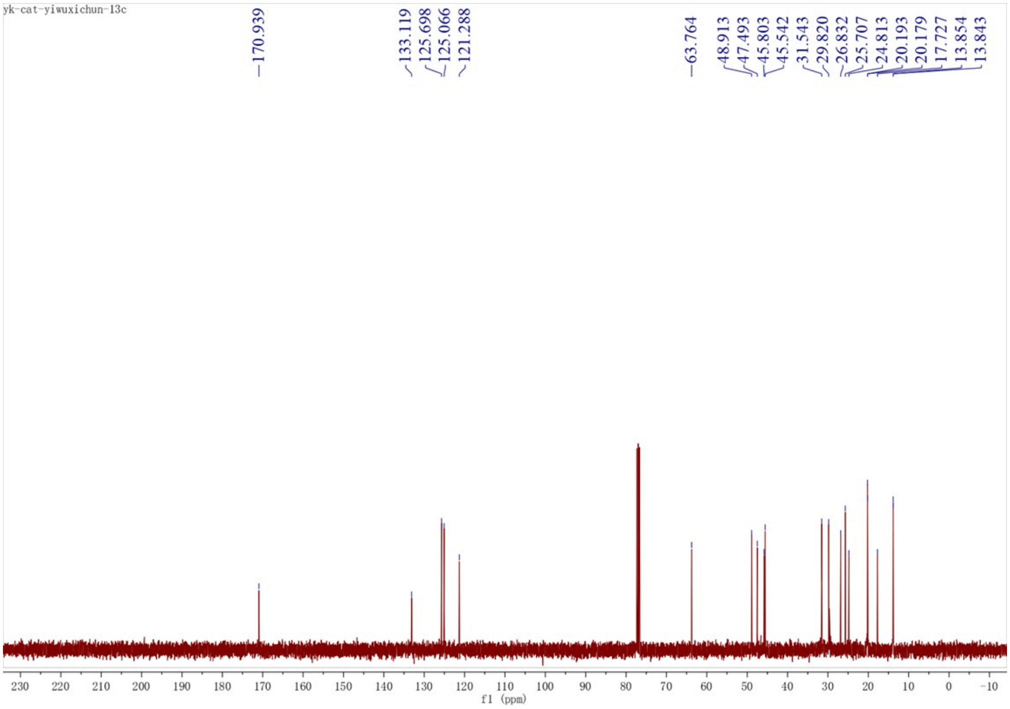
yk-cat-yiwuxichun-13c
170.939
133.119 125.698 125.066 121.288
63.764
48.913 47.493 45.803 45.542 31.543 29.820 26.832 25.707 24.813 20.193 20.179 17.727 13.854 13.843
f1 (ppm)

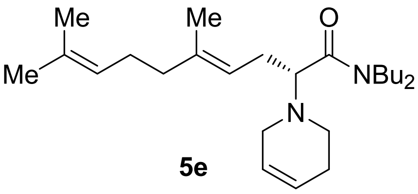

5e

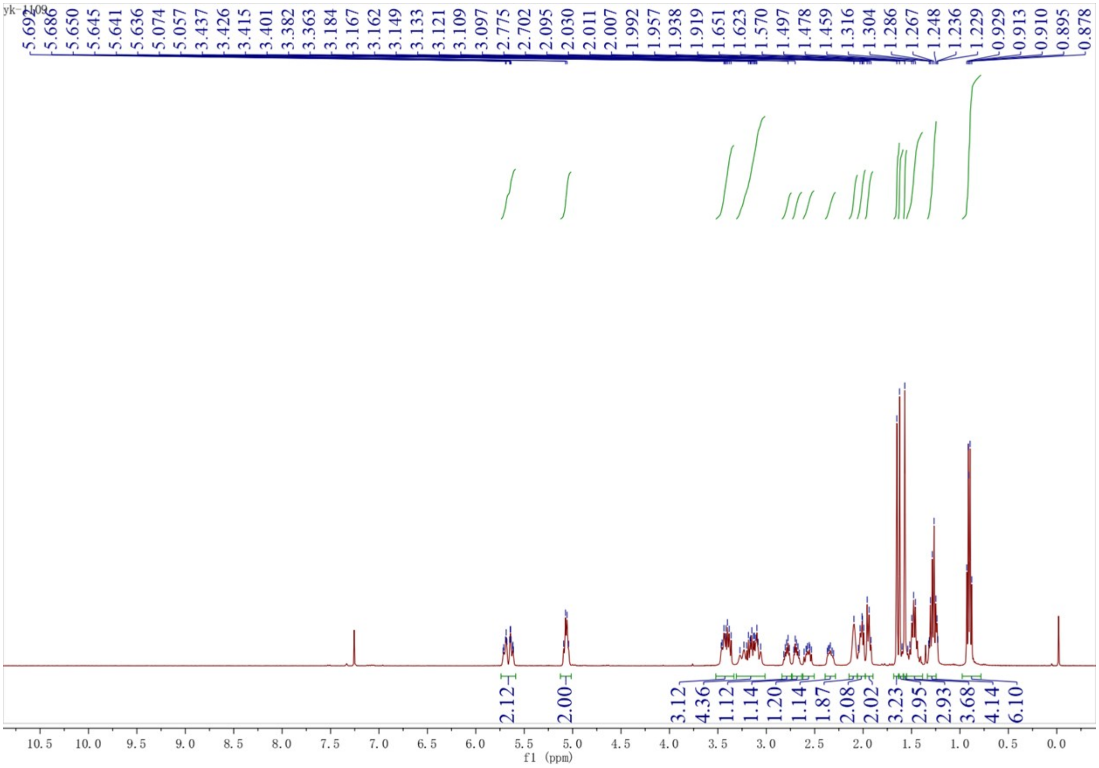

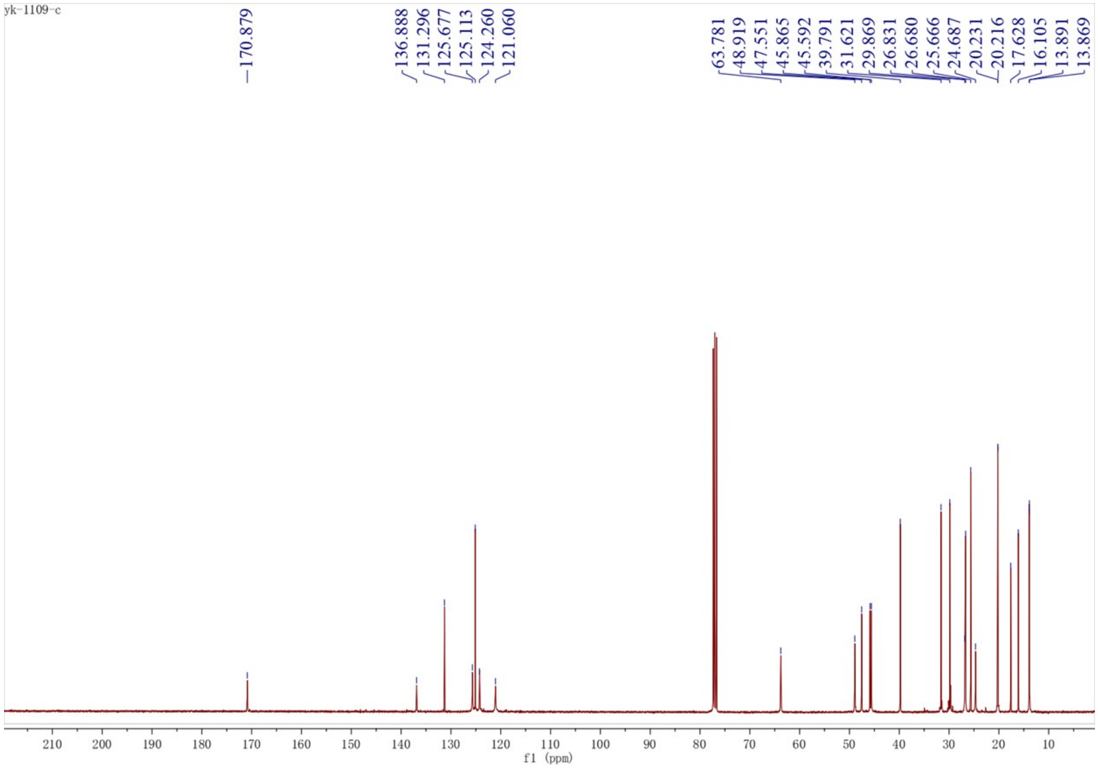

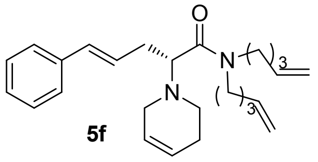

5f

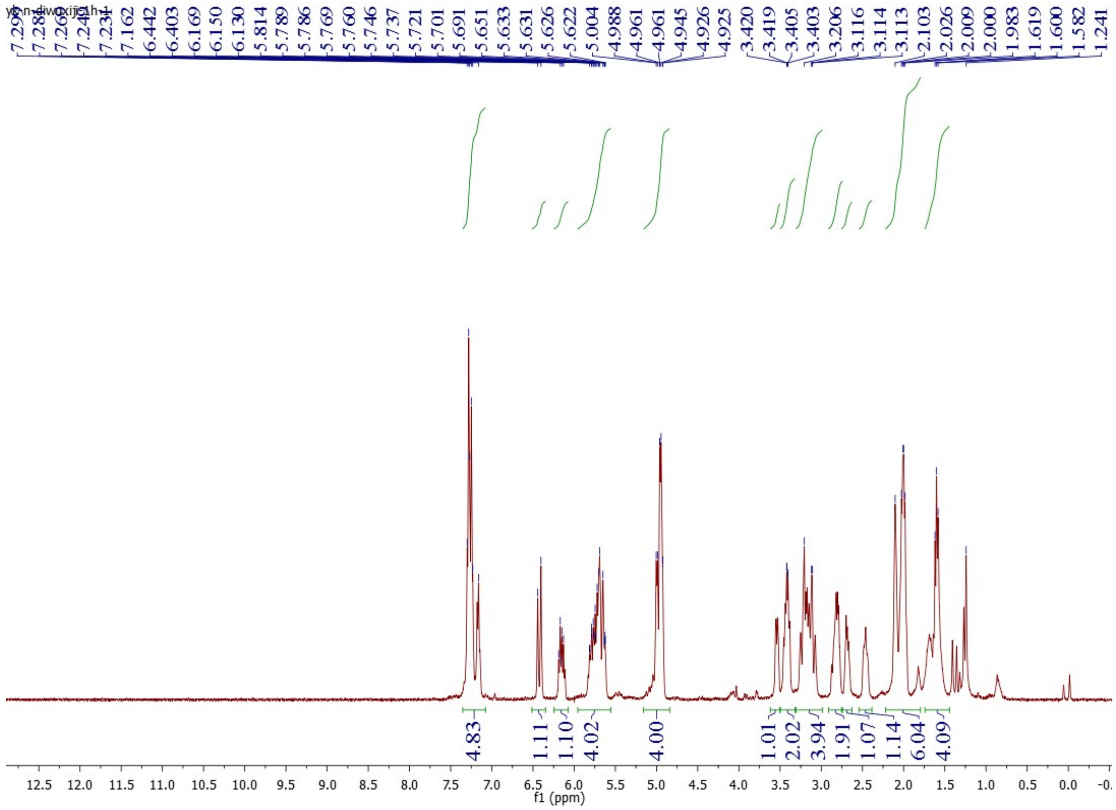

yk-n-diwuxiji-1h-1
7.299
7.281
7.265
7.241
7.231
7.162
6.442
6.403
6.169
6.150
6.130
5.814
5.789
5.786
5.769
5.760
5.746
5.737
5.721
5.701
5.691
5.651
5.633
5.631
5.626
5.622
5.004
4.988
4.961
4.961
4.945
4.926
4.925
3.420
3.419
3.405
3.403
3.206
3.116
3.114
3.113
2.103
2.026
2.009
2.000
1.983
1.619
1.600
1.582
1.241
4.83
1.11
1.10
4.02
4.00
1.01
2.02
3.94
1.91
1.07
1.14
6.04
4.09
f1 (ppm)

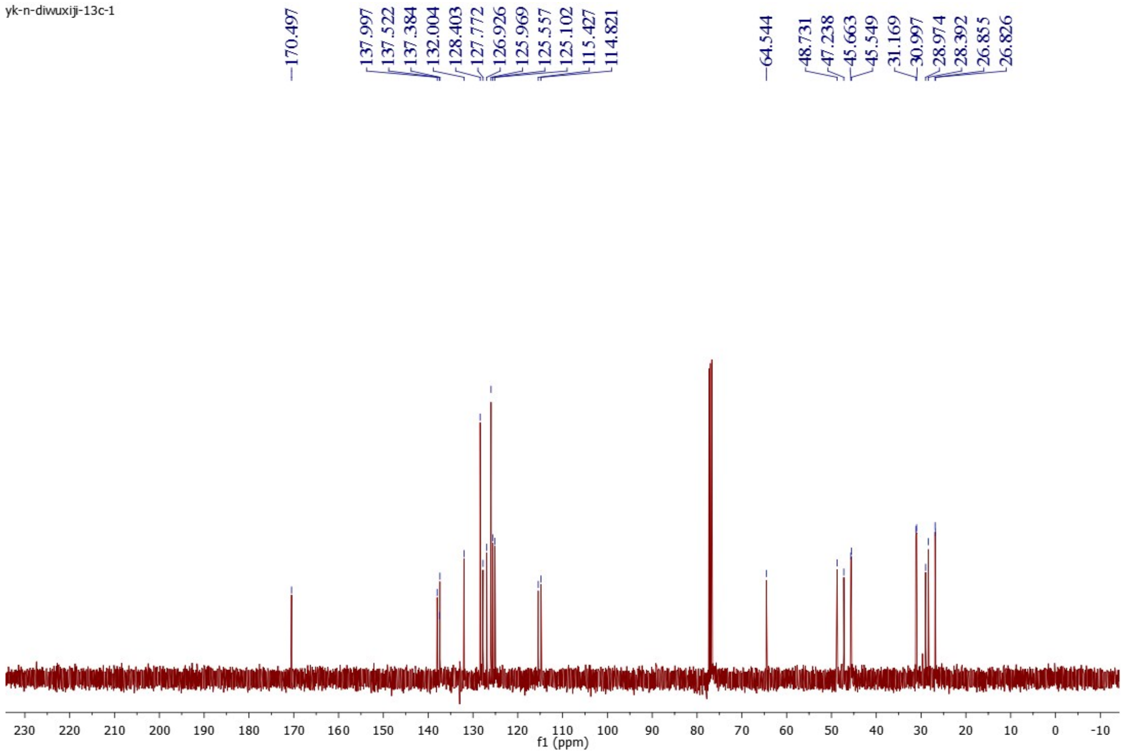

yk-n-diwuxiji-13c-1
170.497
137.997
137.522
137.384
132.004
128.403
127.772
126.926
125.969
125.557
125.102
115.427
114.821
64.544
48.731
47.238
45.663
45.549
31.169
30.997
28.974
28.392
26.855
26.826
f1 (ppm)

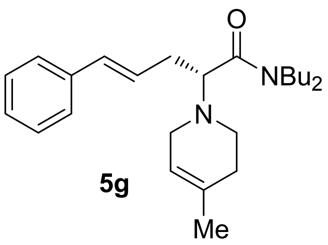

O
NBu2
N
Me

5g

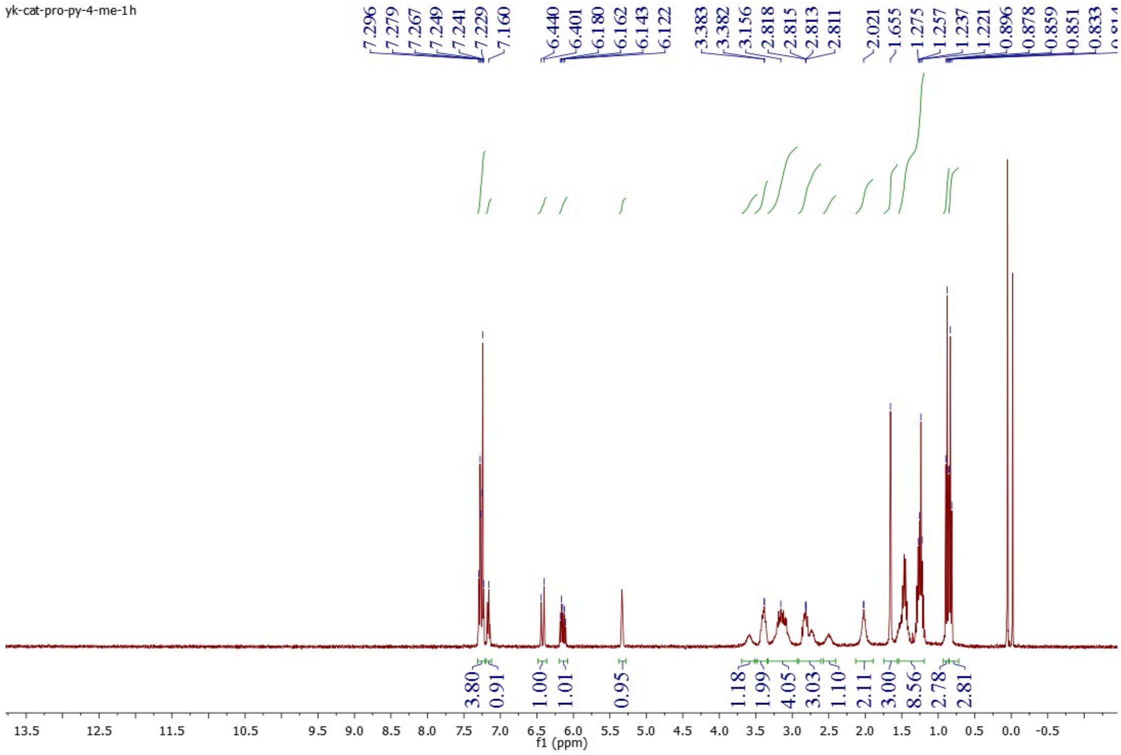

yk-cat-pro-py-4-me-1h
7.296
7.279
7.267
7.249
7.241
7.229
7.160
6.440
6.401
6.180
6.162
6.143
6.122
3.383
3.382
3.156
2.818
2.815
2.813
2.811
2.021
1.655
1.275
1.257
1.237
1.221
0.896
0.878
0.859
0.851
0.833
3.80
0.91
1.00
1.01
0.95
1.18
1.99
4.05
3.03
1.10
2.11
3.00
8.56
2.78
2.81
13.5
12.5
11.5
10.5
9.5
9.0
8.5
8.0
7.5
7.0
6.5
6.0
5.5
5.0
4.5
4.0
3.5
3.0
2.5
2.0
1.5
1.0
0.5
0.0
-0.5
f1 (ppm)

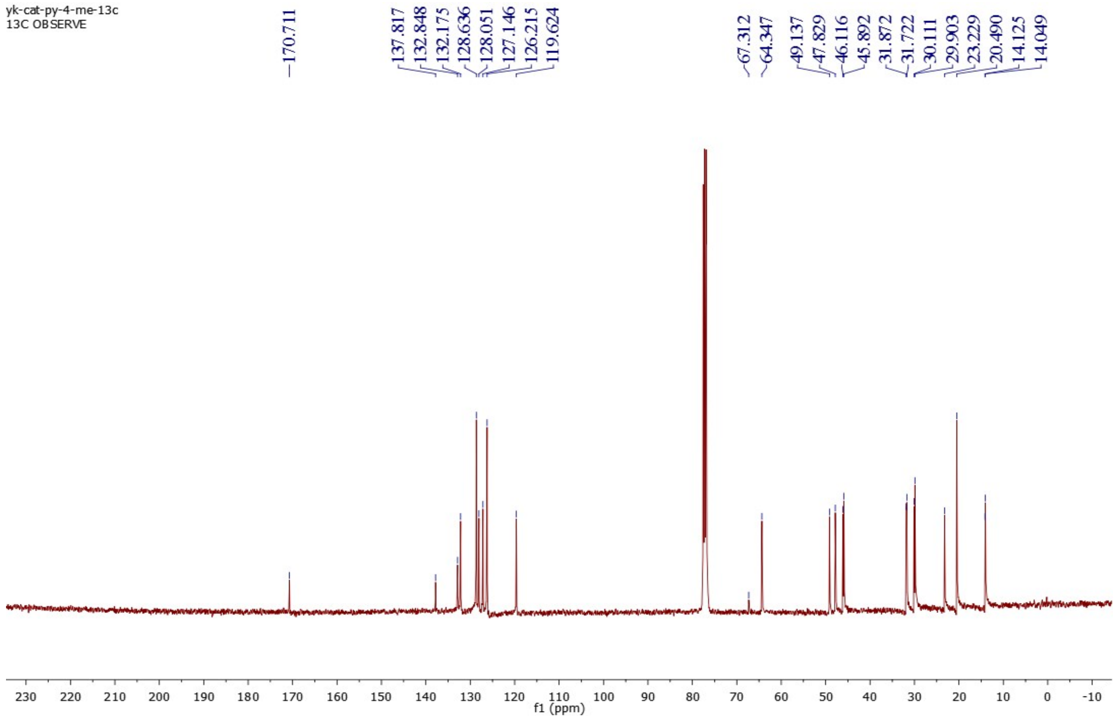

yk-cat-py-4-me-13c
13C OBSERVE
170.711
137.817
132.848
132.175
128.636
128.051
127.146
126.215
119.624
67.312
64.347
49.137
47.829
46.116
45.892
31.872
31.722
30.111
29.903
23.229
20.490
14.125
14.049
230
220
210
200
190
180
170
160
150
140
130
120
110
100
90
80
70
60
50
40
30
20
10
0
-10
f1 (ppm)

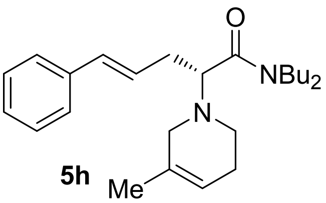
O
NBu2
N
Me

5h

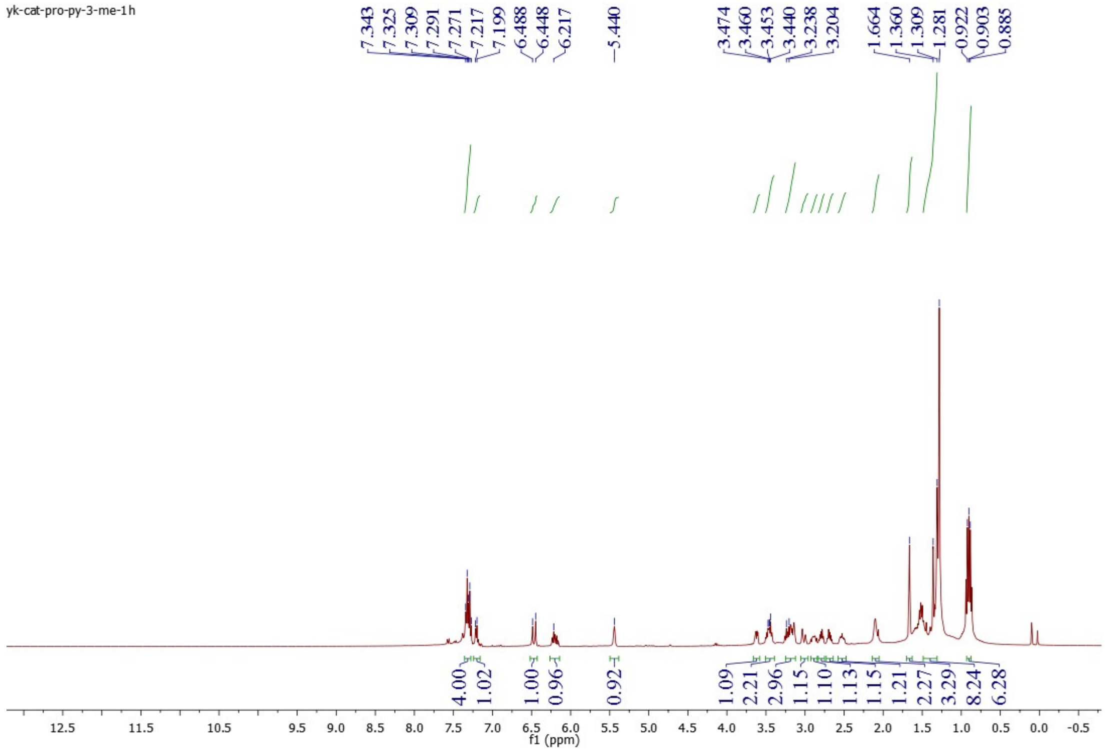
yk-cat-pro-py-3-me-1h
7.343
7.325
7.309
7.291
7.271
7.217
7.199
6.488
6.448
6.217
5.440
3.474
3.460
3.453
3.440
3.238
3.204
1.664
1.360
1.309
1.281
0.922
0.903
0.885
4.00
1.02
1.00
0.96
0.92
1.09
2.21
2.96
1.15
1.10
1.13
1.15
1.21
2.27
3.29
8.24
6.28
f1 (ppm)

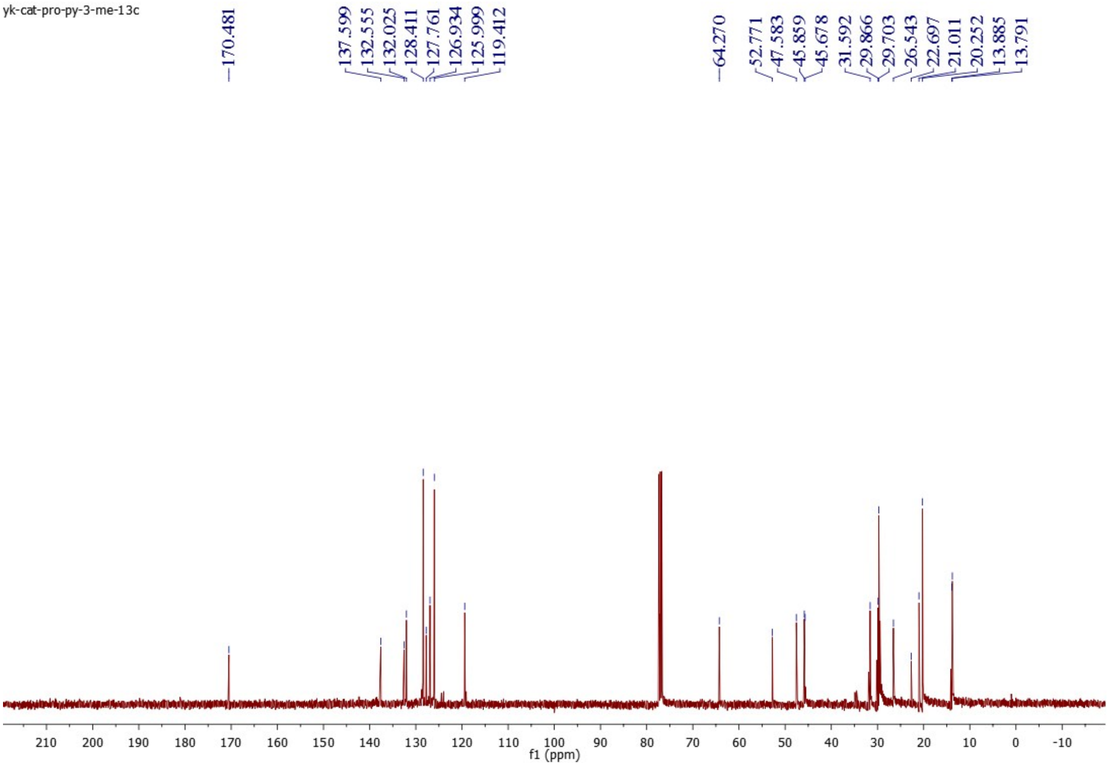
yk-cat-pro-py-3-me-13c
170.481
137.599
132.555
132.025
128.411
127.761
126.934
125.999
119.412
64.270
52.771
47.583
45.859
45.678
31.592
29.866
29.703
26.543
22.697
21.011
20.252
13.885
13.791
f1 (ppm)

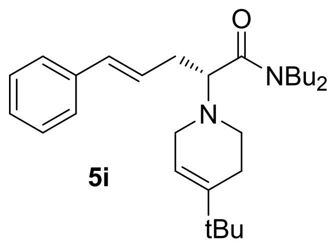

5i

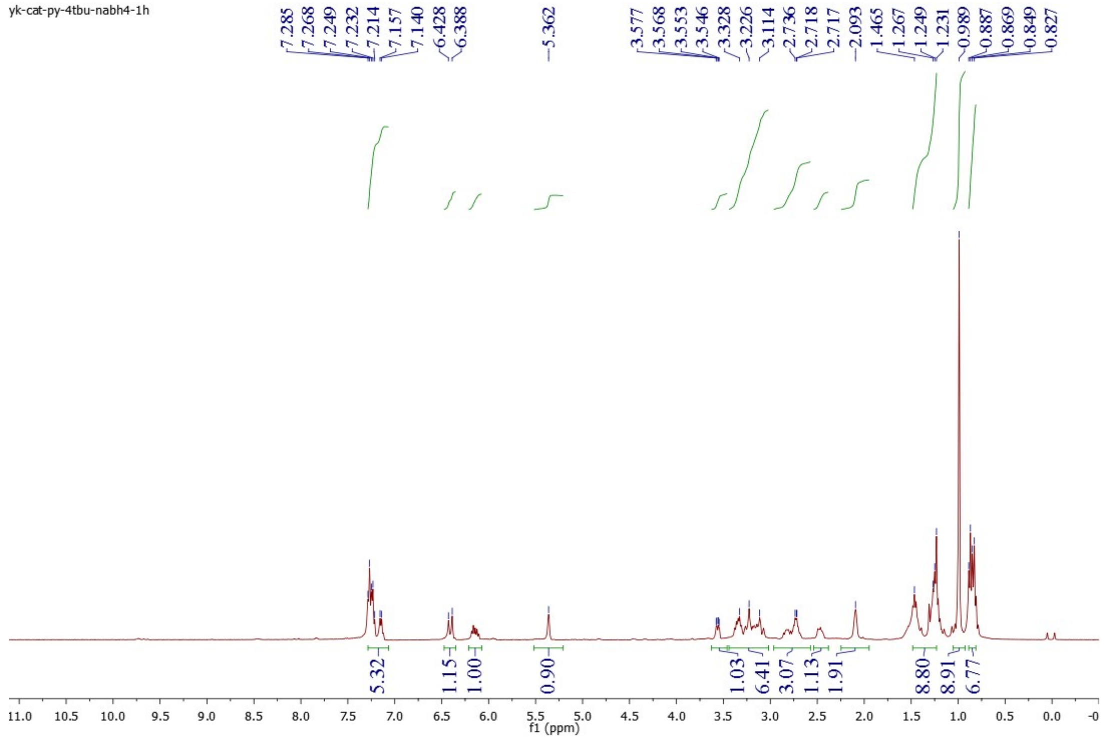

yk-cat-py-4tbu-nabh4-1h
7.285 7.268 7.249 7.232 7.214 7.157 7.140 6.428 6.388 5.362 3.577 3.568 3.553 3.546 3.328 3.226 3.114 2.736 2.718 2.717 2.093 1.465 1.267 1.249 1.231 0.989 0.887 0.869 0.849 0.827
5.32 1.15 1.00 0.90 1.03 6.41 3.07 1.13 1.91 8.80 8.91 6.77
f1 (ppm)

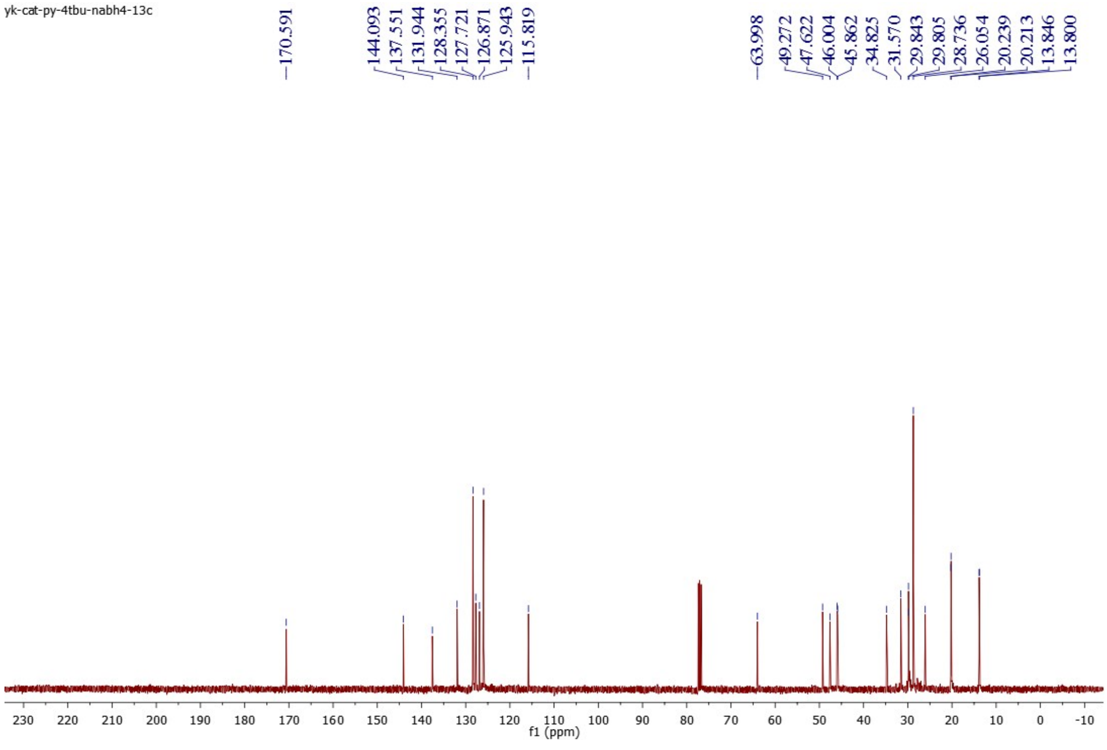

yk-cat-py-4tbu-nabh4-13c
170.591 144.093 137.551 131.944 128.355 127.721 126.871 125.943 115.819 63.998 49.272 47.622 46.004 45.862 34.825 31.570 29.843 29.805 28.736 26.054 20.239 20.213 13.846 13.800
f1 (ppm)

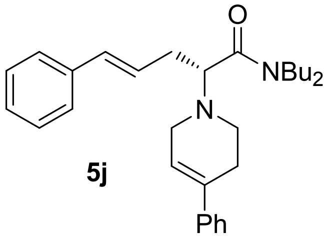
O
NBu2
N
Ph

5j

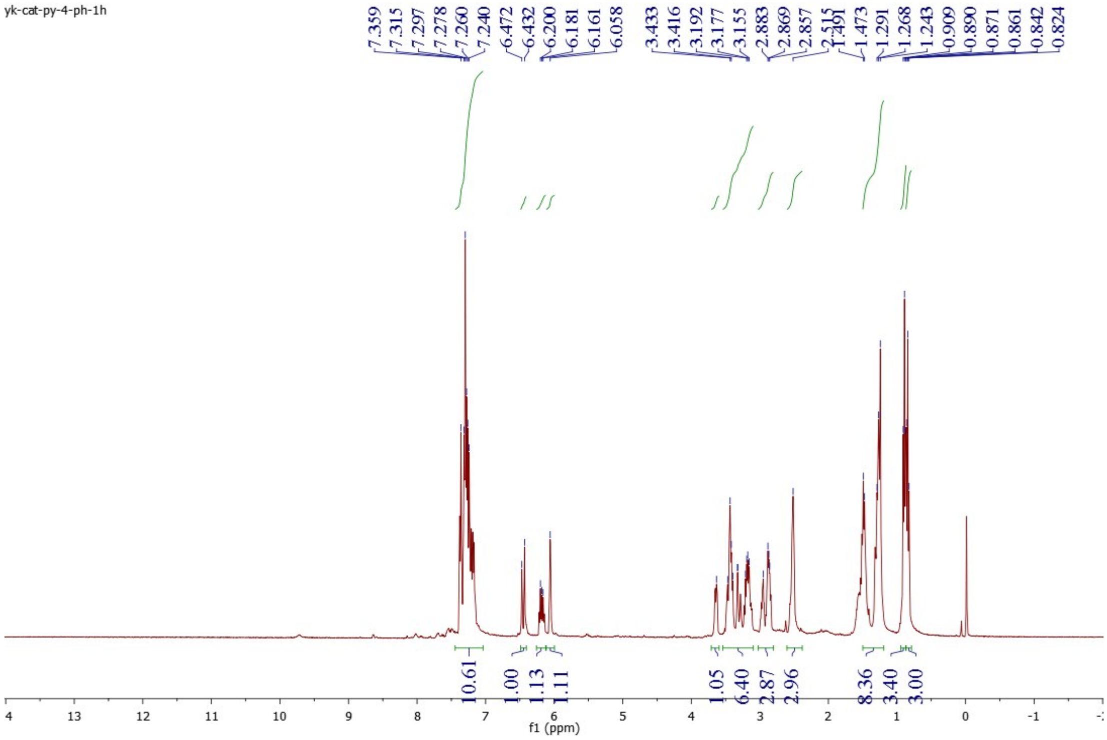
yk-cat-py-4-ph-1h
7.359
7.315
7.297
7.278
7.260
7.240
6.472
6.432
6.200
6.181
6.161
6.058
3.433
3.416
3.192
3.177
3.155
2.883
2.869
2.857
2.515
1.491
1.473
1.291
1.268
1.243
0.909
0.890
0.871
0.861
0.842
0.824
10.61
1.00
1.13
1.11
1.05
6.40
2.87
2.96
8.36
3.40
3.00
f1 (ppm)

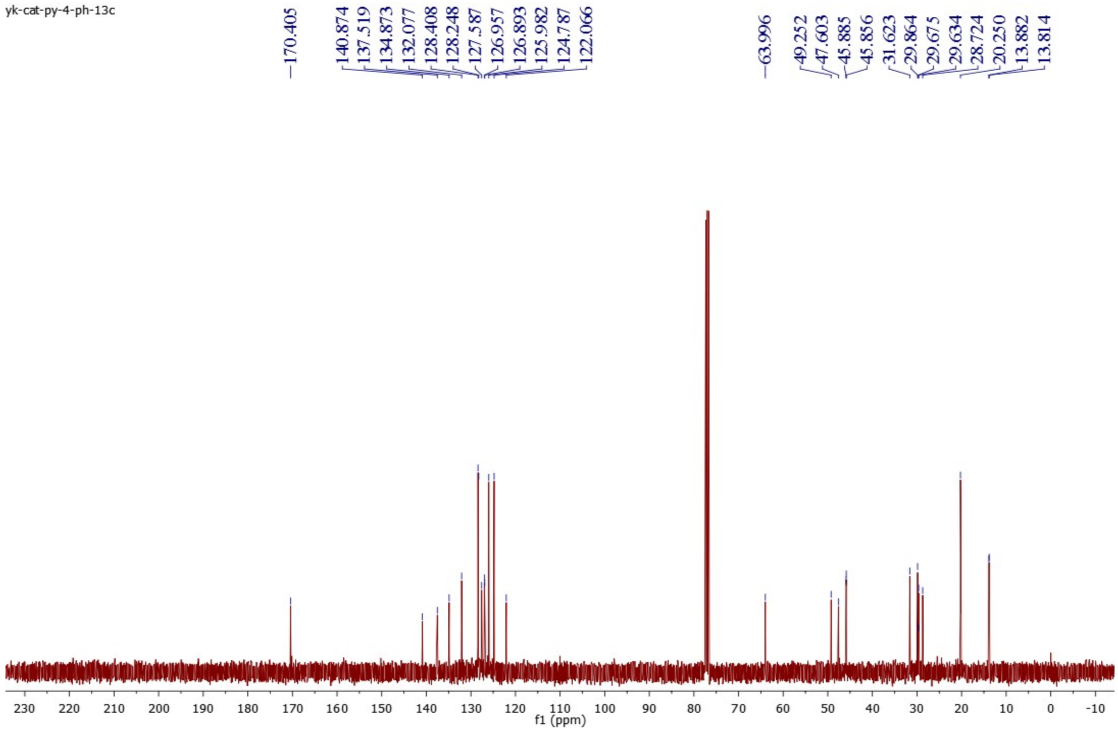
yk-cat-py-4-ph-13c
170.405
140.874
137.519
134.873
132.077
128.408
128.248
127.587
126.957
126.893
125.982
124.787
122.066
63.996
49.252
47.603
45.885
45.856
31.623
29.864
29.675
29.634
28.724
20.250
13.882
13.814
f1 (ppm)

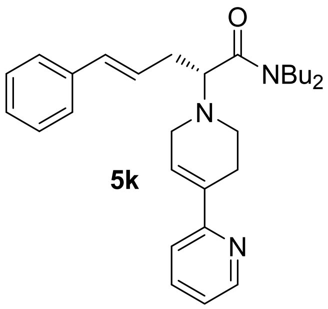
O
NBu2
N
N

5k

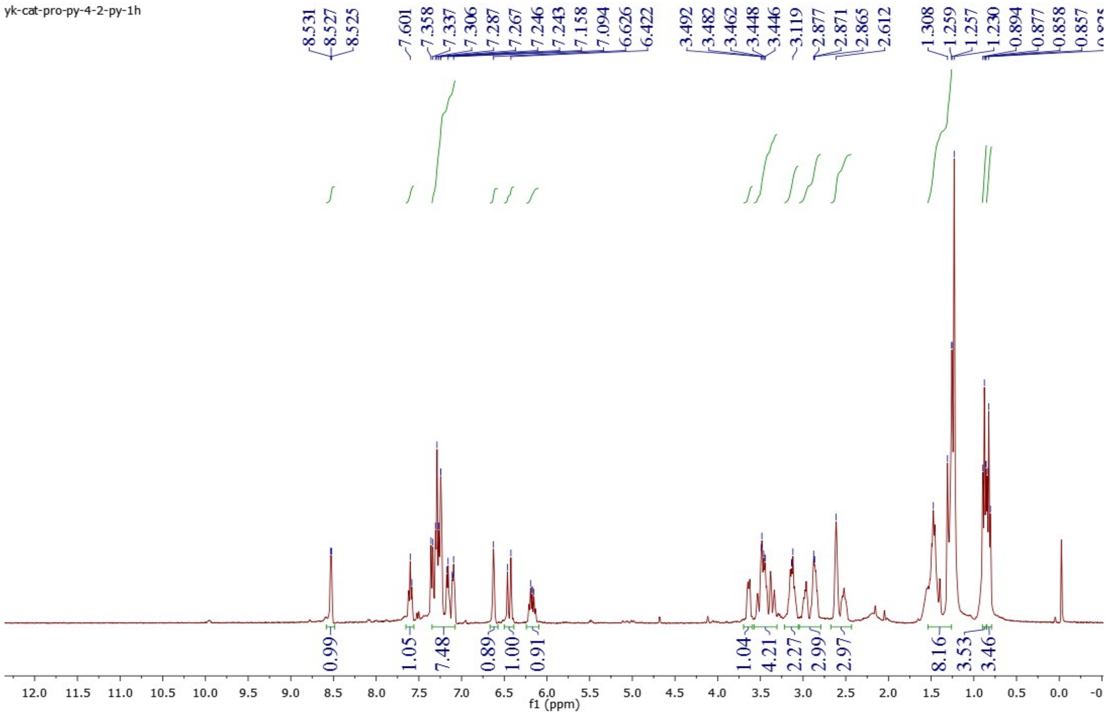
yk-cat-pro-py-4-2-py-1h
8.531
8.527
8.525
7.601
7.358
7.337
7.306
7.287
7.267
7.246
7.243
7.158
7.094
6.626
6.422
3.492
3.482
3.462
3.448
3.446
3.119
2.877
2.871
2.865
2.612
1.308
1.259
1.257
1.230
0.894
0.877
0.858
0.857
0.99
1.05
7.48
0.89
1.00
0.91
1.04
4.21
2.27
2.99
2.97
8.16
3.53
3.46
f1 (ppm)

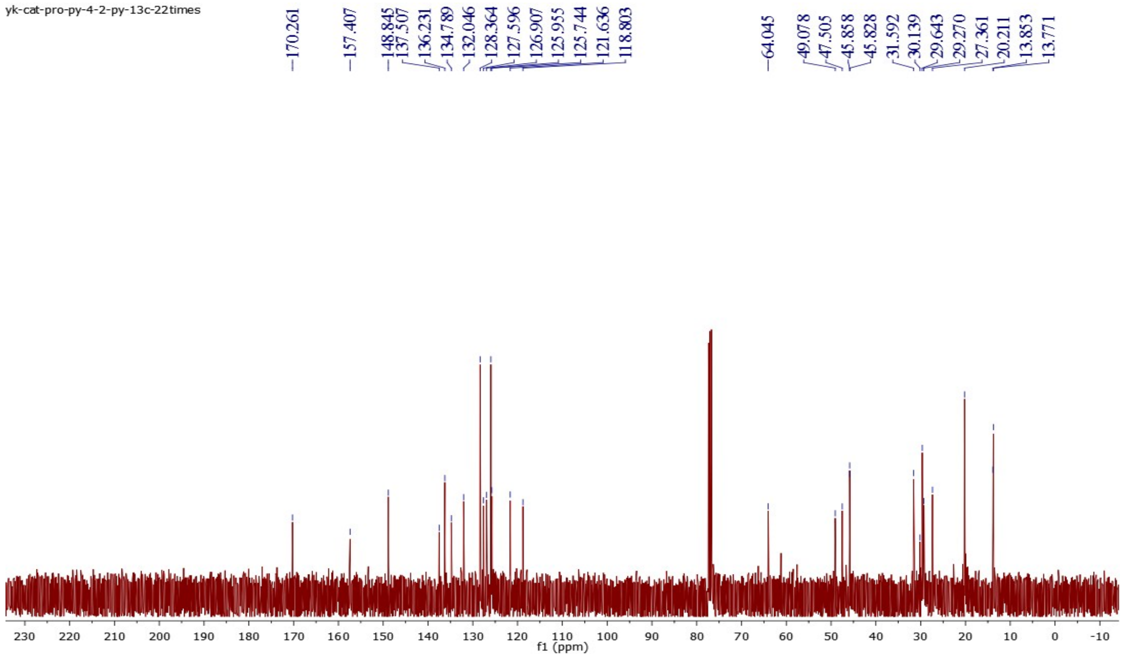
yk-cat-pro-py-4-2-py-13c-22times
170.261
157.407
148.845
137.507
136.231
134.789
132.046
128.364
127.596
126.907
125.955
125.744
121.636
118.803
64.045
49.078
47.505
45.858
45.828
31.592
30.139
29.643
29.270
27.361
20.211
13.853
13.771
f1 (ppm)

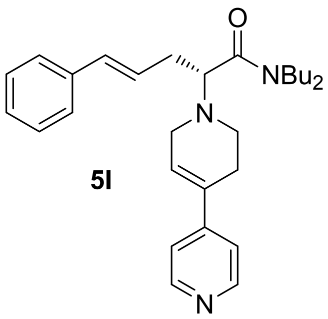

NBu2
N
N

5l

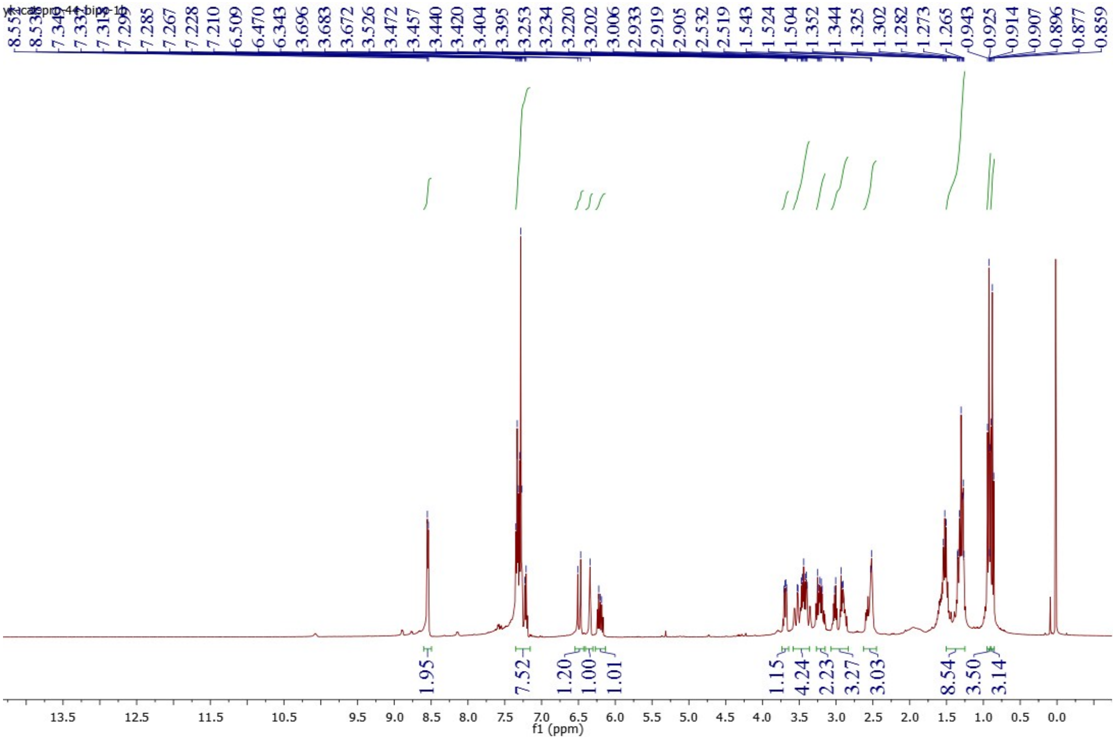

7.285
7.267
7.228
7.210
6.509
6.470
6.343
3.696
3.683
3.672
3.526
3.472
3.457
3.440
3.420
3.404
3.395
3.253
3.234
3.220
3.202
3.006
2.933
2.919
2.905
2.532
2.519
1.543
1.524
1.504
1.352
1.344
1.325
1.302
1.282
1.273
1.265
0.943
0.925
0.914
0.907
0.896
0.877
0.859
1.95
7.52
1.20
1.00
1.01
1.15
4.24
2.23
3.27
3.03
8.54
3.50
3.14
f1 (ppm)

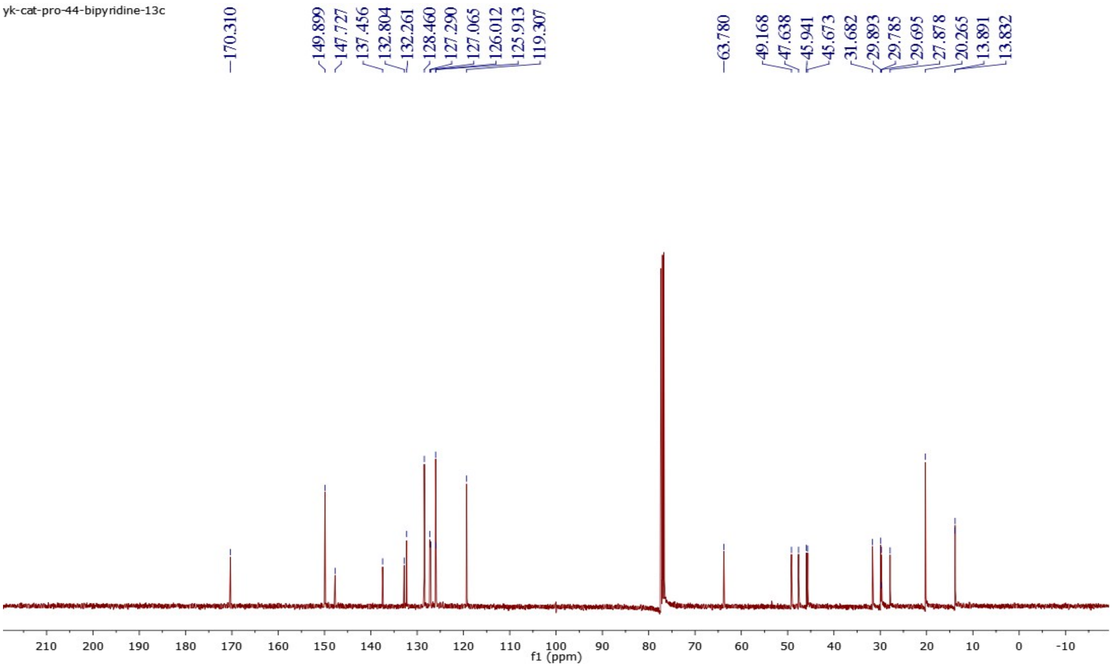

yk-cat-pro-44-bipyridine-13c
170.310
149.899
147.727
137.456
132.804
132.261
128.460
127.290
127.065
126.012
125.913
119.307
63.780
49.168
47.638
45.941
45.673
31.682
29.893
29.785
29.695
27.878
20.265
13.891
13.832
f1 (ppm)

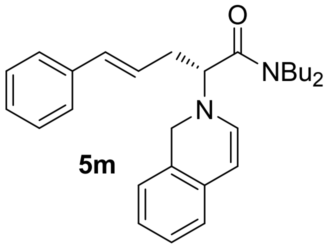

5m

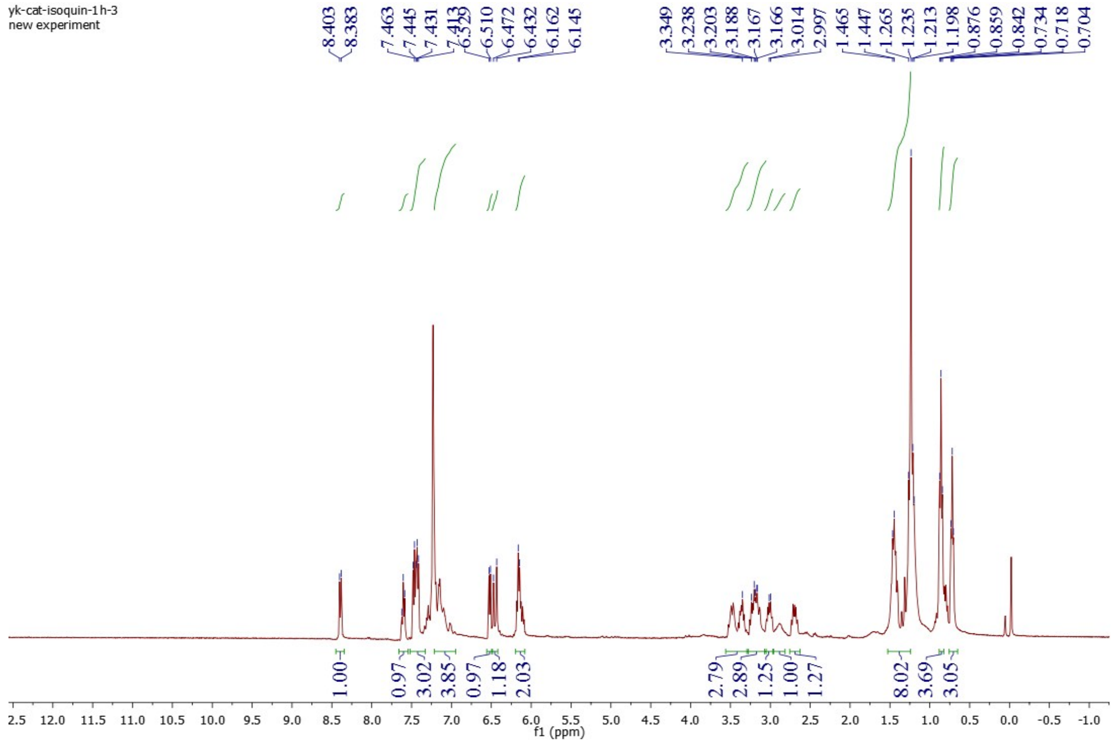
yk-cat-isoquin-1h-3
new experiment
f1 (ppm)

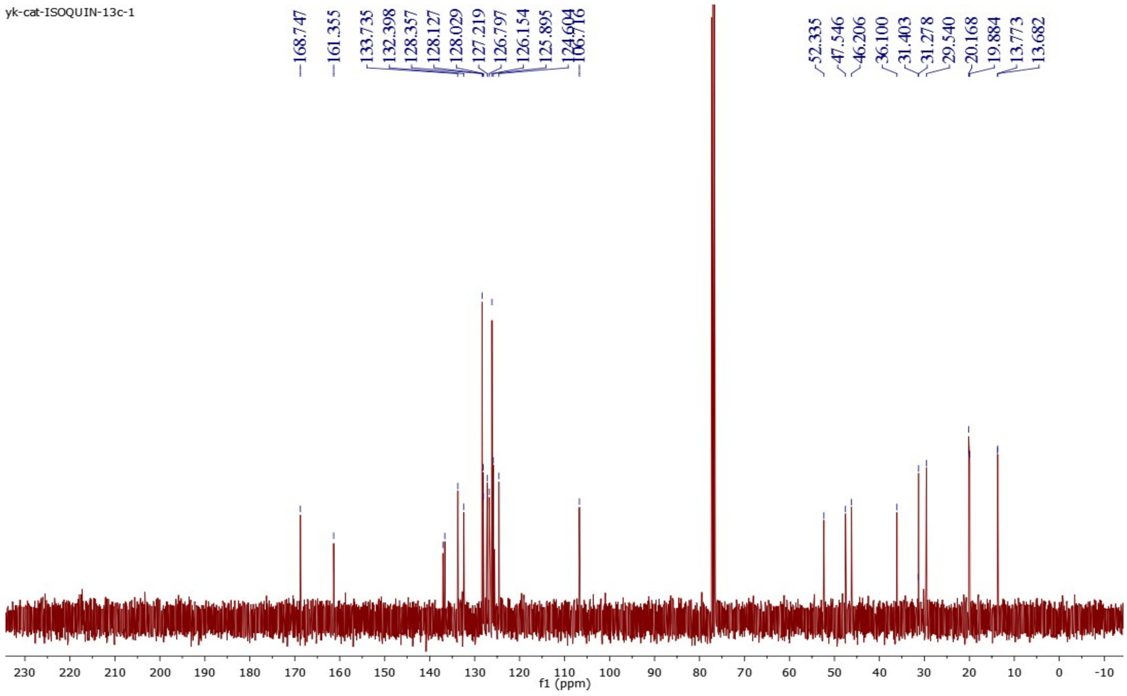
yk-cat-ISOQUIN-13c-1
f1 (ppm)

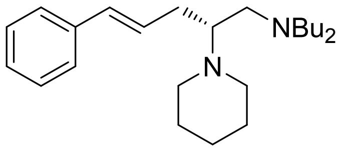
NBu2
N

6

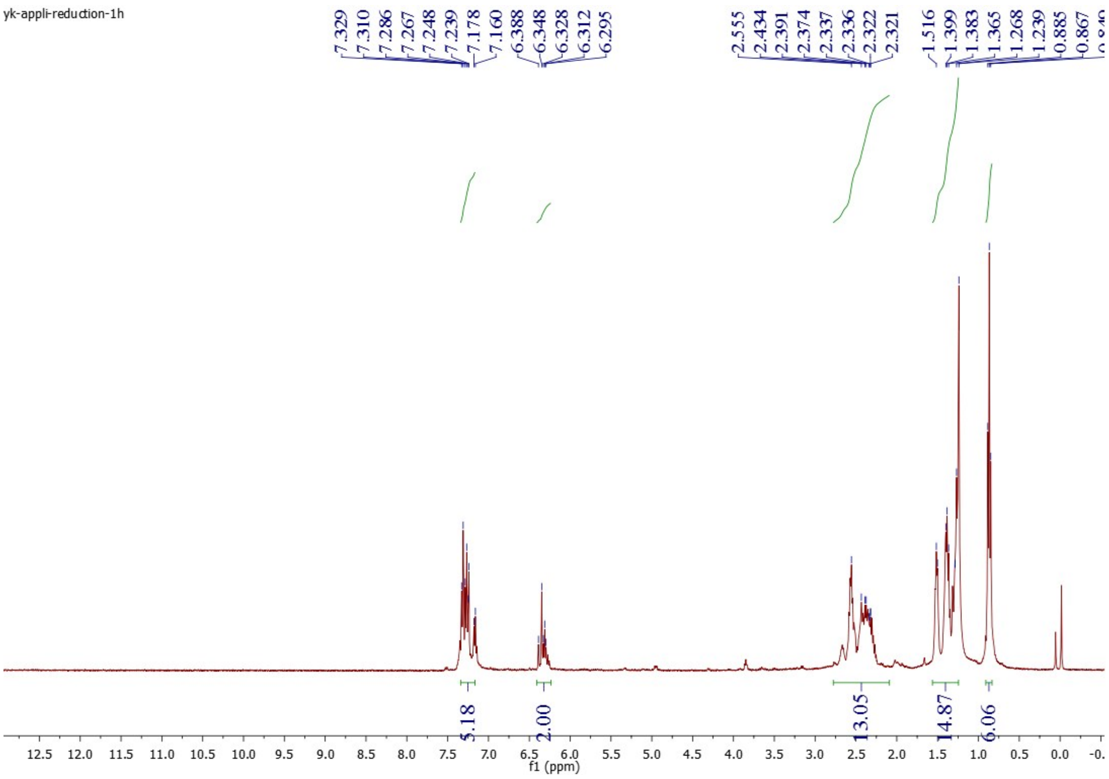
yk-appli-reduction-1h
7.329
7.310
7.286
7.267
7.248
7.239
7.178
7.160
6.388
6.348
6.328
6.312
6.295
2.555
2.434
2.391
2.374
2.337
2.336
2.322
2.321
1.516
1.399
1.383
1.365
1.268
1.239
0.885
0.867
5.18
2.00
13.05
14.87
6.06
f1 (ppm)

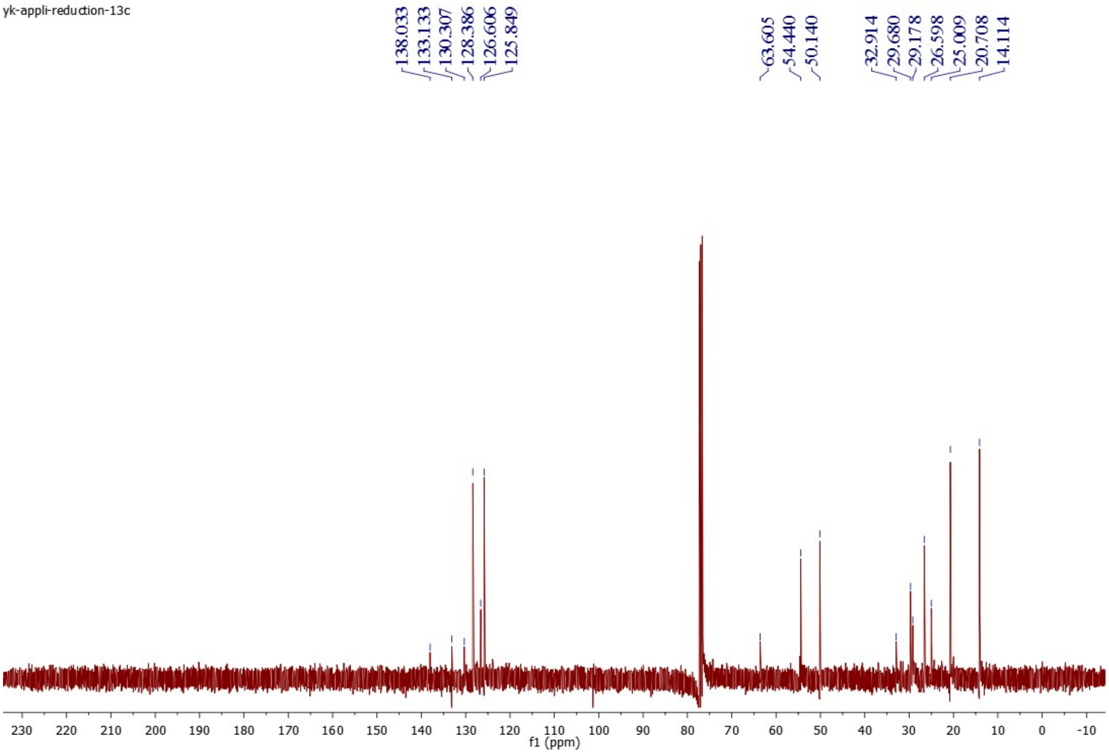
yk-appli-reduction-13c
138.033
133.133
130.307
128.386
126.606
125.849
63.605
54.440
50.140
32.914
29.680
29.178
26.598
25.009
20.708
14.114
f1 (ppm)

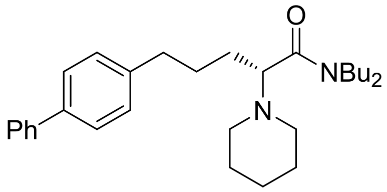
O
NBu2
N
Ph

7

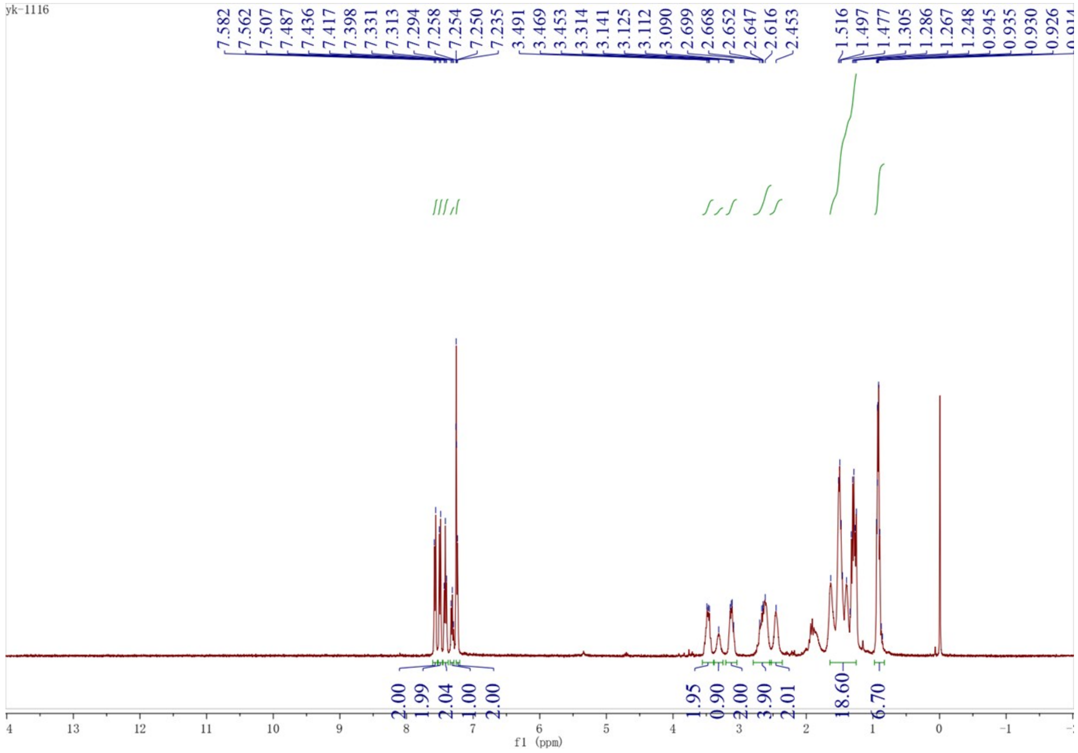
yk-1116
7.582
7.562
7.507
7.487
7.436
7.417
7.398
7.331
7.313
7.294
7.258
7.254
7.250
7.235
3.491
3.469
3.453
3.314
3.141
3.125
3.112
3.090
2.699
2.668
2.652
2.647
2.616
2.453
1.516
1.497
1.477
1.305
1.286
1.267
1.248
0.945
0.935
0.930
0.926
2.00
1.99
2.04
1.00
2.00
1.95
0.90
2.00
3.90
2.01
18.60
6.70
f1 (ppm)

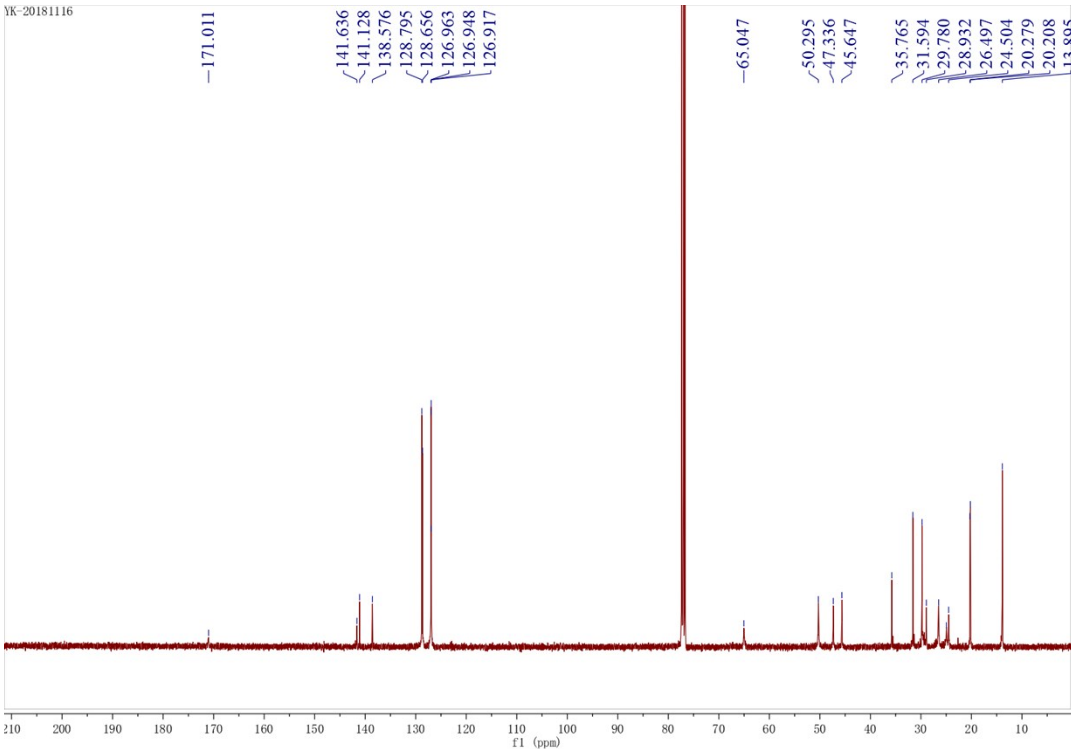
YK-20181116
171.011
141.636
141.128
138.576
128.795
128.656
126.963
126.948
126.917
65.047
50.295
47.336
45.647
35.765
31.594
29.780
28.932
26.497
24.504
20.279
20.208
f1 (ppm)

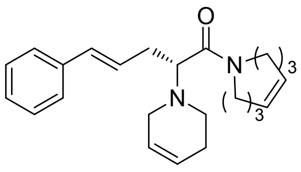

O
N
3
N
3

8

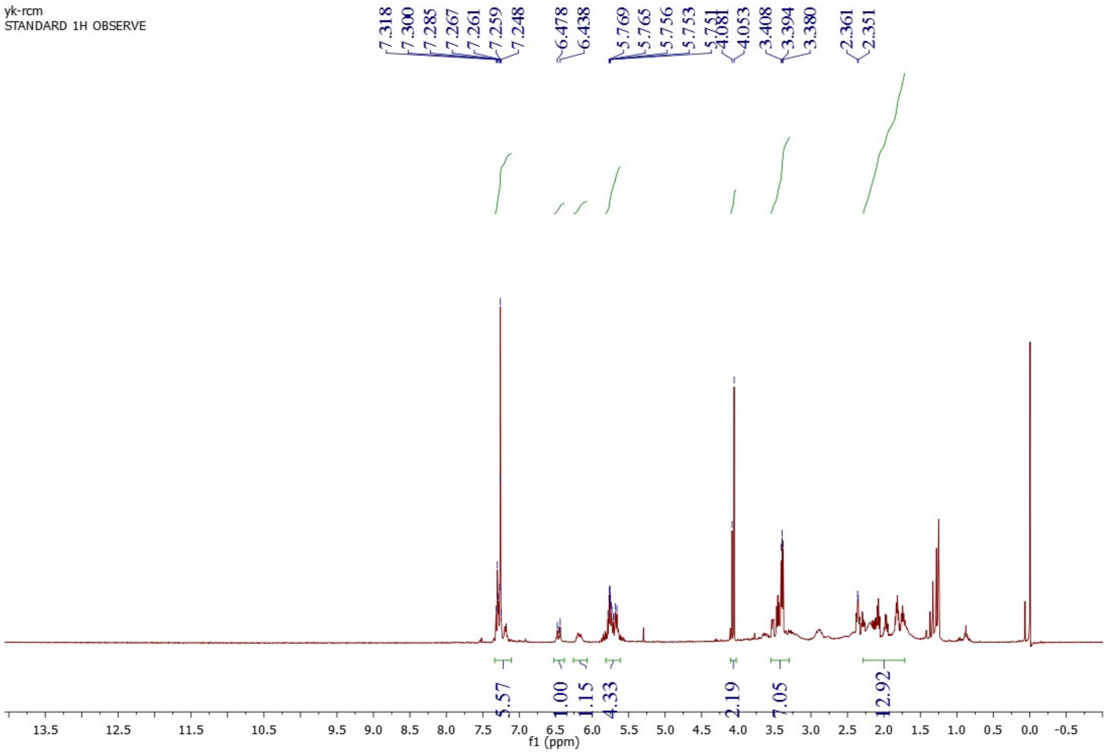

yk-rcm
STANDARD 1H OBSERVE
7.318
7.300
7.285
7.267
7.261
7.259
7.248
6.478
6.438
5.769
5.765
5.756
5.753
5.751
4.081
4.053
3.408
3.394
3.380
2.361
2.351
5.57
1.00
1.15
4.33
2.19
7.05
12.92
13.5
12.5
11.5
10.5
9.5
9.0
8.5
8.0
7.5
7.0
6.5
6.0
5.5
5.0
4.5
4.0
3.5
3.0
2.5
2.0
1.5
1.0
0.5
0.0
-0.5
f1 (ppm)

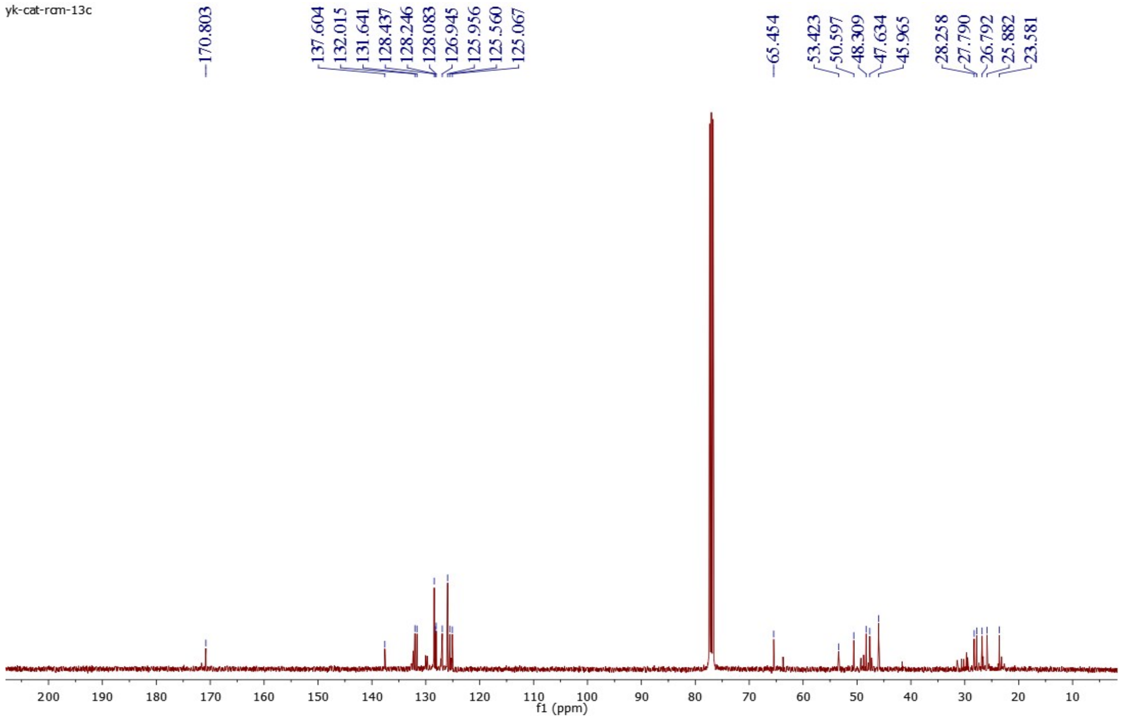

yk-cat-rcm-13c
170.803
137.604
132.015
131.641
128.437
128.246
128.083
126.945
125.956
125.560
125.067
65.454
53.423
50.597
48.309
47.634
45.965
28.258
27.790
26.792
25.882
23.581
200
190
180
170
160
150
140
130
120
110
100
90
80
70
60
50
40
30
20
10
f1 (ppm)

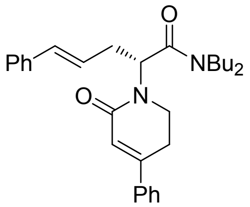
Ph
O
NBu2
O
N
Ph

9

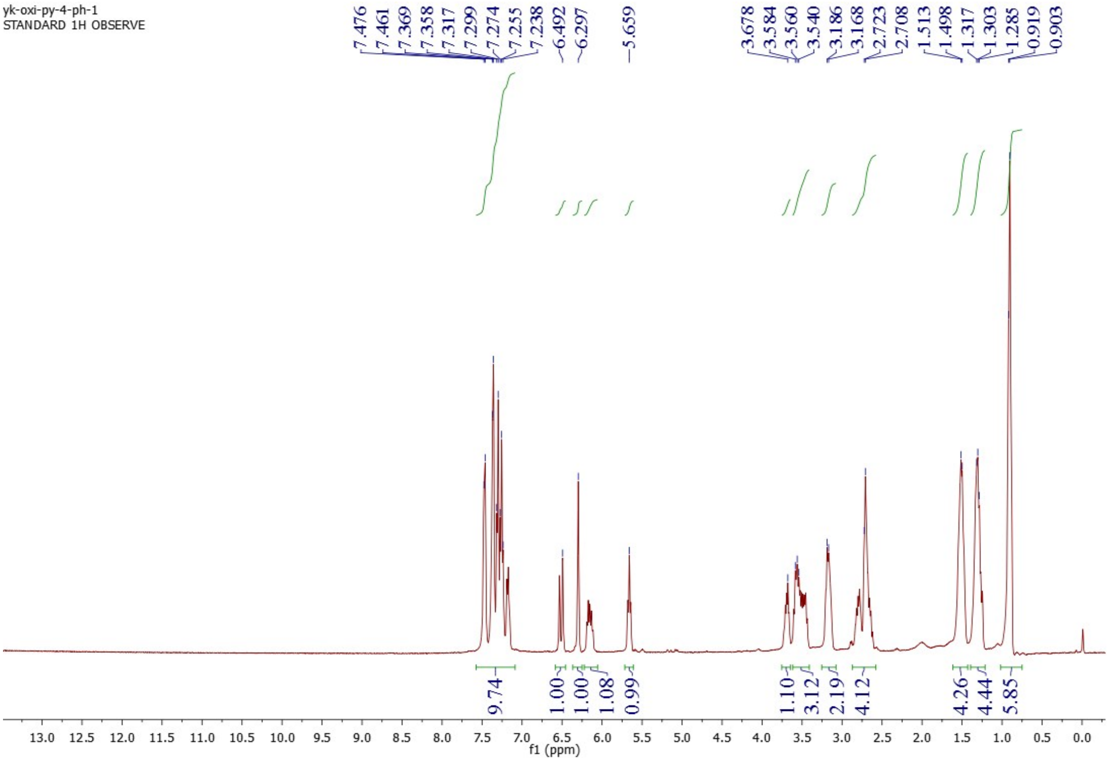
yk-oxi-py-4-ph-1
STANDARD 1H OBSERVE
7.476 7.461 7.369 7.358 7.317 7.299 7.274 7.255 7.238 6.492 6.297 5.659 3.678 3.584 3.560 3.540 3.186 3.168 2.723 2.708 1.513 1.498 1.317 1.303 1.285 0.919 0.903
9.74 1.00 1.00 1.08 0.99 1.10 3.12 2.19 4.12 4.26 4.44 5.85
f1 (ppm)

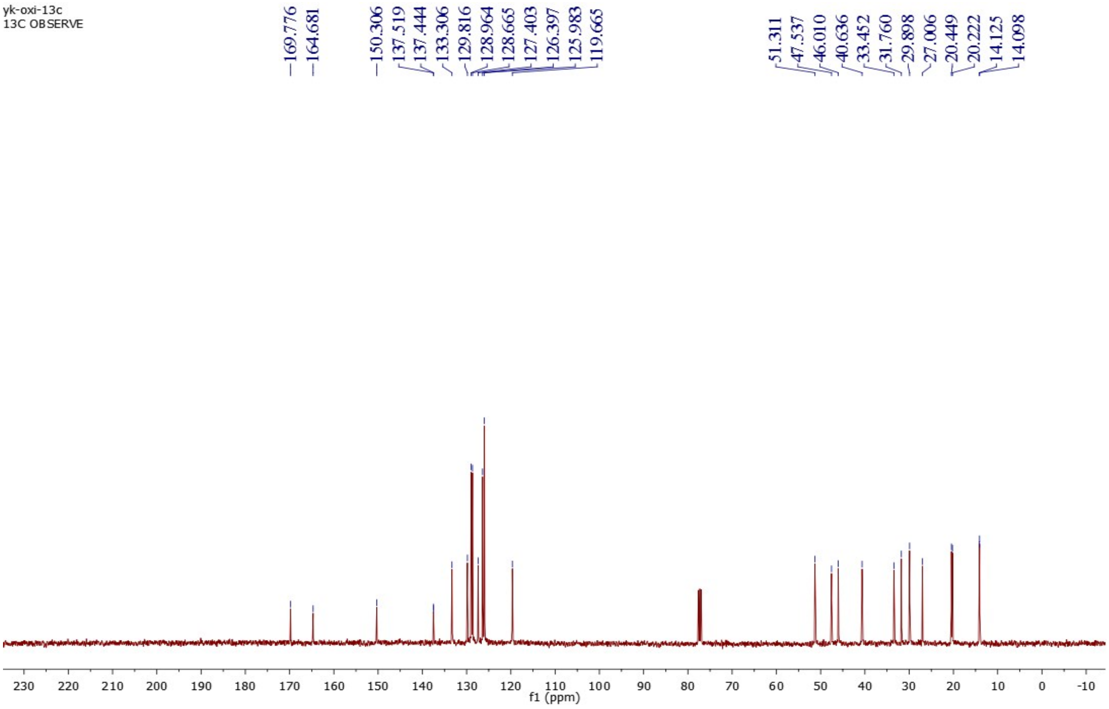
yk-oxi-13c
13C OBSERVE
169.776 164.681 150.306 137.519 137.444 133.306 129.816 128.964 128.665 127.403 126.397 125.983 119.665 51.311 47.537 46.010 40.636 33.452 31.760 29.898 27.006 20.449 20.222 14.125 14.098
f1 (ppm)

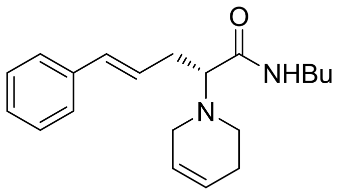
O
NHBu
N

10

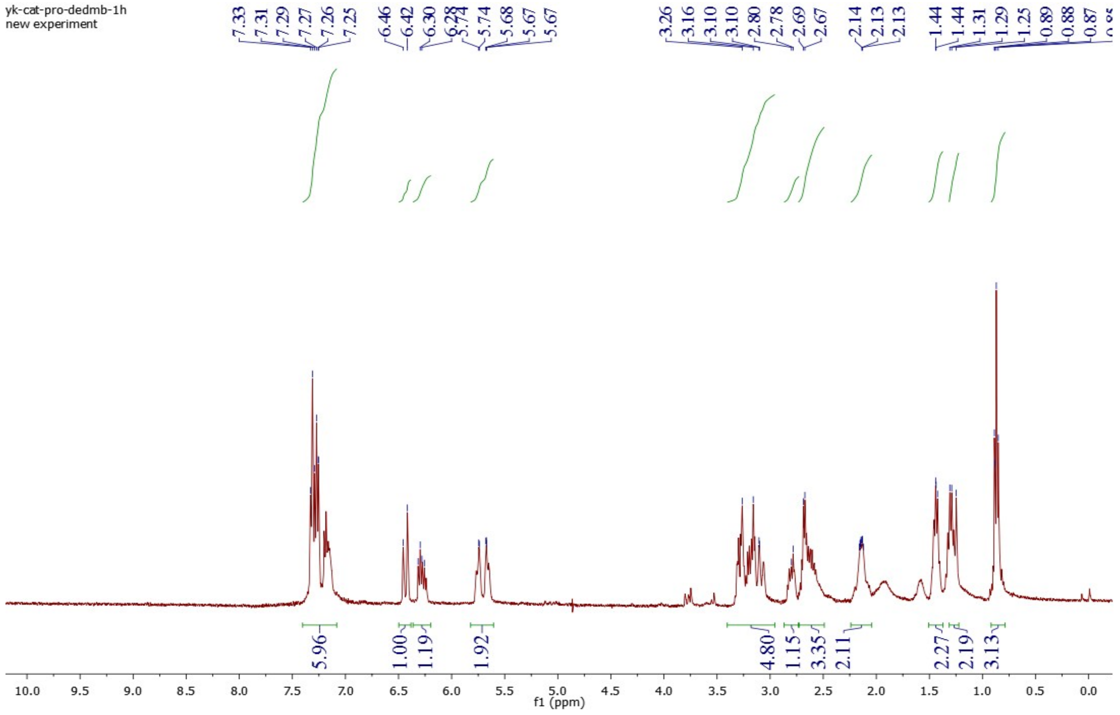
yk-cat-pro-dedmb-1h
new experiment
7.33 7.31 7.29 7.27 7.26 7.25 6.46 6.42 6.30 5.74 5.68 5.67 5.67
3.26 3.16 3.10 3.10 2.80 2.78 2.69 2.67 2.14 2.13 2.13 1.44 1.44 1.31 1.29 1.25 0.89 0.88 0.87
5.96 1.00 1.19 1.92 4.80 1.15 3.35 2.11 2.27 2.19 3.13
f1 (ppm)

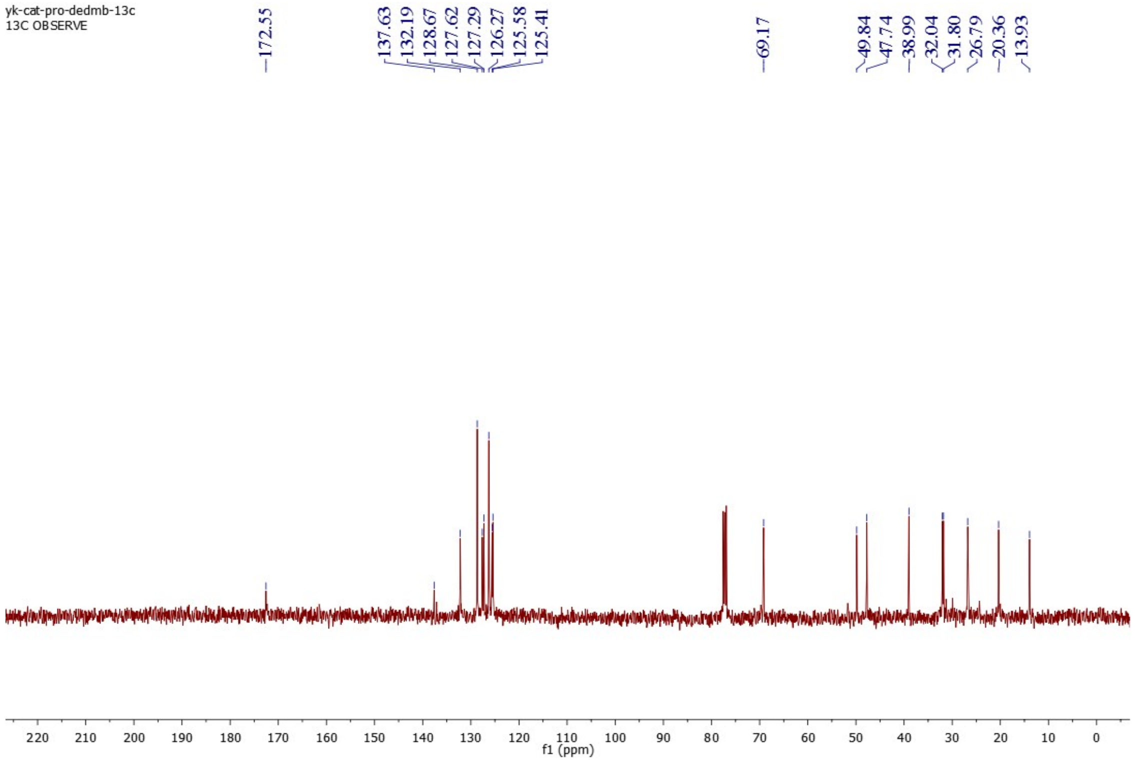
yk-cat-pro-dedmb-13c
13C OBSERVE
172.55
137.63 132.19 128.67 127.62 127.29 126.27 125.58 125.41
69.17
49.84 47.74 38.99 32.04 31.80 26.79 20.36 13.93
f1 (ppm)

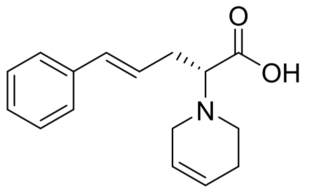
O
OH
N

11

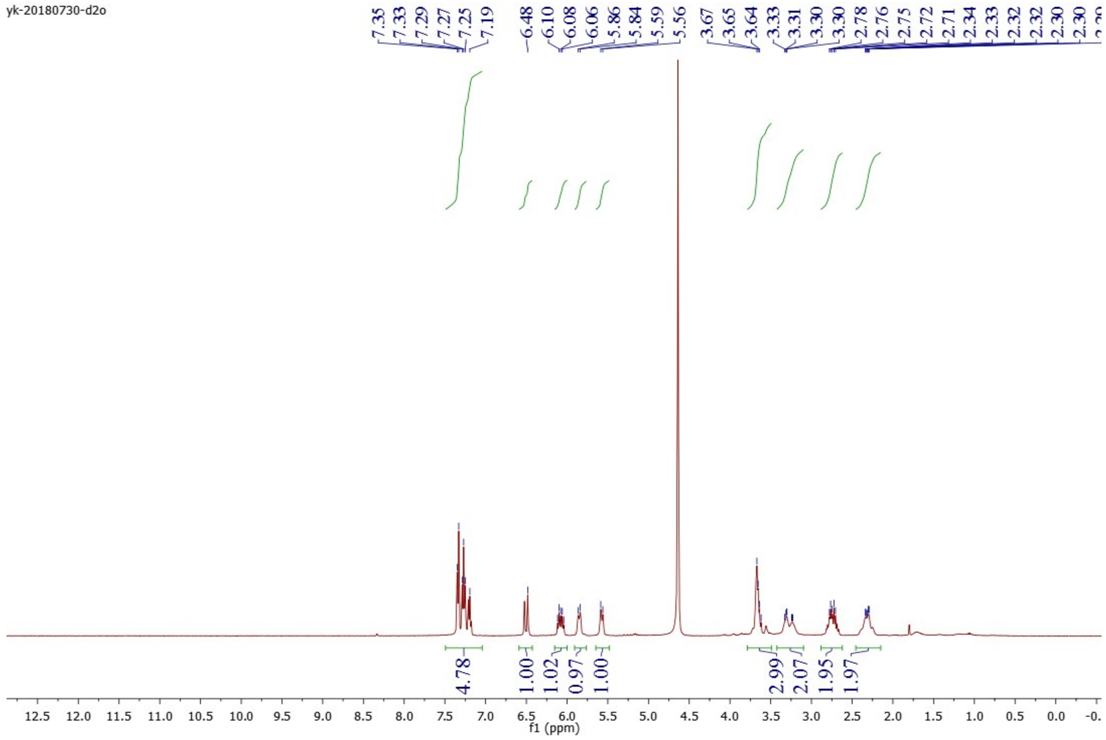
yk-20180730-d2o
7.35
7.33
7.29
7.27
7.25
7.19
6.48
6.10
6.08
6.06
5.86
5.84
5.59
5.56
3.67
3.65
3.64
3.33
3.31
3.30
3.30
2.78
2.76
2.75
2.72
2.71
2.34
2.33
2.32
2.32
2.30
2.30
4.78
1.00
1.02
0.97
1.00
2.99
2.07
1.95
1.97
f1 (ppm)

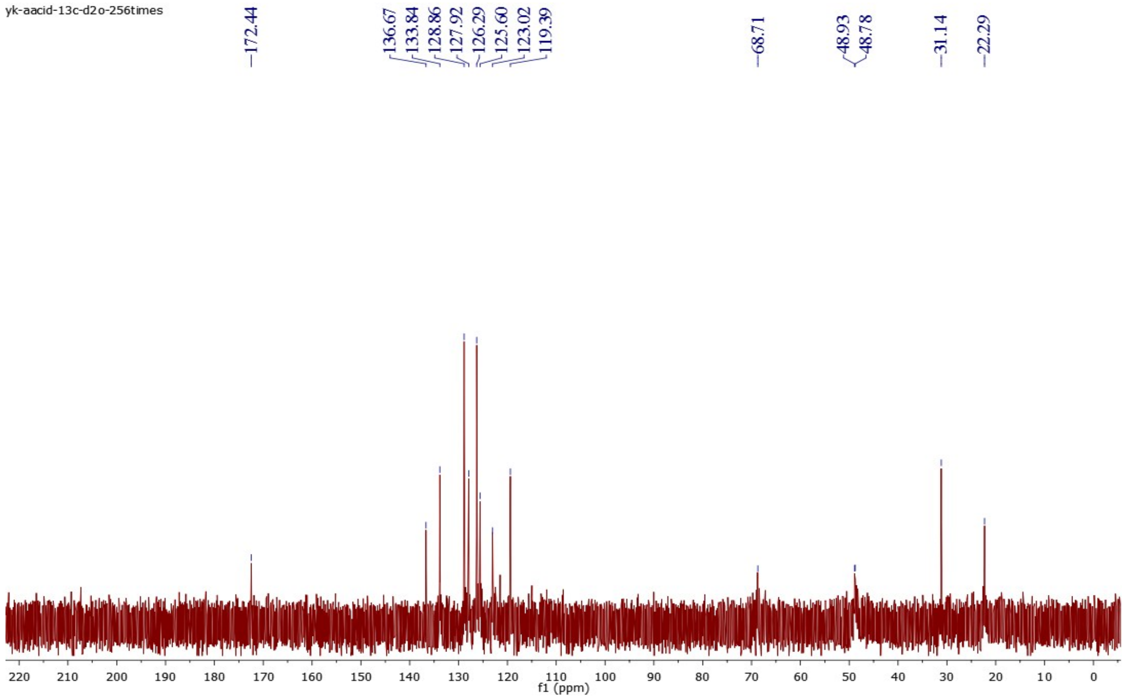
yk-aacid-13c-d2o-256times
172.44
136.67
133.84
128.86
127.92
126.29
125.60
123.02
119.39
68.71
48.93
48.78
31.14
22.29
f1 (ppm)

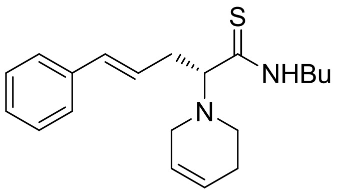
S
NHBu
N

12

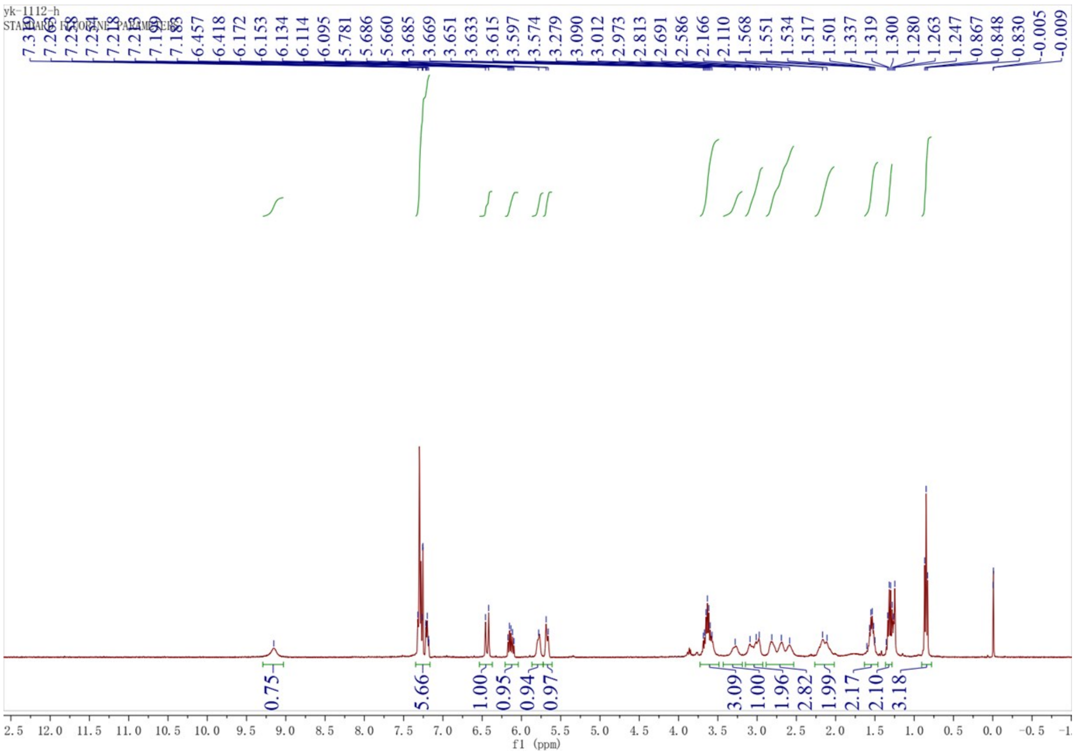
yk-1112-h
STANDARD FLUORINE PARAMETERS
6.457
6.418
6.172
6.153
6.134
6.114
6.095
5.781
5.686
5.660
3.685
3.669
3.651
3.633
3.615
3.597
3.574
3.279
3.090
3.012
2.973
2.813
2.691
2.586
2.166
2.110
1.568
1.551
1.534
1.517
1.501
1.337
1.319
1.300
1.280
1.263
1.247
0.867
0.848
0.830
-0.005
-0.009
0.75
5.66
1.00
0.95
0.94
0.97
3.09
1.00
1.96
2.82
1.99
2.17
2.10
3.18
f1 (ppm)

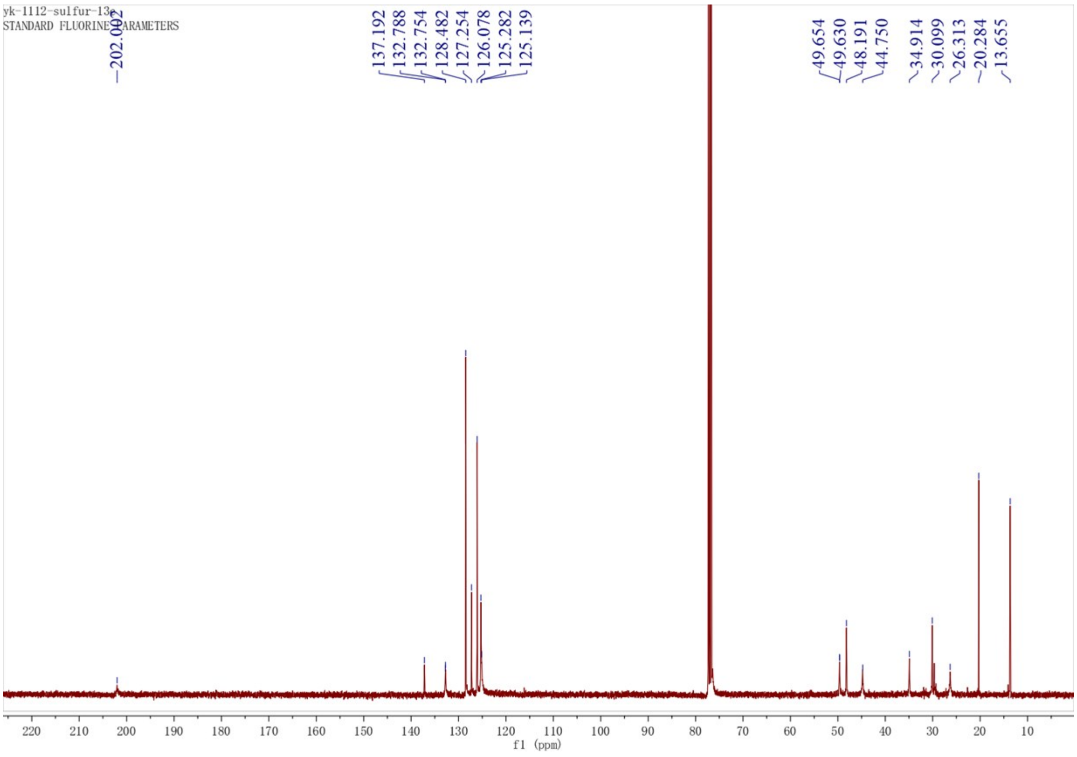
yk-1112-sulfur-13c
STANDARD FLUORINE PARAMETERS
202.002
137.192
132.788
132.754
128.482
127.254
126.078
125.282
125.139
49.654
49.630
48.191
44.750
34.914
30.099
26.313
20.284
13.655
f1 (ppm)

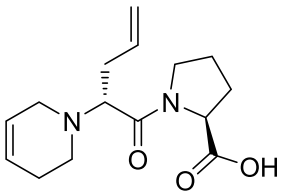

14

^{1}H NMR (DMSO-d_6) of the crude reaction mixture

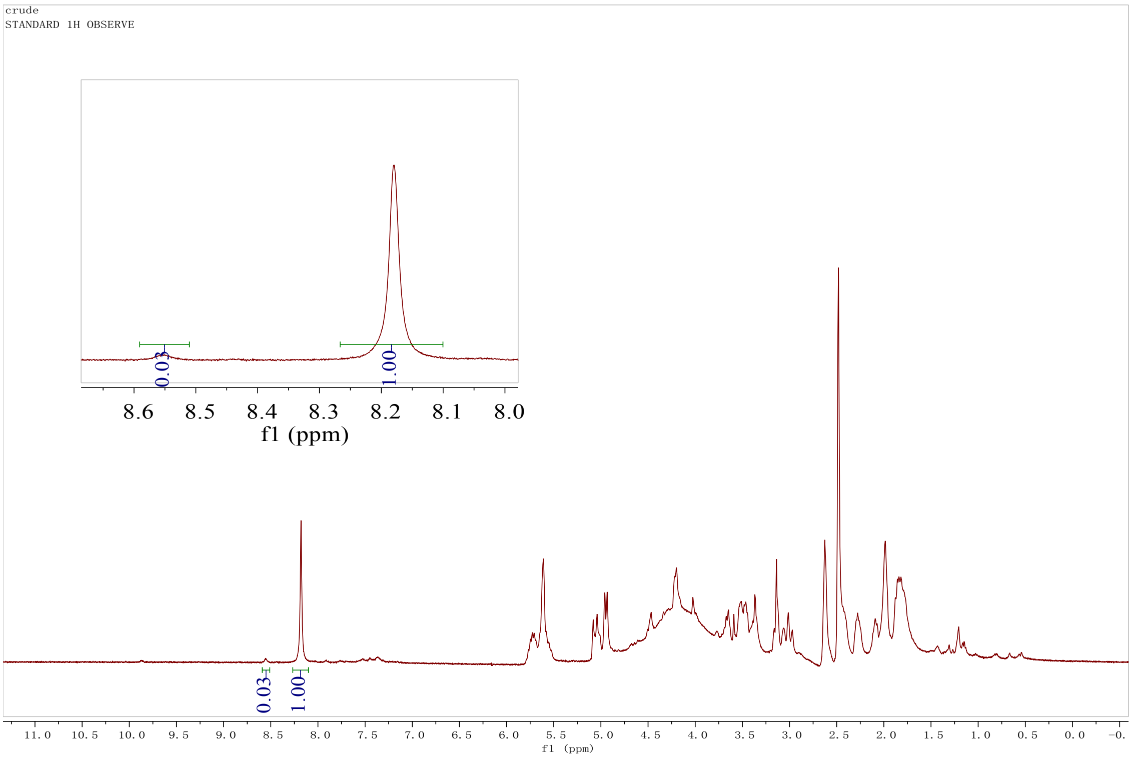

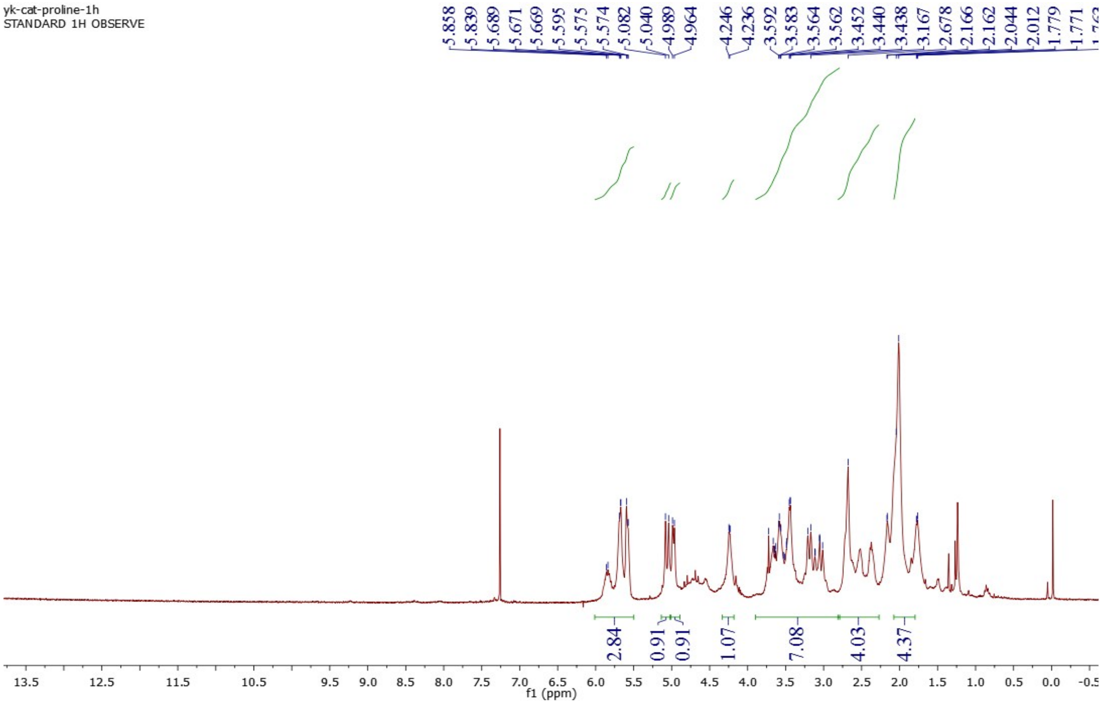

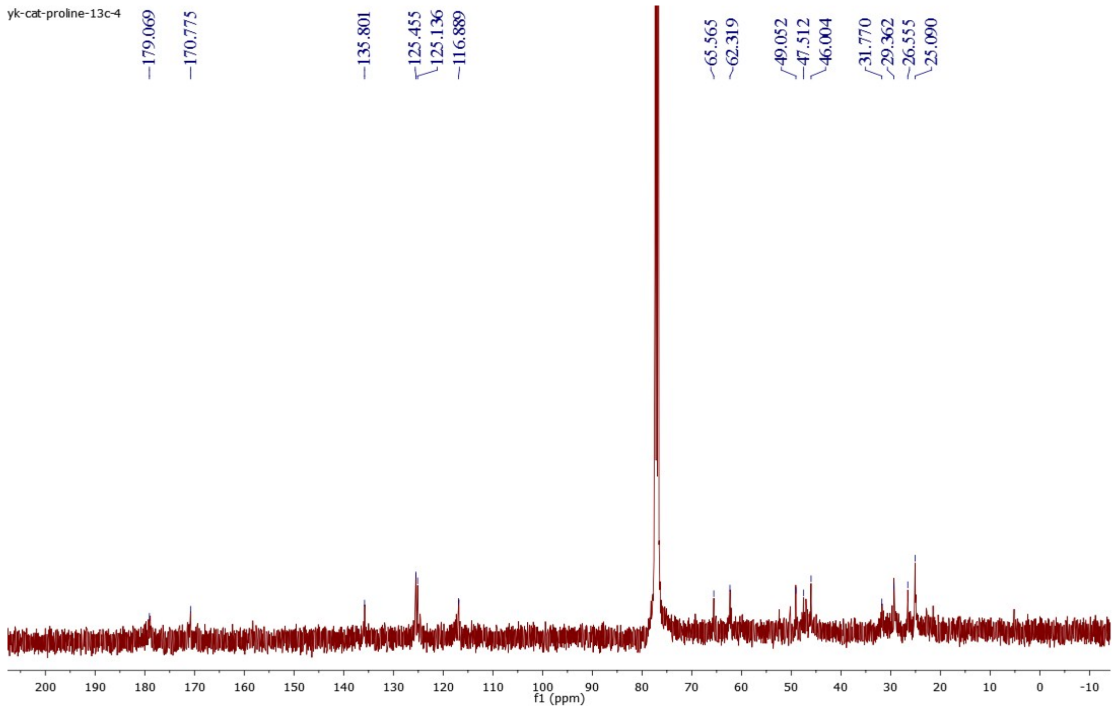
yk-cat-proline-13c-4
179.069
170.775
135.801
125.455
125.136
116.889
65.565
62.319
49.052
47.512
46.004
31.770
29.362
26.555
25.090
f1 (ppm)

9. HPLC Data

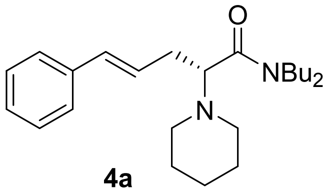

4a

Racemate:

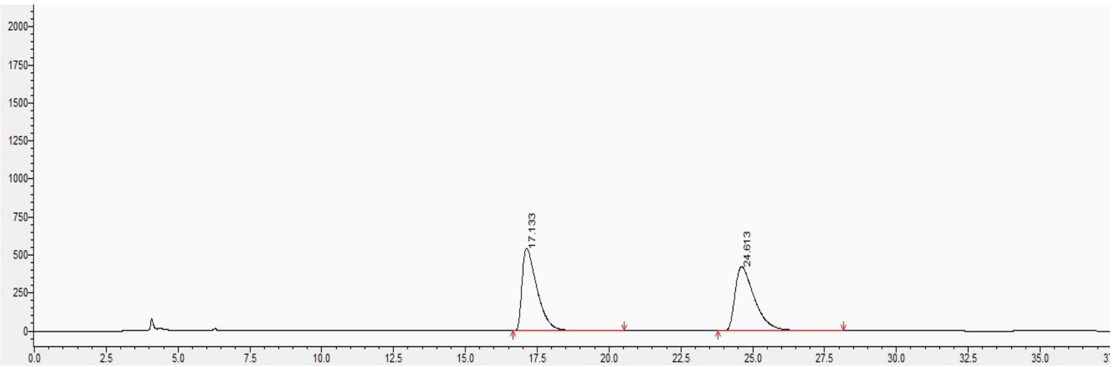

	Retention Time (min)	Area (%)
Peak 1	17.133	49.221
Peak 2	24.613	50.779

Chiral:

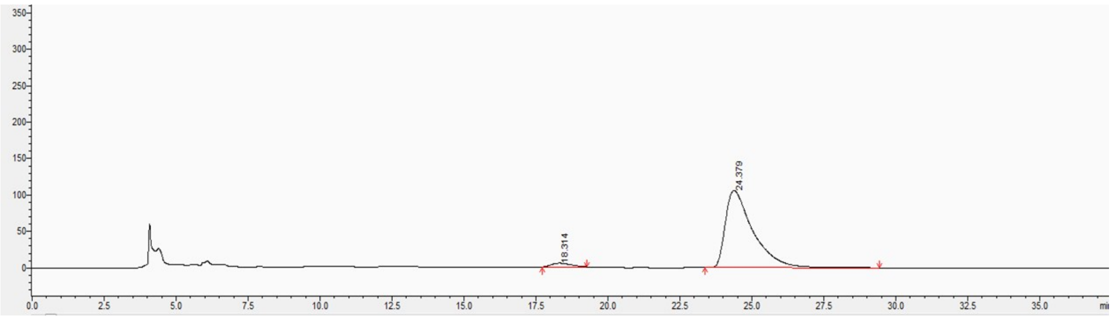

	Retention Time (min)	Area (%)	
Peak 1	18.314	2.608	**95% ee**
Peak 2	24.379	97.392	

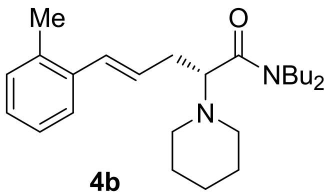

4b

88% *ee*, enantiomeric excess was determined by HPLC, Daicel Chiralpak IC-3 column, *n*-hexane/*i*-PrOH = 95/5, 0.8 mL/min, 254 nm.

Racemate:

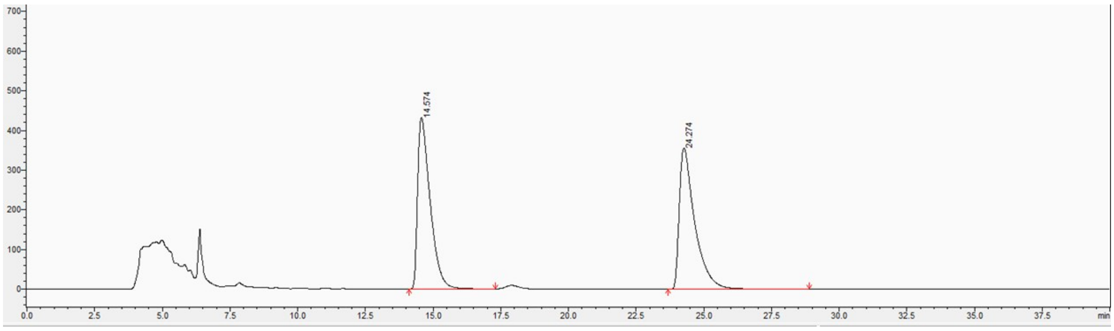

	Retention Time (min)	Area (%)
Peak 1	14.574	49.576
Peak 2	24.274	50.424

Chiral:

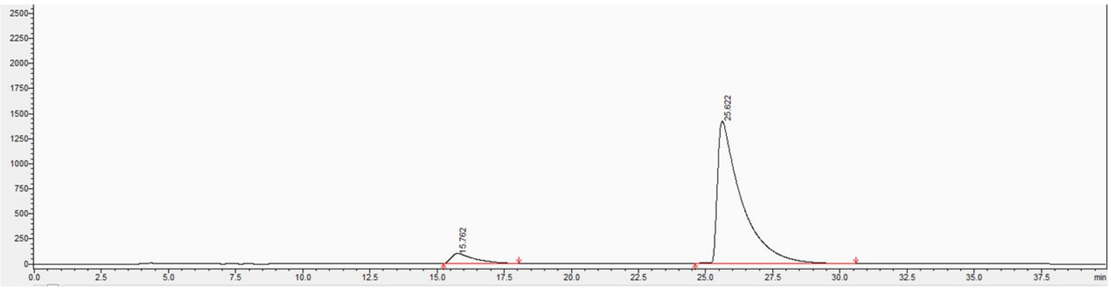

	Retention Time (min)	Area (%)	
Peak 1	14.969	6.242	**88% ee**
Peak 2	25.141	93.758	

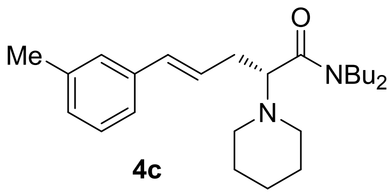

4c

88% *ee*, enantiomeric excess was determined by HPLC, Daicel Chiralpak IC-3 column, *n*-hexane/*i*-PrOH = 95/5, 0.8 mL/min, 254 nm.

Racemate:

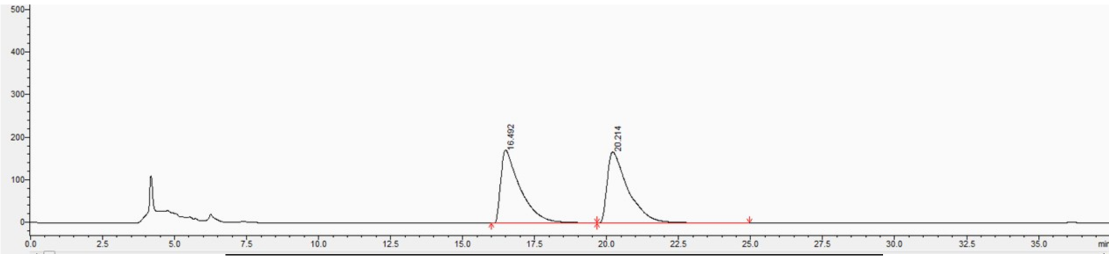

	Retention Time (min)	Area (%)
Peak 1	16.492	48.946
Peak 2	20.214	51.054

Chiral:

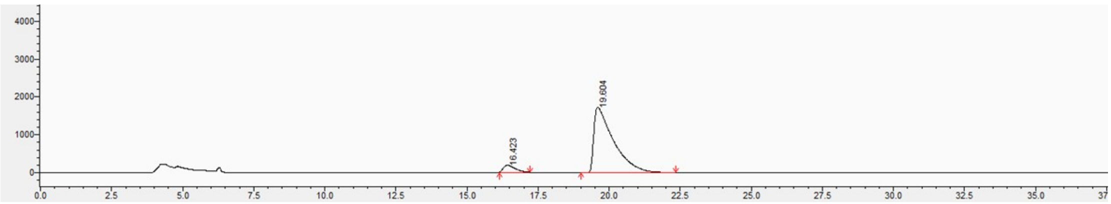

	Retention Time (min)	Area (%)	
Peak 1	16.423	6.119	**88% ee**
Peak 2	19.604	93.881	

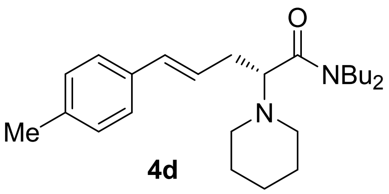

4d

92% *ee*, enantiomeric excess was determined by HPLC, Daicel Chiralpak IC-3 column, *n*-hexane/*i*-PrOH = 95/5, 0.8 mL/min, 254 nm.

Racemate:

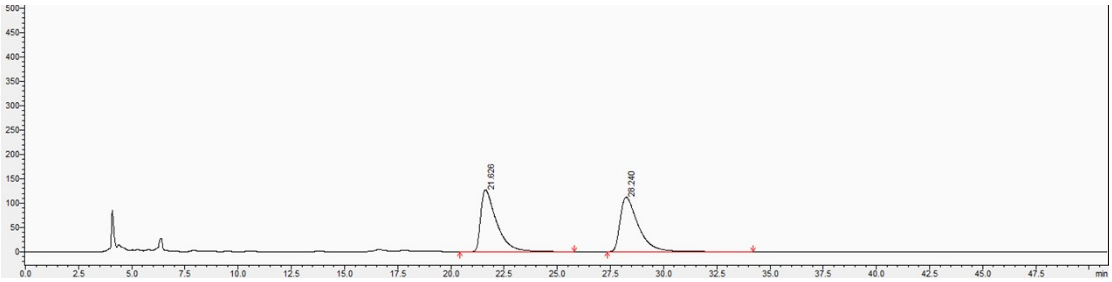

	Retention Time (min)	Area (%)
Peak 1	21.626	49.888
Peak 2	28.240	50.112

Chiral:

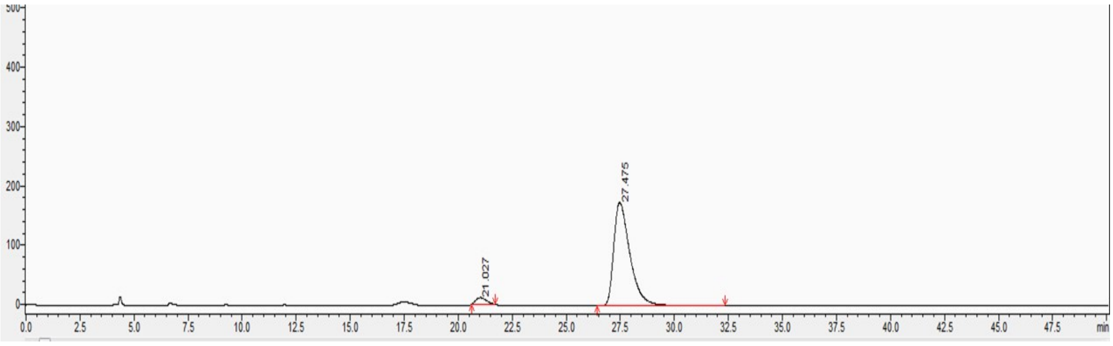

	Retention Time (min)	Area (%)	
Peak 1	21.027	4.121	**92% ee**
Peak 2	27.475	95.879	

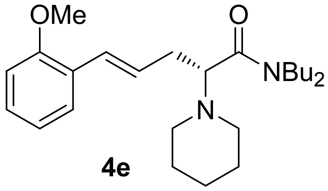

4e

82% *ee*, enantiomeric excess was determined by HPLC, Daicel Chiralpak IC-3 column, *n*-hexane/*i*-PrOH = 85/15, 0.8 mL/min, 254 nm.

Racemate:

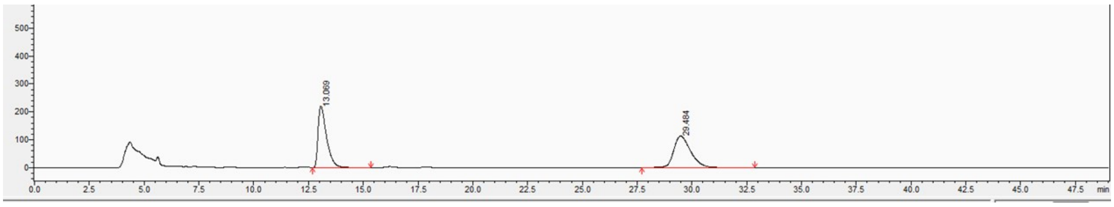

	Retention Time (min)	Area (%)
Peak 1	13.069	49.216
Peak 2	29.484	50.784

Chiral:

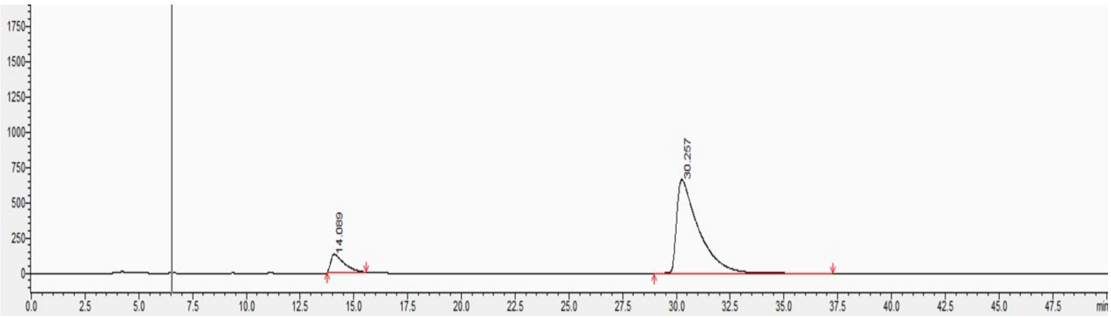

	Retention Time (min)	Area (%)	
Peak 1	14.089	8.902	**82% ee**
Peak 2	30.257	91.098	

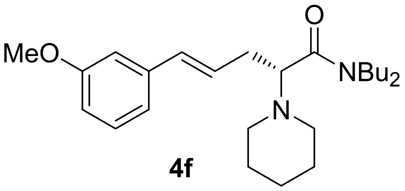

4f

87% *ee*, enantiomeric excess was determined by HPLC, Daicel Chiralpak IC-3 column, *n*-hexane/*i*-PrOH = 90/10, 0.8 mL/min, 254 nm.

Racemate:

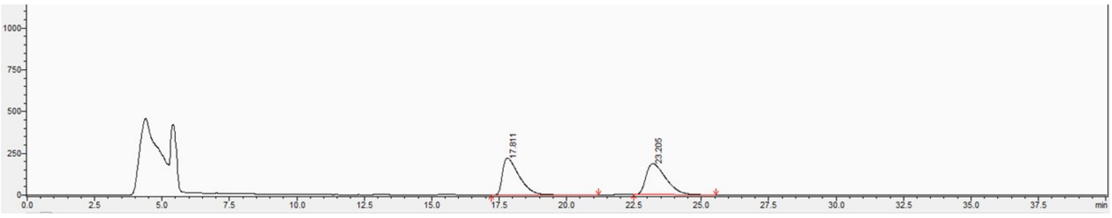

	Retention Time (min)	Area (%)
Peak 1	17.811	50.137
Peak 2	23.205	49.863

Chiral:

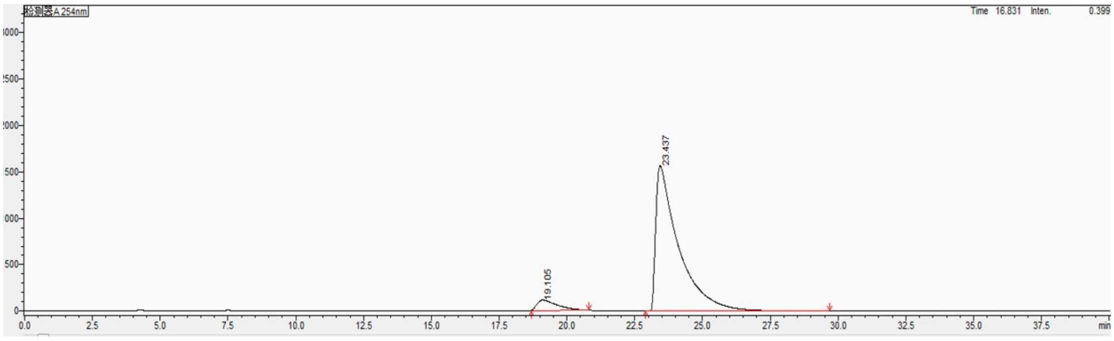

	Retention Time (min)	Area (%)	
Peak 1	19.105	6.264	**87% ee**
Peak 2	23.437	93.736	

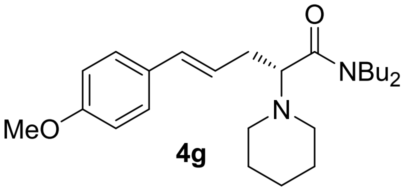

4g

92% *ee*, enantiomeric excess was determined by HPLC, Daicel Chiralpak IC-3 column, *n*-hexane/*i*-PrOH = 90/10, 0.8 mL/min, 254 nm.

Racemate:

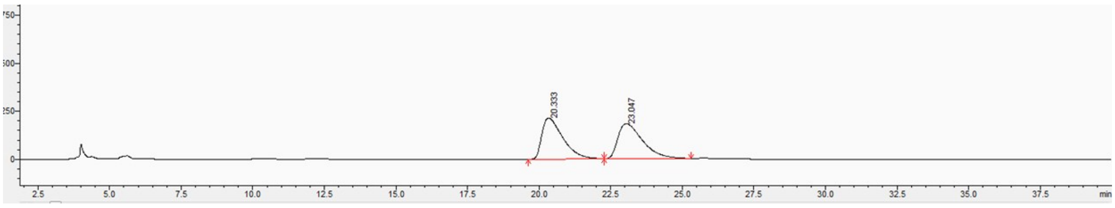

	Retention Time (min)	Area (%)
Peak 1	20.333	49.993
Peak 2	23.047	50.007

Chiral:

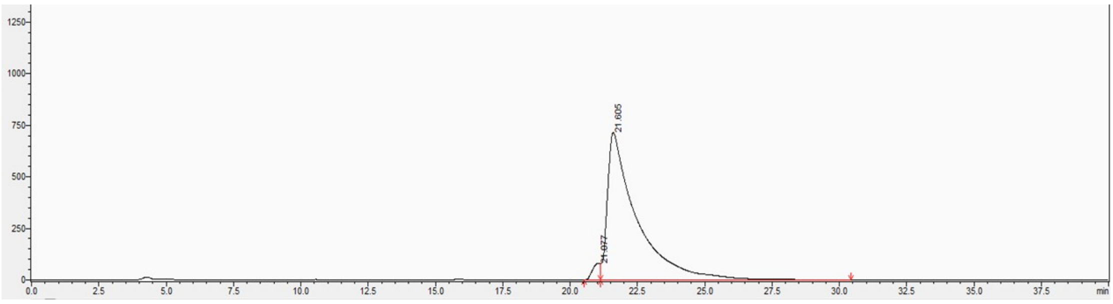

	Retention Time (min)	Area (%)	
Peak 1	21.077	3.879	**92% ee**
Peak 2	21.605	96.121	

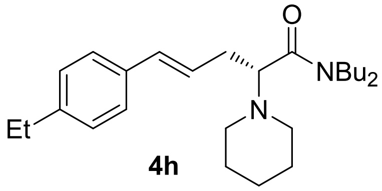

4h

91% *ee*, enantiomeric excess was determined by HPLC, Daicel Chiralpak IC-3 column, *n*-hexane/*i*-PrOH = 95/5, 0.8 mL/min, 254 nm.

Racemate:

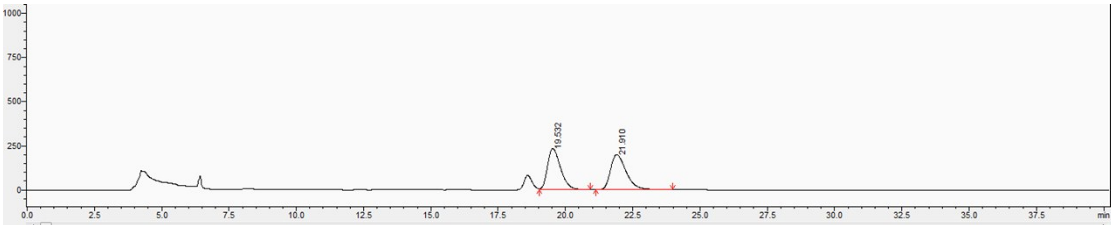

	Retention Time (min)	Area (%)
Peak 1	19.532	49.256
Peak 2	21.910	50.744

Chiral:

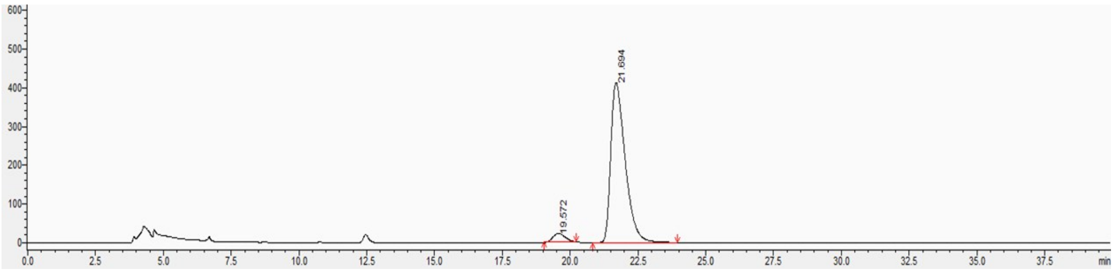

	Retention Time (min)	Area (%)	
Peak 1	19.572	4.262	**91% ee**
Peak 2	21.694	95.738	

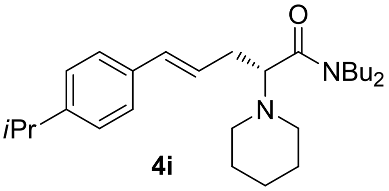

4i

90% *ee*, enantiomeric excess was determined by HPLC, Daicel Chiralpak IC-3 column, *n*-hexane/*i*-PrOH = 95/5, 0.8 mL/min, 254 nm.

Racemate:

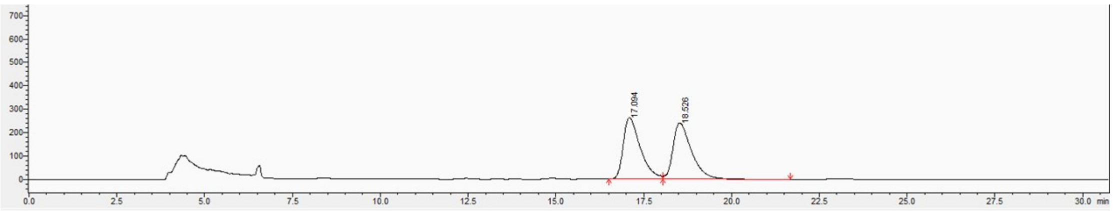

	Retention Time (min)	Area (%)
Peak 1	17.094	50.126
Peak 2	18.526	49.874

Chiral:

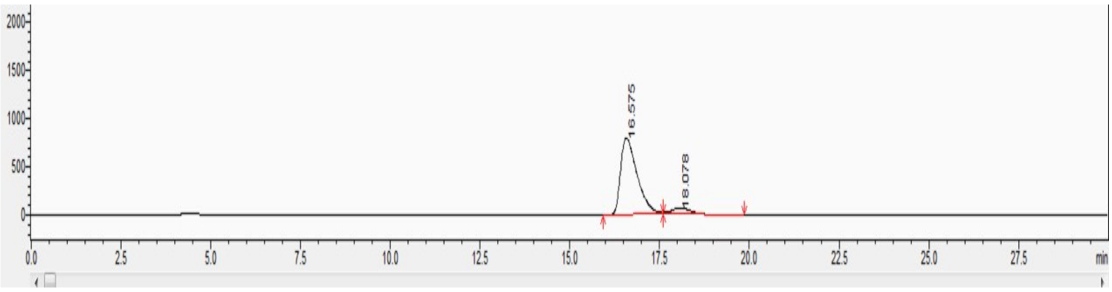

	Retention Time (min)	Area (%)	
Peak 1	16.575	94.809	**90% ee**
Peak 2	18.078	5.191	

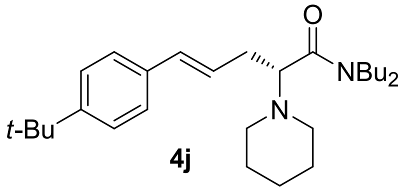

4j

87% *ee*, enantiomeric excess was determined by HPLC, Daicel Chiralpak IC-3 column, *n*-hexane/*i*-PrOH = 95/5, 0.8 mL/min, 254 nm.

Racemate:

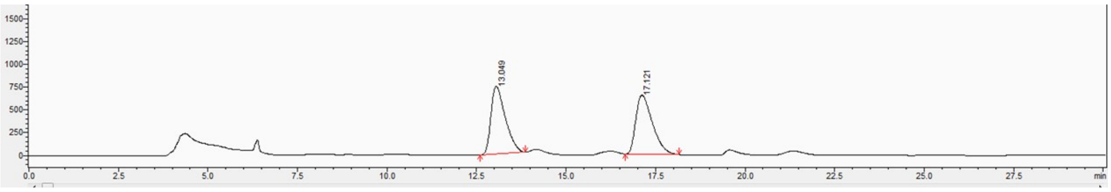

	Retention Time (min)	Area (%)
Peak 1	13.049	49.861
Peak 2	17.121	50.139

Chiral:

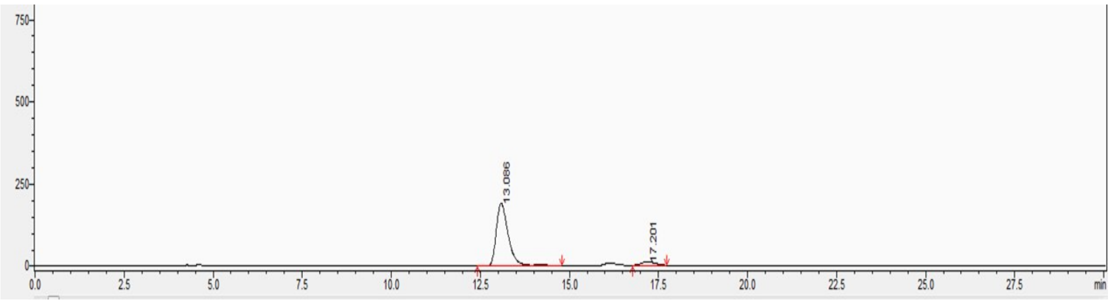

	Retention Time (min)	Area (%)	
Peak 1	13.086	93.692	**87% ee**
Peak 2	17.201	6.308	

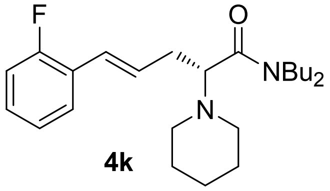

4k

84% *ee*, enantiomeric excess was determined by HPLC, Daicel Chiralpak IC-3 column, *n*-hexane/*i*-PrOH = 95/5, 0.8 mL/min, 254 nm.

Racemate:

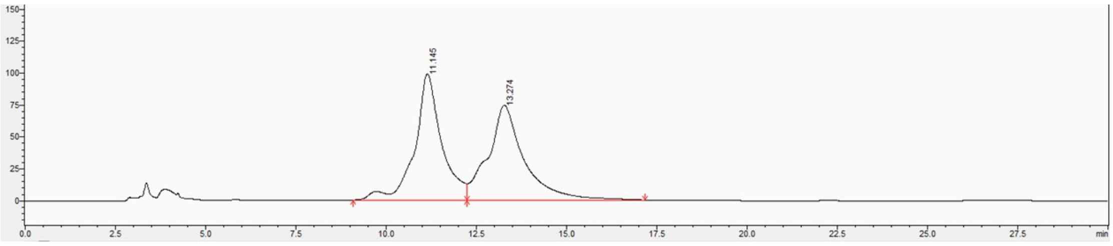

	Retention Time (min)	Area (%)
Peak 1	11.145	49.964
Peak 2	13.274	50.036

Chiral:

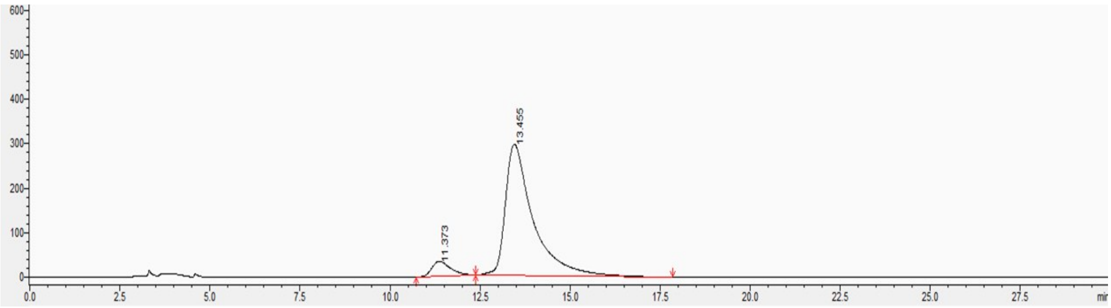

	Retention Time (min)	Area (%)	
Peak 1	11.373	7.947	**84% ee**
Peak 2	13.455	92.053	

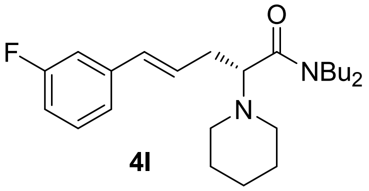

4l

91% *ee*, enantiomeric excess was determined by HPLC, Daicel Chiralpak IC-3 column, *n*-hexane/*i*-PrOH = 95/5, 0.8 mL/min, 254 nm.

Racemate:

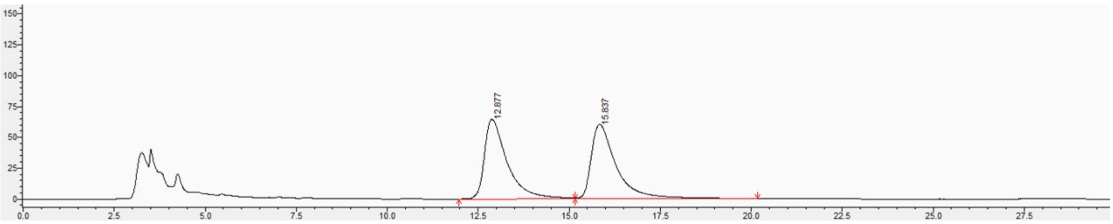

	Retention Time (min)	Area (%)
Peak 1	12.877	50.973
Peak 2	15.837	49.027

Chiral:

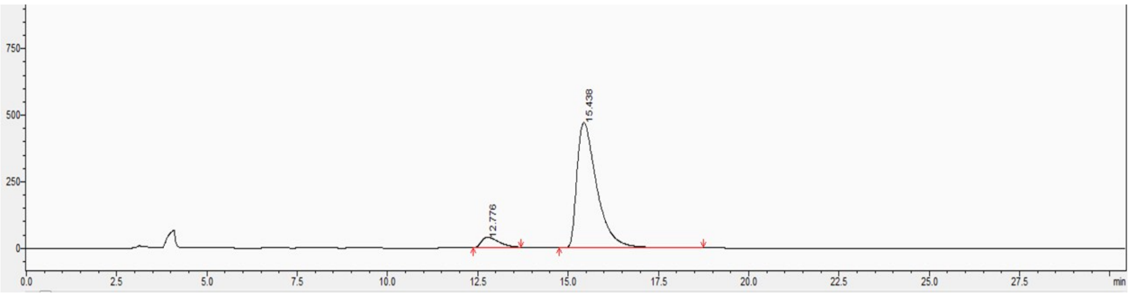

	Retention Time (min)	Area (%)	
Peak 1	12.776	4.577	**91% ee**
Peak 2	15.438	95.423	

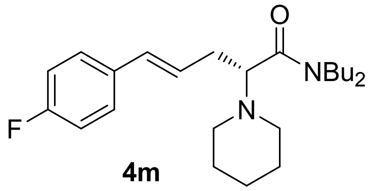

4m

93% *ee*, enantiomeric excess was determined by HPLC, Daicel Chiralpak IC-3 column, *n*-hexane/*i*-PrOH = 95/5, 0.8 mL/min, 254 nm.

Racemate:

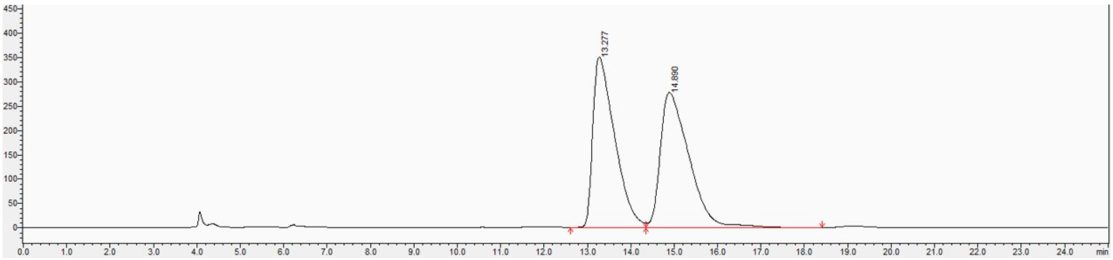

	Retention Time (min)	Area (%)
Peak 1	13.277	49.015
Peak 2	14.890	50.985

Chiral:

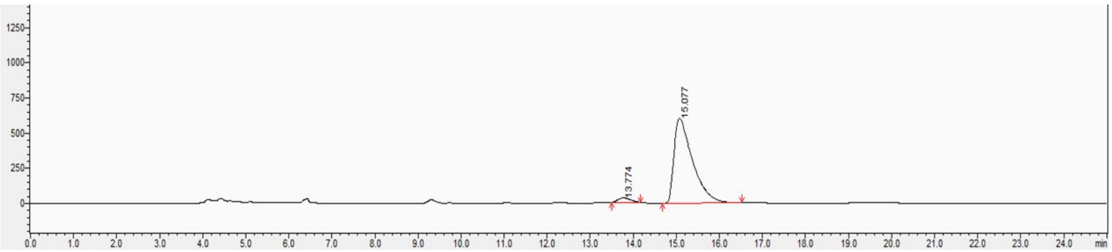

	Retention Time (min)	Area (%)	
Peak 1	13.774	3.597	**93% ee**
Peak 2	15.077	96.403	

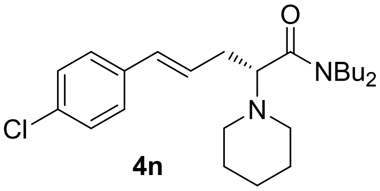

4n

92% *ee*, enantiomeric excess was determined by HPLC, Daicel Chiralpak IC-3 column, *n*-hexane/*i*-PrOH = 95/5, 0.4 mL/min, 254 nm.

Racemate:

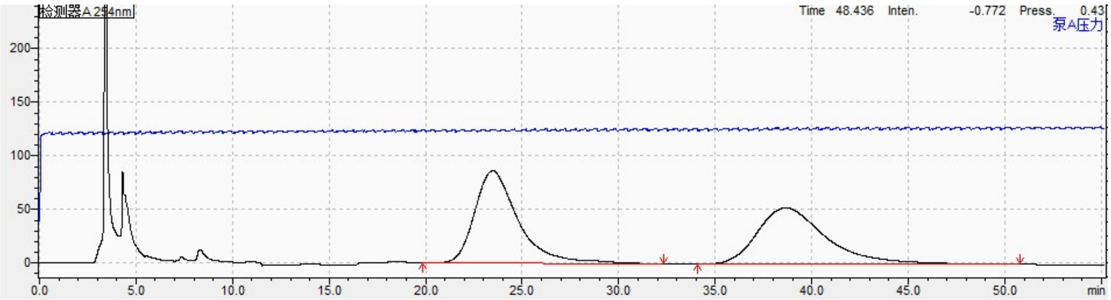

	Retention Time (min)	Area (%)
Peak 1	23.481	49.729
Peak 2	38.651	50.271

Chiral:

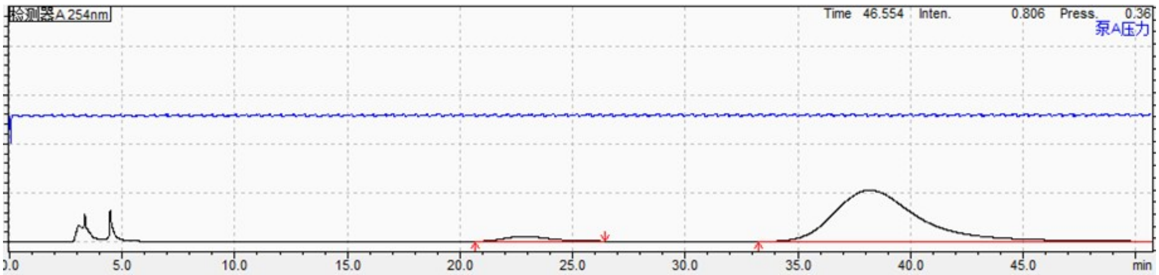

	Retention Time (min)	Area (%)	
Peak 1	22.855	4.014	**92% ee**
Peak 2	38.173	95.986	

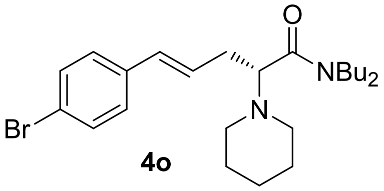

4o

93% *ee*, enantiomeric excess was determined by HPLC, Daicel Chiralpak IC-3 column, *n*-hexane/*i*-PrOH = 95/5, 0.8 mL/min, 254 nm.

Racemate:

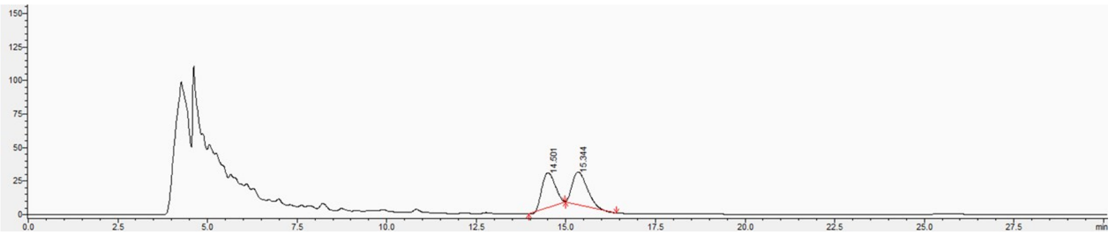

	Retention Time (min)	Area (%)
Peak 1	14.501	48.860
Peak 2	15.344	51.140

Chiral:

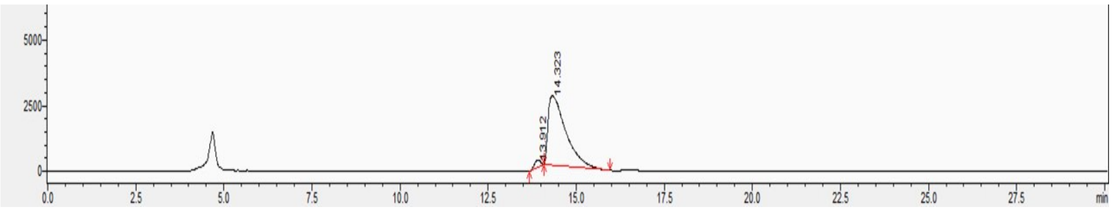

	Retention Time (min)	Area (%)	
Peak 1	17.077	3.543	**93% ee**
Peak 2	25.302	96.457	

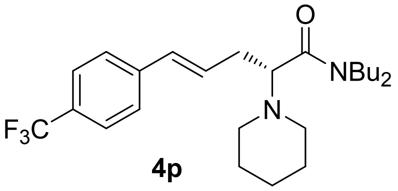

4p

96% *ee*, enantiomeric excess was determined by HPLC, Daicel Chiralpak AD-H column, *n*-hexane/*i*-PrOH = 99.5/0.5, 0.8 mL/min, 254 nm.

Racemate:

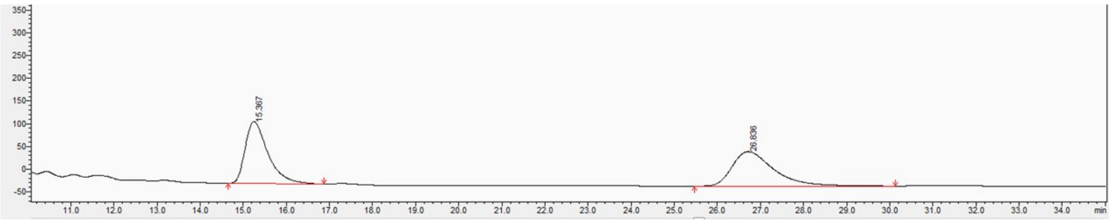

	Retention Time (min)	Area (%)
Peak 1	15.367	49.661
Peak 2	26.836	50.339

Chiral:

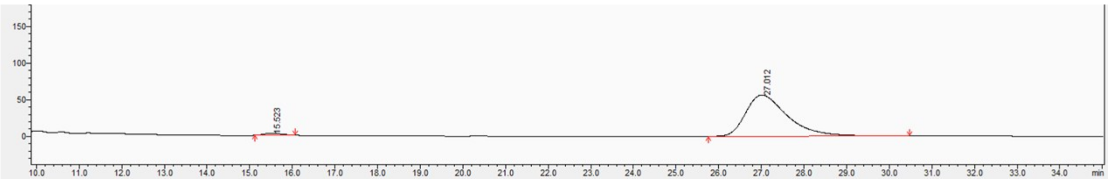

	Retention Time (min)	Area (%)	
Peak 1	15.523	2.065	**96% ee**
Peak 2	27.012	97.935	

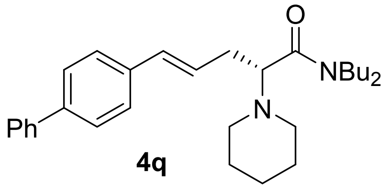

4q

88% *ee*, enantiomeric excess was determined by HPLC, Daicel Chiralpak IC-3 column, *n*-hexane /*i*-PrOH = 95/5, 0.8 mL/min, 254 nm.

Racemate:

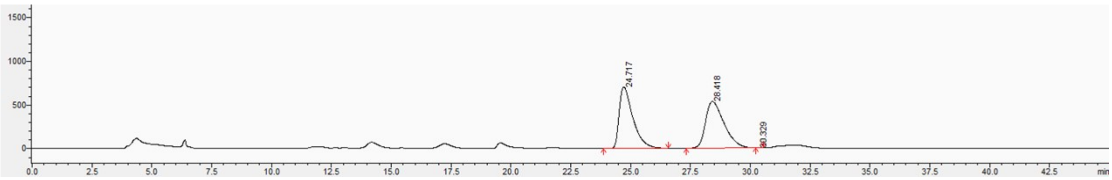

	Retention Time (min)	Area (%)
Peak 1	24.717	49.176
Peak 2	28.418	50.824

Chiral:

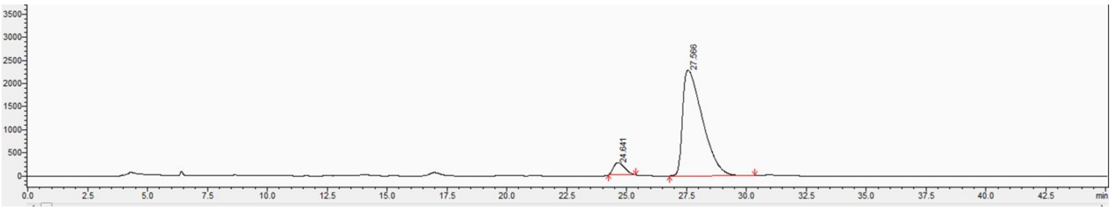

	Retention Time (min)	Area (%)	
Peak 1	24.641	6.073	**88% ee**
Peak 2	27.566	93.927	

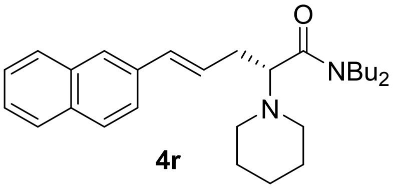

4r

91% *ee*, enantiomeric excess was determined by HPLC, Daicel Chiralpak AD-H column, *n*-hexane /*i*-PrOH = 99/1, 1 mL/min, 254 nm.

Racemate:

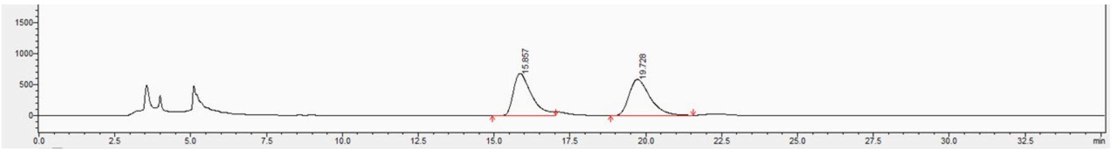

	Retention Time (min)	Area (%)
Peak 1	15.857	50.233
Peak 2	19.728	49.767

Chiral:

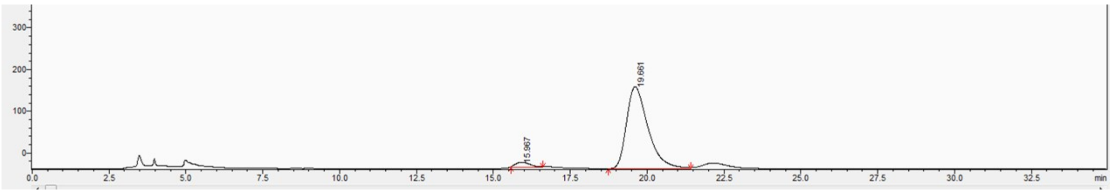

	Retention Time (min)	Area (%)	
Peak 1	15.967	4.469	**91% ee**
Peak 2	19.661	95.531	

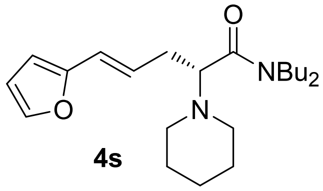

4s

95% *ee*, enantiomeric excess was determined by HPLC, Daicel Chiralpak IC-3 column, *n*-hexane /*i*-PrOH = 95/5, 0.8 mL/min, 254 nm.

Racemate:

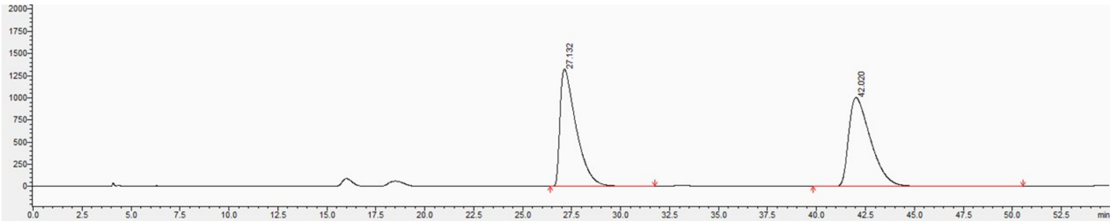

	Retention Time (min)	Area (%)
Peak 1	27.132	49.241
Peak 2	42.020	50.759

Chiral:

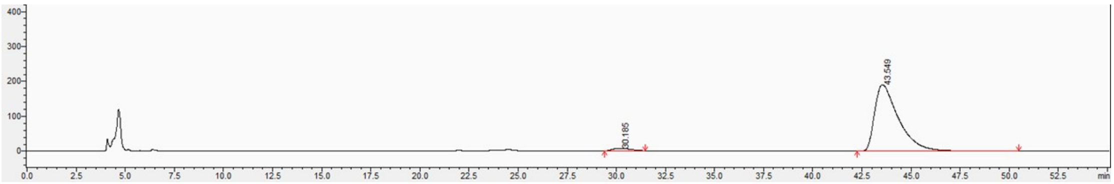

	Retention Time (min)	Area (%)	
Peak 1	30.185	2.728	**95% ee**
Peak 2	43.549	97.272	

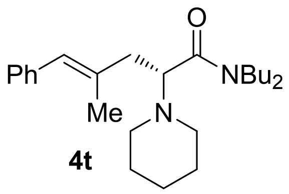

4t

57% *ee*, enantiomeric excess was determined by HPLC, Daicel Chiralpak IC-3 column, *n*-hexane /*i*-PrOH = 97/3, 0.8 mL/min, 254 nm.

Racemate:

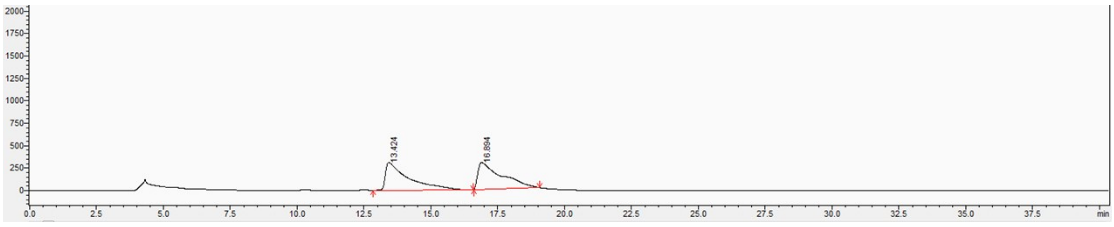

	Retention Time (min)	Area (%)
Peak 1	13.424	50.730
Peak 2	16.894	49.270

Chiral:

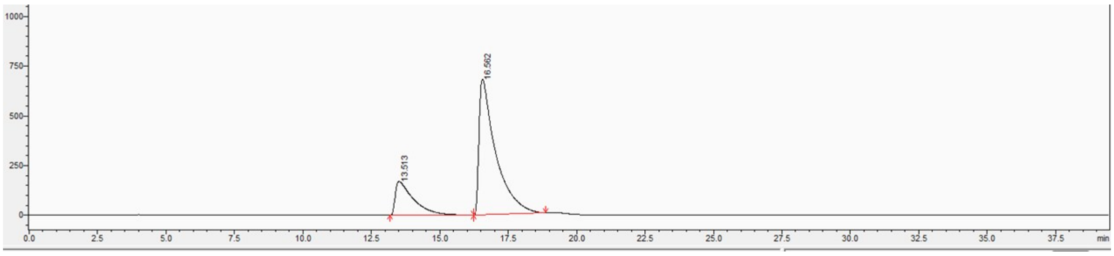

	Retention Time (min)	Area (%)	
Peak 1	13.513	21.427	**57% ee**
Peak 2	16.562	78.573	

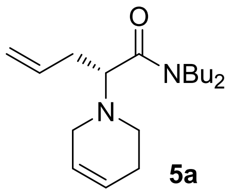

5a

80% *ee*, enantiomeric excess was determined by HPLC, Daicel Chiralpak IC-3 column, *n*-hexane /*i*-PrOH = 95/5, 0.8 mL/min, 210 nm.

Racemate:

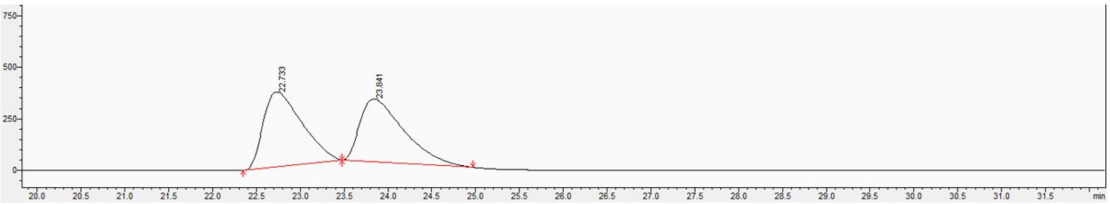

	Retention Time (min)	Area (%)
Peak 1	22.733	51.047
Peak 2	23.841	48.953

Chiral:

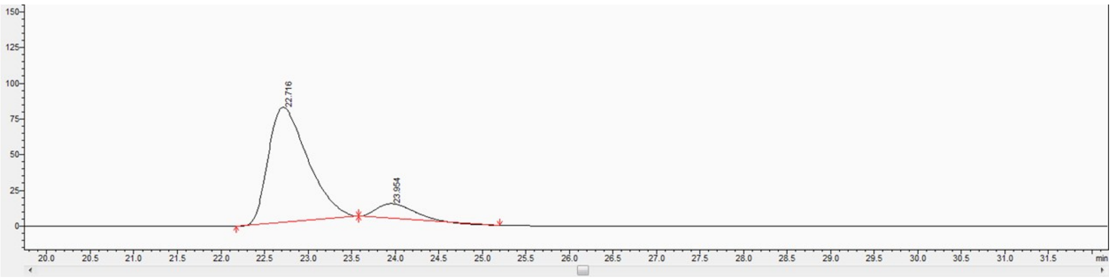

	Retention Time (min)	Area (%)	
Peak 1	22.716	89.816	**80% ee**
Peak 2	23.954	10.184	

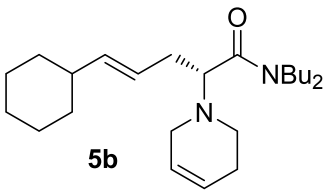

5b

71% *ee*, enantiomeric excess was determined by HPLC, Daicel Chiralpak IC-3 column, *n*-hexane /*i*-PrOH = 98/2, 0.8 mL/min, 210 nm.

Racemate:

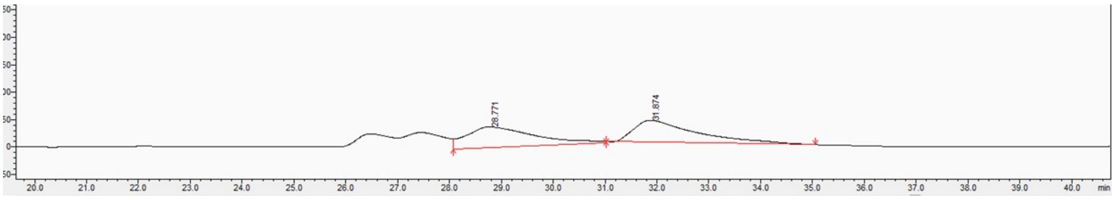

	Retention Time (min)	Area (%)
Peak 1	28.771	50.626
Peak 2	31.874	49.374

Chiral:

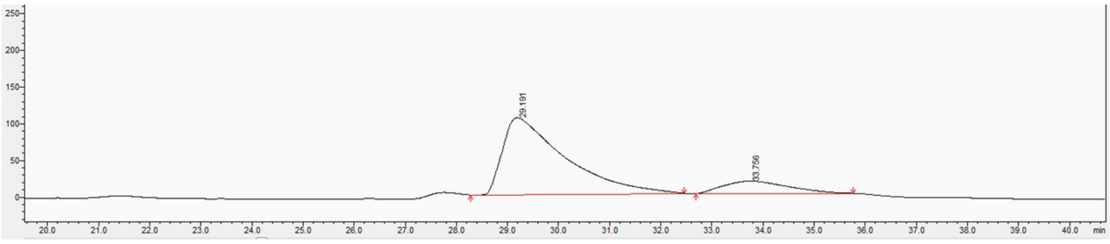

	Retention Time (min)	Area (%)	
Peak 1	29.191	85.607	**71% ee**
Peak 2	33.756	14.393	

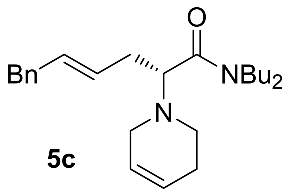

5c

68% *ee*, enantiomeric excess was determined by HPLC, Daicel Chiralpak IC-3 column, *n*-hexane /*i*-PrOH = 98/2, 0.8 mL/min, 210 nm.

Racemate:

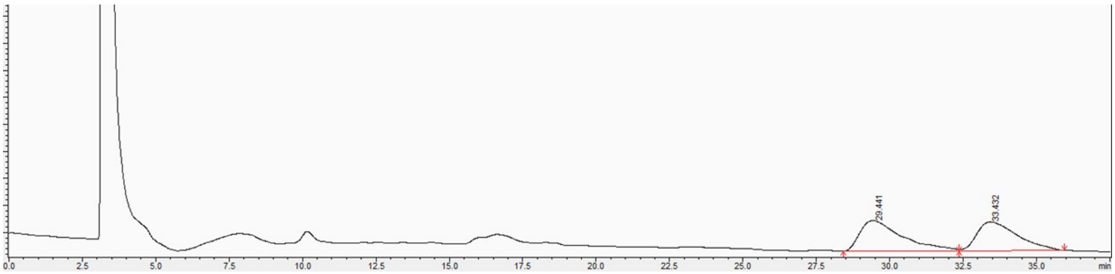

	Retention Time (min)	Area (%)
Peak 1	29.441	51.587
Peak 2	33.432	48.413

Chiral:

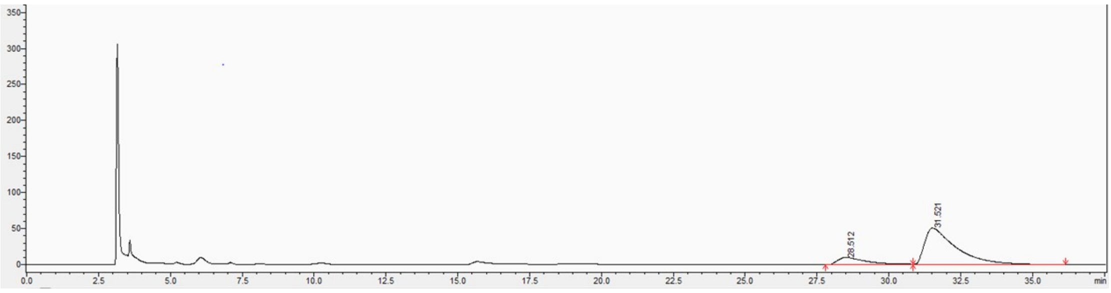

	Retention Time (min)	Area (%)	
Peak 1	28.512	16.026	**68% ee**
Peak 2	31.521	83.974	

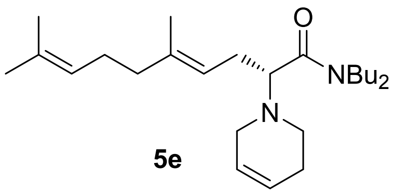

5e

24% *ee*, enantiomeric excess was determined by HPLC, Daicel Chiralpak IC-3 column, *n*-hexane /*i*-PrOH = 97/3, 0.8 mL/min, 210 nm.

Racemate:

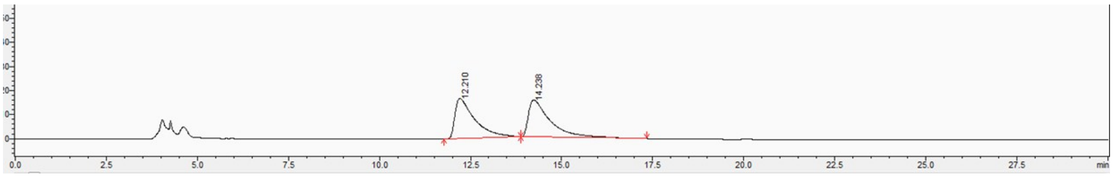

	Retention Time (min)	Area (%)
Peak 1	12.210	49.386
Peak 2	14.238	50.614

Chiral:

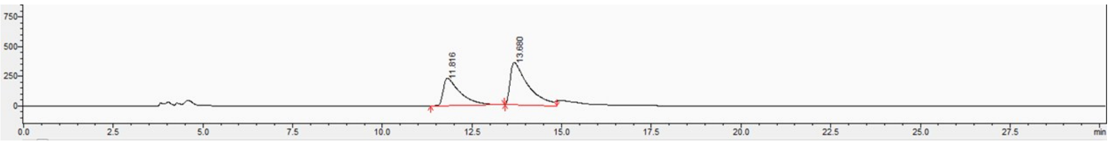

	Retention Time (min)	Area (%)	
Peak 1	11.816	38.002	**24% ee**
Peak 2	13.680	61.998	

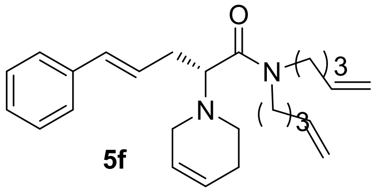

5f

90% *ee*, enantiomeric excess was determined by HPLC, Daicel Chiralpak AD-H column, *n*-hexane /*i*-PrOH = 98/2, 1 mL/min, 254 nm.

Racemate:

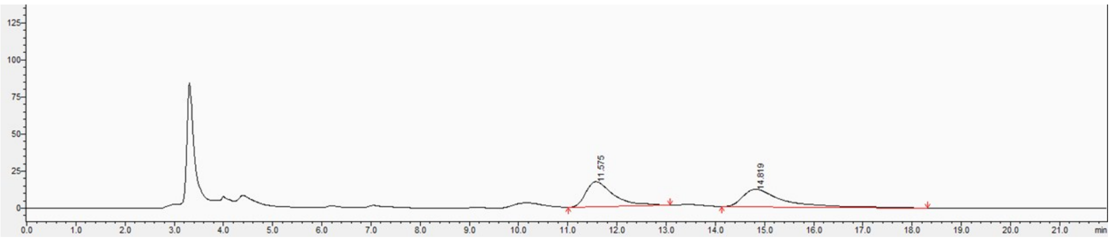

	Retention Time (min)	Area (%)
Peak 1	11.575	50.253
Peak 2	14.819	49.747

Chiral:

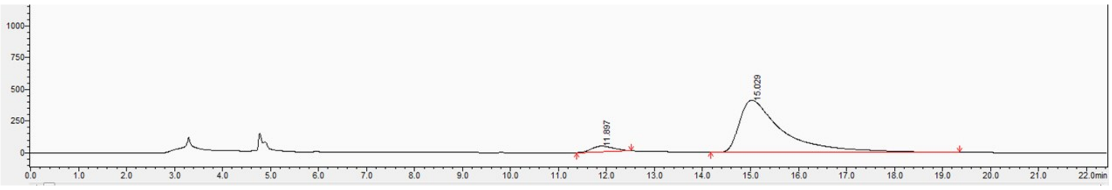

	Retention Time (min)	Area (%)	
Peak 1	11.897	5.215	**90% ee**
Peak 2	15.029	94.785	

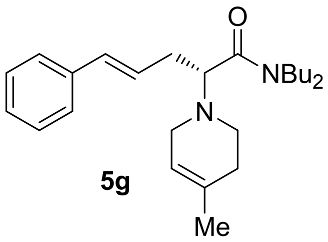

5g

93% *ee*, enantiomeric excess was determined by HPLC, Daicel Chiralpak IC-3 column, *n*-hexane /*i*-PrOH = 95/5, 0.8 mL/min, 254 nm.

Racemate:

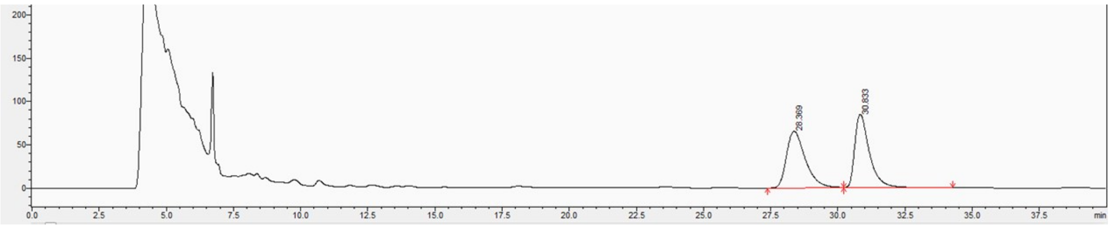

	Retention Time (min)	Area (%)
Peak 1	28.369	49.687
Peak 2	30.833	50.313

Chiral:

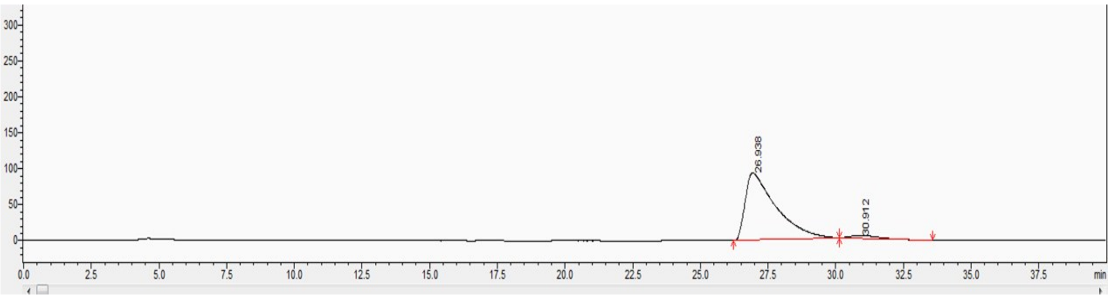

	Retention Time (min)	Area (%)	
Peak 1	26.938	96.530	**93% ee**
Peak 2	30.912	3.470	

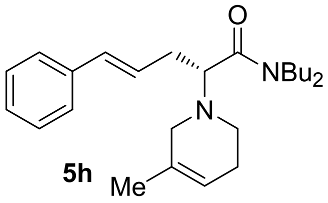

84% *ee*, enantiomeric excess was determined by HPLC, Daicel Chiralpak IC-3 column, *n*-hexane /*i*-PrOH = 95/5, 0.8 mL/min, 254 nm.

Racemate:

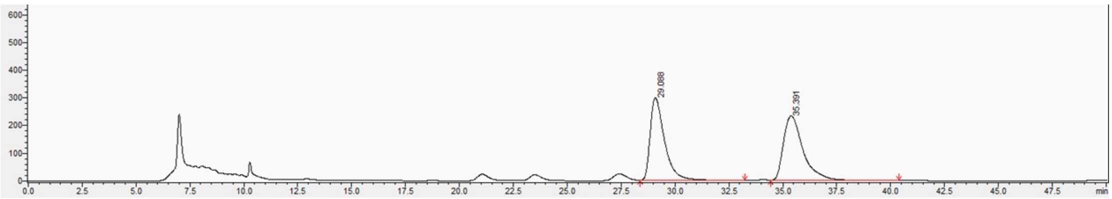

	Retention Time (min)	Area (%)
Peak 1	29.088	48.596
Peak 2	35.391	51.404

Chiral:

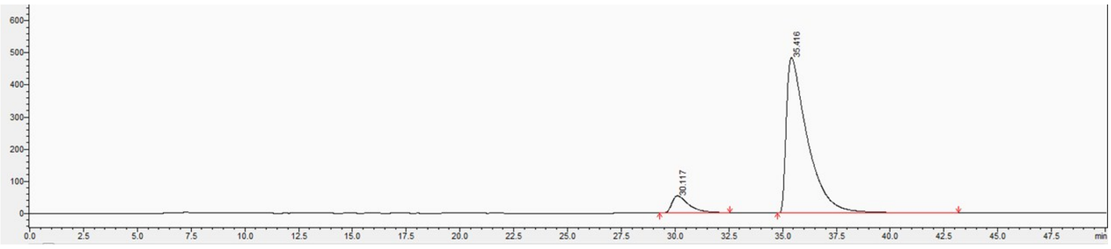

	Retention Time (min)	Area (%)	
Peak 1	30.117	8.147	**84% ee**
Peak 2	35.416	91.853	

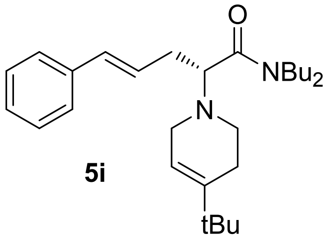

5i

90% *ee*, enantiomeric excess was determined by HPLC, Daicel Chiralpak IC-3 column, *n*-hexane /*i*-PrOH = 95/5, 1 mL/min, 254 nm.

Racemate:

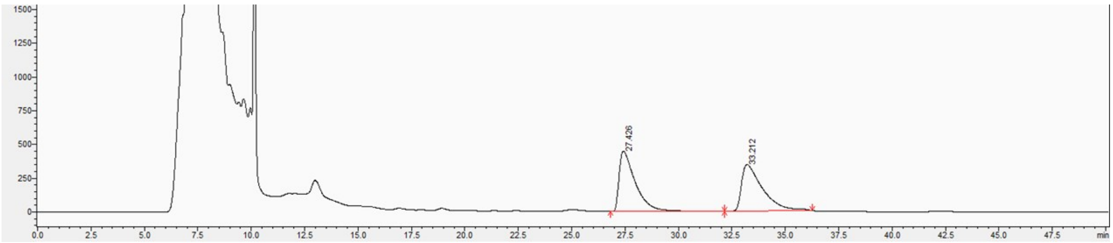

	Retention Time (min)	Area (%)
Peak 1	27.426	50.017
Peak 2	33.212	49.983

Chiral:

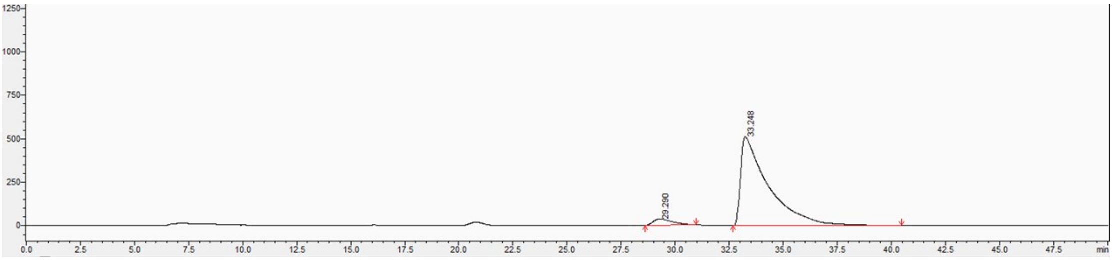

	Retention Time (min)	Area (%)	
Peak 1	29.290	4.801	**90% ee**
Peak 2	33.248	95.199	

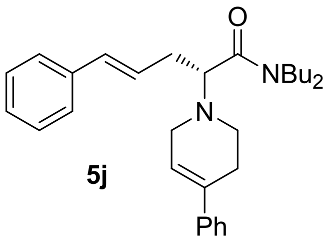

5j

88% *ee*, enantiomeric excess was determined by HPLC, Daicel Chiralpak IC-3 column, *n*-hexane /*i*-PrOH = 95/5, 1 mL/min, 254 nm.

Racemate:

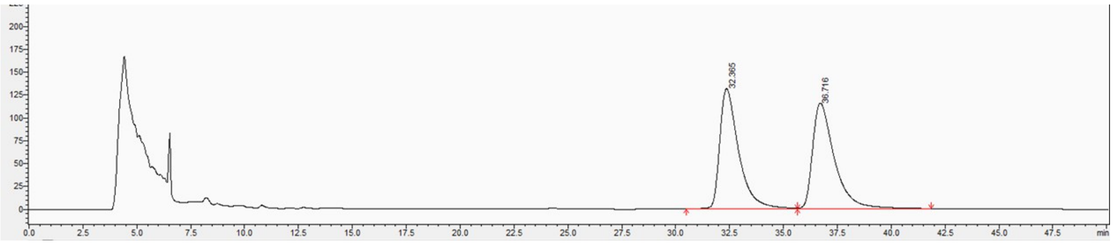

	Retention Time (min)	Area (%)
Peak 1	32.365	49.867
Peak 2	36.716	50.133

Chiral:

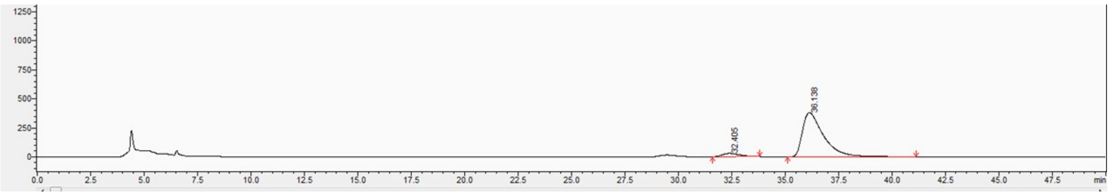

	Retention Time (min)	Area (%)	
Peak 1	32.405	5.962	**88% ee**
Peak 2	36.138	94.038	

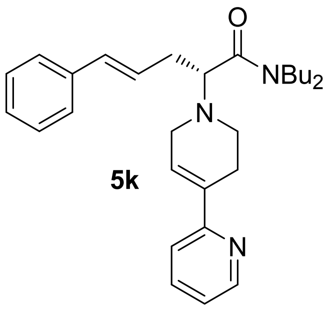

5k

91% *ee*, enantiomeric excess was determined by HPLC, Daicel Chiralpak AD-H column, *n*-hexane /*i*-PrOH = 98.5/1.5, 1 mL/min, 254 nm.

Racemate:

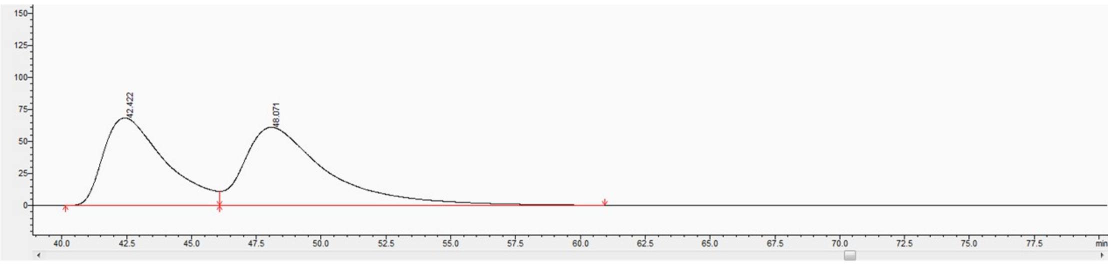

	Retention Time (min)	Area (%)
Peak 1	42.422	48.735
Peak 2	48.071	51.265

Chiral:

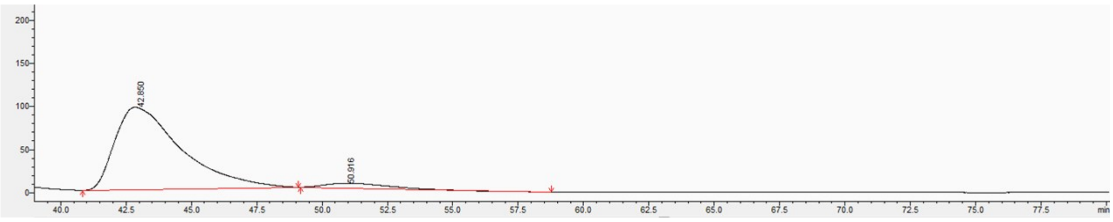

	Retention Time (min)	Area (%)	
Peak 1	42.850	95.371	**91% ee**
Peak 2	50.916	4.629	

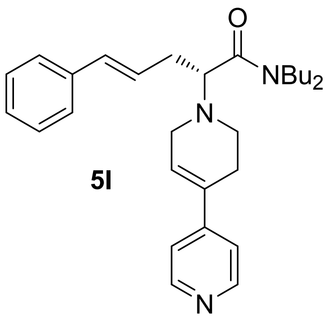

5l

92% *ee*, enantiomeric excess was determined by HPLC, Daicel Chiralpak AD-H column, *n*-hexane /*i*-PrOH = 95/5, 1 mL/min, 254 nm.

Racemate:

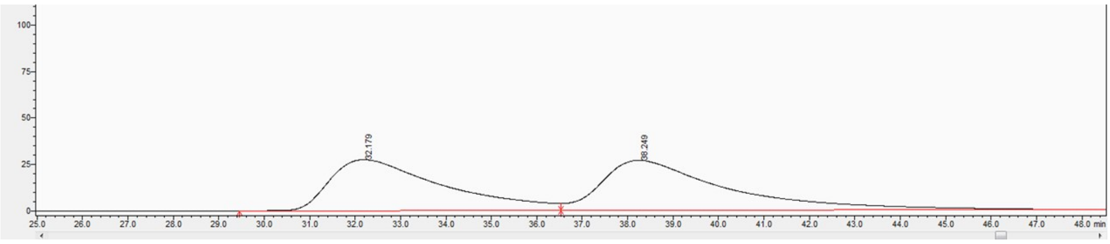

	Retention Time (min)	Area (%)
Peak 1	32.179	47.792
Peak 2	38.249	52.208

Chiral:

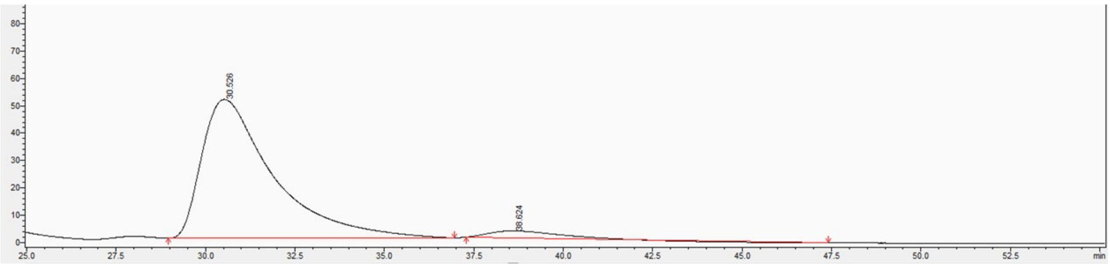

	Retention Time (min)	Area (%)	
Peak 1	30.526	95.910	**92% ee**
Peak 2	38.624	4.090	

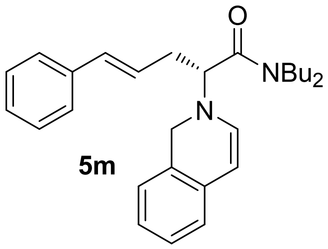

5m

88% *ee*, enantiomeric excess was determined by HPLC, Daicel Chiralpak IC-3 column, *n*-hexane /*i*-PrOH = 95/5, 0.8 mL/min, 254 nm.

Racemate:

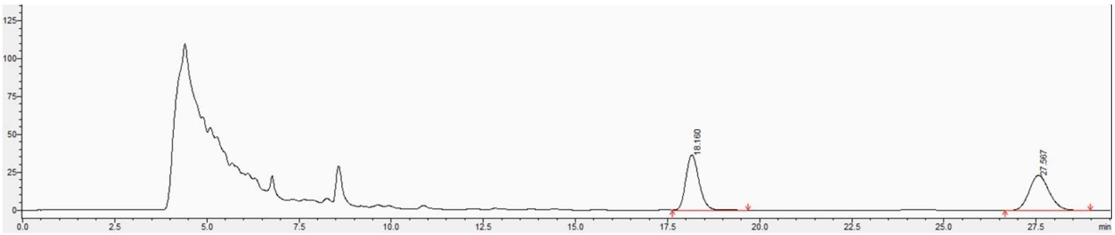

	Retention Time (min)	Area (%)
Peak 1	18.160	50.875
Peak 2	27.567	49.125

Chiral:

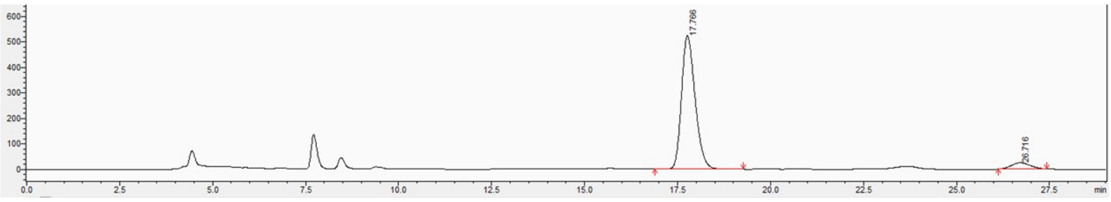

	Retention Time (min)	Area (%)	
Peak 1	17.766	93.794	**88% ee**
Peak 2	26.716	6.206	

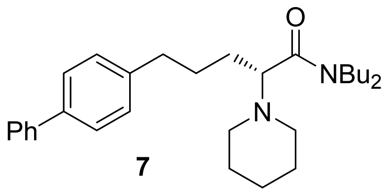

7

88% *ee*, enantiomeric excess was determined by HPLC, Daicel Chiralpak IC-3 column, *n*-hexane /*i*-PrOH = 95/5, 0.8 mL/min, 254 nm.

Racemate:

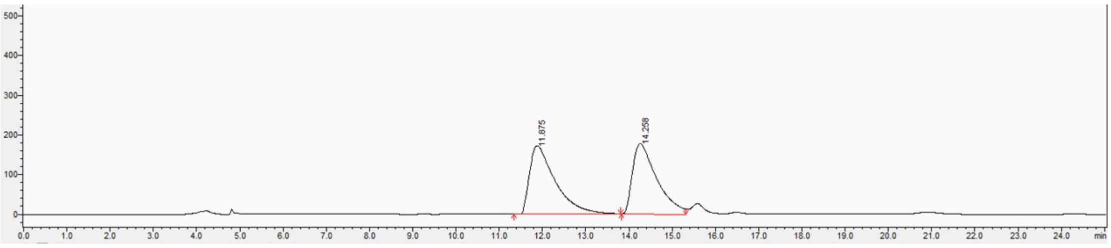

	Retention Time (min)	Area (%)
Peak 1	11.875	50.360
Peak 2	14.258	49.640

Chiral:

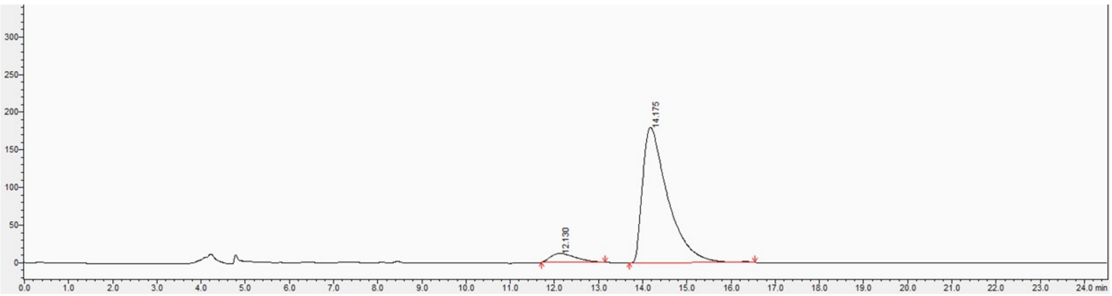

	Retention Time (min)	Area (%)	
Peak 1	12.130	6.148	**88% ee**
Peak 2	14.175	93.852	

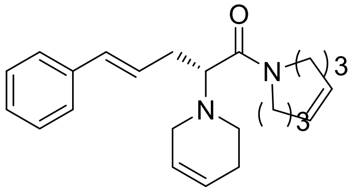

8

90% *ee*, enantiomeric excess was determined by HPLC, Daicel Chiralpak AD-H column, *n*-hexane /*i*-PrOH = 98/2, 0.8 mL/min, 254 nm.

Racemate:

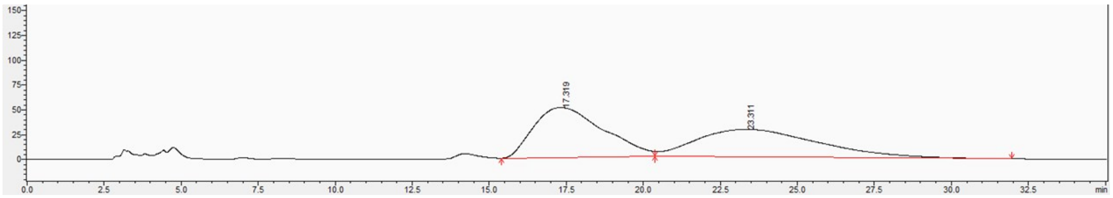

	Retention Time (min)	Area (%)
Peak 1	17.319	51.198
Peak 2	23.311	48.802

Chiral:

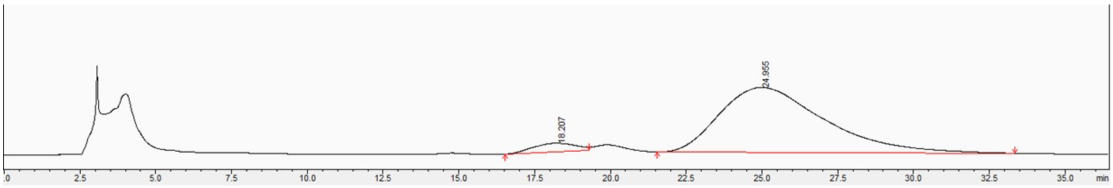

	Retention Time (min)	Area (%)	
Peak 1	18.207	4.950	**90% ee**
Peak 2	24.955	95.050	

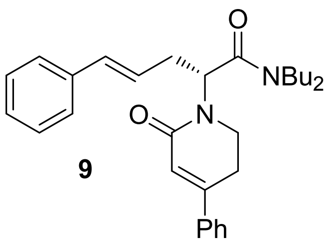

9

88% *ee*, enantiomeric excess was determined by HPLC, Daicel Chiralpak AD-H column, *n*-hexane /*i*-PrOH = 95/5, 1 mL/min, 254 nm.

Racemate:

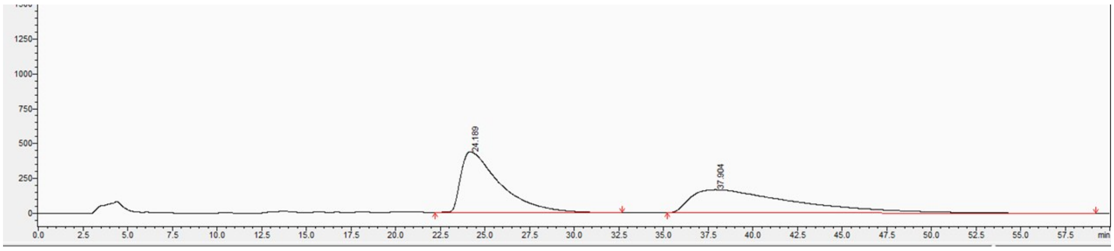

	Retention Time (min)	Area (%)
Peak 1	24.189	50.038
Peak 2	37.904	49.962

Chiral:

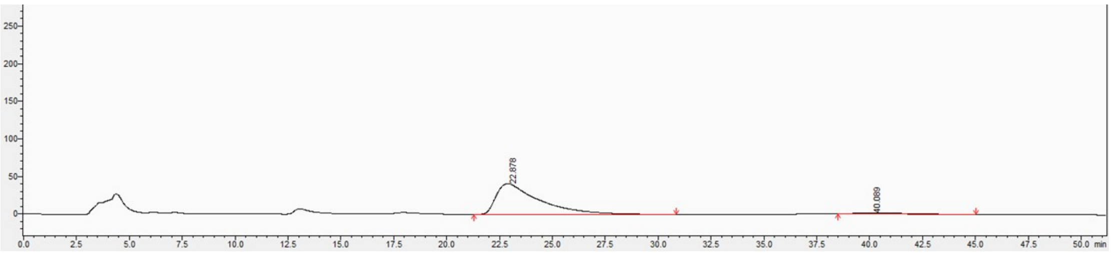

	Retention Time (min)	Area (%)	
Peak 1	22.878	94.076	**88% ee**
Peak 2	40.089	5.924	

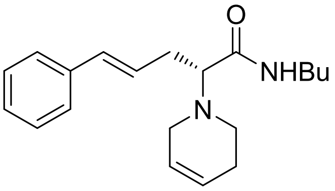

10

90% *ee*, enantiomeric excess was determined by HPLC, Daicel Chiralpak OD-H column, *n*-hexane /*i*-PrOH = 95/5, 1 mL/min, 254 nm.

Racemate:

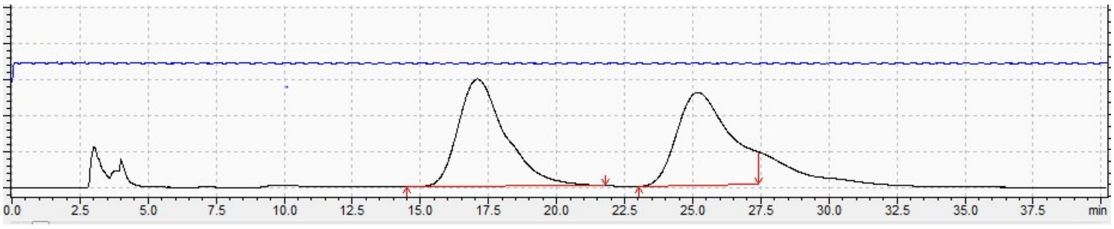

	Retention Time (min)	Area (%)
Peak 1	17.092	50.696
Peak 2	25.188	49.304

Chiral:

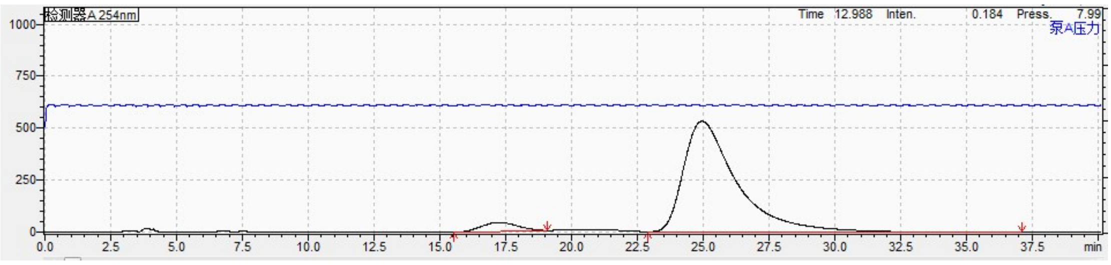

	Retention Time (min)	Area (%)	
Peak 1	17.239	5.169	**90% ee**
Peak 2	24.966	94.831	

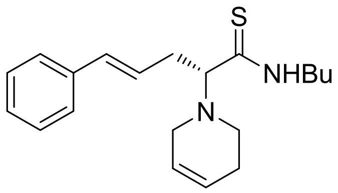

12

88% *ee*, enantiomeric excess was determined by HPLC, Daicel Chiralpak IC-3 column, *n*-hexane /*i*-PrOH = 95/5, 1 mL/min, 254 nm.

Racemate:

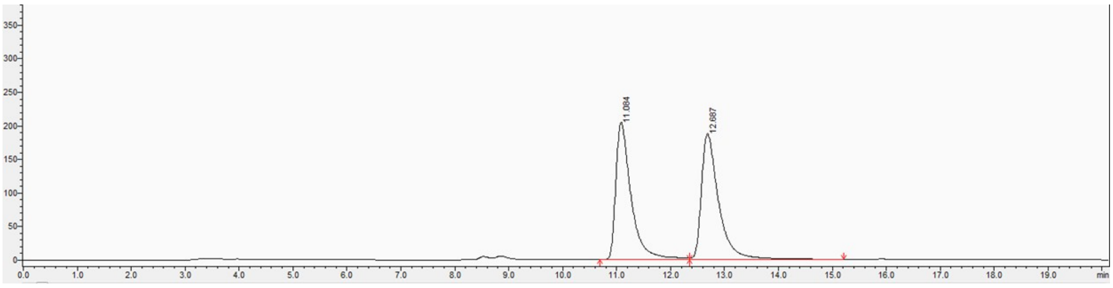

	Retention Time (min)	Area (%)
Peak 1	11.084	49.361
Peak 2	12.687	50.639

Chiral:

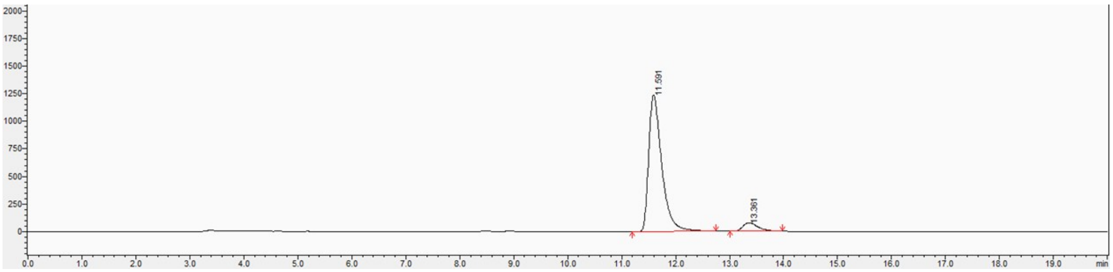

	Retention Time (min)	Area (%)	
Peak 1	11.591	94.016	**88% ee**
Peak 2	13.361	5.984	
